# Cluster randomized trial of comprehensive gender-based violence programming delivered through the HIV/AIDS program platform in Mbeya Region, Tanzania: Tathmini GBV study

**DOI:** 10.1371/journal.pone.0206074

**Published:** 2018-12-06

**Authors:** Susan K. Settergren, Stella Mujaya, Wasima Rida, Lusajo J. Kajula, Hussein Kamugisha, Jessie Kilonzo Mbwambo, Felix Kisanga, Mucho M. Mizinduko, Megan S. Dunbar, Isihaka Mwandalima, Hijja Wazee, Diana Prieto, Saiqa Mullick, Jennifer Erie, Delivette Castor

**Affiliations:** 1 Palladium, Washington, DC, United States of America; 2 Palladium, Dar es Salaam, Tanzania; 3 Consultant to Palladium, Washington, DC, United States of America; 4 Muhimbili University of Health and Allied Sciences, Dar es Salaam, Tanzania; 5 Pangaea Global AIDS, Oakland, California, United States of America; 6 Health for a Prosperous Nation, Dar es Salaam, Tanzania; 7 Walter Reed Program/Henry Jackson Foundation, Mbeya, Tanzania; 8 United States Agency for International Development, Washington, DC, United States of America; 9 Population Council, Johannesburg, South Africa; 10 United States Agency for International Development, Dar es Salaam, Tanzania; TNO, NETHERLANDS

## Abstract

The Tathmini GBV study was a cluster randomized trial to assess the impact of a comprehensive health facility- and community-based program delivered through the HIV/AIDS program platform on reduction in gender-based violence and improved care for survivors. Twelve health facilities and surrounding communities in the Mbeya Region of Tanzania were randomly assigned to intervention or control arms. Population-level effects were measured through two cross-sectional household surveys of women ages 15–49, at baseline (n = 1,299) and at 28 months following program scale-out (n = 1,250). Delivery of gender-based violence services was assessed through routine recording in health facility registers. Generalized linear mixed effects models and analysis of variance were used to test intervention effects on population and facility outcomes, respectively. At baseline, 52 percent of women reported experience of recent intimate partner violence. The odds of reporting experience of this violence decreased by 29 percent from baseline to follow-up in the absence of the intervention (time effect OR = 0.71, 95% CI: 0.57–0.89). While the intervention contributed an additional 15 percent reduction, the effect was not statistically significant. The program, however, was found to contribute to positive, community-wide changes including less tolerance for certain forms of violence, more gender equitable norms, better knowledge about gender-based violence, and increased community actions to address violence. The program also led to increased utilization of gender-based violence services at health facilities. Nearly three times as many client visits for gender-based violence were recorded at intervention (N = 1,427) compared to control (N = 489) facilities over a 16-month period. These visits were more likely to include provision of an HIV test (55.3% vs. 19.6%, p = .002). The study demonstrated the feasibility and impact of integrating gender-based violence and HIV programming to combat both of these major public health problems. Further opportunities to scale out GBV prevention and response strategies within HIV/AIDS service delivery platforms should be pursued.

**Trial Registration**: Pan African Clinical Trials Registry No. PACTR201802003124149.

## Introduction

Growing recognition of the association between gender-based violence (GBV) and HIV infection, particularly in sub-Saharan Africa where both prevalences are high, has motivated study of the pathways of association and demonstrated that the intersection of these two major global health problems is multi-dimensional and complex [1–4]. For example, coerced sex and other forms of sexual violence resulting in genital trauma may directly increase HIV exposure and transmission [5], while exposure to emotional abuse has been found to be associated with faster decline in markers of cell immunity [6]. GBV and HIV share common root causes grounded in power inequities, cultural norms, and gender inequalities [7–9]. Women in violent relationships often are less likely to be able to negotiate the frequency or circumstances of sex, thus exacerbating their HIV risk [10, 11]. Violence or the fear of violence, particularly in the form of intimate partner violence (IPV), has been shown to be a barrier to HIV prevention, care, and treatment services, and adherence to treatment regimens, thus limiting women’s access to life-saving treatment [12–16]. A woman’s HIV diagnosis also can exacerbate her experience of violence, for example, through accusations from her partner or family that she has had sex outside the partnership [17].

In spite of a growing understanding of the dynamics of GBV and HIV, less is known about how to prevent GBV among those living with and without HIV, particularly in low resource settings. Most intervention strategies have focused either on health care and secondary prevention for GBV survivors [18–21] or on efforts to curb violence at the community level [22–26]. Recent reviews of program research and evidence have highlighted the need for holistic programs that tackle GBV through multiple intervention channels at sufficient scale to achieve population level impacts [27–29].

In recognition of the need for accelerated, comprehensive programming on GBV and HIV, the U.S. President’s Emergency Plan for AIDS Relief (PEPFAR) in 2012 launched an initiative to strengthen GBV programming in Tanzania, Mozambique, and Democratic Republic of Congo [30]. As part of the initiative, PEPFAR commissioned an independent evaluation, the Tathmini GBV study, of the Walter Reed Program/Henry Jackson Foundation (WRP/HJFMRI) GBV program, a comprehensive GBV program delivered through HIV/AIDS prevention, care, and treatment platforms in the Mbeya Region of Tanzania. The primary aims of the Tathmini GBV study were to assess the program’s impact on prevalence of IPV at the population level and utilization of GBV services at health facilities.

## Methodology

### WRP/HJFMRI GBV program in Mbeya Region

Mbeya Region, located in the southwestern highlands of Tanzania, is largely agrarian with a population of 2.71 million and population density of 43 persons per km^2^ [31]. Forestry, industry, tourism, and mining serve as other key economic sources. In 2011, Mbeya Region ranked third highest among the 30 regions in Tanzania in HIV prevalence at 11.0 percent among females and 6.7 percent among males [32]. The region also ranked third highest in prevalence of IPV. Sixty-seven percent of ever-married women aged 15–49 years reported that they had ever experienced violence from a partner in comparison to the national prevalence of 50.2 percent [33].

WRP/HJFMRI, supported by the PEPFAR GBV initiative through the U.S. Department of Defense, developed a GBV program, which it scaled out through public health facilities and local community-based organizations that it also supported to strengthen delivery of HIV/AIDS services. The program included five components (given in Table 1) and was framed within a socioecological model that aimed to address GBV at individual, couples, institutional, community, and societal levels.

**Table 1:** WRP/HJFMRI GBV program.

Program component	Description	Implementation
GBV service delivery improvements at public health facilities	Program inputs included: • Orientation for council health management teams • Health care provider training • Medical equipment and supplies • Management support • Supportive supervision	GBV services were managed and delivered by established personnel at the facilities. WRP/HJFMRI program in partnership with Mbeya Regional Medical Office provided training and support to health facility managers and selected health care providers in accordance with national guidelines that call for the integration of GBV services within multiple points of entry at health facilities including departments for casualty or emergency, Prevention of Mother-to-Child Transmission, Reproductive and Child Health/Family Planning, HIV Testing and Counselling, HIV Care and Treatment, Antenatal Care, and Outpatient Care.
Community sensitization and dialogues	• Media and awareness raising events within communities under the campaign, “AMKA SASA,” adapted from SASA! [34] • Door-to-door education • Workshops with community and religious leaders	Organized and delivered by local community-based organizations (CBO) who were grantees of WRP/HJFMRI and who also delivered HIV interventions. Topics included GBV, violence against children (VAC), and gender norms/women’s rights.
Group education	• Based on Men As Partners curriculum [35] • Classes with pre-established groups on a regular basis • Participants encouraged to share knowledge with others	Led by peer educators trained by WRP/HJFMRI and managed by local CBO grantees. Multiple topics were addressed with a focus on gender norms utilizing a participatory learning approach.
Couples skills building	• CoupleConnect curriculum: 14-week course [36] • Well-respected couples in the communities invited to participate • Participants encouraged to share knowledge with and counsel others	Led by peer educators trained by WRP/HJFMRI and managed by local CBO grantees.
Building linkages among services	• Creation and facilitation of local GBV coordination committees at village, ward, district, and regional levels with membership from different sectors and aspects of community life; formal meetings at least quarterly • Referral of GBV survivors to and from health facilities and other services including police, local administrative officials, and legal services	Coordination committees established and trained by WRP/HJFMRI and subsequently facilitated by peer educators. Referral services provided by peer educators and managed by local CBO grantees.

The multicomponent program aimed to increase knowledge of GBV, decrease acceptance of GBV as a cultural norm, shift gender norms toward greater equity, strengthen community responses to GBV, and increase availability and access to GBV services. Layering of the program components was expected to lead to synergies among these intermediate outcomes, which in combination would lead to reduction in the prevalence of GBV and increase in use of GBV services. On the assumption that gender inequitable norms and GBV act as barriers to HIV/AIDS services, the GBV program was also hypothesized to lead to greater utilization of HIV/AIDS services. WRP/HJFMRI launched the program starting with GBV service delivery improvements at health facilities beginning in late 2012, followed by rollout of the other components starting in mid-2013. All components of the program were scaled out as of January 2014.

### Tathmini GBV study: Overall design

The Tathmini GBV study was designed as a matched-pair, cluster randomized trial to compare the effectiveness of the WRP/HJFMRI GBV program with standard practice. A cluster was defined as a public health facility (hospital or health center) together with the communities geographically surrounding the health facility that the facility regarded as its primary service area. Twelve facilities were selected from among the 18 district hospitals and health centers supported by WRP/HJFMRI that had served a minimum of 500 clients with HIV/AIDS services during the 6-month period prior to the start of the GBV program (i.e., April–September 2012). Criteria for selection included a minimum distance between facilities of 30 km by road that would also allow selection of an equal number of hospitals and health centers. Facilities were matched into six pairs based on facility type (hospital or health center), total annual client load, and number of clients provided with HIV/AIDS services in the most recent six months. Clusters within each pair were randomly assigned by the Mbeya Regional Medical Officer, facilitated by the study team, to treatment and control arms by drawing from a hat a piece of paper on which facility names had been written. The population size of each arm was approximately 150,000 people. Selection and assignment of the clusters took place on November 5, 2012, immediately prior to the launch of the first components of WRP/HJFMRI GBV program.

The study had two primary outcomes, recent experience of IPV and utilization of GBV services, measured through baseline and follow-up household surveys in the study communities and routine reporting of GBV services at the study health facilities, respectively. Secondary outcomes included prevalence of specific forms of GBV, women’s acceptance of IPV, knowledge about GBV, gender norms, community actions, availability and quality of GBV services, and utilization of HIV-related services. (See Table 2).

**Table 2:** Study outcome measures.

Outcome	Source	Measures
Experience of GBV	Household surveys	Primary outcome: Report of any intimate partner violence (IPV) in the past 12 months among females aged 15–49 with an intimate partner. This measure comprised a series of questions that referred to specific acts of sexual (2 questions), physical (6 questions), and emotional (3 questions) violence. These were standardized questions used in the Tanzania Demographic and Health Survey (TDHS) 2010 [33]. An individual was determined to have experienced any form of IPV if she reported that she had experienced at least one instance of any of these three types of violence from any of up to three intimate partners in the past 12 months. Secondary outcomes: Specific forms of IPV. Respondent reports of six partner controlling behaviors [33] and forced sex or physical GBV from someone other than a partner.
Utilization of GBV services	Health facilities + Household surveys	Primary outcome: Number of GBV client visits at health facilities (as reported by facilities). Secondary outcome: Survey respondent reports of use of health services for GBV in the past 12 months.
Acceptance of IPV	Household surveys	Secondary outcomes: Respondent reports of acceptance of IPV under six conditions that have been validated in other population-based surveys [33, 37].
Knowledge about GBV	Household surveys	Secondary outcomes: Respondent reports of familiarity with Tanzanian GBV laws and policies and beliefs about sexual violence among children.
Gender norms	Household surveys	Secondary outcomes: Respondent ratings on items from the “Violence” and “Domestic chores and daily life” domains of the Gender Equitable Men (GEM) scale. The Violence scale includes six statements, scaled 1–3, with a possible score range of 6–18, higher scores reflecting lower acceptance of partner violence. The Domestic Chores and Daily Life domain of the GEM scale includes 5 statements regarding women’s and men’s roles in the household, scaled 1–3 with a possible score range of 5–15. Higher scores indicate less traditional attitudes about gender household roles [38].
Community actions	Household surveys	Secondary outcomes: Respondent reports of personal actions and actions taken by others in the study communities to address GBV in the past 12 months.
Availability and quality of GBV services	Health facilities	Secondary outcomes: Number and percent of client visits by type of service provided as defined by national guidelines [39].
Utilization of HIV-related services	Household surveys	Secondary outcomes: Respondent reports of HIV testing and knowledge of availability of HIV PEP.

A study period of approximately 24 months was planned. However, unanticipated delays in scale-out of all components of the GBV program and funding limitations of the study resulted in 16 months of follow-up at the health facilities and 28 months of follow-up at the household level. Data collection was implemented under two different funding mechanisms. The household baseline survey (conducted May 14, 2013–June 16, 2013) and all facility data (reported for July 1, 2013–April 30, 2015) were collected under the first mechanism, while the follow-up household survey (conducted in April 13, 2016–May 13, 2016) was conducted under the second mechanism. WRP/HJFMRI began GBV program rollout at the health facilities in the control clusters in late May 2016.

The Tathmini GBV study received ethical approval from the following institutional review boards: Tanzania National Institute for Medical Research (Ref: NIMR/HQ/R.8a/Vol. IX/1502; March 5, 2013), Muhimbili University of Health and Allied Sciences (Ref: MU/DRP/AEC/Vol. XVI/83; February 4, 2013), Mbeya Medical Research and Ethics Committee (Ref: MRH/R. 10/8/Vol VI/105; March 15, 2013), and the Population Council (Protocol #570; December 6, 2012). The study protocol is provided as supporting information (see S1 File. Study protocol). The study is registered with the Pan African Clinical Trials Registry (No. PACTR201802003124149). The CONSORT checklist is provided as supporting information (see S1 Checklist. CONSORT checklist). Clinical trials registration was obtained subsequent to participant enrolment due to initial misclassification of the study as a non-clinical trial. The authors confirm that all ongoing and related trials for this intervention are registered.

### Household surveys

#### Survey tool

The survey questionnaire captured sociodemographic information about the respondent and household characteristics; respondent’s health, health behaviors, and sexual history; respondent’s intimate partnerships and characteristics for up to three intimate partners in the past 12 months; awareness of and participation in the WRP/HJFMRI GBV program; and information to derive the study outcome measures. The questionnaire was developed in English, translated into Kiswahili, and back translated (for the English version, see S1 File. Study protocol; for the Kiswahili version, see S2 File. Kiswahili questionnaire). The tool underwent three rounds of pilot tests prior to field data collection including an external pilot in Kiswahili in a community outside the study area.

#### Sampling

Multistage, stratified random sampling was used to select survey households and respondents. The same procedure was followed at baseline and follow-up. At the first stage, 10 enumeration areas (EAs) from the 2012 national population census were randomly sampled from among the EAs located within the geographic boundaries of each study cluster. At the second stage, one household was randomly selected from within each selected EA to serve as the starting point for the systematic selection of households to be visited for that EA. A household was defined as one or more individuals who usually lived and ate together, whether or not they were related by blood or marriage, with one person, male or female, acknowledged as the head of the household. Dwelling units, therefore, could have multiple households. At the third stage, one female within each selected household was randomly selected to be interviewed from among the eligible females in the household. All females aged 15–49 living in the household at the time of the survey were eligible for selection. This age group was selected given its high risk of GBV and HIV, and to facilitate comparison of results with other studies, such as the Tanzania Demographic and Health Survey [33]. If the selected person was not at home, the interviewer made at least two more attempts to reach her to conduct the interview.

A minimum sample size of 100 interviews per cluster was chosen. At 80 percent power using a two-sided 0.05 level test and assuming a within-pair coefficient of variation of 0.10, this sample size was estimated to be able to detect a 29 percent relative reduction in IPV, a primary study outcome [40]. This estimation was based on assumptions that at baseline 75 percent of respondents would have an intimate partner within the 12 months prior to the survey and that IPV prevalence in the past 12 months was 55 percent.

#### Field data collection

Three teams of four female interviewers conducted each survey. All interviewers had previous household survey experience, were internationally certified in research ethics, and underwent five days of study team-led training that included sessions on conducting GBV research [41, 42]. Survey teams conducted face-to-face interviews in Kiswahili using a paper-based form on which interviewers recorded respondent’s responses. Interviews were conducted in private settings (i.e., a location within the household dwelling or outside, nearby the household dwelling that provided visual and auditory privacy) and in accordance with World Health Organization guidelines for the safe and ethical collection of data on violence against women [41, 42]. Duration of interviews ranged from 60 to 90 minutes. At the conclusion of the interview, respondents were provided with contact information for the GBV focal person at the study health facility and counseled to contact this individual if they wanted assistance or more information about topics discussed in the interview.

#### Informed consent

Individuals selected for interview were informed of their rights as study participants and asked to provide written consent prior to the interview. Adult individuals (aged 18–49) provided informed consent. Written assent was obtained for minors (aged 15–17) after parental consent was obtained.

### Health facility data collection

Demographic information on clients for whom GBV services were provided, the types of GBV assessed, and the services and referrals provided were captured monthly on a paper-based register by facility service providers. The register was placed in all departments of the facility. A GBV focal person from among the providers was designated by each facility to oversee GBV register data recording and serve as the point of contact with the study team. The Tathmini GBV study team trained facility staff on the register and conducted quarterly supervisory visits to monitor data collection and collect de-identified copies of the register data.

### Quality assurance and data processing

Data were reviewed for completeness, legibility, and out-of-range values by the survey teams in the field and during quarterly supervisory visits to the facilities. All data were electronically double-keyed and discrepancies between twin-entries were resolved with reference to the paper-copy questionnaires. Additional range, logic, and consistency checks were performed. Errors for specific data items that could not be resolved (which occurred in at most 0.20% of cases for a given data item) were treated as missing in the analyses.

### Statistical analysis

Descriptive analyses were performed using SPSS Version 22 [43] on all data prior to fitting statistical models. For the baseline household survey, logistic regression models were used to assess the association between respondent characteristics and report of IPV among respondents who had an intimate partner in the 12 months prior to being surveyed. Characteristics included age, education, employment, marital status, sexual history, household characteristics, and characteristics of the current or most recent intimate partner. Factors associated with IPV at the 0.05 significance level were verified not to have changed significantly from the baseline to follow-up survey among this subsample that served as the basis for analysis of the primary IPV outcomes. Generalized linear mixed effects models (GLMM) were used to assess the differences in outcomes between intervention and control clusters from the follow-up household survey (intervention effect) and to assess changes in outcomes from baseline to follow-up in the absence of intervention (time effect) [44]. Cluster was treated as a random effect. Pair-matching of the clusters was ignored based on analysis of the baseline household survey that showed pairing the clusters did not reduce variability [45]. The models did not adjust for predictors of IPV. The effect of the intervention on GBV service utilization measured at health facilities was tested using analysis of variance (ANOVA) with a single explanatory variable for treatment group. Each cluster was treated as an observation with outcome measures represented by GBV client visit count (for the utilization measure), proportion of GBV client visits where a given service was provided (for the services measures and client characteristics), and cluster mean for continuous variables. All models were fit using Program R software Version 3.3.2 [46].

## Results

### Cluster and individual participant flow

**Fig 1:**
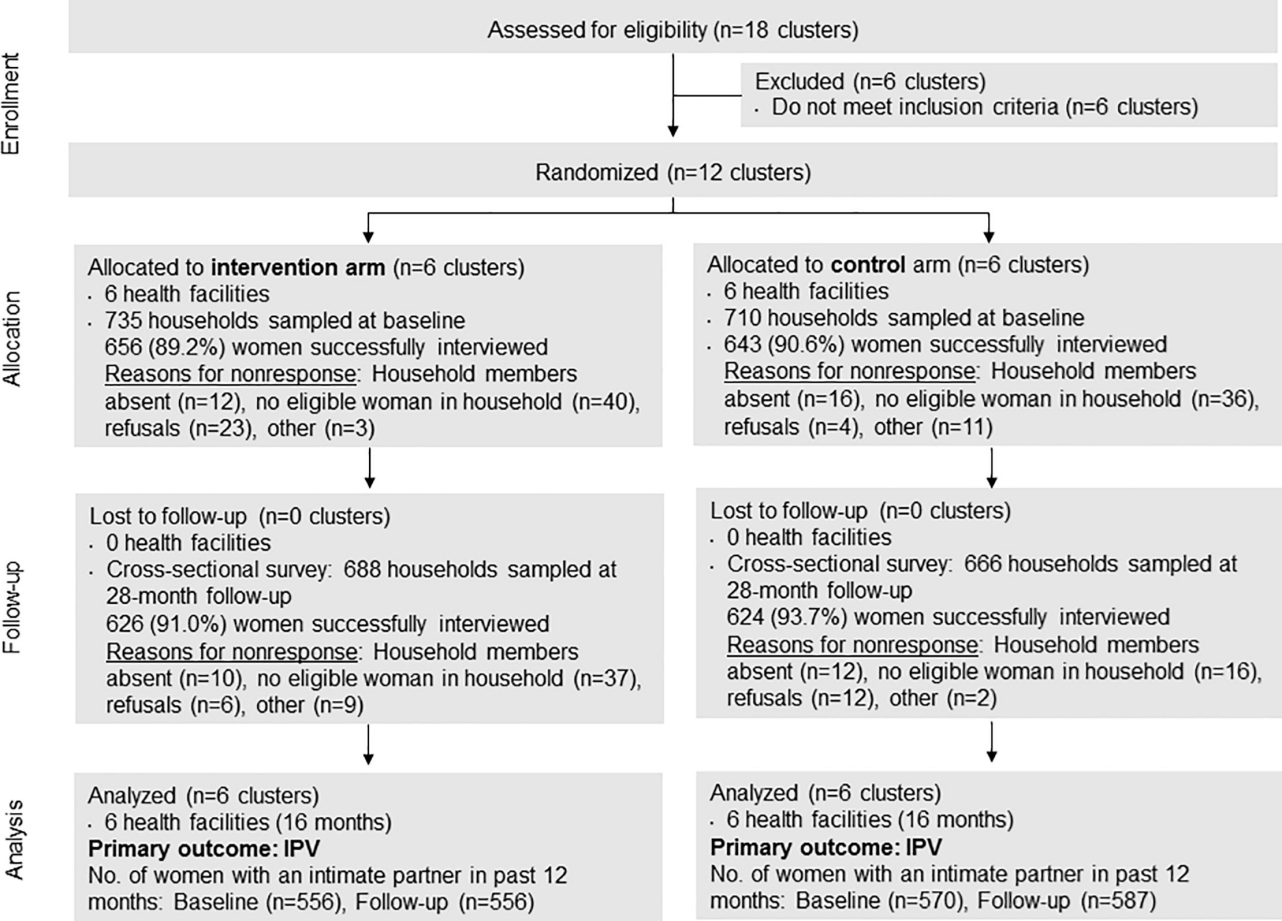
Progress of clusters and individuals through phases of the Tathmini GBV cluster randomized trial.

Results of enrollment, allocation, follow-up, and analysis of clusters and individuals are diagrammed in Fig 1.

### Survey respondent characteristics

Significant differences (p<0.05 or less) were observed between respondents at baseline and follow-up in age (mean of 29.3 and 30.4 years, respectively), currently married or living with a partner (71.6% vs. 77.5%), had an intimate partner in the past 12 months (86.7% vs. 91.4%), parity (82.8% vs. 88.6%), living in a household with piped water (30.7% vs. 37.7%) and with electricity (13.5% vs. 29.4%). Baseline prevalence of participants who had ever attended school (87.1%), worked outside of the home in the last 12 months (73.1%), and had more than one sexual partner in the past 12 months (4.3%) did not differ at follow-up. No statistically significant differences between the study arms were found with the exception that at follow-up control respondents were more likely to be currently married or living with a partner compared to respondents in the intervention arm (81.6% vs. 73.4%, p<0.001). (See Table 3).

**Table 3:** Survey respondent characteristics.

Age of respondent (years)	Baseline			Follow-up		
	Interv’n	Control	Both arms	Interv’n	Control	Both arms
N	656	642	1298	625	623	1248
Mean (SD)	29.0 (8.64	29.6 (8.58)	29.3 (8.61)***	30.4 (8.41)	30.4 (8.65)	30.4 (8.53)***
Median	28.0	29.0	28.0	30.0	30.0	30.0
**Characteristics**	**Percent** **Freq/N**	**Percent** **Freq/N**	**Percent** **Freq/N**	**Percent** **Freq/N**	**Percent** **Freq/N**	**Percent** **Freq/N**
Currently married or living with a partner	68.0 446/656	75.4 485/643	71.6*** 929/1298	73.4^†††^ 455/620	81.6^†††^ 507/621	77.5*** 962/1241
Had an intimate partner in the past 12 months	84.8 556/656	88.6 570/643	86.7*** 1126/1299	88.8 556/626	94.1 587/624	91.4*** 1143/1250
Had sexual intercourse with more than one partner in the past 12 months	4.1 27/651	4.5 29/643	4.3 56/1294	6.0 38/625	4.2 26/624	5.1 64/1249
Ever attended school	89.7 586/653	84.5 538/637	87.1 1124/1290	91.5 549/600	85.8 520/606	88.6 1069/1206
Has ever given birth	81.9 537/656	83.7 538/643	82.8*** 1075/1299	86.9 543/625	90.4 564/624	88.6*** 1107/1249
Worked outside the home in past 12 months	72.0 472/656	74.3 478/643	73.1 950/1299	69.7 434/623	72.0 448/622	70.8 882/1245
Main source of drinking water is piped water	28.4 186/654	33.0 212/643	30.7*** 398/1297	40.4 250/619	35.1 218/621	37.7*** 468/1240
Live in household with electricity	14.5 95/655	12.4 80/643	13.5*** 175/1298	35.6 223/626	23.1 144/624	29.4*** 367/1249

*** Difference between baseline and follow-up (both arms combined) based on likelihood ratio test from a GLMM with cluster as a random effect was statistically significant at p<0.001.

††† Difference between arms at follow-up based on likelihood ratio test from a GLMM with cluster as a random effect was statistically significant at p<0.001. Of note, this difference was not statistically significant among the subsample of those with an intimate partner in the past 12 months.

### Participation in the WRP/HJFMRI GBV program

At follow-up, 47.7 percent of women in the intervention arm reported they had heard of the WRP/HJFMRI GBV program, although only 4.6 percent reported they had participated in a community program launch event. The highest level of participation among WRP/HJFMRI GBV program components was in community dialogues (23.6%), followed by group education (14.3%) and CoupleConnect (5.6%). Some program awareness and participation was also reported among women in the control arm, but at levels significantly lower (p<0.05) than those in the intervention arm. There were, however, two exceptions. No difference was found among participation in CoupleConnect, where participation in both arms was low. And about half of women in both arms (55.9% in the intervention arm and 46.2% in the control arm) reported awareness of media programs on GBV in their communities. Women in the intervention arm, however, were more likely than those in the control arm to name the AMKA SASA media/community sensitization campaign program of the WRP/HJFMRI GBV program (12.2% vs. 4.3%, OR = 3.29, 95% CI: 1.02–10.6). (See Table 4).

**Table 4:** Awareness of and participation in GBV community interventions.

Percent of survey respondents who:	Follow-up		Estimated Odds Ratio (intervention relative to control arm)	95% CI	p value^a^
	Intervention	Control			
	Percent	Percent			
	Freq/N	Freq/N			
Heard about the WRP/HJFMRI GBV program	47.7%	19.6%	3.96	2.34–6.69	<0.001
	298/625	122/624			
Participated in a launch of the WRP/HJFMRI GBV program	4.6%	0.8%	6.08	1.67–22.19	0.006
	29/625	5/624			
Aware of current or recent GBV media programs	55.9%	46.2%	1.5	0.92–2.46	0.106
	348/622	288/623			
Mentioned awareness of AMKA SASA media campaign	12.2%	4.3%	3.29	1.02–10.6	0.046
	76/623	27/623			
Participated in community dialogues on GBV	23.6%	12.5%	2.18	1.40–3.41	<0.001
	147/622	78/624			
Participated in one or more GBV group education sessions	14.3%	4.7%	3.54	1.65–7.59	0.002
	89/622	29/623			
Participated in CoupleConnect	5.6%	1.3%	5.50	0.65–46.4	0.116
	35/621	8/621			

^*a*^ p values are based on a GLMM with cluster treated as a random effect.

### Experience of GBV

At baseline, 52.0 percent of women who had an intimate partner in the 12 months prior to the survey reported experience of any form of IPV in the past 12 months (48.6% and 55.3% in the intervention and control arms, respectively). At follow-up, prevalence of any form of IPV decreased in the intervention and control arms to 37.2 percent and 45.7 percent, respectively, as shown in Table 5. The odds of reporting any form of IPV decreased by 29 percent from baseline to follow-up in the absence of the intervention (time effect OR = 0.71, 95% CI: 0.57–0.89). A 15 percent difference between intervention and control arms in the odds of reporting IPV was observed at follow-up, but the effect was not statistically significant. Emotional IPV was the most prevalent form of IPV at both time points, followed by physical IPV, and sexual IPV. All forms were found to decrease over time in both arms, but the intervention effect approached statistical significance only for sexual IPV (intervention effect OR = 0.73, 85% CI: 0.51–1.05, p = 0.094). (See Table 5).

**Table 5:** Experience of IPV and other forms of GBV.

	Baseline		Follow-up		Odds-ratio of follow-up to baseline prevalence among control clusters (time effect)			Odds-ratio of intervention to control clusters prevalence at follow-up (intervention effect)		
	Interv’n	Control	Interv’n	Control	Est. OR	95% CI	p value^a^	Est. OR	95% CI	p value^a^
	Percent	Percent	Percent	Percent						
	Freq/N	Freq/N	Freq/N	Freq/N						
**Prevalence of reported IPV in past 12 months**										
Any form	48.6	55.3	37.2	45.7	0.71	0.57–0.89	0.004	0.85	0.62–1.16	0.302
	270/556	315/570	207/556	268/587						
Emotional	38.8	46.1	27.3	35.1	0.68	0.54–0.86	0.002	0.80	0.58–1.10	0.176
	216/556	263/570	152/556	206/587						
Physical	31.8	35.3	26.3	29.6	0.78	0.60–1.00	0.048	0.98	0.69–1.39	0.900
	177/556	201/570	146/556	174/587						
Sexual	20.9	23.0	12.8	17.0	0.72	0.55–0.94	0.016	0.73	0.51–1.05	0.094
	116/556	131/570	71/556	100/587						
**Prevalence of reported non-partner GBV in past 12 months**										
Physical GBV from someone other than partner	3.7	7.8	2.7	2.7	0.37	0.21–0.64	<0.001	1.69	0.75–3.80	0.204
	24/655	50/643	17/620	17/622						
Forced sex from someone other than partner	1.5	2.8	1.6	1.1	0.47	0.20–1.11	0.086	1.77	0.59–5.26	0.306
	10/655	18/643	10/622	7/624						
**Prevalence of reported controlling behaviors of partner in past 12 months**										
Experienced jealousy from partner	48.6	50.2	38.8	40.0	0.69	0.55–0.86	<0.001	0.97	0.76–1.38	0.870
	270/556	286/570	216/556	235/587						
Partner insisted on knowing where you are	45.1	45.8	38.3	35.6	0.68	0.54–0.86	0.002	1.11	0.80–1.53	0.550
	251/556	261/570	213/556	209/587						
Partner accused you of being unfaithful	26.8	29.8	22.5	20.8	0.66	0.51–0.86	0.002	1.20	0.84–1.72	0.308
	149/556	170/570	125/556	122/587						
Partner isolated you from friends	16.0	16.3	13.8	14.1	0.90	0.66–1.22	0.502	0.92	0.61–1.39	0.690
	89/556	93/570	77/556	83/587						
Partner controlled your use of money	15.1	14.9	9.7	11.6	0.80	0.58–1.09	0.154	0.82	0.54–1.26	0.368
	84/556	85/570	54/556	68/587						
Partner limited your contact with family	5.2	5.4	5.9	4.8	0.97	0.62–1.54	0.912	1.18	0.70–1.97	0.536
	29/556	31/570	33/556	28/587						

^a^ p values are based on a GLMM with cluster-specific baseline prevalence equal to the true baseline prevalence plus a random effect for all clusters including those randomized to the intervention.

Experience of GBV from a non-partner was reported at much lower rates. At baseline, 3.7 percent and 7.8 percent of intervention and control arms, respectively, reported experience of physical GBV from someone other than a partner. Prevalence in both arms fell to 2.7 percent at follow-up, but only a time effect was observed (time effect OR = 0.37, 95% CI = 0.21–0.64). No time or intervention effects were found for prevalence of reported experience of forced sex from someone other than a partner (1.5% in the intervention arm and 2.8% in the control arm at baseline).

About half of women at baseline reported experiencing at least one form of partner controlling behavior in the 12 months prior to the surveys. The most prevalent forms were jealousy (48.6% intervention, 50.2% control), insistence on knowing whereabouts (45.1% intervention, 45.8% control), and accused of being unfaithful (26.8% intervention, 29.8% control). Significant decreases were seen in reports of these behaviors over time, but no intervention effects were found. No time or intervention effects were seen for the other three partner controlling behaviors that were less prevalent at baseline: isolation from friends (16.0% intervention, 16.3% control), control of money (15.1% intervention, 14.9% control), and limited family contact (5.2% intervention, 5.4% control).

### Acceptance of IPV and gender norms

At baseline about half of respondents (53.4% intervention, 51.0% control) agreed that it is acceptable for a husband to hit or beat his wife if she neglects the children. Acceptance of partner violence in other situations was less prevalent: argues with her partner (44.7% intervention, 41.1%control), goes out without telling her partner (37.3% intervention, 35.8% control), refuses to have sex with her partner (35.4% intervention, 27.4% control), and burns the food (20.0% intervention, 20.4% control). Significant decreases in acceptance of partner violence were observed over time in the absence of the intervention with women at follow-up 22–34 percent less likely (depending on the situation) to report acceptance of husband’s violence (i.e., time effect ORs ranging from 0.78 to 0.66, all p<0.05). The intervention was found to reduce the odds of acceptance of hitting or beating for “refusal to have sex” by another 35 percent (i.e., intervention effect OR = 0.65, 95% CI: 0.46–0.91); no intervention effect was seen for the other reasons. (See Table 6).

**Table 6:** Acceptance of IPV.

	Baseline		Follow-up		Odds-ratio of follow-up to baseline prevalence among control clusters (time effect)			Odds-ratio of intervention to control clusters prevalence at follow-up (intervention effect)		
	Interv’n	Control	Interv’n	Control	Est. OR	95% CI	p value^a^	Est. OR	95% CI	p value^a^
	Percent	Percent	Percent	Percent						
	Freq/N	Freq/N	Freq/N	Freq/N						
**Prevalence of reported acceptance of a husband hitting or beating his wife in the following situations:**										
She neglects the children	53.4	51.0	41.8	45.5	0.78	0.63–0.97	0.028	0.81	0.60–1.09	0.170
	350/656	328/643	261/625	284/624						
She argues with her partner	44.7	41.1	29.3	32.7	0.66	0.53–0.84	<0.001	0.79	0.57–1.08	0.136
	293/656	264/643	183/625	204/624						
She goes out without telling her partner	37.3	35.8	27.7	29.5	0.73	0.57–0.92	0.008	0.89	0.65–1.24	0.500
	245/656	230/643	173/625	184/624						
She refuses to have sex with her partner	35.4	27.4	21.0	23.7	0.78	0.61–1.00	0.048	0.65	0.46–0.91	0.014
	232/656	176/643	131/625	148/624						
She burns the food	20.0	20.4	13.6	15.1	0.67	0.50–0.90	0.006	0.94	0.64–1.40	0.770
	131/656	131/643	85/625	94/624						

^a^ p values are based on a GLMM with cluster-specific baseline prevalence equal to the true baseline prevalence plus a random effect for all clusters including those randomized to the intervention.

Gender norms, as measured by the GEM scale Violence domain, were found to shift toward greater gender equity over time with mean scores increasing from 11.55 at baseline to 13.17 at follow-up in the intervention arm, and from 12.08 to 12.51 in the control arm. Statistically significant time and intervention effects were observed. A positive intervention effect (but not a time effect) was also found for the Domestic Chores and Daily Life domain (difference in average score between intervention and control clusters at follow-up = 1.26, 95% CI = 0.81–1.71). (See Table 7).

**Table 7:** Gender norms.

**GEM scale: Violence domain**	**Baseline**		**Follow-up**	
	**Intervention**	**Control**	**Intervention**	**Control**
N	652	641	624	621
Mean (SD)	11.55 (3.83)	12.08 (3.68)	13.17 (3.98)	12.51 (3.93)
		**95% Confidence Interval**		**p value**^a^
Baseline score (intervention + control)	11.81	11.31–12.31		-
Change in average GEM score in control clusters (time effect)	0.48	0.07–0.89		0.021
Difference in average GEM score between intervention and control clusters at follow-up (intervention effect)	1.08	0.52–1.65		<0.001
**GEM scale: Domestic chores and daily life domain**	**Baseline**		**Follow-up**	
	**Intervention**	**Control**	**Intervention**	**Control**
N	654	643	624	623
Mean (SD)	7.48 (2.69)	7.68 (2.82)	8.74 (3.63)	7.62 (3.14)
		**95% Confidence Interval**		**p value**^a^
Baseline score (intervention + control)	7.56	7.15–7.97		-
Change in average GEM score for control clusters (time effect)	-0.01	-0.34–0.31		0.933
Difference in average GEM score between intervention and control clusters at follow-up (intervention effect)	1.26	0.81–1.71		<0.001

^a^ p values are based on a GLMM model with cluster-specific baseline mean equal to the true baseline mean plus a random effect for all clusters including those randomized to intervention.

### Knowledge about GBV and violence against children

At baseline, 16.5 percent of women in the intervention arm and 17.8 percent in the control arm reported being very or somewhat familiar with Tanzania policies and laws regarding GBV and violence against children (VAC). At follow-up, this increased to 24.6 percent of women in the intervention arm, who were more than twice as likely to report familiarity with these laws and policies than women in the control arm (intervention effect OR = 2.71, 95% CI: 1.85–3.98), where reported familiarity declined from baseline to follow-up (time effect OR = 0.61, 95% CI: 0.45–0.83). Survey respondents were asked the extent to which they agreed with a series of statements reflecting misinformation about child sexual abuse. At baseline, nearly half of respondents agreed that only girls can be sexually abused (48.8% intervention, 41.7% control) and that a child is sexually abused only when sexual intercourse has taken place (46.6% intervention, 42.3% control). More than a third of respondents agreed that when a child is sexually abused, the abuser is rarely a family member (38.0% intervention, 37.5% control) and that it is not possible for children under 10 years of age to experience sexual abuse (35.2% intervention, 35.6% control). A quarter of respondents agreed that children from reputable families do not experience sexual abuse (24.7% intervention, 25.5% control). The intervention was found to decrease the odds of agreement with the misinformation by 32% to 49% among the four most commonly accepted statements. No changes in prevalence of misinformation over time were observed in the control arm. (See Table 8).

**Table 8:** Reported knowledge about GBV including sexual violence against children.

	Baseline		Follow-up		Odds-ratio of follow-up to baseline prevalence among control clusters (time effect)			Odds-ratio of intervention to control clusters prevalence at follow-up (intervention effect)		
	Interv’n	Control	Interv’n	Control	Est. OR	95% CI	p value^a^	Est. OR	95% CI	p value^a^
	Percent	Percent	Percent	Percent						
	Freq/N	Freq/N	Freq/N	Freq/N						
**Prevalence of reported knowledge**										
Familiarity with Tanzania laws and policies on GBV and VAC	16.5	17.8	24.6	11.7	0.61	0.45–0.83	0.002	2.71	1.85–3.98	<0.001
	108/655	115/642	154/623	73/624						
**Prevalence of reported agreement with the following statements about sexual violence against children**										
Only girls are sexually abused	48.8	41.7	34.7	45.2	1.11	0.89–1.39	0.340	0.52	0.38–0.70	<0.001
	319/653	268/643	217/625	282/623						
A child is sexually abused only when sexual intercourse has taken place	46.6	42.3	36.6	48.9	1.28	1.03–1.59	0.030	0.51	0.37–0.69	<0.001
	306/656	272/642	229/625	304/622						
When a child is sexually abused, the abuser is rarely a family member	38.0	37.5	30.4	39.6	1.09	0.88–.35	0.438	0.65	0.49– .88	0.004
	249/656	241/642	190/624	247/623						
It is not possible for children under 10 years of age to experience sexual abuse	35.2	35.6	27.7%	36.2	1.03	0.83–1.28	0.802	0.68	0.51–0.91	0.010
	230/653	229/643	173/624	226/623						
Children from reputable families do not experience sexual abuse	24.7	25.5	21.3	26.7	1.06	0.83–1.35	0.638	0.77	0.55–1.07	0.116
	162/656	164/643	133/623	166/622						

^a^ p values are based on a GLMM with cluster-specific baseline prevalence equal to the true baseline prevalence plus a random effect for all clusters including those randomized to the intervention.

### Community actions on GBV

At baseline, 38.6 percent and 43.8 percent of women in the intervention and control arms, respectively, reported having witnessed an act of GBV or VAC within the 12 months prior to the survey. The percentages at follow-up fell to 31.3 in both arms (time effect OR = 0.60, 95% CI: 0.48–0.75); no effect of the intervention was found. Among those who witnessed an act, slightly more than a third of women at baseline in both intervention and control arms reported taking action to stop the violence or help the survivor. At follow-up, this increased to about half of women in both the intervention and control arms (time effect OR = 1.49, 95% CI: 1.06–2.10) with no observed intervention effect. The intervention was found, however, to influence women’s reported initiation of conversations about GBV or VAC with another person in the past 12 months, with intervention prevalences from baseline to follow-up increasing from 18.8 percent to 21.6 percent in the intervention arm and decreasing from 21.3 percent to 15.8 percent in the control arm (intervention effect OR = 1.56, 95% CI: 1.12–2.18; time effect OR = 0.73, 95% CI: 0.56–0.95). The intervention also contributed to a higher percentage of respondents in the intervention arm at follow-up reporting that community leaders speak out against GBV and VAC (38.5% intervention, 22.8% control; intervention effect OR = 2.02, 95% CI: 1.51–2.71), while no change over time was found in the control group. Similar levels and changes were seen in the prevalence of positive assessments of community responses to GBV with an intervention effect that approached statistical significance (intervention effect OR = 1.35, 95% CI: 0.98–1.87, p = 0.064). A quarter of respondents (25.8%) in the intervention arm at follow-up reported being aware of a community action committee on GBV and about a third (31.0%) reported being aware of volunteers who helped GBV survivors access services, both community services that the WRP/HJFMRI GBV program aimed to establish. They were 2.45 times more likely to report awareness of these community services compared to respondents in the control arm (OR = 2.45, 95% CI = 1.50–4.00; and OR = 2.49, 95% CI = 1.57–3.96, respectively). (See Table 9).

**Table 9:** Community actions to address GBV.

	Baseline		Follow-up				Odds-ratio of follow-up to baseline prevalence among control clusters (time effect)			Odds-ratio of intervention to control clusters prevalence at follow-up (intervention effect)		
	Interv’n	Control	Interv’n		Control		Est. OR	95% CI	p value^a^	Est. OR	95% CI	p value^a^
	Percent	Percent	Percent		Percent							
	Freq/N	Freq/N	Freq/N		Freq/N							
**Prevalence of reported actions in the past 12 months**												
Witnessed an act of GBV or VAC	38.6	43.8	31.3	31.3			0.60	0.48–0.75	<0.001	1.14	0.84–1.55	0.386
	251/650	281/641	192/613	193/616								
Took action to stop GBV or help a survivor (among those who witnessed an act)	39.8	36.6	50.5	47.2			1.49	1.06–2.10	0.022	1.09	0.71–1.69	0.690
	100/251	102/279	97/192	91/193								
Started a conversation about GBV or VAC	18.8	21.3	21.6	15.8			0.73	0.56–0.95	0.020	1.56	1.12–2.18	0.008
	123/656	137/643	135/624	98/622								
**Prevalence of reported assessments and awareness of the community’s response to GBV in the past 12 months**												
Community leaders have ever spoken out or acted to address GBV or VAC	30.2	30.8	38.5		22.8	1.00		0.81–1.24	0.978	2.02	1.51–2.71	<0.001
	198/656	198/643	239/621		142/624							
Community has done a good (or very good) job of responding to IPV and VAC	34.8	28.9	38.3		27.4	0.89		0.70–1.13	0.334	1.35	0.98–1.87	0.064
	227/653	185/641	237/619		171/623							
										**Est. OR**	**95% CI**	**p value**^b^
Aware of a Community Action Group on GBV	Intervention		25.8%			X				2.45	1.50–4.00	<0.001
			161/625									
	Control		12.5%			X				Ref	-	-
			78/624									
Aware of community volunteers who help GBV survivors get to services	Intervention		31.0%			X				2.49	1.57–3.96	<0.001
			193/624									
	Control		15.4%			X				Ref	-	-
			96/624									

^a^ p values are based on a GLMM with cluster-specific baseline prevalence equal to the true baseline prevalence plus a random effect for all clusters including those randomized to the intervention.

^*b*^ p values are based on a GLMM with cluster treated as a random effect.

### Utilization of GBV services at health facilities

Among baseline survey respondents who reported some form of IPV (or physical or sexual GBV from a non-partner) in the past 12 months, few (3.9% and 3.7% in the intervention and control arms, respectively) reported seeking services from a health facility regarding the incident. Prevalence of reports of help-seeking from a health facility changed little at follow-up; neither a time nor intervention effect was found. Data from health facility records, however, showed a higher volume of GBV client visits at intervention facilities (N = 1,427) compared to control facilities (N = 489) over the 16-month study period of January 2014 through April 2015. The monthly number of GBV client visits at intervention facilities fluctuated from 60 to 120 with no apparent trend, while monthly counts at control facilities ranged from 16 to 41 client visits and showed less fluctuation. The total number of GBV client visits ranged from 141 to 445 among the six intervention facilities and from 15 to 136 among the six control facilities. On average, intervention facilities recorded three times as many GBV client visits as control facilities (237.8 compared to 81.5, respectively, p = 0.010). GBV client ages ranged from 0 to 90 years with mean ages of 28.5 and 26.8 at intervention and control facilities, respectively. Seventeen percent of clients at intervention facilities were under age 18 compared with 20.2 percent at control facilities. The majority of clients were female (87.1% and 94.3% at intervention and control facilities, respectively). Among clients aged 15 and over, most were married (70.9% and 77.0% at intervention and control facilities, respectively). None of these sex and age differences between the intervention and control arms were statistically significant. Multiple forms of violence typically were assessed and identified at a given client visit among providers at both intervention and control facilities. Emotional violence was the most commonly identified form of violence at intervention facilities (79.3% of client visits compared to 36.7% at control facilities), while physical violence was the most commonly identified form at control facilities (77.0% of client visits compared to 65.6% at intervention facilities). Percentages of clients visits where sexual violence was identified were similar at intervention and control facilities (17.9% and 18.4%, respectively), as were percentages of client visits where neglect was identified (7.1% and 10.1%, respectively). Emotional violence was the only form where the difference between intervention and control facilities was statistically significant (p = 0.017). (See Table 10).

**Table 10:** Characteristics of GBV clients at study health facilities.

GBV register data (January 2014–April 2015)		Intervention			Control			p value^a^
Number of GBV client visits	**N (clusters)**	6			6			0.010
	**Mean (SD)**	237.8 (110.58)			81.5 (46.09)			
	**Range**	141–445			15–136			
Age of client	**N (visits)**	1419			481			0.464
	**Mean (SD)**	28.5 (12.40)			26.8 (10.92)			
	**Range**	0–90			3–70			
		**N**	**Freq**	**%**	**N**	**Freq**	**%**	**p value**^a^
Clients under age 18		1419	241	17.0	481	97	20.2	0.931
Clients who were female		1426	1243	87.1	488	461	94.3	0.337
Clients age 15+ who were currently married		1287	913	70.9	426	328	77.0	0.503
**Client visits where the following forms of violence (IPV and GBV) were identified**								
Sexual violence		1416	254	17.9	489	90	18.4	0.739
Physical violence		1415	928	65.6	488	376	77.0	0.451
Emotional violence		1422	1127	79.3	488	179	36.7	0.017
Neglect		1402	99	7.1	486	49	10.1	0.409

^a^ p values are based on a simple ANOVA of cluster counts (for number of client visits), cluster means (for age), and cluster proportions (for binomial variables).

### Availability and quality of GBV services at health facilities

The percentages of client visits where various GBV services (those designated by national guidelines) were provided are presented in Table 11.

**Table 11:** Services delivered to GBV clients.

GBV register data (January 2014–April 2015)	Intervention				Control			p value^a^
	N	Freq	%		N	Freq	%	
**Percent of client visits where the following services were provided**								
GBV screening and counseling	1413	1251		88.5	482	442	91.7	0.785
Assessment of physical state	1422	1151		80.9	489	474	96.9	0.326
Assessment of mental state	1420	1044		73.5	489	378	77.3	0.572
Psychosocial counseling	1422	1210		85.1	486	333	68.5	0.195
Counseling on HIV and HIV testing	1416	1038		73.3	488	102	20.9	<0.001
HIV test	1414	782		55.3	489	96	19.6	0.002
STI test	1415	308		21.8	488	56	11.5	0.128
STI prophylaxis/treatment	1408	128		9.1	489	34	7.0	0.387
Pregnancy test (among female sexual violence clients ages 12–59)	204	96		47.1	68	46	67.6	0.141
Family planning counseling (among female clients ages 12–59)	1137	389		34.2	423	62	14.7	0.050
Family planning method (among female clients ages 12–59)	1139	84		7.4	425	36	8.5	0.967
Police form 3 was completed	1413	524		37.1	274	486	56.4	0.290
Tetanus toxoid immunization given (among sexual or physical violence clients)	1052	322		30.6	428	149	34.8	0.738
Forensic exam was performed (among sexual or physical violence clients)	1050	115		11.0	419	190	45.3	0.025
Forensic evidence was collected (among sexual or physical violence clients)	1048	123		11.7	422	101	23.9	0.177
Percent of sexual violence clients who arrived at facility within 72 hours	215	114		53.0	81	51	63.0	0.163
HIV PEP (among sexual violence clients who arrived at facility within 72 hours)	113	75		66.4	51	28	54.9	0.169
PEP adherence counseling (among sexual violence clients who arrived at facility within 72 hours)	112	76		67.9	51	28	54.9	0.141
Emergency contraception (among female sexual violence clients ages 12–59 who arrived at the facility within 72 hours)	78	35		44.9	33	9	27.3	0.066
**Referrals outside the facility:** Percent of client visits where the following referrals were made								
Legal services	1415	667		47.1	489	135	27.6	0.079
Police	1418	566		39.9	489	194	39.7	0.762
Psychosocial care	1419	373		26.3	487	147	30.2	0.865
Safe house or shelter	1412	173		12.3	488	11	2.3	0.216
Clinical care at a higher-level health facility	1412	44		3.1	489	5	1.0	0.532

^a^ p values are based on a simple ANOVA of cluster counts.

GBV counseling in conjunction with screening, assessment of physical and mental states, and psychosocial counselling were provided at most (>70%) GBV client visits at both intervention and control facilities. Other services such as lab tests, family planning services, forensic services, and HIV services were less likely (<70%) to be provided at all facilities. Few differences were observed in the delivery of services between intervention and control facilities among the clients they saw. Of note, however, a higher percentage of GBV client visits at intervention compared to control facilities included counseling on HIV and on HIV testing (73.3% vs. 20.9%, respectively, p<0.001) and an HIV test (55.3% vs. 19.6%, respectively, p = 0.002). Also, a higher percentage of client visits at intervention compared to control facilities included family planning counselling (34.2% vs. 14.7%, respectively, p = 0.050). Unexpectedly, forensic tests were performed during a smaller percentage of visits where clients had been assessed with sexual or physical GBV at intervention compared to control facilities (11.0% vs. 45.3%, p = 0.025). While sexual violence was assessed at four times as many client visits at intervention compared to control facilities (215 vs. 51, respectively), sexual violence clients at intervention facilities were no more likely to arrive within 72 hours than those seen at control facilities (53.0% vs. 63.0%, respectively). Among those who did arrive within 72 hours, those at intervention facilities compared to intervention facilities were somewhat more likely to receive HIV PEP (66.4% vs. 54.9%), PEP adherence counselling (67.9% vs. 54.9%), and emergency contraception (44.9% vs. 27.3%). None of these differences, however, were statistically significant. Referrals to legal services were made in nearly half (47.1%) of client visits at the intervention facilities, while legal services referrals were given at only about a quarter (27.6%) of client visits at control facilities. Police referrals were made at about 40 percent of client visits at both intervention and control facilities. Referrals to psychosocial care were provided less than a third of the time (26.3% and 30.2% at intervention and control facilities, respectively). Far fewer referrals were made to safe houses or shelters (12.3% at intervention facilities and 2.3% at control facilities). Referrals to higher-level health facilities for clinical care were rarely provided (3.1% and 1.0% of client visits at intervention and control facilities, respectively). None of the differences in referrals between the intervention and control arms were statistically significant.

### Utilization of HIV-related services

About three-quarters of survey respondents at baseline in both the intervention and control arms reported they had ever been tested for HIV and these proportions increased at follow-up in both arms to over 85 percent. At baseline, over a third of respondents in both arms had been tested within the past 12 months and the odds of recent HIV testing increased by 50 percent from baseline to follow-up (time effect OR = 1.50, CI: 1.21–1.87). No intervention effect was found for either lifetime or recent HIV testing. Slightly less than a quarter of survey respondents at baseline (22.3% and 20.4% in intervention and control arms, respectively) reported knowing that HIV PEP was available in their communities. Women at follow-up were more than two and half times more likely to report knowledge of availability of HIV PEP in their communities compared to baseline (time effect OR = 2.85, CI: 2.24–3.63); no intervention effect was found. (See Table 12).

**Table 12:** HIV testing and knowledge of PEP availability.

	Baseline		Follow-up		Odds-ratio of follow-up to baseline prevalence among control clusters (time effect)			Odds-ratio of intervention to control clusters prevalence at follow-up (intervention effect)		
	Interv’n	Control	Interv’n	Control	Est. OR	95% CI	p value^a^	Est. OR	95% CI	p value^a^
	Percent	Percent	Percent	Percent						
	Freq/N	Freq/N	Freq/N	Freq/N						
**Prevalence of reported actions in the past 12 months**										
Ever tested for HIV	75.2	73.4	85.8	88.9	1.16	1.11–1.21	<0.001	0.96	0.91–1.02	0.177
	493/656	472/642	537/624	555/626						
Tested for HIV in the past 12 months	39.9	33.6	47.1	44.7	1.50	1.21–1.87	<0.001	0.94	0.70–1.26	0.700
	262/656	216/642	295/626	279/624						
Knows that HIV PEP is available in her community	22.3	20.4	45.7	42.2	2.85	2.24–3.63	<0.001	1.09	0.79–1.51	0.592
	146/655	131/643	282/626	263/623						

^a^ p values are based on a GLMM with cluster-specific baseline prevalence equal to the true baseline prevalence plus a random effect for all clusters including those randomized to the intervention.

## Discussion

Findings from the Tathmini GBV study showed that the comprehensive package of GBV services delivered by WRP/HJFMRI through its HIV/AIDS program platform positively influenced women’s knowledge, attitudes, and beliefs about GBV including more widespread awareness of laws and policies on violence, less acceptance of partner violence for refusal of sex, and a shift toward more gender equitable norms. Although VAC was not a primary focus of the study, the intervention was found to also contribute to better-informed beliefs about sexual violence against children. These results suggest that substantial community change can occur within a relatively short period of approximately two years and with modest program coverage, i.e., only about a quarter of respondents in the intervention arm reported any direct program exposure. They are consistent with findings from the SASA! Study in Kampala, Uganda, which also found that community mobilization to prevent violence and reduce HIV-risk behaviors led to lower social acceptance of IPV among women and greater acceptance that a woman can refuse sex [23]. Diffusion of information and influence, the aim of several WRP/HJFMRI GBV program community components that were based on the SASA! intervention, likely contributed to these population-level impacts as evidenced by the greater likelihood of women in the intervention arm starting conversations about GBV and VAC with others in their community, more favorably assessing community responses to these forms of violence, and reporting awareness of community leaders’ public discourse and action.

The Tathmini GBV study also demonstrated the potential of the WRP/HJFMRI GBV program to reduce the prevalence of IPV in its various forms. In this regard, it makes an important contribution to the growing body of evidence of proven and promising interventions to prevent IPV and other forms of GBV in sub-Saharan Africa [23, 26, 47–51]. While the odds of reporting experience of IPV decreased by 44 percent from baseline to follow-up in the intervention arm, it also decreased in the control arm and the study was not sufficiently powered to detect the smaller contribution of the program. Several factors could have influenced the changes in outcomes over time observed in the control as well as the intervention arm including differences in the baseline and follow-up samples. However, although baseline and follow-up differed on several characteristics, among them only marital status was found to be associated with experience of IPV, i.e., at baseline, prevalence of IPV was significantly greater among women currently married or living with a partner compared to women in other types of relationships. The higher percentage of currently married women at follow-up relative to baseline, thus, could have been expected to contribute to higher IPV prevalence at follow-up, contrary to what was observed. Unknown, however, are sample differences in unmeasured characteristics associated with reported experience of IPV. Program contamination of the control clusters was unlikely given that changes over time in many of the intermediate outcomes were not observed in the control arm. Also, population movement within the study area was limited due to geography of the region and follow-up survey findings showed low levels of awareness of or participation in GBV program activities with the exception that about half of women in both study arms reported awareness of GBV media campaigns other than the WRP/HJFMRI AMKA SASA! campaign. Of note, the TDHS, which was conducted twice during approximately the same time period as the Tathmini GBV study, reported similar IPV prevalence levels and decline over time among women ages 15–49 in Mbeya Region–from 59.9 percent in 2010 [32] to 41.4 percent in 2015–16 [52]. The TDHS findings provide some validation of the study findings and also suggest the presence of other contextual factors that may have influenced the study outcome. Further research is warranted to more fully understand factors that contributed to the significant decline in IPV in the study population and throughout Mbeya Region.

With regard to care for GBV survivors, the intervention was found to positively impact utilization of GBV services at the health facilities with nearly three times more GBV client visits recorded at intervention compared to control facilities. While limitations of the study design preclude determination of the factors that led to this increased utilization, more proactive screening for GBV, a component of the national GBV service delivery guidelines, may have contributed to this outcome in light of the absence of an observed program effect on GBV survivors’ reports in the household survey of seeking help at a health facility. The finding that service providers at intervention compared to control facilities were more likely to identify emotional violence among GBV clients also lends support to this hypothesis. The lower prevalence of emotional violence at control facilities, however, may also be explained by the fact that service providers at these facilities had not been trained to identify emotional violence, a more hidden form of violence. It is also possible that some GBV clients seen at the intervention facilities resided outside the catchment area of those facilities, including from the control cluster communities. A recent study in central Tanzania found that stigmatization of violence led some survivors to seek care at facilities farther away than those where they usually sought health care [17]. Knowledge that improved care was available at the intervention facilities could have provided an added incentive to seek care there. Further research is needed to better understand the motivation and patterns of help-seeking behavior among those who experience various types of GBV.

The intervention was not associated with improved quality of GBV clinical services as measured by the array of tests, services, and referrals provided. For some services, such as emergency contraception, sample sizes were small resulting in insufficient statistical power to detect a difference. But, for others, such as forensic services, findings showed that GBV clients at control facilities were just as, or more likely to be given the service than those at the intervention facilities. This finding could perhaps be explained by a higher proportion of more severe, life-threatening conditions among GBV clients presenting at control facilities (in part, because less severe cases were not identified). Incomplete recording of services in the GBV register, perhaps resulting from the greater client load at intervention facilities, cannot be ruled out. Recent reviews of the health system response to GBV globally and, in particular, of efforts to integrate GBV services into general health care settings, have highlighted the enormous scope of needed efforts and challenges [53, 54]. Failure to demonstrate improved service delivery performance may reflect the realities of early stages of program scale-out; that is, GBV services integration was rolled out just prior to the start of the study and the study facilities were among the first in Tanzania’s national program rollout. Given Tanzania’s pioneering efforts among sub-Saharan African countries to nationally scale-out GBV services integration within the public health system, others could likely benefit from further study of Tanzania’s experience and learning.

The holistic approach of combining GBV prevention and response services into a unified, comprehensive program was a unique characteristic of the WRP/HJFMRI GBV program with the expectation that stronger linkages between community and facility services would lead to better prevention and response outcomes. However, evidence that the program strengthened referrals from health facilities to community and other services was lacking, perhaps in part due to unavailability, limited access, or poor quality of critical services such as police protection, legal services, shelter, psychosocial support, and social protection. Given the importance of services outside the health sector to the well-being of GBV survivors and to curbing GBV, they, too, must be strengthened together with strengthening service linkages within the multi-sectoral network. Within the HIV/AIDS program platform, integration of VAC and IPV interventions within programming for orphans and vulnerable children provides an opportunity to build out the services network and jointly address these two associated forms of violence and their intersection with HIV/AIDS [55].

Of further importance to HIV/AIDS programming, the WRP/HJFMRI GBV program was found to be associated with greater provision of HIV counseling and testing services for GBV survivors, as indicated by the health facility data. Given the association between GBV and HIV, it is likely that these individuals were also among those at elevated risk of HIV. The intervention, however, was not found by the household surveys to affect uptake of HIV testing among women ages 15–49 residing within the study communities. Significant increases over time were seen in both arms with nearly half of survey respondents reporting receipt of an HIV test in the past 12 months and about three-quarters reporting having ever been tested. This result is not surprising given the priority of HIV testing by the WRP/HJFMRI HIV program in all study locations. It does suggest, however, that sensitizing communities about the links between GBV and HIV, a key message of the community interventions, may not have added motivation for HIV testing. Also, no evidence was found that the program made a difference in the timeliness of sexual violence survivors seeking health services, i.e., within 72 hours of the incident, enabling their eligibility for PEP. Increases in knowledge about the availability of PEP increased over time in both intervention and control clusters and this may have diluted an intervention effect. But, more comprehensive messaging about PEP may also be needed along with removal of other barriers, e.g., financial constraints, lack of transport, and stigma, etc., that prevent sexual violence survivors from reaching a health facility in a timely manner.

## Conclusion

The Tathmini GBV study demonstrated the feasibility of integrating a comprehensive GBV prevention and response program within an HIV/AIDS program platform and the effectiveness of the program in fostering community-wide changes in attitudes and norms regarding GBV and VAC, increasing community actions to address violence, and increasing utilization of GBV services at health facilities. Findings confirmed those from other recent studies in sub-Sharan Africa and contributed to the growing evidence base that GBV programs can make important contributions to achievement of local, national, and global GBV and HIV/AIDS goals. The WRP/HJFMRI GBV program, which included a mix of evidence-informed program components that were adapted to the local context and shaped by locally-defined needs and program capacity, pioneered implementation of newly developed national service GBV and VAC delivery guidelines, and embedded GBV programming within existing HIV/AIDS service delivery systems offers a promising model for scaling out GBV programming. Contributions of the program to sustained population-level attitudinal changes, reductions in violence, and improved health and well-being of GBV survivors remain to be tested. Additional studies are merited to determine an optimal mix and dosage of program components, to better understand potential synergistic effects, and to test robustness of the program model in different low- and middle-income settings.

## Supporting information

S1 Checklist CONSORT checklist. (PDF)

S1 File Study protocol. (PDF)

S2 File Kiswahili questionnaire. (PDF)
