# Supplementary material for: Cluster randomized trial of comprehensive gender-based violence programming delivered through the HIV/AIDS program platform in Mbeya Region, Tanzania: Tathmini GBV study
Source: PLoS One. 2018 Dec 6;13(12):e0206074. doi: 10.1371/journal.pone.0206074 (PMC6283609; doi:10.1371/journal.pone.0206074)
Supplement: S1 File — (PDF) [file pone.0206074.s002.pdf]

October 26, 2015

1. **Title Page**

Tathmini GBV

Evaluation of the Outcomes of Comprehensive Gender-based Violence Programming in Tanzania

September 12, 2012 (Revised October 26, 2015)

Principal Investigator:
Susan Settergren, Palladium, susan.settergren@thepalladiumgroup.com
1331 Pennsylvania Avenue NW, Suite 600, Washington, DC 20004 USA, +1 202 775 9680

Co-Investigators:

Megan Dunbar, Pangaea Global AIDS Foundation, mdunbar@pgaf.org,
472 Ninth Street, Oakland, CA, 94607 USA, +1 510 379 4007

Jessie Mbwambo, Muhimbili University of Health and Allied Sciences,
Department of Psychiatry and Mental Health, jmbwambo@gmail.com
PO Box 65001, Dar es Salaam, Tanzania, +255 784 339 747

Study Coordinator: Scott Geibel, Population Council

Study location: Mbeya Region, Tanzania

Project dates: October 1, 2012 –May 31, 2016

Signatures for the Principal Investigator:

Name Susan Settergren **Date** October 26, 2015

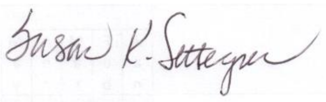

2. Summary of Proposed Research

This research protocol describes an outcome evaluation that the President's Emergency Plan for AIDS Relief (PEPFAR) has funded through USAID to evaluate the comprehensive facility- and community-based gender-based violence (GBV) program that is being rolled out in Tanzania with support from the PEPFAR GBV Initiative. The GBV program and this study focus on intimate partner violence in the forms of sexual, physical, and emotional violence; and on sexual violence among both adults and children, irrespective of the relationship between perpetrator and survivor. The program includes interventions focused both on preventing GBV and on strengthening services for GBV survivors.

The study will employ a pair-matched cluster randomized trial design to compare the effectiveness of this comprehensive programming over time with standard practice in control facilities and communities. The study has two primary aims, or objectives, which are to assess whether the comprehensive GBV program intervention resulted in an expected 1) <u>increase in care for GBV survivors</u>, reflected in delivery and utilization of GBV services at health facilities, and 2) <u>decrease in experience of GBV among females ages 15-49</u>. Secondary study aims include assessment of program effects related to knowledge, attitudes, and norms regarding GBV and gender equality; community-led actions to address GBV; knowledge of and barriers to utilization of GBV services; GBV service components and quality; GBV service and program capacity and coverage; and HIV-related outcomes. Data collection will include a household survey of females ages 15–49 at baseline and endline (i.e., after approximately 24 months of program implementation); extraction of client data from health facility GBV registers; key informant interviews with community representatives and program participants; health facility assessments; and key informant interviews with program managers. The study will take place in 12 clusters across Mbeya region, where each cluster is defined by a Health Center or District Hospital, and communities surrounding these facilities. The program itself will be implemented by local partners who are supported by PEPFAR through the Walter Reed Program in Tanzania.

All studies on GBV involving human subjects pose potential risks for study participants, including risks which may arise from personal distress caused by discussing GBV experiences and from further abuse to the survivor if others learn that s/he has discussed it with the study team. To minimize these risks, the study team will adhere to WHO guidelines regarding the conduct of research on GBV (WHO, 1999) to ensure the safety of study participants and the study team, and confidentiality of participant identity and data. The study will be conducted in a manner that aims to reduce possible distress caused to participants by the study, including ensuring that interviewers are certified in ethical research practice, well trained, and equipped with information and skills to refer participants to GBV services as appropriate.

The proposed study design is methodologically sound and builds upon past research conducted by the study team and others to address key gaps in the evidence base for GBV programming. The study is well timed to coincide with the initial, national roll-out of GBV services by the Ministry of Health and Social Welfare (MOHSW) later this year. Results from the study will inform the scale-up of services and provide critical evidence on public health outcomes. The study team will develop and implement a dissemination plan that will help to ensure that study findings are properly interpreted and used widely to advance GBV policy and programs, both within Tanzania and globally.

3. Description of Protocol

3.1. Background

3.1.1 Rationale for the study

Global interest and investment in programs to prevent GBV and mitigate its harmful impacts on individuals, families, and communities have accelerated in recent years, in part due to growing understanding of the association between GBV and HIV risk. The United Nations Program on HIV/AIDS (UNAIDS) has identified the elimination of GBV as a core pillar of HIV prevention (UNAIDS, 2009). In 2010, PEPFAR launched an initiative that focuses on countries hardest hit by both GBV and HIV; Tanzania is one of three focus countries in this initiative.

> **Gender-based violence**: An umbrella term for any act, omission, or conduct that is perpetuated against a person's will and that is based on socially ascribed differences (gender) between males and females. In this context, GBV includes but is not limited to sexual violence, physical violence and harmful traditional practices, and economic and social violence. The term refers to violence that targets individuals or groups on the basis of their being female or male."
>
> —Ministry of Health and Social Welfare, 2011

Although HIV program efforts to address GBV have grown significantly in recent years, research on the effectiveness of these interventions is scant. The research that has been done has focused either on interventions at community and individual levels to promote gender equality and change GBV-related attitudes and behavior, or on strengthening response services for GBV survivors. A unique contribution of the proposed study is evaluation of a comprehensive program model that includes a full array of community- and facility-based programs and services that are hypothesized to operate synergistically to address both GBV prevention and care for survivors.

The 2010 Tanzania Demographic and Health Survey (TDHS, 2010) found that 44 percent of ever-married women have experienced physical or sexual violence from their current/most recent husband or partner, and 37 percent of ever-married women experienced intimate partner violence (IPV) in the 12 months prior to the survey; 20 percent of women ages 15–49 have ever experienced sexual violence (SV), usually perpetrated by their partners or former partners. Fifty-four percent of women and 38 percent of men ages 15–49 believe that a husband is justified in beating his wife for certain reasons. The 2009 Violence Against Children (VAC) Survey (United Nations Children's Fund et al., 2011) found that nearly three in 10 females and one in seven males have experienced violence prior to age 18. Only one in five female and one in 10 male survivors sought any services. Of those who had their first sexual experience prior to age 18, 29 percent of females and 18 percent of males reported that it was not consensual. The prevalence of sex with two or more partners in the last 12 months was significantly higher among those who experienced childhood sexual violence. Studies elsewhere have shown that sexually abused children are more at risk of acquiring HIV as adults (Slonim-Nevo and Mukuka, 2007).

A qualitative study conducted in Dar es Salaam found several factors associated with child sexual offences, including community passivity, weakness in the legal system, inadequacy of the legal framework, and vulnerability of key players (Kisanga, 2010). Specifically, magistrates and legal investigators were found often to live within the communities they serve, thus

compromising their ability to be objective in handling cases of child sexual abuse. In another study that explored community perceptions about child sexual abuse in Tanzania, focus group discussions with adult community members revealed a lack of trust in the healthcare and legal systems, issues of disclosure related to fear of stigma, myths justifying child sexual abuse, decreased respect for children's rights, and awareness coupled with distress (Kisanga, 2011). The perception gleaned from these studies is that the weakened legal structure, combined with communities' reluctance to get involved in child sexual abuse cases, makes violence against children a particularly complicated issue requiring improved screening and interventions at community and health facility levels.

The following sections outline research conducted by the research team and others on what is known regarding the relationship between GBV and HIV and the effectiveness of various intervention strategies to be implemented as a comprehensive model by PEPFAR-supported partners in Tanzania and evaluated through the study presented in this protocol.

3.1.2 Relationship between GBV and HIV

The gendered nature of the HIV epidemic has received increasing attention, with women now constituting 60 percent of adults living with HIV in sub-Saharan Africa (UNAIDS, 2010). Recent analyses of cohort data regarding incident HIV infection have found IPV to be an independent risk factor for HIV (Jewkes, 2010). Baseline results from the cluster randomized control trial of SASA!, a community mobilization intervention developed by Raising Voices in Uganda to change community and individual attitudes and behaviors that support both the perpetration of violence against women and HIV risk behaviors, showed that reports of sexual behavior differed significantly according to whether or not the respondent reported experience of GBV, suggesting associations between sexual risk behaviors and IPV. Women who had experienced and men who had perpetrated IPV in the past year were more likely than those who had not to have had multiple partners and/or extramarital partners and less likely to use condoms (Abramsky et al., n.d.). A cross-sectional study in South Africa found that young men (under age 25) who had been physically violent towards a female partner had twice the odds of having HIV as young men who had not been violent (Jewkes et al., 2011).

While the association between GBV and HIV acquisition is widely accepted, the pathways are myriad and complex. It can be direct, as with physical trauma from SV inflicted by someone who has HIV. For example, coerced sex and other forms of SV may contribute directly to a woman's HIV risk, being a potential route of exposure to the virus as well as increasing the chances of HIV transmission if lacerations or other genital trauma occur (Maman et al., 2000). Women in violent relationships are also less likely to be able to negotiate the frequency or circumstances of sex, thus exacerbating their HIV risk (Campbell et al., 2008; Jewkes and Morrell, 2010).

Evidence also points to several indirect mechanisms of association between GBV and HIV through a chain of effects that include psychological, economic, and gendered conditions and behaviors that lead to a victim's engagement in high-risk sex. For example, women in violent relationships may be more likely to have multiple partners (He et al., 1998; Dunkle et al., 2004), have unprotected sex (Wingood and DiClemente, 1997), and have had an early sexual debut. Sometimes just the threat or fear of violence is sufficient to keep a woman from protecting herself from HIV.

Compounding the situation are barriers that GBV may create to a survivor's access to HIV services. A review for the U.S. Institute of Medicine, based on studies between 1998 and 2007, found that "violence or fear of violence from an intimate partner is an impediment (to) or a consequence of HIV testing" (Campbell et al., 2008). Fears of violent repercussions may prevent women from uptake of HIV testing or disclosing their HIV status (Maman et al., 2001; Medley et al., 2004; WHO, 2000). Individuals typically keep violence hidden to avoid stigma, discrimination, and harmful repercussions. Parents of victimized girls often keep it secret to prevent shame to the family and the perceived ineligibility of their daughters for marriage. In other situations, girls may be forced to marry the perpetrators of rape or violence to maintain social acceptability. Thus, survivors face enormous barriers to accessing most types of services. Interventions that address the stigma and strengthen linkages between services and the community are urgently needed.

Living with HIV also can put a woman at greater risk of violence. Vulnerability may arise from accusations by her partner or family that she has had sex outside the partnership, or as a socially acceptable form of discrimination against persons with HIV. Against a backdrop of gender inequality, an HIV diagnosis and/or its disclosure may put a woman at increased risk of IPV (Maman et al., 2000). GBV also can prevent survivors from realizing the benefits of health services; for example, a controlling partner may keep a woman from adhering to medication.

Inequalities perpetuated by gender-based norms and roles, cultural practices, and laws collectively contribute to power imbalances between women and men and boys and girls. These gendered power imbalances play out at societal, institutional, relationship, and individual levels, and are powerful drivers of GBV and HIV. Female gender roles that assume women's subservience to men, and power imbalances in sexual relations, often sanction the acceptability of violence against women and can increase vulnerability to HIV (Gupta, 2000). Research has shown that women who believe there are justifiable circumstances for a man's violence toward his partner are more likely to experience IPV (Abramsky et al., 2011). Evidence also indicates that women in less equitable relationships are at increased risk of HIV. For example, Jewkes, Dunkle, Nduna et al. (2010) found that inequity in relationship power was an independent risk factor for incident HIV infection among women, even after controlling for partnership duration and other indicators of risk behavior.

Some sub-Saharan African countries are characterized by male-dominant cultures, such that violence perpetuated by men against women and children is a socially acceptable means of exerting dominance and control. Given that HIV mainly is acquired through heterosexual relations in sub-Saharan Africa, gender inequality and norms of male dominance are a major force behind the confluence of HIV infection and GBV (Kaye, 2004). For example, rape is sometimes used by men as a means of "punishment" or to exert control over women, including young girls. Such behavior has been described in Namibia and South Africa (Jewkes et al., 2005), as well as in Zimbabwe (Meursing et al., 1995). Further, cultural taboos and social stigma prominent in sub-Saharan Africa severely limit open discussion about sexuality (Ecker, 1994). The combination of a male-dominated cultural landscape and cultural taboos against talking about sex condone and perpetuate GBV.

3.1.3 Evidence of effective community-based interventions

While the body of evidence on effective interventions to reduce GBV is relatively small, results from the rigorous studies that do exist are compelling. For example, the IMAGE Study conducted in South Africa demonstrated that a microfinance program for poor women, combined with gender and HIV training, resulted in a 55 percent reduction in past-year IPV among intervention participants over the two-year study period (Pronyk et al., 2006). The study also found increases in uptake of HIV testing, HIV-related communication in the household, and a reduction in the prevalence of unprotected sex at last intercourse with a non-spousal partner among younger intervention participants (Pronyk et al., 2008). Increases in female empowerment, measured as autonomy in decisionmaking, increased financial confidence, and increased participation in collective action, also were found.

SHAZ! (Shaping the Health of Adolescents in Zimbabwe) combined access to sexual and reproductive health services and life skills education with vocational training and grant support for economic activities for adolescent girls and women in urban Zimbabwe. Using a randomized control trial design, the evaluation compared the SHAZ! intervention package to the life skills intervention alone on economic and social empowerment indicators, sexual behavior, and experience of violence. Both groups saw reductions of more than half in reports of physical and sexual violence over the two-year period, but the magnitude of change was greater among intervention participants (Dunbar, 2009). Stepping Stones, a participatory HIV prevention program that aims to improve sexual health through building more gender-equitable relationships, has been shown to reduce sexual risk behaviors and reported levels of perpetration of IPV against women among male participants (Jewkes et al., 2008). The intervention also was associated with a 33 percent reduction in herpes simplex virus -2 (HSV-2) incidence among women and men.

3.1.4 Response services for GBV survivors

The Tanzania MOHSW recently has prioritized improvements and expansion of health services for GBV survivors. This study is especially timely given the September 2011 MOHSW release of the *National Policy Guidelines for the Health Sector Prevention of and Response to GBV* and the *National Management Guidelines for the Health Sector Response to and Prevention of GBV*—and the anticipated training on and roll-out of GBV health services later this year.

GBV has a wide range of health consequences. Apart from physical and emotional harm, and stigma and social ostracism, women suffer sexual and reproductive health implications such as unwanted pregnancy, sexually transmitted infections (STIs), and the risk of HIV infection (WHO, 2006). Health consequences of GBV are summarized in Table 1.

Table 1: Health consequences of gender-based violence

Fatal outcomes	Non-fatal outcomes		
• Homicide<br>• Femicide<br>• Suicide<br>• AIDS-related mortality<br>• Maternal mortality	Physical	Sexual and reproductive	Psychological and behavioral
	• Fractures<br>• Chronic pain syndromes<br>• Fibromyalgia<br>• Permanent disability<br>• Gastrointestinal disorders	• STIs, including HIV<br>• Urinary tract infections<br>• Unwanted pregnancy<br>• Pregnancy complications<br>• Vaginal bleeding<br>• Traumatic gynaecologic fistula<br>• Unsafe abortion<br>• Chronic pelvic pain	• Depression and anxiety<br>• Eating and sleeping disorders<br>• Drug and alcohol abuse<br>• Poor self-esteem<br>• Post-traumatic stress disorder<br>• Self-harm

Source: Adapted from Bott et al., 2005.

Survivors thus may present with a wide range of immediate or more chronic complaints. Given that healthcare workers are often the first and sometimes only point of contact for GBV survivors, numerous opportunities exist for the health sector to provide care for these survivors. Further, early identification can help with treatment, limit the consequences, and decrease further violence (Laisser, 2011). Proper care often is not provided, however. For example, many healthcare workers know little about how to manage SV clients, and scant attention is paid to IPV. Additionally, healthcare workers are rarely informed about national legislation on GBV or local services to which women could be referred (Mullick et al., 2010). Many healthcare providers fail to diagnose and register GBV. This is due to sociocultural and traditional barriers, but also to lack of time and resources, inadequate physical facilities, lack of awareness, limited knowledge, and poor clinical practices with minimal direct communication. Inadequate training and awareness often result in the failure of healthcare providers to complete a full examination of the survivor or register and monitor the effectiveness and quality of care.

Given the complexity of the service needs and delivery systems, the provision of GBV services cannot be implemented in isolation, but must be considered as part of a systematic approach that extends beyond the facility to the community. For example, many barriers in access to services exist. Study findings from Kenya show that barriers to GBV services include, for example, community misconceptions about the necessity of reporting GBV to the police prior to accessing healthcare; social stigma against GBV survivors; and the threat of further violence, resulting in survivors' nondisclosure and non-pursuit of services. At the facility level, challenges include staff attitudes and general stigma against survivors, as well as insufficient provider capacity (Njuki et al., 2012).

Integrated health services provided within the context of well-coordinated referral networks of social services is a recognized strategy for meeting the unique health needs of women and children (Ferdinand, 2009; Global Health Initiative, n.d.; Women Won't Wait, 2010). While health services and programs may be fragmented, an individual survivor's health and social needs are comprehensive, including the need for multiple types of care (e.g., primary care, psychosocial counseling and support, family planning and sexual and reproductive health

services, antenatal and maternal healthcare, child healthcare, among others) and social services (e.g., education, livelihood programs, legal assistance). Strengthening linkages and integration between and among services can increase access, which is a fundamental priority for individuals already facing barriers due to poverty, low social status, lack of education, stigma, discrimination, and GBV (Keesbury and Askew, 2010).

Emerging evidence regarding integrated and linked programs indicates that access to comprehensive services, whether through one-stop centers, co-location of services, or functional referral systems, among other strategies, can produce better outcomes for GBV survivors (Keesbury and Askew, 2010). Training programs for different cadres of healthcare workers, police, and community leaders have been shown to increase individuals' comfort level in addressing GBV, paving the way for survivor-centered services, community-based violence prevention efforts, increased utilization of HIV testing and counseling services, and better adherence to antiretroviral therapy (ART) (Keesbury et al., 2011). Research studies and program evaluations also point to challenges to integration, related largely to the pressure on already overburdened health systems (Keesbury et al., 2011). Healthcare worker shortages, burnout, poor infrastructure, lack of emergency equipment and supplies, long wait times, and inadequate geographic coverage must be addressed not only within the context of HIV prevention, care, and support, but also in broader attempts to integrate services (Keesbury and Askew, 2010; Keesbury et al., 2011).

GBV screening and referral services are also a key strategy of the MOHSW medical management of GBV. In Tanzania, healthcare staff are not well informed and oriented on how to diagnose and offer appropriate management to GBV survivors (URT, 2011). In this regard, a tool for GBV screening among women was piloted by pre-trained hospital staff in Dar es Salaam for about one month. Among the 102 women screened, 78 percent had encountered emotional, physical, or sexual violence. Among them, 62 percent had experienced IPV, 22 percent were survivors of violence inflicted by a relative, and 9.2 percent by a work mate. Staff acknowledged that the tool was useful in assisting them in detecting and assisting abused women, whom they were unable to assist prior to the training (Laisser et al., 2011).

3.2 Specific Aims

The overall purpose of the study is to assess the effectiveness of the comprehensive GBV prevention and response program that will be delivered at facility and community levels through the PEPFAR-supported HIV program platform in Tanzania. The study will compare the effectiveness of this comprehensive programming over time with standard practice in control facilities and communities. The GBV program and this study focus on IPV in the forms of sexual, physical, and emotional violence; and on sexual violence among both adults and children, irrespective of the relationship between perpetrator and survivor.

> **Intimate partner** is defined as including current legal spouses, current common-law spouses, current boyfriends/girlfriends (opposite or same sex), former legal spouses, former common-law spouses, separated spouses, or former boyfriends/girlfriends (opposite or same sex). Intimate partners may or may not be cohabiting. Intimate partners may or may not have an existing sexual relationship.
>
> —From Basile and Saltzman, 2002

Two key objectives of the PEPFAR-supported GBV intervention program in Tanzania are "to increase availability, quality, and utilization of GBV services" and "to reduce societal acceptance of GBV and increase protective factors" in support of the goal "to reduce and respond to GBV by leveraging the PEPFAR platform" for "strengthening the national GBV response." In line with these goals, the primary objectives of the study are to assess whether the comprehensive GBV program intervention resulted in an expected 1) increase in care for GBV survivors, reflected in delivery and utilization of GBV services at health facilities, and 2) decrease in experience of GBV among females ages 15-49.

The study has two primary aims to be measured through three primary outcomes. The research questions representing these aims and the outcomes that will be used to answer them are as follows:

> Did the comprehensive GBV program lead to increased care for GBV survivors? The primary outcomes are **Delivery of comprehensive sexual violence (SV) services at Health Centers and Hospitals** and **Utilization of GBV services at Health Centers and Hospitals.** These outcomes will be measured for adults and children, male and female.
>
> Did the GBV program lead to a decline in GBV? The primary outcome is **Recent experience of IPV (sexual, physical, or emotional)**. This outcome will be measured for females ages 15–49 in heterosexual intimate partnerships.

GBV services, comprehensive SV services, and forms of IPV are defined according to the MOHSW management guidelines (URT, 2011).

The study will address an additional set of associated secondary aims. Secondary research questions include the following:

For care of GBV survivors, did the intervention lead to any of the following:

- Improved quality of GBV services delivered at health facilities?
- Increased GBV screening and referrals at health facilities?
- More comprehensive GBV care–that is, did survivors receive more of the services they needed?
- Changes in referrals to health facilities? In referrals from health facilities to other services?
- Reduced barriers to access to services?
- Reduced stigma associated with GBV?
- Increased knowledge within communities about health services for GBV? About other GBV services?
- Increased community actions (and, if so, what actions) to facilitate survivor access to GBV services?

For a decline in GBV, did the intervention lead to any of the following:

- Greater decrease in some forms of IPV compared to others?
- Decrease in other forms of GBV?
- Decrease within communities in the acceptance of GBV?
- Increase in community-led actions to stop GBV perpetration?

- An increase in community-led actions to assist those at risk in preventing experience of GBV?
- Shift in community norms toward greater gender equality?
- Structural changes aimed at preventing GBV?

<u>For HIV-related outcomes, did the intervention lead to either of the following:</u>
- Increased utilization of HIV services?
- Reduction in HIV risk behaviors?

<u>The study also includes a process evaluation that will characterize the program and highlight lessons learned including the successes and challenges in rolling out a comprehensive GBV program. Key questions include:</u>
- What resources were allocated to the various program components?
- Did the program meet its objectives and targets?
- What was the coverage and rate of roll out?
- Were some components easier to implement than others?
- Were there changes in program quality over time?
- What are the critical success factors?
- What were the greatest challenges and how were they addressed?

The extent to which key external factors influenced the effects of the program also will be measured. These factors include the following:
- Urban compared to rural setting;
- Seasonal patterns of GBV arising from agricultural cycles;
- Alcohol and substance abuse by survivor or perpetrator;
- Distance of survivor's household from nearest Health Center or Hospital; and
- Availability of GBV support services (e.g., psychosocial support, legal services, and safe houses) outside of health facilities.

The study includes three data collection phases: baseline assessment (prior to the start of the program), ongoing facility and program data collection through the intervention period, and endline assessment (12-24 months after start of the GBV program). Objectives for these components are as follows:

<u>Baseline assessment (within the study cluster communities):</u>
- To establish the prevalence of experiencce of GBV among women of reproductive age (i.e., ages 15-49)
- To assess the knowledge and attitudes towards GBV of community members
- To determine the patterns of GBV among women of reproductive age
- To establish the availability and pattern of utilization of GBV services by GBV survivors
- To establish the quality of GBV services provided to and received by GBV survivors
- To determine the HIV risk behaviours among women of reproductive age
- To determine utilization of HIV services by women of reproductive age

<u>Ongoing data collection</u>:

- To assess changes over time in GBV services utilization and quality (within the study health facilities): quarterly data collection at baseline and throughout the study period
- To characterize program roll out: data collection at 4-6 months following program start

<u>Endline assessment:</u>

- To describe successes and challenges in program implementation

Among community and prevention program participants in the intervention clusters:

- To assess changes in prevalence of experience of GBV among women of reproductive age
- To assess changes in knowledge and attitude towards GBV among community members
- To assess changes in community response to GBV.
- To assess changes in patterns of GBV among women of reproductive age
- To assess changes availability and pattern of utilization of GBV services by GBV survivors
- To assess changes in quality of GBV services provided to and received by GBV survivors
- To assess changes in HIV risk behaviours among women of reproductive age
- To assess changes in utilization of HIV services by women of reproductive age.

3.3. Study Location

The study will take place in Mbeya Region, one of three regions[1] on which PEPFAR GBV programming support in Tanzania is focused. Mbeya was selected based on several factors. These include its predominantly rural population, making it representative both of the country overall and other parts of sub-Saharan Africa (for example, compared to Dar es Salaam, which is primarily urban); the ability to simultaneously roll out the GBV health services and the community interventions at scale, offering a clean "start date' critical for evaluation purposes; the possibility to define distinct, geographically distant clusters to allow for control of potential contamination across intervention and control sites; and capacities within the region for enhanced routine data collection for monitoring purposes.

Mbeya is located in southwestern Tanzania and covers approximately 64,000 square kilometers within three ecological zones: lowlands (within the Rift valley); plains (including Usangu wetlands); and highlands (Uporoto highlands, Livingstone mountain ranges, and Mbozi plateau). Key sources for the economy include agriculture, forestry, industry, tourism, and mining. At the time of the 2002 Tanzania National Census, the region had a population size of 2,070,046, which is estimated to have grown to 2.5 million by 2008, with 2 million people living in rural areas. The region comprises seven administrative districts (Mbeya, Chunya, Mbarali, Kyela, Rungwe, Mbozi, and Ileje), with eight local government authorities (i.e., the seven districts plus Mbeya City). The regional capital is Mbeya City.

[1] The others are Dar es Salaam and Iringa. The newly established Njombe Region, formerly part of Iringa Region, also is included.

HIV prevalence in Mbeya is estimated to be 11.0 percent for females and 6.7 percent for males—higher than the national prevalence. Prevalence of GBV also is high. More than 67 percent of women in Mbeya have experienced some form of partner violence since age 15; nearly half have experienced physical violence and 28 percent have experienced sexual violence. Prevalence of all forms of partner violence are higher in Mbeya compared to Tanzania overall. Levels of recent experiences of sexual and physical violence (regardless of perpetrator) also are higher. Compared with the country overall, survivors of GBV in Mbeya are more likely to tell another person about the experience but less likely to seek help. More than half of women in Mbeya agree that a husband is justified in beating his wife for various reasons, which is similar to attitudes among women nationally. More than a third of Mbeya women report that husbands demonstrate controlling behaviors—again, similar to national levels. Statistics on violence among younger ages are not available for Mbeya, but national prevalence estimates indicate that nearly one-third of girls and 13 percent of boys experience sexual violence before age 18. These key indicators are presented in Table 2.

Table 2. Key indicators on GBV and HIV in Mbeya and Tanzania

Form of GBV	Mbeya	All Tanzania
Ever experienced GBV by partner (among ever-married women)[1]		
Physical	46.9%	39.2%
Sexual	28.3%	17.2%
Emotional	51.2%	36.3%
Any	67.0%	50.2%
Experienced physical violence often in the past 12 months[1]	14.6%	9.4 %
Experienced sexual violence in the past 12 months[1]	30.8%	20.3%
Ever experienced sexual or physical violence and told someone[1]	16.8%	10.1%
Ever experienced sexual or physical violence and sought help[1]	36.8%	46.6%
Husband ever demonstrated controlling behaviors (among ever-married women)[1]	37.8%	35.1%
Agree that a husband is justified in hitting or beating his wife for specific reasons [1,2]	56.5%	53.5%
Experienced sexual violence prior to age 18 (among 13–24 year-olds) [3]	-	28% of females<br>13% of males
First sexual experience was nonconsensual (among those who had their first sexual experience prior to age 18) [3]	-	29% of females<br>18% of males
**HIV prevalence** [4]	11.0% female<br>6.7% male	6.2% female<br>3.8% male

[1]Tanzania DHS, 2010, which surveyed women ages 15–49.

[2] Agree with at least one of the following reasons: wife goes out without telling him, neglects the children, argues with him, refuses sexual relations, or burns the food.

[3] National Survey on Violence against Children, 2009, which surveyed girls and boys ages 13–24.

[4]Tanzania HIV/AIDS and Malaria Indicator Survey, 2011-12 (Tanzania Commission for AIDS et al., 2013).

3.4. Program Interventions

The comprehensive GBV program that this study will evaluate includes a set of five interrelated intervention components, some of which will be delivered at the health facility level and others that will be implemented within communities. The five components are the following:

- Facility-based services for GBV survivors;
- Facility-based screening and referral in clinical settings;
- Clinic and community outreach;
- Community-based GBV prevention activities; and
- Referrals to psychosocial support, legal services, and safe houses.

It is important to note that this study and its protocol include the evaluation of program outcomes only and not implementation of the program itself. The intervention program will be carried out by PEPFAR-supported governmental and nongovernmental partners, which are not directly involved in the study except through the collection of routine project monitoring and evaluation (M&E) data that will be included in the study analysis. These partners include the Tanzania MOHSW; the Mbeya Regional Medical Office (RMO); and nongovernmental organizations (NGOs) provided with financial and technical support from PEPFAR through the U.S. Department of Defense (DoD)—specifically, the Walter Reed Army Institute of Research (WRAIR) and the Walter Reed Program (WRP) Tanzania.

The Mbeya RMO will be the primary implementer of GBV facility-based services. The facility-based interventions will be standardized in line with the Tanzanian National GBV guidelines launched in 2011; efforts currently are underway to develop the guidelines training package and accompanying materials, which are directed to doctors, professional nurses, and social welfare officers. The guidelines cover provision of comprehensive care for survivors of GBV, screening for GBV, and the provision of referrals.

The WRP is currently in the process of selecting nongovernmental partners for implementing community-based GBV interventions, including GBV prevention, community outreach, and community-based referrals to services. The intention is to standardize community-based interventions across study clusters and employ an adaptation of the SASA! community mobilization methodology, which focuses on the benefits of non-violence and gender equity to both men and women, promotes discussion of community norms around relationship power and gender inequality, and mobilizes communities to take and promote action and change (Michau, 2008). The program intervention at the community level includes the six components, as follows:

1. **Group workshops on gender and GBV for women and men**. The program will convene group workshops with women and men of the intervention communities to increase their understanding of how existing gender norms negatively affect their lives, their partners and their families. The evidence-based Men As Partners® methodology and curriculum will be used. This intervention will address male and female attitudes in tandem to promote a more synchronized support for gender equity and other healthy behavior changes. These workshops are expected to lead to raised individual awareness on GBV, reduction in norms of acceptability surrounding GBV, and promotion of respectful, non-violent conflict resolution among workshop participants.

2. **Couples counseling**. Couples counseling services will be provided to individuals ages 20 and above who are within established partnerships. The program will be delivered by Faith Based Organization (FBO) leaders and community HIV Voluntary Counseling and Testing counselors, who will be trained in couples counseling using the "CoupleConnect" curriculum, which is an interactive approach that seeks to help couples learn how to increase communication, negotiation skills, build mutual respect and trust, and maintain healthy relationships (where GBV does not occur).

3. **Community sensitization and mobilization using community activists**. Community activists will be identified and trained using the SASA! methodology to host community dialogues and other forums to promote changes in community attitudes, knowledge, and behavior in areas of gender, human rights, gender roles and expectations, and gender based violence. This intervention seeks to increase knowledge and understanding among communities at large of harmful socio-cultural norms and practices that lead to GBV, and to build the capacity of local leaders and community members to act against GBV. Approaches include public campaigns and outreach services that will be provided during special national and international events (Nane Nane, Saba Saba, World AIDS Day, Women day, 16 days of activisms, etc.).

4. **Awareness raising and capacity building among local leaders**. Sensitization trainings and skills building workshops will be conducted with local leaders, such as, Ward Executive Officers, Councillors, Ward Education Coordinators, Teachers and School Administrators, Religious Leaders, and others with influence in the communities, to increase their awareness of GBV in their communities and, in turn, to motivate their leadership in changing harmful socio-cultural gender norms and decreasing tolerance of GBV. The SASA! methodology and curriculum will be used to design and implement this component.

5. **Media campaigns**. Billboards, posters, radio messaging, and community drama will be used to raise awareness of GBV, available services, and GBV policies, laws and the legal protection for GBV survivors; challenge myths and stereotypes about GBV; and promote and encourage active GBV bystander interventions. Media campaign messages and approaches will be coordinated with the work of the community activists and the program for local leaders to reinforce key messages to the community and motivate proposed actions. [Note: It is possible that media campaigns through radio messaging may reach communities within the control clusters, and thus, lead to contamination of the control communities with regard to the intervention. However, the control clusters will not be exposed to messaging delivered through other channels and the other reinforcing community interventions. The effect on study outcomes, therefore, is expected to be minimal.

6. **Training workshops for the justice sector**. Judges, magistrates, prosecutors and lawyers will be trained on the emotional, psychological dimensions of GBV vis-a-vis the survivors and on how to improve their interactions with and management of individuals

and families that have experienced GBV, including referral to health, counseling, and social support services

Community-based interventions will be delivered in the same geographic locations (i.e., administrative wards) as the facility-based services. Emphasis will be placed on supporting a network of linked services and programs in accordance with the multicomponent GBV program model.

The implementing partners in Mbeya will receive additional technical assistance in program implementation from other PEPFAR-funded partners in Tanzania for GBV community and facility-based interventions, and in routine program M&E. Communication protocols have been established between the evaluation team, WRP, implementing program partners, and technical assistance partners to ensure coordination for efficient conduct of the study.

3.5. Study Design

The study will employ a pair-matched cluster randomized trial design to determine the effectiveness of the comprehensive GBV program (Hayes and Moulton, 2009). Randomization at the community rather than individual level is appropriate, since the intervention will be delivered at both community and facility levels.

In this study, a cluster is defined as a Health Center or District Hospital and the communities (defined by administrative ward or wards) that the Health Center or District Hospital serves. Clusters will be selected such that sufficient geographical distance exists between them to minimize cross-cluster contamination that might arise if the population within a given cluster utilizes health facilities within a nearby cluster.

A total of 12 clusters will be defined from locations within the Mbeya Region where WRP currently supports HIV activities. These include 20 Health Centers and 17 Hospitals. These facilities are located in all seven administrative districts and Mbeya City. The facility-based intervention also will be implemented at Mbeya Referral Hospital and Mbeya Regional Hospital in Mbeya City because these hospitals serve as referral facilities for the entire region.

The 12 clusters will be grouped into six matched pairs based on matching factors hypothesized to affect the study outcomes. Key factors to be considered include population density (urban, periurban, or rural), client volume at the health facility (i.e., Health Center or Hospital), availability of GBV services outside of the health facility, and geographic size of the health facility's catchment area. Within each pair, one cluster will be randomized to the intervention group and the other will be assigned to the control group. Randomization within each pair will be implemented at the data management center at Muhimbili University of Health and Allied Sciences (MUHAS). Randomization and notification of sites will occur as close to the time of the GBV program roll-out as is feasible.

The intervention will consist of the immediate roll-out of the GBV program at the health facilities and communities within the six intervention clusters. Roll-out of the GBV program for the six control clusters will be delayed by approximately 20 to 24 months.

Primary outcome measures will be obtained over time from both intervention and control clusters. A cross-sectional household survey will be conducted within all 12 clusters at baseline (prior to initiation of the intervention) and at endline (24 months later). Utilization of health facility services will be tallied from health facility GBV registers on a monthly basis and captured quarterly during the course of the study. Secondary outcomes also will be assessed through these and other data collection methodologies and sources, as described in Section 3.7.

3.6. Study Outcomes

The study will include primary and secondary outcomes hypothesized to form a causal pathway from program interventions to shorter- and longer-term GBV and HIV-related outcomes. The conceptual framework summarizing these outcomes and pathways is presented in Exhibit 1.

Exhibit 1. Conceptual framework

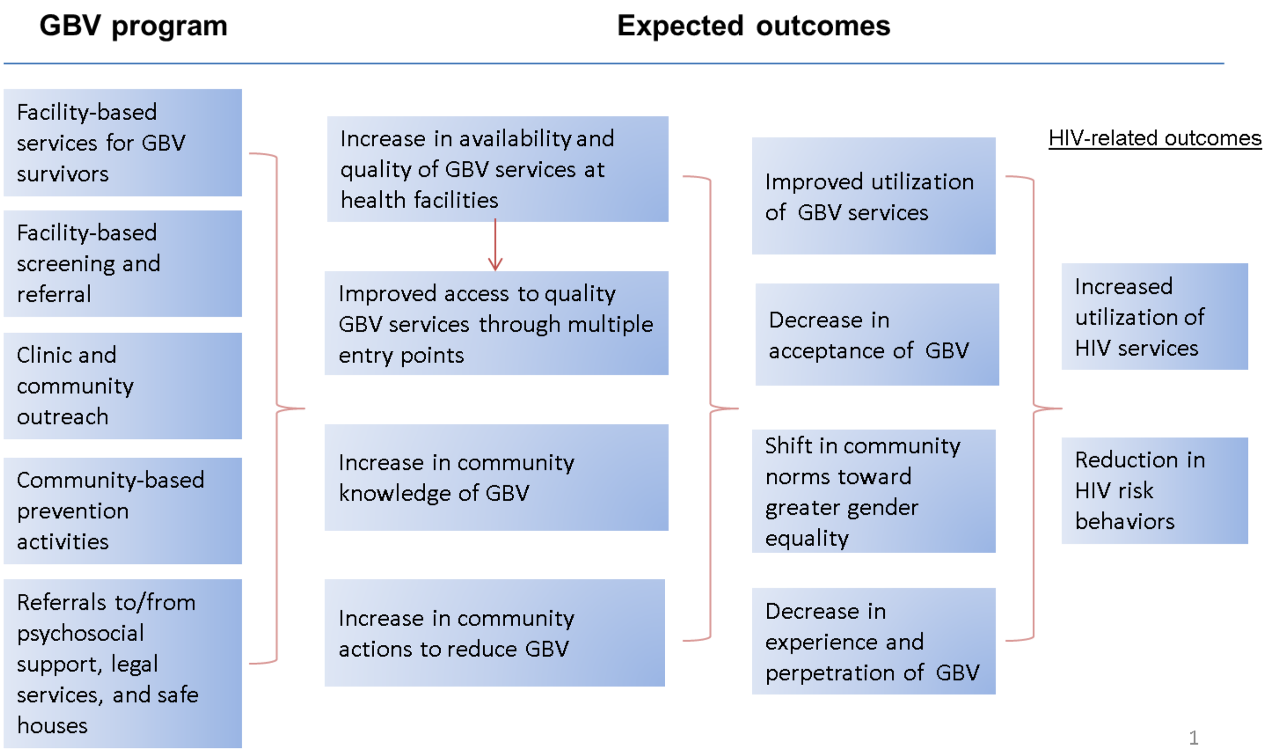

In brief, strengthening of facility-based services for GBV survivors and screening and referral are expected to lead to an increase in the availability and quality of GBV services at health facilities. Increased availability and quality of services, in combination with outreach and referrals to/from service entry points outside of health facilities, are expected to improve access to GBV services. At the same time, all program components are expected to lead to increases in community knowledge of GBV and community actions to reduce GBV. These intermediate outcomes then collectively are hypothesized to result in improved utilization of GBV health services, decrease in community acceptance of GBV, shift in community norms toward greater gender equality, and decline in GBV experience and perpetration. These changes in GBV outcomes also are hypothesized to lead to positive HIV-related outcomes, including increases in

utilization of HIV services and reduction in HIV risk behaviors, both in the near and longer terms.

Below are the three primary outcomes on which the study will be powered:

- Recent experience of IPV (sexual, physical, or emotional) among females ages 15–49 years;
- Utilization of GBV services at Health Centers and Hospitals (by females and males, all ages); and
- Delivery of comprehensive SV services at Health Centers and Hospitals.

A summary of measurements for these three primary outcomes, together with data collection methods, target population or data source, and timing of data collection, are presented in Table 3.

Table 3. Primary study outcomes and measurements

Outcome	Data collection method/instrument	Target population/Data source	Timing
**Recent experience of IPV:** Experience of sexual, physical, or emotional violence from an intimate partner in the past 12 months	Household survey	Females ages 15–49 years in an intimate partnership within the past 12 months	Baseline (BL), Endline (EL)
**Utilization of GBV services at Health Centers and Hospitals:** Number of client encounters (male and female, all ages) for GBV at Hospitals or Health Centers for any form of GBV	Health facility GBV registers	Adult and child GBV survivors seeking services at Health Centers and Hospitals in all study clusters	Monthly
**Delivery of comprehensive SV services at Health Centers and Hospitals:** Number (and %) of SV clients at Health Centers and Hospitals for whom comprehensive SV services were delivered according to MOHSW guidelines	Health facility GBV registers	Adult and child SV clients at Health Centers and Hospitals in all study clusters	Monthly

Outcomes and measurements for secondary outcomes of the study are presented in Table 4.

Table 4. Secondary study outcomes and measurements

Outcome	Data collection method	Target population/data source	Timing
**Recent Experience and Perpetration of GBV**			
Experience of IPV in past 12 months by type: sexual, physical, emotional	Household survey	Females ages 15–49 years in an intimate partnership within the past 12 months	BL, EL

Outcome	Data collection method	Target population/data source	Timing
Experience of GBV in past 12 months by type: sexual, physical, emotional	Household survey	Females ages 15–49 years	BL, EL
Experience of controlling behaviors by partner in the past 12 months	Household survey	Females ages 15–49 years in an intimate partnership within the past 12 months	BL, EL
**Knowledge, Attitudes, and Norms Regarding GBV and Gender Equality**			
Knowledge about GBV	Household survey	Females ages 15–49 years	BL, EL
	KII	Community leaders/gatekeepers and participants of the prevention and community programs in intervention clusters	Second half of study period
Attitudes toward/acceptability of GBV/stigma	Household survey	Females ages 15–49 years	BL, EL
	KII	Community leaders/gatekeepers and participants of the prevention and community programs in intervention clusters	Second half of study period
**Community-led Actions to Address GBV**			
Community mobilization and facility outreach activities	Household survey	Females ages 15–49 years	BL, EL
	KII	Community leaders/gatekeepers and participants of the prevention and community programs in intervention clusters	Second half of study period
	Health facility assessments	All health facilities in study clusters	BL,EL
	Routine M&E, including PEPFAR GBV indicators	Intervention clusters	Semi-annually
Gender norms	Household survey	Females ages 15–49 years	BL, EL
	KII	Community leaders/gatekeepers and participants of the prevention and community programs in intervention clusters	Second half of study period
**Utilization and Knowledge of GBV Services**			
Number of client encounters (male and female, all ages) for GBV at Hospitals or Health Centers by form of GBV (sexual, physical, emotional)	Health facility GBV registers	Adult and child GBV survivors seeking services at health facilities in all study clusters	Monthly
Reported use of GBV services in past 12 months —by type and source of service	Household survey	Females ages 15–49 years, who have experienced GBV in past 12 months	BL, EL
Knowledge of GBV services (what they are, where to go); barriers to utilization of GBV services	Household survey	Females ages 15–49	BL, EL
	KII	Community leaders/gatekeepers and participants of the prevention	Second half of study period

Outcome	Data collection method	Target population/data source	Timing
		and community programs in intervention clusters	
Number of GBV clients referred to health facilities from other source/facilities (by type)	Health facility GBV registers	Adult and child GBV clients at Hospitals and Health Centers at all study sites	Monthly
**GBV Service Delivery at Health Facilities**			
SV service delivery score (reflecting number of component services delivered for each SV client encounter)	Health facility GBV registers	Adult and child SV clients at Health Centers and Hospitals in all study clusters	Monthly
Number (and %) of GBV client encounters at the health facility for which services were delivered according to MOHSW guidelines	Health facility GBV registers	Adult and child GBV survivors seeking services at health facilities in all study clusters	Monthly
Number of clients (male and female, all ages) screened for GBV and referred to GBV services	Health facility GBV registers	All health facilities in the study clusters	Monthly
Number and types of services to which GBV clients were referred by health facilities	Health facility GBV registers	All health facilities in the study clusters	Monthly
Reported components of GBV services offered (by type of service and source)	Household survey	Females ages 15–49 who have sought services for GBV in past 12 months	BL, EL
Number and types of services to which GBV clients were referred by providers of services outside of health facilities	Key informant interviews	Community leaders/gatekeepers in intervention clusters	Second half of study period
**GBV Service and Program Capacity and Coverage**			
Health facility capacity and infrastructure; knowledge, skills, attitudes of providers at health facilities	Facility assessments	All health facilities in study clusters	BL, EL
Number of people reached by an individual, small group, or community-level GBV intervention	Routine M&E	All community intervention programs in intervention sites	Semi-annually
Awareness of and participation in individual, small group, or community-level GBV intervention in past 12 months within a community	Household survey	Females ages 15–49 years	BL, EL
Also see outcomes above for GBV Service Delivery at Health Facilities			
**HIV-related Outcomes**			
Engagement in HIV risk behaviors in last 12 months	Household survey	Females ages 15–49 years	BL, EL
Utilization of HIV services in past 12 months(by type of service)	Household survey	Females ages 15–49 years	BL, EL
	Routine M&E	All study health facilities over study period	Semi-annually

Outcome	Data collection method	Target population/data source	Timing
**Program Implementation**			
Program effort/inputs<br>Successes and challenges<br>Corrective actions taken	Key informant interviews<br>Program workplans	Program managers	4 mos, EL
	Health facility assessments	Facility managers, service providers	BL, EL
Program coverage	Routine M&E	Program data	Semi-annually

3.7. Data Collection and Sampling

Data collection in the study will include five components:

1. Household surveys of females ages 15–49 years;
2. Key informant interviews with community leaders/gatekeepers and participants of the prevention and community programs;
3. Health facility GBV registers: data extraction/review;
4. Health facility assessments; and
5. Key informant interviews with program managers.[2]

Each of these components is described below. The data collection instruments are provided in Annex B. These instruments are in draft form and will be finalized following pre-testing. Subsequently, they will be translated into Kiswahili.

3.7.1. Household surveys of females ages 15–49 years

The study team will conduct a cross-sectional survey of 100 households within each of the 12 clusters at baseline (prior to initiation of the intervention) and approximately 24 months later (at endline). For each household, the team will identify eligible members and select a single member at random to complete the household survey through a one-on-one interview. An eligible member is defined as a female ages 15 through 49 years at the time of the survey. This age group has been selected to represent those at high risk of GBV and to facilitate comparison of results with previous studies, such as the TDHS.

The study team will use a multistage stratified random sampling process to sample households. Within each of the 12 study clusters, it will obtain a list of households from the most recent census and generate a random sample of 130 households. The team will oversample by 30 percent to ensure that approximately 100 households are identified in which at least one female between the ages of 15 and 49 resides and agrees to participate in the survey. A household selection form similar to that employed by the DHS will be used to ascertain whether the selected household has any eligible members (see Annex B-1). When the team identifies more than one eligible household member, one will be chosen randomly for the interview, with no substitution if that person refuses to participate, or if parental consent is not given in the case of minors who have been selected for the interview. If an interview is not completed on the first visit, team members will make further attempts with the sampled household or respondent up to

[2] The mapping of GBV services outside of health facilities is now being conducted by WRP and is not part of the study.

three times over three different days before classifying the case as non-response. The team will schedule subsequent contacts at times when the respondent is more likely to be at home.

The sample size of 100 household surveys per cluster for 12 clusters is based on the formula for a pair-matched cluster randomized trial with a binomial outcome given in Hayes and Bennett (1999). Table 5 shows the minimum percentage reduction in IPV and GBV after 18 months of delivery of the comprehensive GBV program that can be detected with 80 percent power using a two-sided 0.05 level test for varying within cluster pair coefficients of variation (k_m).

In the table, estimates for the proportion of women experiencing IPV and GBV in the past 12 months in the control clusters are based on the Mbeya findings from the 2010 TDHS. Based on data from trials such as SASA! (Abramsky et al., 2012), the coefficient of variation is expected to be in the range of 0.1 to 0.4. If the coefficient of variation is close to 0.1, then percentage reductions in IPV and GBV on the order of 30 to 40 percent should be detectable. However, if the coefficient is closer to 0.4, then only large reductions on the order of 65 percent will be detectable.

Table 5. Minimum Effect Size Detectable in the Study[1]

Endpoint/ Outcome	Household Surveys per Cluster	Estimated Final Sample Size[2]	Estimated Prevalence in Control Cluster	Within-Pair Coefficient of Variation (k_m)	Minimum Effect Size	
					Prevalence in Treated Cluster	Relative Reduction (%)
Recent experience of IPV (any form)[3]	100	75	55%	0.10	39%	29.1%
				0.20	33%	40.0%
				0.30	27%	50.9%
				0.40	20%	63.6%
Recent experience of physical IPV[3]	100	75	50%	0.10	34%	32.0%
				0.20	30%	40.0%
				0.30	24%	52.0%
				0.40	18%	64.0%
Recent experience of sexual IPV[3]	100	75	35%	0.10	22%	37.1%
				0.20	19%	45.7%
				0.30	16%	54.3%
				0.40	12%	65.7%
Recent experience of GBV	100	100	50%	0.10	36%	28.0%
				0.20	30%	40.0%
				0.30	24%	52.0%
				0.40	18%	64.0%
Recent experience of physical GBV	100	100	45%	0.10	31%	31.1%
				0.20	27%	40.0%
				0.30	22%	51.1%
				0.40	16%	64.4%
Recent experience	100	100	30%	0.10	19%	36.7%
				0.20	17%	43.3%

of sexual				0.30	13%	56.7%
GBV				0.40	10%	66.7%
[1]For a two-sided 0.05-level test with 80 percent power.<br>[2]Assumes that 75 percent of women sampled are or have been in a partnership in the past 12 months.<br>[3]Among women who are or have been in a partnership in the past 12 months.						

Questions on the occurrence of GBV are based on questions used in the 2010 TDHS, in which respondents were asked about their experiences of specific acts from partners and non-partners without reference to leading terms such as ‘abuse’ or ‘violence.’ The target population for the household survey was chosen to reflect the population most likely to have experienced gender-based violence—women ages 15–49 living within the defined study clusters. The sampling frame thus comprises households situated in all enumeration areas in which participants (in intervention clusters) or passive volunteers (in control clusters) live.

While the study would benefit from the inclusion of household survey information from men, they are not included for the following reasons. Specifically,

- A separate survey instrument would need to be developed for males as the questions that would be asked would differ from those asked of females. This would require additional effort and time beyond that which is needed for development of the female questionnaire as most of the GBV-related questions in the female survey are taken from existing, tested survey instruments. These survey instruments do not exist for men. Thus, more extensive development and pre-testing would be required for the male questionnaire.
- Separate sampling frames would be needed for women and men. Given the sensitive nature of the topic and ethics and safety considerations, men and women cannot be sampled from the same or nearby households.
- Additional numbers of individuals would need to be sampled in order to achieve the current estimated levels of statistical power, which are based among other factors on estimated prevalence of GBV among women. In other words, the current sample could not simply be distributed among males and females and still maintain the same statistical power of the study.
- A separate data collection team would need to be employed and trained as female interviewers could not interview males (and vice versa), and the questionnaires would differ.

Given the resources available for the study and the fact that prevalence of GBV in Mbeya is significantly higher among females compared to males among all ages, the experience of females was chosen over that of men as the focus of the household survey. Although the study will not collect data from males in a household survey, the facility-based component will collect data on experience of GBV among both males and females – and of all ages. Additionally, data on gender norms and attitudes will be collected from male participants in the GBV program through key informant interviews (described in the following section).

3.7.2. Key informant interviews with community leaders/gatekeepers and male participants of the prevention and community programs

The team will conduct all key informant interviews in private settings with no interference, so that participants can communicate freely. Interviews will be audio-recorded, and written notes will capture the information shared. The team will inform participants of the modalities for capturing information before the onset of data collection.

Two groups of key informants will be interviewed. The types of information that the team will collect from these groups are described below.

Community Leaders/Gatekeepers

Key informant interviews (KIIs) will be conducted with government officials and community leaders and gatekeepers, including ward leaders, police, religious leaders, traditional leaders, leaders of women's NGOs, managers/providers of GBV services outside of health facilities, and others. The purpose of the KIIs is to gather opinions about these individuals' experiences in handling GBV cases, their own attitudes toward GBV, and whether they believe attitudes have changed in the community during the study period. They also will be asked about the strengths and weaknesses of the GBV program, their suggestions for ongoing improvements, and any community-led actions to address GBV that came about due to implementation of the program. The study team will conduct approximately 20 KIIs within the intervention clusters during the second half of the study period. The interview guide is provided in Annex B-2.

Participants (female and male) of the prevention and community programs

The team will conduct qualitative key informant interviews (KIIs) with females and males who have participated in the prevention and/or community-based programs within the six intervention clusters. The purpose of these interviews is to learn about participants' experiences with the program, their personal experiences with GBV, how their program involvement may have influenced their attitudes and behaviors, and their suggestions for improving the GBV program. Results of these interviews will be pooled across clusters and used to help interpret the quantitative findings from the household surveys with women, and the data from the health facilities.

The study team will work with program implementers to select a purposeful sample of females and males aged 18-49 years old who are participating in, or who have been exposed to the community and/or prevention programs. In order to be eligible for selection, they will have participated in at least one program component delivered as part of the community program. Efforts will be made to select interviewees who represent a wide range of women and men in the community, for example, women and men across the selected age range, married and unmarried, and women and men with different educational levels and professional backgrounds.

The study team will conduct the KIIs during months 12-14 the study, before and separate from the when the team conducts of the end-line household survey for women. By interviewing participants at this time point, we will be able to select participants who have been a part of, or exposed to, the program for enough duration that changes could have taken effect. The study team will verify selected individuals' participation in the program(s) with program implementers (following the criteria noted above for selection) and also record the intensity and length of their program participation.

Interview topics will include:

- Knowledge of the comprehensive GBV program
- Awareness, norms, and attitudes about gender and GBV
- Response to GBV
- Experience of GBV
- Knowledge of and experience with GBV services

Questions will focus on those related to the individual's perceptions and self-reported changes in his knowledge, perceptions, attitudes, and behaviors as a result of the program. Interviewees also will be asked to describe changes they have seen in the community that also may be related to the GBV program.

The team will conduct a total of 48 interviews (8 per each intervention cluster). The interview guide is provided in Annex B-3.

3.7.3 Health facility GBV registers: Data extraction and review

The national GBV register, as developed by the MOHSW, will be adapted and implemented at each of the health facilities in both intervention and control clusters. Adaptations to the existing register, which currently collects information on socio-demographic data, type of violence, and some elements of care provided, will include additional information on date and time of GBV act, type of GBV experienced, information on HIV testing, and data reflecting where the client was referred and any referrals provided. The GBV register is provided in Annex B-4.

Healthcare providers at the health facilities will complete the GBV register on an ongoing basis and fill it in for all GBV clients screened (adult and child, male and female) and treated for GBV at the health facility. GBV registers from the various health facility departments will be collected and stored in a central location at each facility. The registers will be printed in duplicate form. One copy will remain at the health facility and the study team will collect the other copy. Registers from all health facilities will be collected on a monthly basis and sent to the study office at MUHAS in Dar es Salaam for data entry. The register forms will be produced so that the study team's copy will exclude client-identifying information.

Training for providers on the use of the registers will occur as part of GBV service delivery training. The study team, in collaboration with the RMO, WRP, and PEPFAR-supported partners providing technical assistance for GBV M&E, will provide additional training in the month prior to the start of the study. Participants will include healthcare providers from facilities in the control clusters. The study team, in collaboration with the M&E officer at the WRP, will provide periodic technical support to ensure completeness and accuracy of data recording in the GBV register.

Data collection using the Tathmini GBV register will continue for 16 months, at which time facilities will transition to the national GBV register. For months 17-24 of the 24-month evaluation period, we will use national register data reports submitted by facilities through the Health Management Information System to triangulate facility and household survey endline data.

3.7.4. Health facility assessments

The study team will conduct a health facility assessment at the Health Centers and Hospitals in the study. This assessment aims to document health facility readiness and capacity to deliver GBV services as stipulated in the 2011 Tanzanian national GBV guidelines. The data will be used in the evaluation to assess service quality and its effects on study outcomes. The team will conduct assessments at baseline and 14-16 months after the start of the comprehensive program.

The health facility assessment will collect information on infrastructure, supplies and equipment, staff training, and provider knowledge and attitudes. Data will be collected through a combination of surveys of facility heads, observation at facilities, and interviews with staff involved in the provision of GBV services at point of first contact.

The assessment will include two components:

1) *Audits of health facilities through surveys of facility heads and study team observation*

For each health facility, the team will identify facility heads and obtain verbal consent to conduct the assessment and interview him or her. The team also will conduct survey interviews with the facility head at each Health Center and Hospital in the study clusters. The questionnaire is provided in Annex B-5. This survey is based on the tool that the MOHSW and PEPFAR-supported clinical partners developed to assess facility readiness to provide GBV services. It also includes a standardized observational checklist widely used by other researchers for similar purposes (Christofides et al., 2006).

2) *Key informant interviews with service providers*

At each facility, the team will interview one or two key staff involved in the provision of services related to GBV and selected by the facility manager. Interview topics include provider knowledge, skills, attitudes, and working environment. Also included will be general understanding of GBV, perceptions and attitudes toward provision of GBV clinical services, training skills related to STI management, skills on provision of emergency contraception, and other topics. The provider's knowledge of the service delivery guidelines, attitudes toward providing services, and practices will be assessed using a structured questionnaire. The team also will ask open-ended questions to explore challenges and achievements at the facility level. The interview guide is provided in Annex B-6.

3.7.5. Key informant interviews with program managers

The study will include a process evaluation to characterize the roll out and implementation of the comprehensive program over the 12-month study period. Key informant interviews with program managers will be the primary data source. The key informants will be selected from two types of managers: 1) GBV program managers at WRP and 2) community program managers who are sub-partners of WRP. This information will be supplemented with information from the health facility assessments (e.g., interviews with facility managers and service providers) to describe the roll out, ongoing implementation, successes and challenges of all components of the comprehensive program.

Key informants at WRP will include the overall program manager, the clinical services manager, the community program manager, and the M&E officer. Two representatives from each of the sub-partner community program organizations will be interviewed. All will be interviewed at 4-6 months and again at 12 months. Interview topics will include:

- Roles and responsibilities
- Description of the overall program or component and workplan
- Resources allocated/utilized
- Progress vis a vis plans
- Implementation successes
- Implementation challenges
- Corrective actions taken
- Recommendations for program improvement

The interview guide is given in Annex B-7.

3.8. Routine M&E Data Collected by Organizations Implementing the Programs

Implementing partners will collect routine monitoring data, including three indicators being piloted by PEPFAR under the GBV initiative. Two of the indicators relate to facility-based services and are compatible with primary and secondary facility outcomes in this study. [3] The third indicator, which captures community coverage, is of particular importance and is as follows:

- Number of people reached by an individual, small group, or community-level intervention or service that explicitly addresses GBV and coercion

This indicator will be used to determine coverage and confirm and document study assumptions that community interventions are operating at scale (and if they are not, to inform the interpretation of our study outcomes).

The study also will draw from any project evaluation studies to be conducted by implementing partners to evaluate specific components of the comprehensive program model. The results of these evaluation studies will be used to interpret and triangulate the findings of the outcome evaluation.

All data collection activities involving human subjects are described in this protocol. Additional M&E activities carried out by WRP as part of this protocol will not involve human subjects. Rather they will comprise routine program data monitoring (e.g. document the number of workshops carried out, and the number of participants, etc.), but will not include the collection of research data from these participants. The study team will work closely with the WRP to coordinate M&E activities throughout the implementation of the study. At the design and planning stages, routine

[3] The two facility-based indicators are (1) number of GBV service encounters at a health facility and (2) percentage of health facilities with GBV and coercion services available.

communications have been established between the two parties and opportunities for M&E collaboration have been identified. In particular, at the facility level, where GBV register data will be reported through RMO (and WRP) structures in addition to being collected by the study team, arrangements are being made for collaborative data collection and capacity building of implementing partners. Similarly, WRP and Tathmini GBV will collaborate in the conduct of facility assessments. Similar arrangements and agreements will be developed for data collection and use on the community intervention side. Specific roles and responsibilities will be detailed in the operations manuals for the study. We will explore options for formalizing these arrangements, for example, through a memorandum of understanding.

3.9 Data Analysis

For the baseline household survey, all demographic variables will be summarized by intervention and control clusters. Demographic variables will include age, marital status, number of children, education, recent employment, and alcohol usage. For women who have ever been married or lived with a male partner, the education, employment, and alcohol usage of the most recent husband/partner will also be summarized. Those variables for which a significant imbalance occurs between intervention and control clusters will be identified and used in an adjusted analysis of the primary endpoint drawn from the endline household survey.

A logistic regression analysis of the matching factors on the proportion of women reporting IPV in the past 12 months will be performed with the data from the baseline household survey. The multiple correlation coefficient R^2, which estimates the percentage variation in the outcome variable explained by the matching factors, will be calculated. If the estimated R^2 exceeds 0.5, a matched paired t-test will be utilized in the analysis of the primary endpoint from the endline household survey. Otherwise, an unmatched t-test will be used to assess the reduction in physical or sexual violence due to the intervention. Breaking the matches when the matching factors are poor or weak predictors of the outcome should increase the statistical power of the study (Diehr et al., 1995). Since the multiple correlation coefficient is based on the baseline survey, not the endline survey, this approach should not inflate the Type 1 error probability.

For the endline household survey, the primary analysis will be based on the intention-to-treat principle, in which all surveys are included in the analysis regardless of compliance with the study protocol. Additional analyses, in which surveys are excluded for reasons of non-compliance, including lack of exposure to the intervention, will be performed as part of a sensitivity analysis.

Depending on the degree to which the matching factors predict the primary outcome, either a paired t-test, in which the matching is preserved, or a two-sample t-test, in which clusters are unmatched, will be used to assess the association between the intervention and report of IPV in the past 12 months. The appropriate t-test will use cluster summaries of the proportion of women reporting IPV and, for the primary analysis, will not adjust for imbalances in demographic variables or other potential confounders. The estimated difference in the proportion of women reporting IPV between the intervention and control groups, along with its two-sided 95 percent confidence interval, will be reported. Also, the Mantel-Hanzel estimate of the common odds ratio and its two-sided 95 percent confidence interval will be provided.

As part of a sensitivity analysis, an analysis in which an adjustment is made for the imbalance in demographic variables will be conducted using the two-stage procedure suggested by Hayes and Moulton (2009). Briefly, a logistic regression model is fit to the individual-level data, in which the explanatory variables are the demographic variables for which the baseline survey found an imbalance. No intervention effect is included in the model. For each individual, her predicted outcome is determined. Then, for each cluster, the predicted number of outcomes is obtained by summing over the individuals in the cluster. The cluster-level difference residual R_d is calculated as the observed number of outcomes minus the predicted number divided by the number of surveys completed in the cluster. The adjusted t-test is then performed on the difference residuals. Hayes and Moulton further provide a formula for calculating a two-sided 95 percent confidence interval for the adjusted difference in the proportion of women reporting GBV between intervention and control groups.

Linear mixed effects (LME) models will be used to evaluate the utilization of GBV services at health facilities over time. From the monthly GBV registers maintained at each facility, the number of encounters at the facility in which a person reports GBV will be tabulated and used as the outcome variable. The number of encounters will be broken down further by sex, age group (i.e., <15, ≥15), and type of violence experienced (i.e., physical, sexual, or emotional) by the person reporting GBV. Fixed explanatory variables will include study month, type of facility, and an indicator variable for whether or not the facility is in an intervention cluster. Study month will be treated as a categorical variable to assess the seasonality of GBV as well as the impact of the availability of GBV services on utilization over time. Other explanatory variables will be explored, such as staffing levels and the number and mix of health facilities in a given cluster. A random effect for each facility will be included in the model to account for correlated outcomes within a facility over time. Suitable linear contrasts will be used to estimate and test the significance of the difference in GBV utilization between intervention and control clusters as well as the change in utilization between baseline and the end of the study.

LME models also will be used to assess the difference between intervention and control clusters as well as the change over time in the percentage of persons reporting SV for whom comprehensive services were delivered according to MOHSW guidelines. Since comprehensive services for SV survivors are not available at Dispensaries, these models will be restricted to GBV register data from Health Centers and Hospitals. The number of visits in which a person reported SV for a given study month and facility will be tabulated and treated as an offset variable. The number of visits in which comprehensive services were delivered according to MOHSW guidelines will be the outcome variable. Such models will allow an assessment of the proportion of SV survivors receiving comprehensive services by the end of the study.

For SV survivors seen at Health Centers or Hospitals, an additional score will be derived for each survivor that reflects the number of services received. This score will be used as an outcome variable in suitable LME models to assess the difference between intervention and control clusters as well as the change over time in the level of services provided to SV survivors.

The analysis of secondary endpoints from the household survey, GBV registers, key informant interviews, facility assessments, and services mapping will be provided in a separate and more detailed data analysis plan.

3.10 Data Management

The data will be housed in the project office in Dar es Salaam. The office has Internet, fax, phone, photocopy, printing, and computer facilities available to staff engaged on the project to allow for project monitoring. The office is also equipped with locked data storage cabinets to ensure the safety and security of the information collected from the field.

Data collection forms from the household survey will be reviewed in the field by the interviewers and a data collection supervisor for inconsistencies, errors, and missing data so as to immediately flag potential data quality issues. Data from the field will be transported in safe boxes to the project office. There, the study team data managers will review and code the data, conduct double entry of all the quantitative data, and conduct initial data cleaning simultaneously with data entry.

Recorded key informant interviews will be transcribed verbatim within two to five days after collection to keep abreast with what occurred during data collection. The transcribed text will be translated from Kiswahili into English by bilingual project staff members skilled in transcript translation. A sample of the translated material will be back-translated to Kiswahili by an independent qualitative skilled translator to check for accuracy and consistency. This information then will be entered and processed for analysis using NVIVO software.[4] Back-ups of audio tapes and transcribed data will be produced and stored separately in the secured storage cabinets.

Backup of electronic data will be done whenever newer entries are completed. Electronic data will be stored both on computer and external hard disk drives. Project computers will have log-in passwords, and external hard disk drives will be password protected and kept in locked cabinets. Data forms will be stored in locked cabinets. Only members of study team will have access to the data. Standard operating procedures and data quality checks will be developed and all data collection and data management team members will be trained on these procedures. The team's study coordinator will ensure compliance with all procedures.

3.11 Risk and Benefit

The proposed study will use a robust design to evaluate a comprehensive GBV program model of facility- and community-based interventions to provide information on the validity of the conceptual model and contribute new knowledge to the GBV program field. Results of the study will be used by the Tanzanian MOHSW; the Ministry of Gender, Children and Community Development; and other national policy and program actors to inform and strengthen GBV prevention and response services scale-up in Tanzania. The experience of monitoring community- and facility-based activities and outcomes in study clusters will strengthen M&E capacities at the regional, district, and national levels. This study also is designed to provide evidence to a broader set of global stakeholders, including PEPFAR; the Global Fund to Fight AIDS, TB and Malaria; other national governments; and other multilateral organizations on the impact of programs responding to GBV. These programs ultimately benefit adults and children most at risk of both GBV and HIV.

---

[4] NVIVO is a qualitative data analysis computer software package produced by QSR International.

All studies on GBV involving human subjects pose potential risks for study participants. For example, asking individuals to disclose difficult and painful experiences may create personal distress, and the act of research itself may put individuals at further risk of abuse. A respondent may suffer physical and/or emotional harm if a partner finds out that s/he has been talking to others about their relationship. Because many violent individuals closely control the actions of their partners, even the act of speaking to another person without permission may trigger violence. On the other hand, asking respondents to reveal stories of trauma can be a transforming experience for both the respondents and the interviewer. The experience of the study team and other researchers indicates that many women welcome the opportunity to tell their stories if asked in a sympathetic, nonjudgmental manner. Interviews also provide an opportunity to provide participants with information on GBV. The need to establish rapport with the respondent to ensure confidentiality and privacy during the interview is important for the entire survey, but is also critical for ensuring the validity of the data on GBV. Complete privacy is also essential to ensure the security of the respondent and the interviewer. Asking about or reporting violence, especially in households where the perpetrator may be present at the time of interview, may carry the risk of further violence.

The study team acknowledges its ethical responsibility to minimize these risks and maximize the benefits, and will conduct this study accordingly. Specifically, the study team will adhere to WHO guidelines regarding the conduct of research on violence against women (WHO, 1999). In this regard:

- The study team will consider the safety of respondents and the team as its highest priority in all project decisions.
- The proposed study design is methodologically sound and builds upon the research experience of the team and lessons learned from other researchers in the GBV field to minimize under-reporting of GBV.
- Confidentiality of participant identity and data will be safeguarded to ensure both participants' safety and data quality.
- All current research team members are experienced in GBV research and have undergone trainings in research ethics; all additional team members will be carefully selected, certified in research ethics, and receive specialized training and ongoing support.
- The study design includes a number of actions aimed at reducing any possible distress caused to the participants by the research.
- If for any reason privacy cannot be assured the interviewer will be trained not to proceed with the interview on GBV.
- Fieldworkers will be provided with information on available local GBV support services and resources, and trained to refer study participants for assistance.
- The study team will develop and implement a dissemination plan that will help to ensure that findings are properly interpreted and used to advance policy and intervention development within Tanzania and globally.

The study team will inform participants that their participation will assist the team in learning how to provide services that can improve the health and well-being of communities in Mbeya and throughout Tanzania. Participants also will be told that they may not benefit directly from their participation in the study and that no monetary compensation will be provided to them. The team also will inform participants that some of the questions will cover intimate topics and may be embarrassing or make them feel uncomfortable. Participants will be advised to respond to questions according to their own comfort level. All study participants will be advised prior to participating in data collection that they have the right to withdraw from the study at any time or refuse to answer any questions with which they may feel uncomfortable.

All interviewers will be of the same sex as the respondent and trained in ethical procedures, the informed consent process, and confidentiality. Training will include specific considerations for research on GBV using existing guidelines (Ellsberg and Heise, 2005). Above all, interviewers will be trained to ensure safety for the participant and the highest level of privacy. Interviewers also will be trained to stop the interview and offer the client a referral to services if the participant is visibly distressed. The study team will comprise interviewers from areas outside of Mbeya to ensure that participants are comfortable in discussing personal issues, and to minimize the possibility of a breach of confidentiality.

Steps that will be taken to minimize harm to study participants

- Only one woman per household will be interviewed in the survey.
- The wider community will not be informed that the household survey focuses on GBV.
- Male key informants will not come from the same households in which the survey has been conducted and women have been asked about violence.
- All interviews will be conducted in complete privacy.
- Dummy questionnaires may be used if others enter the room during the interview.
- Interviewers will be trained to recognize and deal with a respondent's distress during the interview and provide referrrals, as appropriate.
- All interviews will end on a positive note that emphasizes the respondent's strengths.

Interviewers will be advised that discussion of sensitive issues such as GBV may cause discomfort for them as well. Although GBV-related issues have been addressed widely both in the media and research findings, hearing first-person accounts may evoke strong emotions during some interviews. Members of the study team will be provided with the opportunity to debrief each other regularly during the research process and will be provided with details on how to seek counseling should a team member wish to do so. Two of the study co-investigators are trained and practicing mental health specialists, and are available to provide counseling support to team members.

During the initial design phase of the study, the study team consulted broad groups of stakeholders at the national and Mbeya regional levels regarding the proposed study outcomes, design, and conduct. Stakeholders included among others Tanzanian government officials and leads for the roll-out of GBV services in health facilities, program implementing partners, community and religious leaders, policymakers, health care providers, and U.S. government agency and multi-lateral donor representatives. The study team will establish a Technical Advisory Group that will serve to assist the team in addressing technical issues that may arise during the conduct of the study and provide comments on study results and interpretation. Approximately six representatives

from among stakeholders consulted earlier will serve on the TAG. The study team also will liaise with Community Advisory Boards (CAB) in Mbeya, established by WRP, to serve as community monitor for the study and to build support for the study within the cluster communities. The study team will meet with CAB representatives during quarterly monitoring visits establish routine communication mechanisms including email for more frequent updates on study progress and feedback from the CAB.

3.12 Confidentiality

No individual GBV survivors will be targeted directly in this study. None of the data collected, including GBV registers from health facilities, household survey questionnaires, key informant interview notes, and audio tapes, will include personal identification information. Participants and records of cases will be identified only by record numbers assigned at the facility level or by a study number assigned by the research team.

To minimize the risk of breach of confidentiality, the team will conduct household survey interviews in a private location within the household setting; no partners or other family members will be present. The team will not record names on the questionnaires or other data collection forms. The team member will give each participant a study identification number that will be used in place of his or her name on the interview form. Only study team members will have access to the questionnaires and data collection forms. The questionnaires and forms will be kept in a locked cabinet at the MUHAS offices in Dar es Salaam. The team will inform those participating in key informant interviews that the discussions may be recorded, and that the recordings will be used for research purposes only and destroyed once the information has been transcribed.

Participants will be asked to acknowledge the possibility that an interview may be requested by a representative of the trial's sponsor to determine whether they gave informed consent to the study team. If the sponsor requests an interview, participants will be informed that they have the option to accept or refuse the interview.

Interviewers having at minimum bachelor's degrees will be trained intensively to develop and refine research skills over a period of five days. This training will include (1) understanding the concept of GBV, its relationship with HIV, and its effects; (2) developing and improving interviewing skills, both quantitative and qualitative; (3) understanding special considerations in conducting research on GBV and applying skills; and (4) understanding and practicing on the specific research tools for the study. Furthermore, staff will undergo a research ethics course and certification test. The training will highlight the topic of confidentiality and its importance.

The data collection team also will be trained to provide participants with information about GBV services in their communities and to refer participants, if the participants so choose. A cluster-specific list of GBV support services will be developed from the services mapping activity. This list will be given to all study participants. In accordance with GBV research best practice and approaches previously employed in Tanzania (e.g., in the VAC survey), the list of services will also include services not directly associated with GBV (e.g., malaria, HIV/AIDS, alcohol use, etc.). This will be done to protect the privacy and safety of participants and minimize potential stigmatization and/or harm from others.

This list of services also will be used by the study team to refer participants to services, as needed. Following the guidelines used in the VAC survey, if a participant becomes upset during the interview the interviewer will offer to place the participant in direct contact with a service provider located near the participant. (Interviewers will be trained to recognize signs of anxiety and/or agitation during the interview process and to follow these steps). Further, for all participants who report experiencing GBV in the past 12 months, interviewers will be instructed to ask participants whether they currently feel safe in their living situation. If a respondent indicates that she does not feel safe, the interviewer will offer to place the participant in direct contact with someone who can help (a service provider). If a participant indicates that she would like a direct referral, the interviewer will ask permission to obtain her contact information, including her name and a safe place (way) where a service provider can find her. The interviewer will record the contact information on a separate form, which will not be linked in any way with the interview form. The interviewer will then give the contact form to the data collection team leader, who will identify an appropriate service provider from the directory list and deliver the form directly to the provider. Information shared by the participant during the interview will not be shared with the service provider.

3.13 Compensation

Study participants will not be paid for their participation in the study. It is anticipated that household interviews will be conducted at or near participants' homes. However, participants who may need to travel to participate in key informant interviews may be provided with fare for public transport and refreshments.

4. Informed Consent

For all data collection methods involving interactions with participants (i.e., the household survey, key informant interviews, and facility assessments), the study team will inform participants about the objectives of the study and ask them for written informed consent prior to participating. For study participants ages 15–17, parental consent also will be required. A participant's autonomy to choose whether or not s/he wishes to be interviewed will be respected. The informed consent process will include a description of the purpose of the study, procedures to be followed, possible discomfort and risks, benefits, compensation, and confidentiality. The team also will tell participants that no monetary compensation will be provided for participation. Team members will obtain informed consent in the local language (Kiswahili).

4.1 Household surveys

The sample for the household surveys includes both minors (ages 15–17) and adults (ages 18–24). Parental/guardian consent will be obtained for the minors. The team will not interview nor obtain any other data directly from minors without their prior assent and parental/guardian consent. Three types of consent forms will be used for the household surveys:

1) Informed consent for females ages 18–49,
2) Minor assent for females ages 15–17, and
3) Parental consent for females ages 15–17.

For the household surveys, the consent forms will describe the program that is the focus of the study as aiming "to improve the health and well-being of women and children." They will not

include specific reference to GBV. The potential sensitivity of questions that will be asked, however, will be clearly communicated in the consent form. Language will be included that informs participants they are free to not answer the questions or to end the interview without penalty or loss of any benefits to which they are entitled. This approach is based on the following:

- The absence of consensus within the global GBV research community on the explicitness of violence language that should be used in the consent process (see Ellsberg and Heise, 2005; WHO, 1999). In particular, many researchers argue that it is sufficient (and advisable) to include only language that communicates the sensitivity of topics that will be raised. Key reasons cited in the literature for excluding explicit GBV language include protection of the participant from potential harm inflicted by others as a result of others more easily becoming aware that questions on GBV will be asked; and, poor validity of the study findings resulting from potentially high rates of refusal and bias in the response sample (Scholar et al, 1995).

- Similar concerns raised by the research community on violence against children (VAC). A recent review of literature published by the Technical Working Group on Data Collection on Violence against Children (CP MERG, 2012) highlights a lack of consensus on ethical guidance for VAC research and notes the problematic nature of the human subjects requirement for honesty and transparency about the nature of the research. Specifically, several studies reviewed note both child and adult participants may be vulnerable to further abuse or retribution as a consequence of participating in the research. Therefore, it may not be in the best interests of participants for the researchers to be explicit about the nature of the research. The literature review report concludes that terminology used in the information provided may in itself affect levels of anxiety, and sometimes labeling certain descriptors, for example labeling experiences as "domestic violence" or "abuse," may be stigmatizing. Other researchers have noted that given that a significant proportion of women and children are abused by people they know, the assumption that parents have the best interests of the child at heart cannot be assumed in VAC research as the parent may be abusive or have conflicting allegiances between the child and another abusive adult (Knight et al., 2000).

- Advisement by the study team's locally based co-PIs and MUHAS research ethics advisors to follow the practice previously approved and carried out in Tanzania for similar studies. These include the WHO multi-country violence against women (VAW) study (WHO, 2004), the 2009 Tanzania Violence Against Children (VAC) Study (UNICEF et al, 2011), and the 2010 Tanzania Demographic and Health Survey (Tanzania DHS, 2010). In all three of these studies, which included large scale surveys, the consent forms did not include any reference to violence or GBV. Rather, they utilized an oral consent process that referred to the survey as a study on women's (or children's) health. Additionally, the WHO VAW and UNICEF VAC study consent forms included language to indicate the studies also were interested in learning about "life experiences." The WHO VAW study consent also noted, "Some of the topics discussed may be personal and difficult to talk about, but many women have found it useful to have the opportunity to talk." In all three surveys, participants were told that they could end the interview at

any time or skip any question they did not want to answer. Other smaller studies on VAC in Tanzania have followed a similar approach.

The study team will use a two-stage "consent" process for the household surveys. The first stage comprises the consent process described above and signing of consent forms prior to the interview. The second stage involves introducing the concept of GBV immediately prior to asking questions about GBV experience and informing participants again about the sensitive nature of the questions, that they can end the interview at any time, or they can skip any question they do not want to answer. This approach was used in the WHO VAW Study and the Tanzania DHS. Researchers have noted that this approach allowed for rapport to develop before introducing the topic of violence, but still gave the participant the opportunity to opt out of the violence-related questions at the time they were introduced. These studies noted that this consenting process was well received by survey respondents in Tanzania.

4.2 Key informant interviews

Key informant interviews will be conducted with adults (ages 18–49) only. Since all key informants (i.e., community leaders/gatekeepers and male participants of the prevention and community programs, and health care providers at the study sites) will have some familiarity with the GBV program, the consent forms include language that conveys that the purpose of the study is to measure the effects of the GBV program. Like the household survey consent forms, the key informant consent forms include language on the potential sensitivity of the issues and the participant's choice to not answer the questions or to end the interview. Within the interview just prior to asking sensitive questions, the interviewer will remind the participant that s/he is free to not respond.

There are two key informant consent forms, one for community leaders/gatekeepers, participants of the prevention and community programs, and program managers; and the other for health care providers who will be interviewed as part of the facility assessment. The forms differ only in the language within the section, "Why have I been invited to take part?" and reflect the different perspectives sought from these groups.

4.3 Health facility GBV registers

The team will not request consent from individuals for data collected through the health facility GBV register. The facility-based services provided as part of the intervention are those included in established MOHSW national guidelines. Data for the study will be extracted by the service provider from routine client records that are maintained separate and apart from the study. Data forms from the registers collected by the study team will not include client names or other personal identification information.

4.4 Consent forms

Consent forms are provided in Annex A as follows:

Annex A-1a. Informed Consent Form for the Household Survey with Females ages 18–49 years (English and Kiswahili)

Annex A-1b. Minor Assent Form for the Household Survey for Females ages 15–17 years (English and Kiswahili)
Annex A-1c. Parental Consent Form for the Household Survey (for females ages 15–17 years) (English and Kiswahili)
Annex A-2. Informed Consent Form for Key Informant Interviews
Annex A-3. Informed Consent Form for Facility Assessment: Key Informant Interviews with Service Providers (English and Kiswahili)

5. Questionnaires

Questionnaires and other data collection tools to be used in the study are included in Annex B. They include:

Annex B-1a. Household Survey Questionnaire (English and Kiswahili): Baseline
Annex B-1b. Household Survey Questionnaire (English and Kiswahili): Endline
Annex B-2. Key informant interview guide: Community leaders/gate-keepers
Annex B-3. Key informant interview guide: Participants of the prevention and community programs
Annex B-4. Health facility GBV register (English and Kiswahili)
Annex B-5. Health Facility Audit Tool (English)
Annex B-6. Key informant interview guide: Service providers (English and Kiswahili)

Annex B-7. Key informant interview guide: Program managers (English)

6. Ethical Training Certification

All principal investigators and key study personnel have completed a research ethics training course and received certification. These certifications were received within the past three years. Certificates for these individuals are provided in Annex C.

References

Abramsky, T., C.H. Watts, C. Garcia-Moreno, K. Devries, L. Kiss, M. Ellsberg, H.A. Jansen, and L. Heise. 2011. "What Factors Are Associated with Recent Intimate Partner Violence? Findings from the WHO 39 Multi-country Study on Women's Health and Domestic Violence." *BMC Public Health*11: 109.

Abramsky, T., L. Francisco, L. Kiss, L. Michau, T. Musuya, D. Kaye, C. Watts. n.d. *SASA! Baseline Report*. Retrieved August 7, 2012 from http://www.raisingvoices.org/files/sasa/sasastudy/SASA!BaselineStudyReport.pdf.

Basile, K.C., and L.E. Saltzman. 2002. *Sexual Violence Surveillance: Uniform Definitions and Recommended Data Elements*. Version 1.0. Atlanta, GA: Centers for Disease Control and Prevention, National Center for Injury Prevention and Control.

Bott, S., A. Morrison, and M. Ellesberg. 2005. "Preventing and Responding to Gender-based Violence in the Middle and Low-income Countries: A Global Review and Analysis." *World Bank Working Paper Series 3618*. Washington, DC: World Bank.

Campbell, J., M. Baty, R. Ghandour, J. Stockman, L. Francisco, and J. Wagman. 2008. "The Intersection of Violence against Women and HIV/AIDS: A Review." *International Journal of Injury Control and Safety Promotion* 15(4): 221–231.

Christofides, N., R. Jewkes, J. Lopes, and E. Dartnall. 2006. *How to Conduct a Situation Analysis of Health Services for Survivors of Sexual Assault: Health Care Providers*. SVRI, MRC, SAGBVHI.

CP MERG. 2012. *Ethical principles, dilemmas and risks in collecting data on violence against children: A review of available literature*, Statistics and Monitoring Section/Division of Policy and Strategy, UNICEF, New York.

Diehr, P., D.C. Martin, T. Koepsell, and A. Cheadle. 1995. "Breaking the Matches in a Paired T-test for Community Interventions When the Number of Pairs Is Small." *Statistics in Medicine* 14: 1491–1504.

Dunbar, M.S., C. Maternowska, M.J. Kang, S.M. Laver, I. Mudekunye, and N.S Padian. 2009. "Findings from SHAZ!: A Feasibility Study of a Microcredit and Life-skills HIV Prevention Intervention to Reduce Risk Among Adolescent Female Orphans in Zimbabwe." *Journal of Prevention & Intervention in the Community*.

Dunkle, K.L., R.K. Jewkes, H.C. Brown, G.E. Gray, J.A. McIntryre, and S.D. Harlow. 2004. "Gender-based Violence, Relationship Power, and Risk of HIV Infection in Women Attending Antenatal Clinics in South Africa." *Lancet* 363: 1415–21.

Ecker, N. 1994. "Culture and Sexual Scripts Out of Africa. A North American Trainer's View of Taboos, Tradition, Trouble and Truth." *SIECUS Rep* 22(2): 16–21.

El-Bassel, N., L. Gilbert, V. Rajah, A. Foleno, and V. Frye. 2000. "Fear and Violence: Raising the HIV Stakes." *AIDS Educ Prev* 12: 154–70.

El-Bassel, N., L, Gilbert, E. Wu, H. Go, and J. Hill. 2005. "HIV and Intimate Partner Violence Among Methadone-maintained Women in New York City." *Social Science & Medicine* 61: 171– 83.

Ellsberg, M., and L. Heise. 2005. *Researching Violence Against Women: A Practical Guide for Researchers and Activists*. Washington, DC: WHO, PATH.

Ferdinand, D. 2009. A Manual for integrating the Programmes and Services of HIV and Violence against Women. Washington, DC: UNIFEM, Development connections.

Gilbert, L., N. El-Bassel, V. Rajah, A. Foleno, J. Fontdevila, V. Frye, and B.L. Richman. 2000. "The Converging Epidemics of Mood-altering-drug Use, HIV, HCV, and Partner Violence: A Conundrum for Methadone Maintenance Treatment." *Mt Sinai J Med* 67: 452–64.

Gupta, G.R. 2000. "Gender, Sexuality, and HIV/AIDS: The What, the Why, and the How." *Can HIV AIDS Policy Law Rev* 5: 86–93.

Hayes, R.J., and S. Bennett. 1999. "Simple Sample Size Calculation for Cluster-randomized Trials." *International Journal of Epidemiology* 28: 319–326.

Hayes, R.J., and L.H. Moulton. 2009. *Cluster Randomized Trials*. Chapman & Hall.

He, H., H.V. McCoy, S.J. Stevens, and M.J. Stark. 1998. "Violence and HIV Sexual Risk Behaviors Among Female Sex Partners of Male Drug Users." *Women Health* 27: 161–75.

Jewkes, R., K. Dunkle, M. Nduna, J. Levin, N. Jama, N. Khuzwayo, M. Koss, A. Puren, and N. Duvvury. 2006. "Factors Associated with HIV Sero-Status in Young Rural South African Women: Connections Between Intimate Partner Violence and HIV." *International Journal of Epidemiology* 35: 1461–1468.

Jewkes, R.K., K. Dunkle, M. Nduna, and N. Shai. 2010. "Intimate Partner Violence, Relationship Power Inequity, and Incidence of HIV Infection in Young Women in South Africa: A Cohort Study." *The Lancet* 376: 41–48.

Jewkes, R., and R. Morrell. 2010. "Gender and Sexuality: Emerging Perspectives from the Heterosexual Epidemic in South Africa and Implications for HIV Risk and Prevention." *J Int AIDS Soc* 13: 6.

Jewkes, R., M. Nduna, J. Levin, N. Jama, K. Dunkle, A. Puren, and N. Duvvury. 2008. "Impact of Stepping Stones on Incidence of HIV and HSV-2 and Sexual Behaviour in Rural South Africa: Cluster Randomised Controlled Trial." *BMJ*. 337: a506.

Jewkes, R., L. Penn-Kekana, and H. Rose-Junius. 2005. ''If They Rape Me, I Can't Blame Them.': Reflections on Gender in the Social Context of Child Rape in South Africa and Namibia." *Soc Sci Med* 61:1809–1820.

Jewkes, R., Y. Sikweyiya, R. Morrell, and K. Dunkle. 2011. "The Relationship Between Intimate Partner Violence, Rape and HIV Amongst South African Men: A Cross-Sectional Study." *PLoS ONE* 6(9): e24256. doi:10.1371/journal.pone.0024256.

Kaye, D. 2004. "Gender Inequality and Domestic Violence: Implications for Human Immunodeficiency Virus (HIV) Prevention." *African Health Sciences* 4(1): 67–70.

Keesbury, J., and I. Askew. 2010. *Comprehensive Responses to Gender-based Violence in Low- resource Settings: Lessons Learned from Implementation.* Lusaka, Zambia: Population Council.

Keesbury, J., G. Morgan, and B. Owino. 2011. "Is Repeat Use of Emergency Contraception Common Among Pharmacy Clients?" *Evidence from Kenya, Contraception* 83(4):346–351.

Kim, J.C., C.H. Watts, J.R. Hargreaves, L.X. Ndhlovu, G. Phetla, L.A. Morison, J. Busza, J.D. Porter, and P. Pronyk. 2007. "Understanding the Impact of a Microfinance-based Intervention on Women's

Empowerment and the Reduction of Intimate Partner Violence in South Africa." *Am J Public Health* 97: 1794–1802.

Kisanga, F., J. Mbwambo, N. Hogan, Nystrom L., M. Emmelin, and G. Lindmark. 2010. "Perceptions of Child Sexual Abuse—A Qualitative Interview Study with Representatives of the Socio-legal System in Urban Tanzania." *Journal of Child Sexual Abuse* 19(3): 290–309.

Kisanga, F., L. Nystrom, N. Hogan, and M. Emmelin. 2011. "Child Sexual Abuse: Community Concerns in Urban Tanzania." *Journal of Child Sexual Abuse* 20(2): 196–217.

Laisser, R., H. Lugina, G. Lindmark, L. Nystrom, and M. Emmelin. 2009. "Striving to Make a Difference: Health Care Worker Experiences with Intimate Partner Violence Clients in Tanzania." *Health Care for Women International* 30: 64–78.

Laisser, R. 2011. "Prevention of Intimate Partner Violence- Community Healthcare Workers' Perceptions in Urban Tanzania." Department of Public Health and Clinical Medicine. Epidemiology and Global Health. Umeå, Sweden: Umeå University, SE-901 87.

Laisser, R.M., L. Nyström, G. Lindmark, H.I. Lugina, and M. Emmelin. 2011. "Screening of Women for Intimate Partner Violence: A Pilot Intervention at an Outpatient Department in Tanzania." *Glob Health Action* 4: 7288.

Maman, S., J. Campbell, M.D. Sweat, and A.C. Gielen. 2000. "The Intersections of HIV and Violence: Directions for Future Research and Interventions." *Soc Sci Med* 50: 459–478.

Maman, S., J. Mbwambo, N.M. Hogan, G.P. Kilonzo, and M. Sweat. 2001. "Women's Barriers to HIV-1 Testing and Disclosure: Challenges for HIV-1 Voluntary Counseling and Testing." *AIDS Care* 13: 595–603.

Medley, A., C. Garcia-Moreno, S. McGill, and S. Maman. 2004. "Rates, Barriers and Outcomes of HIV Serostatus Disclosure Among Women in Developing Countries: Implications for Prevention of Mother-to-child Transmission Programmes." *Bull World Health Organ* 82: 299–307.

Meursing, K., T. Vos, O. Coutinho, M. Moyo, S. Mpofu, and O. Oneko, et al. 1995. "Child Sexual Abuse in Matabeleland, Zimbabwe." *Soc Sci Med* 41: 1693–1704.

Michau, L. 2008. *The SASA ! Activist Kit for Preventing Violence against Women and HIV.* Kampala, Uganda: Raising Voices.

Mullick, S., M. Teffo-Menziwa, E. Williams, and R. Jina. 2010. "Women and Sexual Violence." *South African Health Review* 49–58.

Njuki, R., J. Okal, C.E. Warren, F. Obare, T. Abuya, L. Kanya , B. Bellows, C.C. Undie, B. Bellows, and I. Askew. 2012. "Exploring the Effectiveness of the Output-based Aid Voucher Program to Increase Uptake of Gender-based Violence Recovery Services in Kenya: A Qualitative Evaluation." *BMC Public Health* 12(1): 426.

Pronyk, P., J. Hargreaves, J. Kim, L. Morison, G. Phetla, C. Watts, J. Busza, and J. Porter. 2006. "Effect of a Structural Intervention for the Prevention of Intimate-partner Violence and HIV in Rural South Africa: A Cluster Randomised Trial." *The Lancet, Elsevier* 368(9551): 1973–1983.

Pronyk, P.M., J.C. Kim, T. Abramsky, G. Phetla, J.R. Hargreaves, L.A. Morison, C. Watts, J. Busza, and J.D. Porter. 2008. "A Combined Microfinance and Training Intervention Can Reduce HIV Risk Behaviour in Young Female Participants." *AIDS* 22: 1659–1665.

Pulerwitz, J., and G. Barker. 2007. "Measuring Attitudes Toward Gender Norms Among Young Men in Brazil: Development and Psychometric Evaluation of the GEM Scale." *Men and Masculinities* (published online ahead of print, 18 May).

Socolar, R., D. Runyan, and L. Amaya-Jackson. 1995. "Methodological and ethical issues related to studying child maltreatment." *Journal of Family Issues*, 16, 565-586.

Slonim-Nevo, V., and L. Mukuka. 2007. "Child Abuse and AIDS-related Knowledge, Attitudes and Behavior Among Adolescents in Zambia." *Child Abuse and Neglect* 31: 143–159.

Tanzania Commission for AIDS (TACAIDS), Zanzibar AIDS Commission (ZAC), National Bureau of Statistics (NBS), Office of the Chief Government Statistician (OCGS), and Macro International Inc. 2008. *Tanzania HIV/AIDS and Malaria Indicator Survey 2007–08.* Dar es Salaam, Tanzania: TACAIDS, ZAC, NBS, OCGS, and Macro International Inc.

2010 Tanzania Demographic and Health Survey. 2010. Dar es Salaam, Tanzania: National Bureau of Statistics.

UNAIDS: Joint Action for Results: *UNAIDS Outcome Framework 2009–2011*. 2009. Geneva: UNAIDS.

United Nations Children's Fund, United Republic of Tanzania, CDC, Muhimbili University. 2011. *Violence Against Children in Tanzania: Findings from a National Survey 2009*.

UN Joint Programme on HIV/AIDS. 2010. *Global Report: UNAIDS Report on the Global AIDS Epidemic 2010*. UNAIDS.

United Republic of Tanzania (URT) 2011. Ministry of Health and Social Welfare. *National Management Guidelines for the Health Sector Response to and Prevention of Gender-based Violence (GBV).*

U.S. Government. May 2011. *Guidance for Global Health Initiative Country Strategies*, GHI Guidance 2.0. Retrieved August 10, 2012 from http://www.ghi.gov/resources/guidance/164904.htm.

Violence Against Women and HIV/AIDS: Critical Intersections – Sexual Violence in Conflict Settings and the Risk of HIV. 2004. Geneva: World Health Organization.

WHO. *Putting Women's Safety First: Ethical and Safety Recommendations for Research on Domestic Violence Against Women.* 1999. Report No.: WHO/EIP/GPE/99.2. Geneva: Global Programme on Evidence for Health Policy, World Health Organization.

WHO (editors). *Violence Against Women and HIV/AIDS: Setting the Research Agenda*. 2000. In Meeting Report. Geneva: WHO.

WHO, Department of Gender, Women and Health (GWH), and Department of HIV/AIDS (HIV). 2006. *Addressing Violence Against Women in HIV Testing and Counseling: A Meeting Report*. Geneva: WHO.

Wingood, G.M., and R.J. DiClemente. 1997. "The Effects of an Abusive Primary Partner on the Condom Use and Sexual Negotiation Practices of African-American Women." *American Journal of Public Health* 87: 1016–1018.

"Women Won't Wait." 2010. Retrieved August 1, 2012 from http://www.un.org/documents/ga/res/48/a48r104.htm.

ANNEX A-1a.

Informed Consent Form for the Household Survey with Females ages 18-49 years

English

Informed Consent Form for the Household Survey with Females ages 18-49 years

Introduction

You are invited to take part in a research study. Before you decide whether to participate, you need to understand why the research is being done and what it will involve. Please take the time to read or to listen as I read the following information. You may talk to others about the study if you wish. Please ask me if there is anything that is not clear, or if you would like more information. When all of your questions have been answered and you feel that you understand this study, you will be asked if you wish to participate in the study, and if yes, to sign this Informed Consent form. You will be given a signed copy to keep, if you would like one.

Purpose of the Study and Study Requirements

What is the study? The study is being conducted by a team of researchers from Muhimbili University of Health and Allied Sciences (MUHAS) in Dar es Salaam and the Futures Group, Population Council, and Pangaea Global AIDS Foundation in the United States. The overall purpose of the study is to measure the effect of a set of programs and services that will be delivered in health facilities and in the community to improve the health and well-being of women and children. The study will compare data from communities where these programs and services are being delivered with data from communities that aren't yet receiving these services to determine if the program had the desired effect.

The study has been funded by the United States Government.

Why have I been invited to take part? You have been invited to take part in the study because, as part of the data collection, we are visiting a number of households in the area where you live to interview one individual in each selected household. Your household has been selected by chance from a list of all households in your area. The information you give us regarding your knowledge and experiences with the services provided in your community will be important to understanding how these services are delivered and the effect they are having in the community. In addition the information you provide will assist the study team to understand what works and does not work in order to strengthen services in Mbeya and other regions.

What will happen if I take part? If you agree to take part in the study, we will ask you to sign this form indicating that you agree to be part of the study. An interviewer will sit with you and ask you a series of questions. The interviewer will record your responses on a form. The information that you provide will be kept completely confidential. Your responses will be combined with those of other participants in the study. Together this information will be analysed to determine if the programs and services are making a difference.

How long will the interview last? The interview will take between 60 and 90 minutes.

We may contact you again if we need to obtain further information or clarifications regarding the information you provide us with today.

Risks

What are the risks of the study?
If you decide to participate, you may be inconvenienced by the time and effort that it will take to complete the interview. You may find one or more questions that we ask to be sensitive. You do not have to respond to any question that makes you uncomfortable. You may end the interview at any time without penalty or loss of any benefits to which you are entitled.

Breach of confidentiality (that is, something you say in confidence during the interview that then accidentally becomes known to others) is a potential risk of your participation in the study. We will take precautions, however, to see that this does not happen.

Benefits

What are the benefits of participating? There may be no direct benefit to you for participating in the study. However, you may find an indirect benefit in knowing you have participated in an important study that could help others in the future. You may also appreciate the opportunity to share your experiences in confidence with another person. If you wish to receive it, we can also provide you with a list of relevant services in your area. You may also ask the interviewer to refer you for services if you wish to seek help. This information will not be shared with anyone.

Confidentiality

Will my participation in the study be kept confidential? The information that is collected during this interview will not include your name or any other information to personally identify you. Only members of the study team will have access to your questionnaire and it will be kept in a locked cabinet at the MUHAS offices in Dar es Salaam. You will not be identified in any reports or publications regarding the study. Your participation in the study will not be divulged to anyone.

Voluntary Participation

What are my rights as a study participant? Your participation in this study is completely voluntary. If you decide not to participate, you will not lose any existing benefits to which you are entitled. If you agree to participate in this study, you may end your participation at any time without penalty or loss of existing benefits to which you are entitled. If you decide to take part, you are free to refrain from answering any questions. You are free to withdraw at any time without affecting your relationship with the services you are entitled to as a community member.

Additional Information

What will I receive for participating? You will not receive any monetary or other incentive for participation.

What will happen to the results of the research study? The results and progress of the study will be presented in research publications and at meetings and conferences in Tanzania and in other countries. The Ministry of Health and Social Welfare (MOHSW), Regional Medical Office (RMO), and community partners will use the results to make improvements to their programs and services. In addition, the study progress and results will be put on a website so that others can learn from this study and strengthen services in their countries.

Who has reviewed the study for ethical issues? This study has been reviewed by research Institutional Review Boards at the Population Council, MUHAS, Tanzanian National Institute for Medical Research (NIMR), and Mbeya Research and Ethics Committee.

What if I need more information? If you have a concern about any aspect of the study, you should ask to speak to the researchers, who will do their best to answer your questions and address your concerns. You can contact Dr. Jessie Mbwambo, a psychiatrist at MUHAS and a principal investigator of the study, telephone number: +255 784 339 747; or Ms. Stella Mujaya, Study Coordinator, at the Futures Group, telephone number: +255 754 307 205.

What if there is a problem? For any concern you may have about the way you have been treated during the study or any possible harm you might suffer, please contact: Professor Mainen Moshi, Director of Research and Publications, MUHAS, Phone number: +255 222 150 302/6.

Subject Statement: I have read or listened to the Informed Consent for this study. I have received an explanation of the planned research, procedures, risks and benefits and privacy of my personal information. I agree to take part in this study. I understand that my participation in this study is voluntary.

Your name: ____

Your signature: ____**Date:**___________

Investigator or person who conducted Informed Consent discussion:
I confirm that I have personally explained the nature and extent of the planned research, study procedures, potential risks and benefits and confidentiality of personal information.

Name of person obtaining consent:_____________________________________

Signature of person obtaining consent: ____________________**Date:**___________

ANNEX A-1b.

Minor Assent Form for the Household Survey for Females ages 15-17 years

English

Minor Assent Form for the Household Survey for Females ages 15-17 years

You are invited to take part in a research study. Before you decide whether to participate, you need to understand why the research is being done and what it will involve. Please take the time to read or to listen as I read the following information. You may talk to others about the study if you wish. Please ask me if there is anything that is not clear, or if you would like more information. When all of your questions have been answered and you feel that you understand this study, you will be asked if you wish to participate in the study, and if yes, to sign this Informed Consent form. You will be given a signed copy to keep, if you would like one.

Your parent or guardian has already given permission for you to participate in the study. However, you do not have to say yes. We have talked to your parent or guardian and he/she agrees that you do not have to say yes to participate in the study.

Purpose of the Study and Study Requirements

What is the study? The study is being conducted by a team of researchers from Muhimbili University of Health and Allied Sciences (MUHAS) in Dar es Salaam and the Futures Group, Population Council, and Pangaea Global AIDS Foundation in the United States. The overall purpose of the study is to measure the effect of a set of programs and services that will be delivered in health facilities and in the community to improve the health and well-being of women and children. The study will compare data from communities where these programs and services are being delivered with data from communities that aren't yet receiving these services to determine if the program had the desired effect.

The study has been funded by the United States Government.

Why have I been invited to take part? You have been invited to take part in the study because, as part of the data collection, we are visiting a number of households in the area where you live, to interview one individual in each selected household. Your household has been selected by chance from a list of all households in your area. The information you give us regarding your knowledge and experiences with the services provided in your community will be important to understanding how these services are delivered and the effect they are having in the community. In addition the information you provide will assist the study team to understand what works and does not work in order to strengthen services in Mbeya and other regions.

What will happen if I take part? If you agree to take part in the study, we will ask you to sign this form indicating that you agree to be part of the study. An interviewer will sit with you and ask you a series of questions. The interviewer will record your responses on a form. The information that you provide will be kept completely confidential. Your responses will be combined with those of other participants in the study. Together this information will be analysed to determine if the programs and services are making a difference.

How long will the interview last? The interview will take between 60 and 90 minutes.

We may contact you again if we need to obtain further information or clarifications regarding the information you provide us with today.

Risks

What are the risks of the study? If you decide to participate, you may be inconvenienced by the time and effort it will take to complete the interview. You may find one or more questions that we ask to be sensitive. You do not have to respond to any question that makes you uncomfortable. You may end the interview at any time without penalty or loss of any benefits to which you are entitled.

Breach of confidentiality (that is, something you say in confidence during the interview that then accidentally becomes known to others) is a potential risk of your participation in the study. We will take precautions, however, to see that this does not happen.

Benefits

What are the benefits of participating? There may be no direct benefit to you for participating in the study. However, you may find an indirect benefit in knowing you have participated in an important study that could help others in the future. You may also appreciate the opportunity to share your experiences in confidence with another person. If you wish to receive it, we can also provide you with a list of relevant services in your area. You may also ask the interviewer to refer you for services if you wish to seek help. This information will not be shared with anyone.

Confidentiality

Will my participation in the study be kept confidential? The information that is collected during this interview will not include your name or any other information to personally identify you. Only members of the study team will have access to your questionnaire and it will be kept in a locked cabinet at the MUHAS offices in Dar es Salaam. You will not be identified in any reports or publications regarding the study. Your participation in the study will not be divulged to anyone. We will not share any of your information with your parents.

Voluntary Participation

What are my rights as a study participant? Your participation in this study is completely voluntary. If you decide not to participate, you will not lose any existing benefits to which you are entitled. If you agree to participate in this study, you may end your participation at any time without penalty or loss of existing benefits to which you are entitled. If you decide to take part, you are free to refrain from answering any questions. You are free to withdraw at any time without affecting your relationship with the services you are entitled to as a community member.

Additional Information

What will I receive for participating? You will not receive any monetary or other incentive for participation.

What will happen to the results of the research study? The results and progress of the study will be presented in research publications and at meetings and conferences in Tanzania and in other countries. The Ministry of Health and Social Welfare (MOHSW), Regional Medical Office (RMO), and community partners will use the results to make improvements to their programs and services. In addition, the study progress and results will be put on a website so that others can learn from this study and strengthen services in their countries.

Who has reviewed the study for ethical issues? This study has been reviewed by research Institutional Review Boards at the Population Council, MUHAS, the Tanzanian National Institute for Medical Research (NIMR), and Mbeya Research and Ethics Committee.

What if I need more information? If you have a concern about any aspect of the study, you should ask to speak to the researchers, who will do their best to answer your questions and address your concerns. You can contact Dr. Jessie Mbwambo, a psychiatrist at MUHAS and a principal investigator of the study, telephone number: +255 784 339 747; or Ms. Stella Mujaya, Study Coordinator, at the Futures Group, telephone number: +255 754 307205.

What if there is a problem? For any concern you may have about the way you have been treated during the study or any possible harm you might suffer, please contact: Professor Mainen Moshi, Director of Research and Publications, MUHAS, Phone number: +255 222 150 302/6.

Subject Statement: I have read or listened to the Assent Form information for this study. I have received an explanation of the planned research, procedures, risks and benefits, and privacy of my personal information. I agree to take part in this study. I understand that my participation in this study is voluntary.

Your name: ____

Your signature: ____**Date:**___________

Investigator or person who conducted Minor Assent discussion:
I confirm that I have personally explained the nature and extent of the planned research, study procedures, potential risks and benefits, and confidentiality of personal information.

Name of person obtaining consent: ____________________________________

Signature of person obtaining consent: ___________________**Date:**___________

ANNEX A-1c.

Parental Consent Form for the Household Survey (for females ages 15-17 years)

English

Parental Consent Form for the Household Survey (for females ages 15-17 years)

Your child is invited to take part in a research study. Before you decide whether to allow your child to participate, you need to understand why the research is being done and what it would involve. Please take the time to read or to listen as I read the following information. You may talk to others about the study if you wish. Please ask me if there is anything that is not clear, or if you would like more information. When all of your questions have been answered and you feel that you understand this study, you will be asked if you would like your child to participate in the study, and if yes to sign this Parental Consent form. You will be given a signed copy to keep, if you would like one. Your child will also be asked whether she agrees to participate in this study and to sign a consent form. We will conduct the interview only if she agrees to participate.

Purpose of the Study and Study Requirements

What is the study? The study is being conducted by a team of researchers from Muhimbili University of Health and Allied Sciences (MUHAS) in Dar es Salaam and the Futures Group, Population Council, and Pangaea Global AIDS Foundation in the United States. The overall purpose of the study is to measure the effect of a set of programs and services that will be delivered in health facilities and in the community to improve the health and well-being of women and children. The study will compare data from communities where these programs and services are being delivered with data from communities that aren't yet receiving these services to determine if the program had the desired effect.

The study has been funded by the United States Government.

Why has my child been invited to take part? Your child has been invited to take part in the study because, as part of the data collection, we are visiting a number of households in the area where you live to interview one individual in each selected household. Your household has been selected by chance from a list of all households in your area. The information your child gives us regarding her knowledge and experiences with the services provided in your community will be important to understand how these services are delivered and the effect they are having in the community. In addition, the information she provides will assist the study team to understand what works and does not work in order to strengthen services in Mbeya and other regions.

What will happen if my child takes part? If you agree to let your child take part in the study, we will ask you to sign this form. An interviewer will sit with your child and ask her a series of questions. The interviewer will record her responses on a form. The information that she provides will be kept completely confidential. Her responses will be combined with those of other participants in the study. Together this information will be analysed to determine if the programs and services are making a difference.

How long will the interview last? The interview with your child will take between 60 and 90 minutes.

We may contact you again if we need to obtain further information or clarifications regarding the information you provide us today.

Risks

What are the risks of the study? If you give permission for your child to participate, she may be inconvenienced by the time and effort it will take to complete the interview. She may find one or more questions that we ask to be sensitive. She does not have to respond to any question that makes her uncomfortable. She may end the interview at any time without penalty or loss of any

benefits to which she is entitled.

Breach of confidentiality (that is, something she says in confidence during the interview that then accidentally becomes known to others) is a potential risk of her participation in the study. We will take precautions, however, to see that this does not happen.

Benefits

What are the benefits of participating? There may be no direct benefits to you or your child for participating in the study. You and/or your child may find an indirect benefit in knowing your child has participated in an important study that could help others in the future. If you or your child wishes to receive a list of relevant services in your area, you will be provided with such a list from the interviewer. You may also ask the interviewer to refer you or your child for services if you wish to seek help. This information will not be shared with anyone.

Confidentiality

Will my child's participation in the study be kept confidential? The information that is collected during this interview will not include your name, your child's name or any other information to personally identify her. Only members of the study team will have access to your child's questionnaire and it will be kept in a locked cabinet at the MUHAS offices in Dar es Salaam. Your child will not be identified in any reports or publications regarding the study. Your child's participation in the study will not be divulged to anyone.

Voluntary Participation

What are my child's rights as a research participant? Your child's participation in this study is completely voluntary. If your child decides not to participate, she will not lose any existing benefits to which she is entitled. If you agree to let your child participate in this study, you or she may end her participation at any time without penalty or loss of existing benefits to which she is entitled.

Additional Information

What will my child receive for participating? Neither you nor your child will receive any monetary or other incentive for participation.

What will happen to the results of the research study? The results and progress of the study will be presented in research publications and at meetings and conferences in Tanzania and in other countries. The Ministry Of Health and Social Welfare (MOHSW), Regional Medical Office (RMO), and community partners will use the results to make improvements to their programs and services. In addition, the study progress and results will be put on a website so that others can learn from this study and strengthen services in their countries.

Who has reviewed the study for ethical issues? This study has been reviewed by Institutional Review Boards at the Population Council, MUHAS, the Tanzanian National Institute for Medical Research (NIMR), and the Mbeya Research and Ethics Committee.

What if I need more information? If you have a concern about any aspect of the study, you should ask to speak to the researchers who will do their best to answer your questions and address your concerns. You can contact: Dr. Jessie Mbwambo, a psychiatrist at MUHAS, and a principal investigator of the study, telephone number: +255 784 339 747, or Ms. Stella Mujaya, Study Coordinator, at the Futures Group, telephone number: +255 754 307205.

What if there is a problem? For any concern about the way your child has been treated during the study or any possible harm your child might suffer, please contact: Professor Mainen Moshi, Director of Research and Publications, MUHAS, Phone number: +255 222 150 302/6.

Subject Statement: I have read or listened to the Parental Consent for this study. I have received an explanation of the planned research, procedures, risks and benefits and privacy of my child's personal information. I agree to allow my child to take part in this study. I understand that my child's participation in this study is voluntary.

Your name: ____

Your signature: ____**Date:**____________

Investigator or person who conducted Informed Consent discussion:
I confirm that I have personally explained the nature and extent of the planned research, study procedures, potential risks and benefits, and confidentiality of personal information.

Name of person obtaining consent: ______________________________________

Signature of person obtaining consent: ____________________**Date:**____________

ANNEX A-2.

Informed Consent Form for Key Informant Interviews

ENGLISH

Informed Consent Form for Key Informant Interviews

Introduction

You are invited to take part in a research study. Before you decide whether to participate, you need to understand why the research is being done and what it would involve. Please take the time to read or to listen as I read the following information. You may talk to others about the study if you wish. Please ask me if there is anything that is not clear, or if you would like more information. When all of your questions have been answered and you feel that you understand this study, you will be asked if you wish to participate in the study, and if yes to sign this Informed Consent form. You will be given a signed copy to keep.

Purpose of the Study and Study Requirements

What is the study? The study is being conducted by a team of researchers from Muhimbili University of Health and Allied Sciences (MUHAS) in Dar es Salaam and the Futures Group, Population Council, and Pangaea Global AIDS Foundation in the United States. The overall purpose of the study is to measure the effect of a set of services to improve the health and well-being of women and children. These programs and services are intended to address gender-based violence (including physical, sexual, and emotional violence), which most typically takes the form of violence against women and children. The study will compare data from communities where these programs and services are being delivered with data from communities that aren't yet receiving these services to determine if the program had the desired effect.

The study has been funded by the United States Government.

Why have I been invited to take part? You have been invited to take part because the information you give us regarding your knowledge and experiences with the programs and services provided in your community will be important to understanding how these services are delivered and the effect they are having in the community. In addition, the information you provide will assist the study team to understand what works and does not work in order to strengthen services in Mbeya and other regions.

What will happen if I take part? If you agree to take part in the study, we will ask you to sign this form. You will also be asked to answer some questions regarding your knowledge, opinions, and experiences with programs and services that are being provided to address prevention and services for violence (including physical violence, sexual violence and other types of abuse).

How long will the focus group discussion last? This will take between 60-90 minutes.

We may contact you again if we need to obtain further information or clarifications regarding the information you provide us today.

Risks

What are the risks of the study?

An inconvenience may be the time and effort you take to be a participant. You may find one or more questions that we ask to be upsetting or emotionally sensitive particularly as the study focuses on violence against women and children and sexual violence. You do not have to respond to any question that makes you uncomfortable. You may end the interview at any time without penalty or loss of any benefits to which you are entitled.

A risk may be a breach of confidentiality (something you say during the interview) but we will take precautions to see that this does not happen.

Benefits

What are the benefits of participating? There are no direct benefits to you for participating in the study. You may find an indirect benefit in knowing you have participated in an important study that could help others in the future. The study will help the government of Tanzania to strengthen services to prevent and respond to violence against adults and children.

Confidentiality

Will my participation in the study be kept confidential? The information that is collected during this interview will not include your name, only your occupation or designation. Only members of the study team will have access to the information we record form the interview and that will be kept in a locked cabinet at the MUHAS offices in Dar es Salaam. You will not be identified in any reports or publications regarding the study. Your participation in the study will not be divulged to anyone else.

Voluntariness

What are my rights as a research participant/subject? Your participation in this study is completely voluntary. If you decide not to participate, you will not lose any existing benefits to which you are entitled. If you agree to participate in this study, you may end your participation at any time without penalty or loss of existing benefits to which you are entitled. If you decide to take part, you are free to refrain from answering any questions. You are free to withdraw at any time without affecting your relationship with the services you are entitled to as a community member.

Additional Information

What will I receive for participating? You will not receive any monetary or other incentive for participation.

What will happen to the results of the research study? The results and progress of the study will be presented in research publications and at meetings and conferences in Tanzania and in other countries. The MOHSW and community partners will use the results to make improvements to their programs and services. In addition, the study progress and results will be put on a website so that others can learn from this study and strengthen services in their countries.

Who has reviewed the study for ethical issues? This study has been reviewed by Institutional Review Boards at the Population Council, MUHAS, Tanzanian National Institute for Medical Research (NIMR), and Mbeya Research and Ethics Committee.

What if I need more information? If you have a concern about any aspect of the study, you should ask to speak to the researchers, who will do their best to answer your questions and address your concerns. You can contact Dr. Jessie Mbwambo, a psychiatrist at MUHAS and a principal investigator of the study, telephone number: +255 784 339 747; or Ms. Stella Mujaya, Study Coordinator, at the Futures Group, telephone number: +255 754 307 205.

What if there is a problem? For any concern you may have about the way you have been treated during the study or any possible harm you might suffer, please contact: Professor Mainen Moshi, Director of Research and Publications, MUHAS, Phone number: +255 222 150 302/6.

Subject Statement: I have read the Informed Consent for this study. I have received an explanation of the planned research, procedures, risks and benefits and privacy of my personal information. I agree to take part in this study. I understand that my participation in this study is voluntary.

Your name:____

Your signature:____**Date:**____________

Investigator or person who conducted Informed Consent discussion: I confirm that I have personally explained the nature and extent of the planned research, study procedures, potential risks and benefits, and confidentiality of personal information.

Name of person obtaining consent:______________________________________

Signature of person obtaining consent: ____________________**Date:**____________

ANNEX A-3.

Informed Consent Form for Service Providers

English

Informed Consent Form for Service Providers

Introduction

You are invited to take part in a research study. Before you decide whether to participate, you need to understand why the research is being done and what it will involve. Please take the time to read or to listen as I read the following information. You may talk to others about the study if you wish. Please ask me if there is anything that is not clear, or if you would like more information. When all of your questions have been answered and you feel that you understand this study, you will be asked if you wish to participate in the study, and if yes, to sign this Informed Consent form. You will be given a signed copy to keep, if you would like one.

Purpose of the Study and Study Requirements

What is the study? The study is being conducted by a team of researchers from Muhimbili University of Health and Allied Sciences (MUHAS) in Dar es Salaam and the Futures Group, Population Council, and Pangaea Global AIDS Foundation in the United States. The overall purpose of the study is to measure the effect of a set of programs and services that will be delivered in health facilities and in the community to improve the health and well-being of women and children. These programs and services are intended to address gender-based violence (including physical, sexual, and emotional violence), which most typically takes the form of violence against women and children. The study will compare data from communities where these programs and services are being delivered with data from communities that aren't yet receiving these services to determine if the program had the desired effect.

The study has been funded by the United States Government.

Why have I been invited to take part? You have been invited to take part in this study because you have been identified as someone who may provide services to survivors of gender-based violence (GBV) at this health facility. The information you give us regarding your knowledge and experiences with the services you provide will assist the study team to understand how health services are being provided and what gaps and challenges may exist. The information you give us will help to strengthen services in Mbeya and other regions.

What will happen if I take part? If you agree to take part in the study, we will ask you to sign this form indicating that you agree to be part of the study. You will also be asked to answer some questions regarding your knowledge of GBV and the types of services for GBV survivors that are being provided at your health facility. An interviewer will sit with you and ask you a series of questions. The interviewer will record your responses on a form. The information that you provide will be kept completely confidential. Your responses will be combined with those of other participants in the study.

How long will the interview last? The interview will take between 60 and 90 minutes.

We may contact you again if we need to obtain further information or clarifications regarding the information you provide us with today.

Risks

What are the risks of the study?
If you decide to participate, you may be inconvenienced by the time and effort that it will take to complete the interview. You may find one or more questions that we ask to be sensitive. You do not have to respond to any question that makes you uncomfortable. You may end the interview at any time without penalty or loss of any benefits to which you are entitled.

Breach of confidentiality (that is, something you say in confidence during the interview that then accidentally becomes known to others) is a potential risk of your participation in the study. We will take precautions, however, to see that this does not happen.

Benefits

What are the benefits of participating? There may be no direct benefit to you for participating in the study. However, you may find an indirect benefit in knowing you have participated in an important study that could help others in the future. You may also appreciate the opportunity to share your experiences in confidence with another person.

Confidentiality

Will my participation in the study be kept confidential? The information that is collected during this interview will not include your name or any other information to personally identify you. Only members of the study team will have access to your questionnaire and it will be kept in a locked cabinet at the MUHAS offices in Dar es Salaam. You will not be identified in any reports or publications regarding the study.

Voluntary Participation

What are my rights as a study participant? Your participation in this study is completely voluntary. If you decide not to participate, you will not lose any existing benefits to which you are entitled. If you agree to participate in this study, you may end your participation at any time without penalty or loss of existing benefits to which you are entitled. If you decide to take part, you are free to refrain from answering any questions. You are free to withdraw at any time without affecting your relationship with your employer or the community.

Additional Information
What will I receive for participating? You will not receive any monetary or other incentive for participation.

What will happen to the results of the research study? The results and progress of the study will be presented in research publications and at meetings and conferences in Tanzania and in other countries. The Ministry of Health and Social Welfare (MOHSW), Regional Medical Office (RMO), and community partners will use the results to make improvements to their programs and services. In addition, the study progress and results will be put on a website so that others can learn from this study and strengthen services in their countries.

Who has reviewed the study for ethical issues? This study has been reviewed by research Institutional Review Boards at the Population Council, MUHAS, Tanzanian National Institute for Medical Research (NIMR), and Mbeya Research and Ethics Committee.

What if I need more information? If you have a concern about any aspect of the study, you should ask to speak to the researchers, who will do their best to answer your questions and address your concerns. You can contact Dr. Jessie Mbwambo, a psychiatrist at MUHAS and a principal investigator of the study, telephone number: +255 784 339 747; or Ms. Stella Mujaya, Study Coordinator, at the Futures Group, telephone number: +255 754 307 205.

What if there is a problem? For any concern you may have about the way you have been treated during the study or any possible harm you might suffer, please contact: Professor Mainen Moshi, Director of Research and Publications, MUHAS, Phone number: +255 222 150 302/6.

Subject Statement: I have read or listened to the Informed Consent for this study. I have received an explanation of the planned research, procedures, risks and benefits and privacy of my personal information. I agree to take part in this study. I understand that my participation in this study is voluntary.

Your name: ____

Your signature: ____**Date:**___________

Investigator or person who conducted Informed Consent discussion:
I confirm that I have personally explained the nature and extent of the planned research, study procedures, potential risks and benefits and confidentiality of personal information.

Name of person obtaining consent:______________________________________

Signature of person obtaining consent: ____________________**Date:**___________

ANNEX B-1a.

Household Survey Questionnaire

Baseline

English

ID: [ ][ ][ ][ ][ ][ ][ ]

**HOUSEHOLD IDENTIFICATION**
STUDY CLUSTER NAME ___________________________ DISTRICT NAME ___________________ CODE: [ ] [ ]
ENUMERATION AREA (EA) NUMBER: [ ] [ ] [ ] [ ] [ ] [ ] [ ] [ ] [ ] [ ] [ ] [ ]
NAME OF WARD ____ CODE: [ ] [ ] [ ]
VILLAGE/HAMLET/ STREET ________________________________ HOUSE NUMBER (if available) _____________
OTHER INFO TO IDENTIFY HOUSEHOLD (if needed) ____

INTERVIEW PARTICULARS

	1st VISIT	2nd VISIT	3rd VISIT	FINAL VISIT
DATE (DD/MM/YYYY)	________________	________________	________________	DATE _ _ /_ _ / _ _ _ _ DD/ MM/ YYYY
INTERVIEWERS ID	________________	________________	________________	
RESULT*	[ ] [ ]	[ ] [ ]	[ ] [ ]	RESULT* [ ] [ ]
NEXT VISIT, IF SCHEDULED Date:	________________	________________		Total Number of visits: [ ]
Time:	________________	________________		

* RESULT CODES:<br>01. Interview completed<br>02. No competent adult in household at time of visit<br>03. No eligible females living in household<br>04. Entire household absent for extended period of time<br>05. Refused by household head/representative<br>06. Selected participant not present; rescheduled<br>07. Selected participant not present; could not reschedule<br>08. Refused by selected female<br>09. Refused by parent<br>10. Other specify: _____________________	TOTAL # PERSONS IN HOUSEHOLD [ ][ ]<br><br>TOTAL # ELIGIBLE FEMALES AGED 15-49 [ ]<br><br>LINE # OF SELECTED ELIGIBLE FEMALE [ ]

STUDY CONSENT SUMMARY

For selected respondents ages 18-49, ADULT INFORMED CONSENT obtained?	YES [ ] NO [ ]
For selected respondents ages 15-17, PARENTAL CONSENT obtained?	YES [ ] NO [ ]
MINOR ASSENT obtained?	YES [ ] NO [ ]

QUESTIONNAIRE REVIEW AND PROCESSING

TEAM LEADER SIGN OFF	QUESTIONNAIRE CHECKED BY	OFFICE EDITOR	ENTERED BY
NAME ________________<br>DATE _ _/_ _/ _ _ _ _<br>dd /mm/yyyy	NAME<br>___________________<br>DATE _ _/_ _/ _ _ _ _<br>dd /mm/yyyy	NAME<br>________________<br>DATE _ _/_ _/ _ _ _ _<br>dd /mm/yyyy	ENTRY 1: ________<br>ENTRY 2: ________

ELIGIBLE FEMALES IN THE HOUSEHOLD		
INTERVIEWER OR TEAM LEADER INSTRUCTIONS:<br><br>For purposes of this study, a household is defined as: A group of individuals who usually live and eat together, whether or not they are related by blood or marriage, with one person, male or female, acknowledged as the head of the household. A household can consist of one person or many persons.<br><br>Several households may reside in one dwelling. If this is the case, randomly select one of the households (writing household labels on pieces of paper, then randomly drawing from a bag).<br><br>Upon reaching the house, ask to speak to the household head. If s/he is not available, ask to speak to any (competent) adult who resides in the household. If such individual is not there, ask for a time when s/he will return.<br><br>Then proceed with <u>Brief Introduction of the Study</u>. Then ask the following questions of the household head/adult.		
1.	Please can you tell me how many people live here, and share food regularly?	TOTAL NUMBER OF<br>PEOPLE IN HOUSEHOLD [ ][ ]
2.	Is the head of the household male or female?	MALE ....1<br>FEMALE 2
3.	What is your relationship to the head of this household?	[ ] [ ] CIRCLE CODE BELOW AND ENTER HERE

CODES

01 HEAD		
02 WIFE (PARTNER)	11 HUSBAND(PARTNER)	20 OTHER RELATIVE, SPECIFY: ____________________________
03 DAUGHTER	12 SON	
04 DAUGHTER IN LAW	13 SON IN LAW	
05 GRANDDAUGHTER	14 GRAND SON	21 OTHER NON RELATIVE, SPECIFY: ____________________________
06 MOTHER	15 FATHER	
07 MOTHER IN LAW	16 FATHER IN LAW	
08 SISTER	17 BROTHER	
09 SISTER IN LAW	18 BROTHER IN LAW	
10 ADOPTED/FOSTER/STEP DAUGHTER	19 ADOPTED/FOSTER/STEP SON	

	LIST OF FEMALE HOUSEHOLD MEMBERS AGES 15-49 WHO HAVE LIVED IN HH FOR PAST 6 MONTHS	RELATIONSHIP TO HEAD OF HH	AGE
4.	Today I would like to talk to one girl or woman from your household. To enable me to identify with whom I should talk, would you please give me the first names of:<br>• all girls and women ages 15-49 who usually live in your household (and share food) AND<br>• who have lived in the household for the past 6 months	What is the relationship of NAME to the head of the household?<br><br>USE CODES BELOW	How old is NAME?
INTEVIEWER INSTRUCTIONS: List eligible females in the order given to you.			
**LINE #**	**NAME**	**CODE**	**AGE (YEARS)**
**1**			
**2**			
**3**			
**4**			
**5**			
**6**			
**7**			
**8**			

CODES

01 HEAD	07 MOTHER IN LAW
02 WIFE (PARTNER)	08 SISTER
03 DAUGHTER	09 SISTER IN LAW
04 DAUGHTER IN LAW	22 ADOPTED/FOSTER/STEP DAUGHTER
05 GRANDDAUGHTER	23 OTHER RELATIVE, SPECIFY:____________________________
06 MOTHER	10 OTHER NON RELATIVE, SPECIFY:________________________

RANDOM SELECTION OF PARTICIPANT

INTERVIEWER INSTRUCTIONS:

- Identify the last digit of the **ID Number**. Go to this **row** in the table below.
- Obtain the total **number of eligible women** from the listing above. Go to this column number.
- Follow the selected row and column to the cell where they meet and **circle the number** in the cell. This is the line number of the female to be selected for the interview (from table above).
- Write the name and line number of the selected female in the space below the table.

LAST DIGIT OF THE ID NUMBER	TOTAL NUMBER OF ELIGIBLE WOMEN IN HOUSEHOLD							
	**1**	**2**	**3**	**4**	**5**	**6**	**7**	**8**
**0**	1	2	2	4	3	6	5	4
**1**	1	1	3	1	4	1	6	5
**2**	1	2	1	2	5	2	7	6
**3**	1	1	2	3	1	3	1	7
**4**	1	2	3	4	2	4	2	8
**5**	1	1	1	1	3	5	3	1
**6**	1	2	2	2	4	6	4	2
**7**	1	1	3	3	5	1	5	3
**8**	1	2	1	4	1	2	6	4
**9**	1	1	2	1	2	3	7	5

NAME OF SELECTED WOMAN_____________________ LINE NUMBER OF SELECTED WOMAN [ ]

AGE VERIFICATION

Interviewer instructions:
Ask to speak with the selected girl/woman. Ask her age and date of birth and record:

Reported age: _ _ Date of birth: _ _/_ _/_ _ _ _ Calculated age: _ _

- If reported or calculated age is 20 or younger:

– Ask to see written documentation. This could include birth certificate, clinic card, passport, religion card, ID card, or other official document.

– If documentation indicates she is aged of 15-17, continue with Parental Consent process.
If documentation indicates she is aged 18+, continue with Adult Informed Consent.

– If no documentation is available to verify age, then remove the name of this person from the list of eligible female members and move up all other names by one row. Repeat the random selection process, and then age verification, as indicated by reported age.

[ ] Tick here if you had to repeat the random selection because of no age documents

- If reported or calculated age is age 21-49, continue with the Adult Informed Consent process.

If the selected girl/woman is not available, reschedule a visit. If her age on the roster of eligible females is 20 or younger, before you leave attempt to verify her age through written documentation with others in the household.

RESPONDENT QUESTIONNAIRE

Introduction

INTERVIEWER: As you have just learned, the overall purpose of this study is to measure the effect of a set of programs and services delivered in health facilities and in the community to improve the health and well-being of women and children. I am going to ask you a series of questions and then will record your answers on this form. The interview will take from one to one and one half hours. As we noted in the consent form, you may find some questions that I will ask to be sensitive. You do not have to respond to any question that makes you uncomfortable. You may end the interview at any time. We will only begin the interview once we have ensured we are in a private place and you are comfortable. If anyone enters the room, we will pause the interview and look for another private space, if necessary, to ensure your comfort in moving forward with the interview.

RECORD START TIME OF INTERVIEW: [ ] [ ]:[ ] [ ] (use 24 hour clock)

Section 1 Respondent's background and her children

QUESTIONS and FILTERS		CODING CATEGORIES	SKIP TO
INTERVIEWER: If you don't mind, I'd like to start by asking you about and you, your family, and your household.			
➢ Household characteristics			
001	What is the main source of drinking water for members of your household?	PIPED WATER . . . . 1<br>*PIPED INTO DWELLING, PIPED INTO YARD/PLOT, PUBLIC TAP, NEIGHBOUR'S TAP*<br>WATER FROM OPEN WELL. . . . . .2<br>*OPEN WELL IN DWELLING, OPEN WELL IN YARD/PLOT, OPEN PUBLIC WELL, NEIGHBOUR'S OPEN WELL*<br>WATER FROM COVERED WELL OR BOREHOLE . . . . 3<br>*PROTECTED WELL IN DWELLING, PROTECTED WELL IN YARD/PLOT, PROTECTED PUBLIC WELL, NEIGHBOUR'S BOREHOLE*<br>SURFACE WATER . . . . .4<br>*SPRING, RIVER/STREAM, POND/LAKE, DAM*<br>RAINWATER . . . . .5<br>TANKER TRUCK . . . . 6<br>WATER VENDOR . . . . 7<br>BOTTLED WATER .8<br>OTHER ________________________ 9	
002	What kind of toilet facility do members of your household usually use?	FLUSH/POUR FLUSH TO: PIPED SEWER SYSTEM, PIPED SEPTIC TANK, PIPED PIT LATRINE, OR ELSEWHERE . . . . 1<br>PIT LATRINE 2	

QUESTIONS and FILTERS		CODING CATEGORIES	SKIP TO
		COMPOSTING TOILET/ECOSAN . . . . 3<br>BUCKET . . . . 4<br>NO FACILITY/BUSH/FIELD . . . . 5<br>OTHER . . . . .6<br>(SPECIFY) ____________________________	
003	Does your household have electricity?	YES . . . . 1<br>NO 2	
➢ Respondent's Age			
101	In which month and year were you born?	MONTH. . . . . [ ] [ ]<br>DON'T KNOW MONTH . . . . 98<br><br>YEAR . . . . [ ] [ ] [ ] [ ]<br>DON'T KNOW YEAR 9 9 9 8	
102	How old were you at your last birthday?<br><br>COMPARE AGE WITH Q101 AND CORRECT AGE IF NECESSARY	AGE IN COMPLETED YEARS. . . . . . . [ ] [ ]	
➢ Respondent's Literacy/Education			
103	Are you able to read and write?	YES . . . . 1<br>NO 2	
104	Have you ever attended school?	YES . . . . 1<br>NO 2	 ➢ 107
105	What is the highest level of school you attended?	PRE-PRIMARY/NURSERY . . . . 0<br>PRIMARY . . . . 1<br>POST-PRIMARY TRAINING . . . . 2<br>SECONDARY . . . . 3<br>POST-SECONDAY TRAINING . . . . 4<br>UNIVERSITY . . . . .5<br>DON'T KNOW 8	 <br><br><br><br><br>➢ 107
106	What is the highest grade/form/year that you completed at that level?<br><br>IF COMPLETED LESS THAN ONE YEAR AT THAT LEVEL, RECORD '00'	GRADE/FORM/YEAR. . . . . [ ] [ ]<br><br>DON'T KNOW. 98	
➢ Respondent's Work and Property			
107	As you know, some women take up jobs for which they are paid in cash or kind. Others sell things, have a small business or work on the family farm or in the family business. Aside from your own housework, have you done any of these things or any other work at any time during the past 12 months?	YES . . . . 1<br>NO 2	 ➢ 111

QUESTIONS and FILTERS		CODING CATEGORIES	SKIP TO
108	What is your occupation-- that is, what kind of work did you mainly do in the past 12 months?	a) RECORD IN WORDS WHAT R SAYS:<br><br>______________________________<br><br>b) CODE LATER WITH TEAM LEADER:<br>Professional/technical/managerial. . . . . . 1<br>Clerical. . . . . 2<br>Sales and services. . . . . 3<br>Skilled manual. . . . . 4<br>Unskilled manual .5<br>Domestic service. 6<br>Agriculture. . . . . 7<br>Student . . . . .8<br>Other 9	
109	Did you do this work throughout the year, or did you work seasonally, or only once in a while?	THROUGHOUT THE YEAR . . . . 1<br>SEASONALLY . . . . 2<br>ONCE IN A WHILE 3	
110	Were you paid in cash or in kind for this work, or were you not paid at all?<br><br>By "in kind" I mean when payment is made in goods, commodities or services -- instead of cash.	CASH ONLY . . . . 1<br>CASH AND IN KIND . . . . 2<br>IN KIND ONLY . . . . .3<br>NOT PAID 4	
111	Do you own this or any other house either alone or jointly with someone else?	OWNS ALONE . . . . 1<br>OWNS JOINTLY . . . . 2<br>DOES NOT OWN 3	
112	Do you own any land either alone or jointly with someone else?	OWNS ALONE . . . . 1<br>OWNS JOINTLY . . . . 2<br>DOES NOT OWN 3	
➢ Births and Children			
INTERVIEWER: Now I would like to ask about the births you have had during your life.			
113	a) Are you currently pregnant or have you been pregnant in the past 12 months?	YES. . . . . 1<br>NO . . . . .2<br>DON'T KNOW 8	
	b) Have you ever given birth?	YES . . . . 1<br>NO 2	➢ 120
114	Do you have any sons or daughters to whom you have given birth who are now living with you?	YES . . . . 1<br>NO 2	➢ 116
115	How many sons live with you?<br>And how many daughters live with you?<br><br>IF NONE, RECORD '00'.	a) SONS AT HOME . . . . [ ][ ]<br>b) DAUGHTERS AT HOME . . . . [ ][ ]	

QUESTIONS and FILTERS		CODING CATEGORIES	SKIP TO
116	Do you have any sons or daughters to whom you have given birth who are alive but do not live with you?	YES . . . . 1<br>NO 2	 ➢ 118
117	How many sons are alive but do not live with you?<br>And how many daughters are alive but do not live with you?<br>IF NONE, RECORD '00'.	a) SONS ELSEWHERE. . . . . . . [ ][ ]<br>b) DAUGHTERS ELSEWHERE. . [ ][ ]	
118	Have you ever given birth to a boy or girl who was born alive but later died?	YES . . . . 1<br>NO 2	 ➢ 120
119	How many children have died?	NUMBER OF CHILDREN WHO HAVE DIED . . . . [ ] [ ]<br>DON'T KNOW. 98	
➢ Partner Status: Current and in past 12 months			
120	What is your current marital status?	Married. . . . . .1<br>Living with a man as if married . . . . . . 2<br>Widowed. . . . . .3<br>Separated. . . . . 4<br>Divorced. . . . . 5<br>Never married/never lived with a man. 6	➢ 124<br>➢ 124<br><br><br><br>➢ 122
121	<u>At any time in the past 12 months</u>, were you married or living together with a man as if married?	Yes, married or living with a man as if married . . . . 1<br>No, not married or living with a man 2	
122	Do you currently have a steady boyfriend even if you did not live together or have sex?	YES . . . . 1<br>NO 2	➢ 124
123	<u>At any time in the past 12 months</u>, have you had a steady boyfriend even if you did not live together or have sex?	Yes, had boyfriend in past 12 mos. . . . . 1<br>No, did not have boyfriend in past 12 mos. . . . 2	
**SUMMARY**<br>**Interviewer instructions: Review responses to Q120-123 and code the following 2 variables. These variables will be used at the start of Section 3.**			
**124**	**Partner in past 12 months?**	YES. . . . . 1<br><u>If ANY of the following conditions are met</u>:<br>Q120 = 1 or 2<br>Q121=1<br>Q122=1<br>Q123=1<br>NO . . . . 2<br><u>If NONE of the conditions above are met</u>	
**125**	**Currently have a partner?**	YES. . . . . 1<br><u>If ANY of the following conditions are met</u>:<br>Q120 = 1 or 2<br>Q122=1	

QUESTIONS and FILTERS		CODING CATEGORIES	SKIP TO
		NO . . . . 2<br>If NONE of the conditions above are met	

Section 2 Respondent's health, health behaviors, and sexual history

INTERVIEWER: Now I would like to ask you some questions about your health and some other important aspects of a woman's life relating to health.

QUESTIONS and FILTERS		CODING CATEGORIES			SKIP TO
➢ Health and use of health services					
201	In general, would you describe your overall health as excellent, good, fair, poor, or very poor?	EXCELLENT. . . . . 1<br>GOOD. . . . . 2<br>FAIR. . . . . 3<br>POOR. . . . . 4<br>VERY POOR. 5			
202	In the past 12 months, did you have, or feel, the need for health services for yourself?	YES. . . . . 1<br>NO. 2			
203	In the past 12 months, did you visit a dispensary, health center, or hospital to obtain health services for yourself?	YES. . . . . 1<br>NO. 2			➢ 205
204	In the past 12 months, did a health care provider ever ask you about your experiences with violence?	YES. . . . . 1<br>NO. . . . . 2<br>DON'T KNOW/DON'T REMEMBER. 8			
205	What types of HIV services are available to women in your community?<br><br>READ EACH RESPONSE BELOW AND MARK RESPONSE.	**Available**	**Not available**	**Don't Know**	
	a. HIV testing and counseling for herself	1	2	8	
	b. HIV counseling and testing for couples (i.e., with her partner)	1	2	8	
	c. PMTCT	1	2	8	
	d. Antiretroviral treatment	1	2	8	
	e. Other clinical services such as medical treatment for opportunistic infections or other conditions that result from having AIDS	1	2	8	
	f. HIV post-exposure prophylaxis (PEP)	1	2	8	
	g. Education/prevention information	1	2	8	
	h. Psychosocial support/support group	1	2	8	

QUESTIONS and FILTERS		CODING CATEGORIES	SKIP TO
206	I don't want to know the results, but have you ever been tested to see if you have the AIDS virus?	YES . . . . 1<br>NO . . . . 2<br>DON'T KNOW. 8	 ➢ 209<br>➢ 209
207	When was the last time you were tested?	Within the past 12 MONTHS . . . . 1<br>13 - 24 MONTHS AGO. . . . . 2<br>MORE 2 YEARS AGO. . . . . 3<br>Don't know 8	
208	Again, I don't want to know the results, but did you get the results of the test?	YES . . . . 1<br>NO . . . . 2<br>DON'T KNOW. 8	
INTERVIEWER: Now I am going to ask you some questions that you may find to be sensitive. Please remember that everything you tell me today will be kept confidential and you can skip any question you are not comfortable answering.			
➢ Alcohol and substance use			
209	In the past 12 months, did you ever drink alcohol?	YES . . . . 1<br>NO 2	 ➢ 212
210	How often did you drink to the point of being drunk: often, sometimes, only once, or never?<br><br>By drunk, I mean feeling high, or tipsy, or losing control of your senses.	OFTEN . . . . .1<br>SOMETIMES . . . . 2<br>ONCE IN PAST 12 MOS. . . . . 3<br>NEVER . . . . .4<br>DON'T KNOW. 8	
211	With whom did you usually drink?<br><br>[RECORD ALL THAT THE RESPONDENT MENTIONS; DO NOT READ]	[ ] a. ALONE<br>[ ] b. WITH HUSBAND/PARTNER<br>[ ] c. WITH BOYFRIEND<br>[ ] d. WITH IN-LAWS<br>[ ] e. WITH OWN FAMILY MEMBERS<br>[ ] f. WITH FEMALE FRIENDS<br>[ ] g. WITH MALE FRIENDS<br>[ ] h. OTHER: SPECIFY ___________________	
212	In the past 12 months, did you ever smoke or otherwise use marijuana?	YES . . . . 1<br>NO 2	 ➢ 215
213	How often did you smoke or use marijuana to the point of feeling high: often, sometimes, only once or never?	OFTEN . . . . .1<br>SOMETIMES . . . . 2<br>ONCE IN PAST 12 MOS. . . . . 3<br>NEVER . . . . .4<br>DON'T KNOW. 8	
214	With whom did you usually smoke or otherwise use marijuana?<br><br>[RECORD ALL THAT THE RESPONDENT MENTIONS; DO NOT READ]	[ ] a. ALONE<br>[ ] b. WITH HUSBAND/PARTNER<br>[ ] c. WITH BOYFRIEND<br>[ ] d. WITH IN-LAWS<br>[ ] e. WITH OWN FAMILY MEMBERS<br>[ ] f. WITH FEMALE FRIENDS<br>[ ] g. WITH MALE FRIENDS<br>[ ] h. OTHER: SPECIFY ___________________	
215	At any time in your life, as a child or as an adult, has anyone ever forced you in any way to have sexual intercourse or perform any other sexual acts?	YES . . . . 1<br>NO . . . . 2<br>DID NOT ANSWER. 9	

QUESTIONS and FILTERS		CODING CATEGORIES	SKIP TO
216	How old were you when you had sexual intercourse for the very first time?	AGE IN YEARS . . . . [ ] [ ]<br>DON'T KNOW . . . . 98<br>Never had sexual intercourse. 00	➢ GO TO FILTER BELOW
217	The first time you had sexual intercourse, would you say that you had it because you wanted to, or because you were forced to have it against your will?	WANTED TO. . . . . 1<br>FORCED TO . . . . 2<br>DID NOT ANSWER. 9	
218	In total, with how many different people have you had sexual intercourse with in the last 12 months?	Total number of sexual partners in last 12 months [ ] [ ] [ ]<br>DON'T KNOW . . . . 998<br>DID NOT ANSWER. 999	

FILTER	
**INTERVIEWER FILTER:** INTIMATE PARTNER IN THE PAST 12 MONTHS? See Q124	
If Q124 = YES, CONTINUE WITH SECTION 3	IF Q124=NO, GO TO SECTION 5

Section 3 Intimate partner characteristics

INSTRUCTIONS TO INTERVIEWER:

THE QUESTIONS BELOW SHOULD BE ASKED FOR UP TO 3 INTIMATE PARTNERS THAT RESPONDENT (R) HAS HAD IN THE PAST 12 MONTHS.

BEGIN BY ASKING ABOUT CURRENT PARTNER, IF SHE HAS ONE (Q125=YES), or MOST RECENT PARTNER IF SHE CURRENTLY HAS NO PARTNER. THE RESPONDENT MAY HAVE CONCURRENT PARTNERS, e.g., CURRENTLY MARRIED AND A STEADY BOYFRIEND. WE HAVEN'T ASKED ABOUT THIS PREVIOUSLY, BUT THE FOLLOWING QUESTIONS ALLOW FOR IT. IN THIS CASE, YOU CAN REFER TO A PRIMARY PARTNER. HOWEVER, THE ORDER IN WHICH THE PARTNERS ARE ASKED ABOUT, DOESN'T MATTER.

ASK QUESTIONS VERTICALLY FROM Q301 THROUGH Q424 (SECTION 4).
THEN FOLLOW INSTRUCTIONS IN 424, BEGINNING AGAIN WITH Q301 IF R HAS HAD MORE THAN ONE INTIMATE PARTNER IN THE PAST 12 MONTHS.

INTERVIEWER: Next, I would like to ask you some questions about your current (or recent) male partner. Again, please let me assure you that your answers will be kept completely confidential and will not be told to anyone. If we should come to any question that you don't want to answer, just let me know and we will go on to the next question. You told me previously, that you currently have a partner/had a partner in the past year…

QUESTIONS and FILTERS		a) CURRENT, MOST RECENT, OR PRIMARY PARTNER	b) OTHER PARTNER (1)	c) OTHER PARTNER (2)
301	What is the relationship of this partner to you?	HUSBAND. . . . . 1<br>LIVE-IN PARTNER. . .2<br>EX-HUSBAND . . . . . .3<br>EX-LIVE IN PARTNER . . . . .4<br>STEADY BOYFRIEND (NOT LIVING WITH RESPONDENT). . . . . 5<br>EX-STEADY BOYFRIEND 6	HUSBAND. . . . . 1<br>LIVE-IN PARTNER. . .2<br>EX-HUSBAND . . . . . .3<br>EX-LIVE IN PARTNER . . . . .4<br>STEADY BOYFRIEND (NOT LIVING WITH RESPONDENT). . . . . 5<br>EX-STEADY BOYFRIEND 6	HUSBAND. . . . . 1<br>LIVE-IN PARTNER. . .2<br>EX-HUSBAND . . . . . 3<br>EX-LIVE IN PARTNER . . . . .4<br>STEADY BOYFRIEND (NOT LIVING WITH RESPONDENT). . . . . 5<br>EX-STEADY BOYFRIEND 6
302	Approximately how old is/was this partner compared to you?	YOUNGER THAN YOU ARE . . . . 1<br>SAME AGE AS YOU ARE . . . . .2<br>1-9 YEARS OLDER . . . 3<br>10+ YEARS OLDER. . . 4<br><br>DON'T KNOW 8	YOUNGER THAN YOU ARE . . . . 1<br>SAME AGE AS YOU ARE . . . . .2<br>1-9 YEARS OLDER . . . 3<br>10+ YEARS OLDER. . . 4<br><br>DON'T KNOW 8	YOUNGER THAN YOU ARE . . . . 1<br>SAME AGE AS YOU ARE . . . . .2<br>1-9 YEARS OLDER . . . 3<br>10+ YEARS OLDER. . . 4<br><br>DON'T KNOW 8
303	Did he ever attend school?	YES . . . . 1<br>NO . . . . 2 / DON'T KNOW. 8 — Go to 306	YES . . . . 1<br>NO . . . . 2 / DON'T KNOW. 8 — Go to 306	YES . . . . 1<br>NO . . . . 2 / DON'T KNOW. 8 — Go to 306
304	What is the highest level of school he attended?	PRE-PRIMARY/ NURSERY . . . . 0<br>PRIMARY . . . . 1<br>POST-PRIMARY TRAINING . . . . 2<br>SECONDARY . . . . . . 3<br>POST-SECONDAY TRAINING . . . . .4<br>UNIVERSITY . . . . 5<br>DON'T KNOW . . . . . . 8<br>[GO TO 306]	PRE-PRIMARY/ NURSERY . . . . 0<br>PRIMARY . . . . 1<br>POST-PRIMARY TRAINING . . . . 2<br>SECONDARY . . . . . . 3<br>POST-SECONDAY TRAINING . . . . .4<br>UNIVERSITY . . . . 5<br>DON'T KNOW . . . . . . 8<br>[GO TO 306]	PRE-PRIMARY/ NURSERY . . . . 0<br>PRIMARY . . . . 1<br>POST-PRIMARY TRAINING . . . . 2<br>SECONDARY . . . . . . 3<br>POST-SECONDAY TRAINING . . . . .4<br>UNIVERSITY . . . . 5<br>DON'T KNOW . . . . . . 8<br>[GO TO 306]
305	What is the highest grade/form/year he completed at that level?<br><br>IF COMPLETED LESS THAN ONE YEAR AT THAT LEVEL, RECORD '00'	GRADE/FORM/YEAR . . . . [ ] [ ]<br><br>DON'T KNOW 98	GRADE/FORM/YEAR . . . . [ ] [ ]<br><br>DON'T KNOW 98	GRADE/FORM/YEAR . . . . [ ] [ ]<br><br>DON'T KNOW 98
306	What is/was his occupation? That is, what kind of work does/did he mainly do?	1) RECORD IN WORDS WHAT R SAYS:<br><br>______________________	1) RECORD IN WORDS WHAT R SAYS:<br><br>______________________	1) RECORD IN WORDS WHAT R SAYS:<br><br>______________________

	QUESTIONS and FILTERS	a) CURRENT, MOST RECENT, OR PRIMARY PARTNER	b) OTHER PARTNER (1)	c) OTHER PARTNER (2)
		2) CODE WITH TEAM LEADER:<br>NOT WORKING . . . . .0<br>Professional/technical/ managerial. . . . . . 1<br>Clerical. . . . . .2<br>Sales and services . . . 3<br>Skilled manual. . . . . 4<br>Unskilled manual . . . . 5<br>Domestic service. . . . 6<br>Agriculture. . . . . 7<br>Student . . . . 8<br>R doesn't know 9	2) CODE WITH TEAM LEADER:<br>NOT WORKING . . . . .0<br>Professional/technical/ managerial. . . . . . 1<br>Clerical. . . . . .2<br>Sales and services . . . 3<br>Skilled manual. . . . . 4<br>Unskilled manual . . . . 5<br>Domestic service. . . . 6<br>Agriculture. . . . . 7<br>Student . . . . 8<br>R doesn't know 9	2) CODE WITH TEAM LEADER:<br>NOT WORKING . . . . .0<br>Professional/technical/ managerial. . . . . . 1<br>Clerical. . . . . .2<br>Sales and services . . . 3<br>Skilled manual. . . . . 4<br>Unskilled manual . . . . 5<br>Domestic service. . . . 6<br>Agriculture. . . . . 7<br>Student . . . . 8<br>R doesn't know 9
307	Does/did he have any other partners, a wife or wives, or does/did he live with another woman/other women as if married?	YES . . . . 1<br>NO . . . . 2<br>DON'T KNOW 8	YES . . . . 1<br>NO . . . . 2<br>DON'T KNOW 8	YES . . . . 1<br>NO . . . . 2<br>DON'T KNOW 8
308	In the past year, did your partner ever drink alcohol?	YES . . . . 1<br>NO. . . . . 2 / DON'T KNOW.8 — GO TO 311	YES . . . . 1<br>NO. . . . . 2 / DON'T KNOW.8 — GO TO 311	YES . . . . 1<br>NO. . . . . 2 / DON'T KNOW. 8 — GO TO 311
309	How often did he drink to the point of being drunk: often, sometimes, only once, or never?<br><br>By drunk, I mean feeling high, or tipsy, or losing control of your senses.	OFTEN . . . . .1<br>SOMETIMES . . . . 2<br>ONCE in past 12 mos. 3<br>NEVER . . . . .4<br><br>DON'T KNOW. 8	OFTEN . . . . .1<br>SOMETIMES . . . . 2<br>ONCE in past 12 mos. 3<br>NEVER . . . . .4<br><br>DON'T KNOW. 8	OFTEN . . . . .1<br>SOMETIMES . . . . 2<br>ONCE in past 12 mos. 3<br>NEVER . . . . .4<br><br>DON'T KNOW. 8
310	With whom did he usually drink?<br><br>[RECORD ALL THAT THE RESPONDENT MENTIONS; DO NOT READ]	[ ] a. ALONE<br>[ ] b. WITH RESPONDENT<br>[ ] c. WITH GIRLFRIEND<br>[ ] d. WITH IN-LAWS<br>[ ] e. WITH OWN FAMILY MEMBERS<br>[ ] f. WITH FEMALE FRIENDS<br>[ ] g. WITH MALE FRIENDS<br>[ ] h. OTHER: SPECIFY<br>___________________	[ ] a. ALONE<br>[ ] b. WITH RESPONDENT<br>[ ] c. WITH GIRLFRIEND<br>[ ] d. WITH IN-LAWS<br>[ ] e. WITH OWN FAMILY MEMBERS<br>[ ] f. WITH FEMALE FRIENDS<br>[ ] g. WITH MALE FRIENDS<br>[ ] h. OTHER: SPECIFY<br>___________________	[ ] a. ALONE<br>[ ] b. WITH RESPONDENT<br>[ ] c. WITH GIRLFRIEND<br>[ ] d. WITH IN-LAWS<br>[ ] e. WITH OWN FAMILY MEMBERS<br>[ ] f. WITH FEMALE FRIENDS<br>[ ] g. WITH MALE FRIENDS<br>[ ] h. OTHER: SPECIFY<br>___________________
311	In the past 12 months, did your partner ever smoke or otherwise use marijuana?	YES . . . . 1<br>NO. . . . . 2 / DON'T KNOW.8 — GO TO	YES . . . . 1<br>NO. . . . . 2 / DON'T KNOW.8 — GO TO	YES . . . . 1<br>NO. . . . . 2 / DON'T KNOW. 8 — GO TO

QUESTIONS and FILTERS		a) CURRENT, MOST RECENT, OR PRIMARY PARTNER		b) OTHER PARTNER (1)		c) OTHER PARTNER (2)	
			314		314		314
312	How often did he smoke or use marijuana to the point of feeling high: often, sometimes, only once or never?	OFTEN . . . . .1<br>SOMETIMES . . . . 2<br>ONCE . . . . 3<br>NEVER . . . . 4<br>DON'T KNOW. 8		OFTEN . . . . .1<br>SOMETIMES . . . . 2<br>ONCE . . . . 3<br>NEVER . . . . 4<br>DON'T KNOW. 8		OFTEN . . . . .1<br>SOMETIMES . . . . 2<br>ONCE . . . . 3<br>NEVER . . . . 4<br>DON'T KNOW. 8	
313	With whom did he usually smoke or otherwise use marijuana?<br><br>[RECORD ALL THAT THE RESPONDENT MENTIONS; DO NOT READ]	[ ] a. ALONE<br>[ ] b. WITH RESPONDENT<br>[ ] c. WITH GIRLFRIEND<br>[ ] d. WITH IN-LAWS<br>[ ] e. WITH OWN FAMILY MEMBERS<br>[ ] f. WITH FEMALE FRIENDS<br>[ ] g. WITH MALE FRIENDS<br>[ ] h. OTHER: SPECIFY<br>___________________		[ ] a. ALONE<br>[ ] b. WITH RESPONDENT<br>[ ] c. WITH GIRLFRIEND<br>[ ] d. WITH IN-LAWS<br>[ ] e. WITH OWN FAMILY MEMBERS<br>[ ] f. WITH FEMALE FRIENDS<br>[ ] g. WITH MALE FRIENDS<br>[ ] h. OTHER: SPECIFY<br>___________________		[ ] a. ALONE<br>[ ] b. WITH RESPONDENT<br>[ ] c. WITH GIRLFRIEND<br>[ ] d. WITH IN-LAWS<br>[ ] e. WITH OWN FAMILY MEMBERS<br>[ ] f. WITH FEMALE FRIENDS<br>[ ] g. WITH MALE FRIENDS<br>[ ] h. OTHER: SPECIFY<br>___________________	
314	Have you had sexual intercourse with this partner?	YES, in past 12 mos . . . . 1<br><br>Yes, but more than 12 mos ago . . . . 2<br>GO TO SECTION 4<br><br>No, never had sex with this partner. . . . . 3<br>GO TO Section 4		YES, in past 12 mos . . . . 1<br><br>Yes, but more than 12 mos ago . . . . 2<br>GO TO Section 4<br><br>No, never had sex with this partner. . . . . 3<br>GO TO Section 4		YES, in past 12 mos . . . . 1<br><br>Yes, but more than 12 mos ago . . . . 2<br>GO TO Section 4<br><br>No, never had sex with this partner. . . . . 3<br>GO TO Section 4	
315	Approximately how often did you have sexual intercourse with this person <u>in the last 12 months</u>?<br><br>READ CATEGORIES	OFTEN<br>(2-3 times/week) . . . .1<br><br>SOMETIMES<br>(few times/mo). . . . . 2<br><br>RARELY. . . . . .3<br>ONCE. . . . . 4<br>DON'T KNOW. 8		OFTEN<br>(2-3 times/week) . . . .1<br><br>SOMETIMES<br>(few times/mo). . . . . 2<br><br>RARELY. . . . . .3<br>ONCE. . . . . 4<br>DON'T KNOW. 8		OFTEN<br>(2-3 times/week) . . . .1<br><br>SOMETIMES<br>(few times/mo). . . . . 2<br><br>RARELY. . . . . .3<br>ONCE. . . . . 4<br>DON'T KNOW. 8	
316	The last time you had intercourse with this person, was a condom used?	YES. 1		YES. 1		YES. 1	
		NO. . . . . . . 2<br>DON'T KNOW . . . 8	GO TO SECTION 4	NO. . . . . . . 2<br>DON'T KNOW . . . 8	GO TO SECTION 4	NO. . . . . . . 2<br>DON'T KNOW . . . 8	GO TO SECTION 4
317	<u>In the past 12 months</u>, did you use a condom every time you had sexual intercourse with this person?	YES. . . . . 1<br>NO. . . . . 2<br>DON'T KNOW 8		YES. . . . . 1<br>NO. . . . . 2<br>DON'T KNOW 8		YES. . . . . 1<br>NO. . . . . 2<br>DON'T KNOW 8	

Section 4 IPV in the past 12 months

INTERVIEWER: Read this only the first time/for first partner. I am now going to ask you about some situations that happen to some women. I'd like you to think back over the past 12 months and please tell me if they happened to you. You may find some of these questions to be sensitive. Please remember if we should come to any question that you do not want to answer, just let me know and we will go on to the next question. I want to remind you that if you prefer, we can stop the interview at any time.

QUESTIONS and FILTERS		a) CURRENT, MOST RECENT, OR PRIMARY PARTNER	b) OTHER PARTNER (1)	c) OTHER PARTNER (2)
**INTERVIEWER**: So continuing with the partner we were just discussing, please let me know how often, if at all, this happened to you in the past year.				
➢ Controlling partner behavior				
401	In the last 12 months, was your partner jealous or angry if you talked to other men—often, sometimes, just once, or not at all?	OFTEN. . . . . 1<br>SOMETIMES. . . . . . . 2<br>ONCE. . . . . 3<br>NEVER. . . . . 4<br>DON'T KNOW . . . . . 8<br>NO ANSWER 9	OFTEN. . . . . 1<br>SOMETIMES. . . . . . . 2<br>ONCE. . . . . 3<br>NEVER. . . . . 4<br>DON'T KNOW . . . . . 8<br>NO ANSWER 9	OFTEN. . . . . 1<br>SOMETIMES. . . . . . . 2<br>ONCE. . . . . 3<br>NEVER. . . . . 4<br>DON'T KNOW . . . . . 8<br>NO ANSWER 9
402	Did he accuse you of being unfaithful?	OFTEN. . . . . 1<br>SOMETIMES. . . . . . . 2<br>ONCE. . . . . 3<br>NEVER. . . . . 4<br>DON'T KNOW . . . . . 8<br>NO ANSWER 9	OFTEN. . . . . 1<br>SOMETIMES. . . . . . . 2<br>ONCE. . . . . 3<br>NEVER. . . . . 4<br>DON'T KNOW . . . . . 8<br>NO ANSWER 9	OFTEN. . . . . 1<br>SOMETIMES. . . . . . . 2<br>ONCE. . . . . 3<br>NEVER. . . . . 4<br>DON'T KNOW . . . . . 8<br>NO ANSWER 9
403	Did he try to isolate you from meeting your female friends?	OFTEN. . . . . 1<br>SOMETIMES. . . . . . . 2<br>ONCE. . . . . 3<br>NEVER. . . . . 4<br>DON'T KNOW . . . . . 8<br>NO ANSWER 9	OFTEN. . . . . 1<br>SOMETIMES. . . . . . . 2<br>ONCE. . . . . 3<br>NEVER. . . . . 4<br>DON'T KNOW . . . . . 8<br>NO ANSWER 9	OFTEN. . . . . 1<br>SOMETIMES. . . . . . . 2<br>ONCE. . . . . 3<br>NEVER. . . . . 4<br>DON'T KNOW . . . . . 8<br>NO ANSWER 9
404	Did he try to limit your contact with your family?	OFTEN. . . . . 1<br>SOMETIMES. . . . . . . 2<br>ONCE. . . . . 3<br>NEVER. . . . . 4<br>DON'T KNOW . . . . . 8<br>NO ANSWER 9	OFTEN. . . . . 1<br>SOMETIMES. . . . . . . 2<br>ONCE. . . . . 3<br>NEVER. . . . . 4<br>DON'T KNOW . . . . . 8<br>NO ANSWER 9	OFTEN. . . . . 1<br>SOMETIMES. . . . . . . 2<br>ONCE. . . . . 3<br>NEVER. . . . . 4<br>DON'T KNOW . . . . . 8<br>NO ANSWER 9
405	Did he generally insist on knowing where you were at all times?	OFTEN. . . . . 1<br>SOMETIMES. . . . . . . 2<br>ONCE. . . . . 3<br>NEVER. . . . . 4<br>DON'T KNOW . . . . . 8<br>NO ANSWER 9	OFTEN. . . . . 1<br>SOMETIMES. . . . . . . 2<br>ONCE. . . . . 3<br>NEVER. . . . . 4<br>DON'T KNOW . . . . . 8<br>NO ANSWER 9	OFTEN. . . . . 1<br>SOMETIMES. . . . . . . 2<br>ONCE. . . . . 3<br>NEVER. . . . . 4<br>DON'T KNOW . . . . . 8<br>NO ANSWER 9
406	Did he try to control your use of money?	OFTEN. . . . . 1<br>SOMETIMES. . . . . . . 2<br>ONCE. . . . . 3<br>NEVER. . . . . 4<br>DON'T KNOW 8	OFTEN. . . . . 1<br>SOMETIMES. . . . . . . 2<br>ONCE. . . . . 3<br>NEVER. . . . . 4<br>DON'T KNOW 8	OFTEN. . . . . 1<br>SOMETIMES. . . . . . . 2<br>ONCE. . . . . 3<br>NEVER. . . . . 4<br>DON'T KNOW 8

QUESTIONS and FILTERS		a) CURRENT, MOST RECENT, OR PRIMARY PARTNER	b) OTHER PARTNER (1)	c) OTHER PARTNER (2)
		NO ANSWER 9	NO ANSWER 9	NO ANSWER 9
407	Would you say that the money you earned in the past 12 months was more than what your partner earned, less than what he earned, or about the same?	MORE THAN HE EARNED. . . . . 1<br>LESS THAN HE EARNED. . . . . 2<br>ABOUT THE SAME . . 3<br>DON'T KNOW 8	MORE THAN HE EARNED. . . . . 1<br>LESS THAN HE EARNED. . . . . 2<br>ABOUT THE SAME . . 3<br>DON'T KNOW 8	MORE THAN HE EARNED. . . . . 1<br>LESS THAN HE EARNED. . . . . 2<br>ABOUT THE SAME . . 3<br>DON'T KNOW 8
➢ Experience of Emotional violence				
408	In the last 12 months:<br>Did he say or do anything to humiliate you in front of others?	OFTEN. . . . . 1<br>SOMETIMES. . . . . . . 2<br>ONCE. . . . . 3<br>NEVER. . . . . 4<br>DON'T KNOW . . . . . 8<br>NO ANSWER 9	OFTEN. . . . . 1<br>SOMETIMES. . . . . . . 2<br>ONCE. . . . . 3<br>NEVER. . . . . 4<br>DON'T KNOW . . . . . 8<br>NO ANSWER 9	OFTEN. . . . . 1<br>SOMETIMES. . . . . . . 2<br>ONCE. . . . . 3<br>NEVER. . . . . 4<br>DON'T KNOW . . . . . 8<br>NO ANSWER 9
409	Did he threaten to hurt or harm you or someone close to you?	OFTEN. . . . . 1<br>SOMETIMES. . . . . . . 2<br>ONCE. . . . . 3<br>NEVER. . . . . 4<br>DON'T KNOW . . . . . 8<br>NO ANSWER 9	OFTEN. . . . . 1<br>SOMETIMES. . . . . . . 2<br>ONCE. . . . . 3<br>NEVER. . . . . 4<br>DON'T KNOW . . . . . 8<br>NO ANSWER 9	OFTEN. . . . . 1<br>SOMETIMES. . . . . . . 2<br>ONCE. . . . . 3<br>NEVER. . . . . 4<br>DON'T KNOW . . . . . 8<br>NO ANSWER 9
410	Did he insult you or make you feel bad about yourself?	OFTEN. . . . . 1<br>SOMETIMES. . . . . . . 2<br>ONCE. . . . . 3<br>NEVER. . . . . 4<br>DON'T KNOW . . . . . 8<br>NO ANSWER 9	OFTEN. . . . . 1<br>SOMETIMES. . . . . . . 2<br>ONCE. . . . . 3<br>NEVER. . . . . 4<br>DON'T KNOW . . . . . 8<br>NO ANSWER 9	OFTEN. . . . . 1<br>SOMETIMES. . . . . . . 2<br>ONCE. . . . . 3<br>NEVER. . . . . 4<br>DON'T KNOW . . . . . 8<br>NO ANSWER 9
➢ Experience of Physical violence				
411	Did he push you, shake you, or throw something at you?	OFTEN. . . . . 1<br>SOMETIMES. . . . . . . 2<br>ONCE. . . . . 3<br>NEVER. . . . . 4<br>DON'T KNOW . . . . . 8<br>NO ANSWER 9	OFTEN. . . . . 1<br>SOMETIMES. . . . . . . 2<br>ONCE. . . . . 3<br>NEVER. . . . . 4<br>DON'T KNOW . . . . . 8<br>NO ANSWER 9	OFTEN. . . . . 1<br>SOMETIMES. . . . . . . 2<br>ONCE. . . . . 3<br>NEVER. . . . . 4<br>DON'T KNOW . . . . . 8<br>NO ANSWER 9
412	Did he Slap you?	OFTEN. . . . . 1<br>SOMETIMES. . . . . . . 2<br>ONCE. . . . . 3<br>NEVER. . . . . 4<br>DON'T KNOW . . . . . 8<br>NO ANSWER 9	OFTEN. . . . . 1<br>SOMETIMES. . . . . . . 2<br>ONCE. . . . . 3<br>NEVER. . . . . 4<br>DON'T KNOW . . . . . 8<br>NO ANSWER 9	OFTEN. . . . . 1<br>SOMETIMES. . . . . . . 2<br>ONCE. . . . . 3<br>NEVER. . . . . 4<br>DON'T KNOW . . . . . 8<br>NO ANSWER 9

QUESTIONS and FILTERS		a) CURRENT, MOST RECENT, OR PRIMARY PARTNER	b) OTHER PARTNER (1)	c) OTHER PARTNER (2)
413	Did he Twist your arm or pull your hair?	OFTEN. . . . . 1<br>SOMETIMES. . . . . . . 2<br>ONCE. . . . . 3<br>NEVER. . . . . 4<br>DON'T KNOW . . . . . 8<br>NO ANSWER 9	OFTEN. . . . . 1<br>SOMETIMES. . . . . . . 2<br>ONCE. . . . . 3<br>NEVER. . . . . 4<br>DON'T KNOW . . . . . 8<br>NO ANSWER 9	OFTEN. . . . . 1<br>SOMETIMES. . . . . . . 2<br>ONCE. . . . . 3<br>NEVER. . . . . 4<br>DON'T KNOW . . . . . 8<br>NO ANSWER 9
414	Did he punch you with his fist or with something that could hurt you?	OFTEN. . . . . 1<br>SOMETIMES. . . . . . . 2<br>ONCE. . . . . 3<br>NEVER. . . . . 4<br>DON'T KNOW . . . . . 8<br>NO ANSWER 9	OFTEN. . . . . 1<br>SOMETIMES. . . . . . . 2<br>ONCE. . . . . 3<br>NEVER. . . . . 4<br>DON'T KNOW . . . . . 8<br>NO ANSWER 9	OFTEN. . . . . 1<br>SOMETIMES. . . . . . . 2<br>ONCE. . . . . 3<br>NEVER. . . . . 4<br>DON'T KNOW . . . . . 8<br>NO ANSWER 9
415	Did he kick you, drag you or beat you up?	OFTEN. . . . . 1<br>SOMETIMES. . . . . . . 2<br>ONCE. . . . . 3<br>NEVER. . . . . 4<br>DON'T KNOW . . . . . 8<br>NO ANSWER 9	OFTEN. . . . . 1<br>SOMETIMES. . . . . . . 2<br>ONCE. . . . . 3<br>NEVER. . . . . 4<br>DON'T KNOW . . . . . 8<br>NO ANSWER 9	OFTEN. . . . . 1<br>SOMETIMES. . . . . . . 2<br>ONCE. . . . . 3<br>NEVER. . . . . 4<br>DON'T KNOW . . . . . 8<br>NO ANSWER 9
416	Did he try to choke you or burn you on purpose?	OFTEN. . . . . 1<br>SOMETIMES. . . . . . . 2<br>ONCE. . . . . 3<br>NEVER. . . . . 4<br>DON'T KNOW . . . . . 8<br>NO ANSWER 9	OFTEN. . . . . 1<br>SOMETIMES. . . . . . . 2<br>ONCE. . . . . 3<br>NEVER. . . . . 4<br>DON'T KNOW . . . . . 8<br>NO ANSWER 9	OFTEN. . . . . 1<br>SOMETIMES. . . . . . . 2<br>ONCE. . . . . 3<br>NEVER. . . . . 4<br>DON'T KNOW . . . . . 8<br>NO ANSWER 9
417	Did he threaten or attack you with a knife, gun, or any other weapon?	OFTEN. . . . . 1<br>SOMETIMES. . . . . . . 2<br>ONCE. . . . . 3<br>NEVER. . . . . 4<br>DON'T KNOW . . . . . 8<br>NO ANSWER 9	OFTEN. . . . . 1<br>SOMETIMES. . . . . . . 2<br>ONCE. . . . . 3<br>NEVER. . . . . 4<br>DON'T KNOW . . . . . 8<br>NO ANSWER 9	OFTEN. . . . . 1<br>SOMETIMES. . . . . . . 2<br>ONCE. . . . . 3<br>NEVER. . . . . 4<br>DON'T KNOW . . . . . 8<br>NO ANSWER 9
➢ Experience of Physical sexual violence				
418	Did he physically force you to have sexual intercourse with him even when you did not want to?	OFTEN. . . . . 1<br>SOMETIMES. . . . . . . 2<br>ONCE. . . . . 3<br>NEVER. . . . . 4<br>DON'T KNOW . . . . . 8<br>NO ANSWER 9	OFTEN. . . . . 1<br>SOMETIMES. . . . . . . 2<br>ONCE. . . . . 3<br>NEVER. . . . . 4<br>DON'T KNOW . . . . . 8<br>NO ANSWER 9	OFTEN. . . . . 1<br>SOMETIMES. . . . . . . 2<br>ONCE. . . . . 3<br>NEVER. . . . . 4<br>DON'T KNOW . . . . . 8<br>NO ANSWER 9
419	Did he force you to do anything sexual that you did not want to?	OFTEN. . . . . 1<br>SOMETIMES. . . . . . . 2<br>ONCE. . . . . 3<br>NEVER. . . . . 4<br>DON'T KNOW . . . . . 8<br>NO ANSWER 9	OFTEN. . . . . 1<br>SOMETIMES. . . . . . . 2<br>ONCE. . . . . 3<br>NEVER. . . . . 4<br>DON'T KNOW . . . . . 8<br>NO ANSWER 9	OFTEN. . . . . 1<br>SOMETIMES. . . . . . . 2<br>ONCE. . . . . 3<br>NEVER. . . . . 4<br>DON'T KNOW . . . . . 8<br>NO ANSWER 9

QUESTIONS and FILTERS		a) CURRENT, MOST RECENT, OR PRIMARY PARTNER	b) OTHER PARTNER (1)	c) OTHER PARTNER (2)
➢ Experience of severe Physical violence				
**INTERVIEWER**: Did the following ever happen in the past 12 months as a result of what this partner did to you?				
420	You had cuts, bruises, or aches?	OFTEN. . . . . 1<br>SOMETIMES. . . . . . . 2<br>ONCE. . . . . 3<br>NEVER. . . . . 4<br>DON'T KNOW . . . . . 8<br>NO ANSWER 9	OFTEN. . . . . 1<br>SOMETIMES. . . . . . . 2<br>ONCE. . . . . 3<br>NEVER. . . . . 4<br>DON'T KNOW . . . . . 8<br>NO ANSWER 9	OFTEN. . . . . 1<br>SOMETIMES. . . . . . . 2<br>ONCE. . . . . 3<br>NEVER. . . . . 4<br>DON'T KNOW . . . . . 8<br>NO ANSWER 9
421	You had eye injuries, sprains, dislocations, or burns?	OFTEN. . . . . 1<br>SOMETIMES. . . . . . . 2<br>ONCE. . . . . 3<br>NEVER. . . . . 4<br>DON'T KNOW . . . . . 8<br>NO ANSWER 9	OFTEN. . . . . 1<br>SOMETIMES. . . . . . . 2<br>ONCE. . . . . 3<br>NEVER. . . . . 4<br>DON'T KNOW . . . . . 8<br>NO ANSWER 9	OFTEN. . . . . 1<br>SOMETIMES. . . . . . . 2<br>ONCE. . . . . 3<br>NEVER. . . . . 4<br>DON'T KNOW . . . . . 8<br>NO ANSWER 9
422	You had deep wounds, broken bones, broken teeth, or any other serious injury?	OFTEN. . . . . 1<br>SOMETIMES. . . . . . . 2<br>ONCE. . . . . 3<br>NEVER. . . . . 4<br>DON'T KNOW . . . . . 8<br>NO ANSWER 9	OFTEN. . . . . 1<br>SOMETIMES. . . . . . . 2<br>ONCE. . . . . 3<br>NEVER. . . . . 4<br>DON'T KNOW . . . . . 8<br>NO ANSWER 9	OFTEN. . . . . 1<br>SOMETIMES. . . . . . . 2<br>ONCE. . . . . 3<br>NEVER. . . . . 4<br>DON'T KNOW . . . . . 8<br>NO ANSWER 9
➢ Perpetration of Physical violence				
423	In the past 12 months, did you ever hit, slap, kick, or do anything else to physically hurt your partner at times when he was not already beating or physically hurting you?	OFTEN. . . . . 1<br>SOMETIMES. . . . . . . 2<br>ONCE. . . . . 3<br>NEVER. . . . . 4<br>DON'T KNOW . . . . . 8<br>NO ANSWER 9	OFTEN. . . . . 1<br>SOMETIMES. . . . . . . 2<br>ONCE. . . . . 3<br>NEVER. . . . . 4<br>DON'T KNOW . . . . . 8<br>NO ANSWER 9	OFTEN. . . . . 1<br>SOMETIMES. . . . . . . 2<br>ONCE. . . . . 3<br>NEVER. . . . . 4<br>DON'T KNOW . . . . . 8<br>NO ANSWER 9
**FILTER:** Other partners in past 12 months?				
424	Apart from (this person, these two people) we have just discussed, in the past 12 months did you have any other male partners (i.e., husband, live-in partner, ex-husband, ex-live in partner, boyfriend, ex-boyfriend)?	YES . . . . .1<br>GO BACK TO 301 and ask Q301 through Q424 for second partner; RECORD RESPONSES IN COLUMN 2<br><br>No 2	YES . . . . .1<br>GO BACK TO 301 and ask Q301 through Q424 for third partner; RECORD RESPONSES IN COLUMN 3<br><br>No 2	GO TO SECTION 5

Section 5 Physical and sexual violence from others in past 12 months

INTERVIEWER: I would now like to ask you about types of violence that you may have experienced from others, (IF SECTIONS 3 AND 4 WERE ASKED, THEN ADD: besides the partners we have just been discussing).

QUESTIONS and FILTERS		CODING CATEGORIES	SKIP TO
➢ Physical violence			
501	In the past 12 months, has anyone [other than the partner/partners we have just talked about (husband, live-in partner, steady boyfriend)] hit, slapped, kicked or done anything else to hurt you physically?	YES. . . . . 1<br>NO . . . . 2<br>NO ANSWER 9	 ➢ 503<br>➢ 503
502	Who has hurt you in this way? DO NOT READ CATEGORIES, BUT TICK ALL THAT R MENTIONS; USE ONLY ONE CATEGORY FOR EACH PERSON MENTIONED.<br>THEN ASK ABOUT FREQUENCY FOR THOSE TICKED:<br>Did this happen often, sometimes or only once in the past 12 months?		
	a. [ ] MEMBER OF OWN FAMILY BY BIRTH	OFTEN. . . . . 1<br>SOMETIMES. . . . . . . 2<br>ONCE. 3	
	b. [ ] IN-LAW/MEMBER OF PARTNER'S FAMILY	OFTEN. . . . . 1<br>SOMETIMES. . . . . . . 2<br>ONCE. 3	
	c. [ ] FORMER HUSBAND/LIVE-IN PARTNER R HAD MORE THAN 12 MOS AGO	OFTEN. . . . . 1<br>SOMETIMES. . . . . . . 2<br>ONCE. 3	
	d. [ ] FORMER BOYFRIEND R HAD MORE THAN 12 MOS AGO	OFTEN. . . . . 1<br>SOMETIMES. . . . . . . 2<br>ONCE. 3	
	e. [ ] TEACHER	OFTEN. . . . . 1<br>SOMETIMES. . . . . . . 2<br>ONCE. 3	
	f. [ ] EMPLOYER/SOMEONE AT WORK	OFTEN. . . . . 1<br>SOMETIMES. . . . . . . 2<br>ONCE. 3	
	g. [ ] POLICE/SOLDIER	OFTEN. . . . . 1<br>SOMETIMES. . . . . . . 2<br>ONCE. 3	
	h. [ ] NEIGHBOR/SOMEONE IN COMMUNITY	OFTEN. . . . . 1<br>SOMETIMES. . . . . . . 2<br>ONCE. 3	
	i. [ ] STRANGER	OFTEN. . . . . 1<br>SOMETIMES. . . . . . . 2<br>ONCE. 3	
	j. [ ] OTHER- SPECIFY_____________	OFTEN. . . . . 1<br>SOMETIMES. . . . . . . 2<br>ONCE. 3	
➢ Forced sexual intercourse			
503	In the past 12 months, has anyone other than the partners we have already talked about [husband, live-in	YES. . . . . 1<br>NO 2	 ➢505<br>➢505

QUESTIONS and FILTERS		CODING CATEGORIES	SKIP TO
	partner, steady boyfriend] forced you to have sexual intercourse against your will?	NO ANSWER 9	
504	Who has forced you to have sexual intercourse? DO NOT READ CATEGORIES, BUT TICK ALL THAT R MENTIONS; USE ONLY ONE CATEGORY FOR EACH PERSON MENTIONED.<br><br>THEN ASK ABOUT FREQUENCY FOR THOSE TICKED:<br>Did this happen often, sometimes or only once in the past 12 months?		
	a. [ ] FATHER	OFTEN. . . . . 1<br>SOMETIMES. . . . . . . 2<br>ONCE. 3	
	b. [ ] OTHER BIRTH RELATIVE	OFTEN. . . . . 1<br>SOMETIMES. . . . . . . 2<br>ONCE. 3	
	c. [ ] STEP-FATHER	OFTEN. . . . . 1<br>SOMETIMES. . . . . . . 2<br>ONCE. 3	
	d. [ ] INLAW	OFTEN. . . . . 1<br>SOMETIMES. . . . . . . 2<br>ONCE. 3	
	e. [ ] FORMER HUSBAND/PARTNER/BOYFRIEND R HAD MORE THAN 12 MOS AGO	OFTEN. . . . . 1<br>SOMETIMES. . . . . . . 2<br>ONCE. 3	
	f. [ ] OWN FRIEND/ACQUAINTANCE	OFTEN. . . . . 1<br>SOMETIMES. . . . . . . 2<br>ONCE. 3	
	g. [ ] FRIEND OF FAMILY OR FAMILY MEMBER	OFTEN. . . . . 1<br>SOMETIMES. . . . . . . 2<br>ONCE. 3	
	h. [ ] TEACHER	OFTEN. . . . . 1<br>SOMETIMES. . . . . . . 2<br>ONCE. 3	
	i. [ ] EMPLOYER/SOMEONE AT WORK	OFTEN. . . . . 1<br>SOMETIMES. . . . . . . 2<br>ONCE. 3	
	j. [ ] POLICE/SOLDIER	OFTEN. . . . . 1<br>SOMETIMES. . . . . . . 2<br>ONCE. 3	
	k. [ ] PRIEST/PASTOR/RELIGIOUS LEADER	OFTEN. . . . . 1<br>SOMETIMES. . . . . . . 2<br>ONCE. 3	
	l. [ ] NEIGHBOR/SOMEONE IN COMMUNITY	OFTEN. . . . . 1<br>SOMETIMES. . . . . . . 2<br>ONCE. 3	
	m. [ ] STRANGER	OFTEN. . . . . 1<br>SOMETIMES. . . . . . . 2<br>ONCE. 3	
	n. [ ] OTHER- SPECIFY_____________	OFTEN. 1	

QUESTIONS and FILTERS		CODING CATEGORIES	SKIP TO
		SOMETIMES. . . . . . . 2<br>ONCE. 3	
505	As far as you know, did your father ever beat your mother?	YES . . . . .1<br>NO . . . . .2<br>DON'T KNOW. . .8<br>NO ANSWER . . . 9	

SUMMARY<br>Interviewer instructions: Review responses to Q411-417 and Q501 and code the following 2 variables.		
**506**	**Experienced at least one form of physical violence in the past 12 months?**	YES. . . . . 1<br>If ANY of the following conditions are met:<br>Q411 = 1, 2 or 3<br>Q412=1, 2 or 3<br>Q413=1, 2 or 3<br>Q414=1, 2 or 3<br>Q415=1, 2 or 3<br>Q416=1, 2 or 3<br>Q417=1, 2 or 3<br>Q501=1<br>NO/DON'T KNOW/NO ANSWER. . . . . 2<br>If NONE of the conditions above are met
**507**	**Experienced at least one form of sexual violence in the past 12 months?**	YES. . . . . 1<br>If ANY of the following conditions are met:<br>Q418 = 1, 2 or 3<br>Q419=1, 2 or 3<br>Q503=1<br>NO/DON'T KNOW/NO ANSWER . . . . 2<br>If NONE of the conditions above are met
**FILTER**		
**If Q506 =1 OR Q507=1 then CONTINUE WITH SECTION 6**<br>**If Q506=2 AND Q507=2 then GO TO SECTION 7**		

Section 6 Use of services for IPV or other GBV within past 12 months

INTERVIEWER: You have just shared with me that you recently experienced an act or situation that I'm going to refer to as "gender-based violence." I would now like to ask you about any actions you took as a result of this experience.

QUESTIONS and FILTERS		CODING CATEGORIES	SKIP TO
601	During the last 12 months, did you ever seek help to stop any type of this gender-based violence you experienced -- or seek care or	YES, SOUGHT HELP . . . . .1<br>NO, DID NOT SEEK HELP 2	➢ SECTION 7

QUESTIONS and FILTERS		CODING CATEGORIES	SKIP TO
	treatment for the consequences of violence you experienced?		
602	Where and who did you go to for help in the past 12 months?<br><br>DO NOT READ; TICK ALL THAT R MENTIONS	a. [ ] OWN FAMILY MEMBER<br>b. [ ] IN-LAWS<br>c. [ ] HUSBAND/PARTNER<br>d. [ ] FORMER PARTNER<br>e. [ ] BEST MAN/BEST WOMEN<br>f. [ ] FRIEND<br>g. [ ] NEIGHBOUR<br>h. [ ] RELIGIOUS LEADER<br>i. [ ] LOCAL OFFICIAL<br>j. [ ] POLICE<br>k. [ ] LAWYER/LEGAL SERVICES<br>l. [ ] NGO/FBO<br>m. [ ] WELFARE OFFICE<br>n. [ ] SCHOOL OFFICIAL<br>o. [ ] SAFE HOUSE/SHELTER<br>p. [ ] HEALTH FACILITY<br>q. [ ] OTHER: SPECIFY<br>____________________	
603	Where/who did you go to first for help?<br><br>RECORD LETTER (a. – q.) OF RESPONSE FROM Q602	WENT TO FIRST [ ]	
604	How many times in the past 12 months did you visit a <u>health facility</u> to get care, treatment, or support for the gender-based violence you experienced?	NUMBER OF VISITS . . . [ ] [ ] [ ]<br><br>IF GREATER THAN 1, BEGIN BY ASKING Q605 ABOUT THE MOST RECENT VISIT AND CONTINUE ASKING ABOUT UP TO 3 VISITS	IF “0,” SKIP TO SECTION 7

QUESTIONS and FILTERS		a.<br>MOST RECENT HEALTH FACILITY VISIT	b.<br>NEXT MOST RECENT HEALTH FACILITY VISIT	c.<br>NEXT MOST RECENT HEALTH FACILITY VISIT
**INTERVIEWER:** I am now going to ask you some questions about this visit/these visits, starting with your most recent visit.				
605	Was the health facility a dispensary, health centre, or hospital?	Dispensary. . . . . 1<br>Health centre . . . . . . 2<br>Hospital . . . . .3<br>Other. . . . . 4<br>Specify: ___________	Dispensary. . . . . 1<br>Health centre . . . . . . 2<br>Hospital . . . . .3<br>Other. . . . . .4<br>Specify: ___________	Dispensary. . . . . 1<br>Health centre . . . . . . 2<br>Hospital . . . . .3<br>Other. . . . . 4<br>Specify: ___________
606	What is the name of the health facility?	Facility name:<br>____________________	Facility name:<br>____________________	Facility name:<br>____________________
607	FILTER:<br>DO NOT ASK THIS QUESTION FOR COLUMN a. ASK ONLY FOR COLUMNS b. AND c., AS APPROPRIATE.<br>Was this visit for the same incident of violence as your previous visit to a health facility?	X	YES, SAME INCIDENT . . . . .1<br>NO, DIFFERENT INCIDENT. 2	YES, SAME INCIDENT . . . . .1<br>NO, DIFFERENT INCIDENT. 2
608	What types of services were you seeking or being referred to?<br><br>DO NOT READ; RECORD ALL THAT R MENTIONS	[ ] TREATMENT FOR PHYSICAL INJURIES<br>[ ] TREATMENT FOR RAPE (OR SEXUAL VIOLENCE)<br>[ ] PSYCHOSOCIAL COUNSELING/SUPPORT<br>[ ] TEST FOR SEXUALLY TRANSMITTED INFECTION (STI)<br>[ ] TREATMENT FOR STI<br>[ ] PREGNANCY TEST<br>[ ] FAMILY PLANNING<br>[ ] EMERGENCY CONTRACEPTION<br>[ ] HIV TEST<br>[ ] HIV PEP<br>[ ] PEP ADHERENCE<br>[ ] FORENSIC EXAM<br>[ ] REFERRAL TO ANOTHER SERVICE<br>[ ] OTHER, Specify:<br>_______________	[ ] TREATMENT FOR PHYSICAL INJURIES<br>[ ] TREATMENT FOR RAPE (OR SEXUAL VIOLENCE)<br>[ ] PSYCHOSOCIAL COUNSELING/SUPPORT<br>[ ] TEST FOR SEXUALLY TRANSMITTED INFECTION (STI)<br>[ ] TREATMENT FOR STI<br>[ ] PREGNANCY TEST<br>[ ] FAMILY PLANNING<br>[ ] EMERGENCY CONTRACEPTION<br>[ ] HIV TEST<br>[ ] HIV PEP<br>[ ] PEP ADHERENCE<br>[ ] FORENSIC EXAM<br>[ ] REFERRAL TO ANOTHER SERVICE<br>[ ] OTHER, Specify:<br>_______________	[ ] TREATMENT FOR PHYSICAL INJURIES<br>[ ] TREATMENT FOR RAPE (OR SEXUAL VIOLENCE)<br>[ ] PSYCHOSOCIAL COUNSELING/SUPPORT<br>[ ] TEST FOR SEXUALLY TRANSMITTED INFECTION (STI)<br>[ ] TREATMENT FOR STI<br>[ ] PREGNANCY TEST<br>[ ] FAMILY PLANNING<br>[ ] EMERGENCY CONTRACEPTION<br>[ ] HIV TEST<br>[ ] HIV PEP<br>[ ] PEP ADHERENCE<br>[ ] FORENSIC EXAM<br>[ ] REFERRAL TO ANOTHER SERVICE<br>[ ] OTHER, Specify:<br>_______________

QUESTIONS and FILTERS		a.<br>MOST RECENT HEALTH FACILITY VISIT	b.<br>NEXT MOST RECENT HEALTH FACILITY VISIT	c.<br>NEXT MOST RECENT HEALTH FACILITY VISIT
609	FILTER: Is the category "Treatment for rape (Sexual Violence) ticked in Q608?	YES . . . . 1<br>NO . . . . 2<br>GO TO 611	YES . . . . 1<br>NO . . . . 2<br>GO TO 611	YES . . . . 1<br>NO . . . . 2<br>GO TO 611
610	Did you go to the health facility within 72 hours of the rape/sexual violence incident?	YES . . . . 1<br>NO . . . . 2<br>DON'T KNOW 8	YES . . . . 1<br>NO . . . . 2<br>DON'T KNOW 8	YES . . . . 1<br>NO . . . . 2<br>DON'T KNOW 8
611	How or why did you choose or go to this facility?<br><br>DO NOT READ; RECORD ALL THAT R MENTIONS<br>PROBE: If you were referred, who referred you?	[ ] AWARE THAT THIS FACILITY CAN TREAT THE PROBLEM<br>[ ] FACILITY IS CLOSE/ NEARBY<br>[ ] ALWAYS GO HERE FOR HEALTH SERVICES<br>[ ] COULD GO HERE WITHOUT OTHERS KNOWING<br>[ ] REFERRED HERE OR ACCOMPANIED BY POLICE<br>[ ] REFERRED HERE BY LOCAL AUTHORITIES<br>[ ] REFERRED HERE BY ANOTHER HEALTH FACILITY (SPECIFY_________)<br>[ ] REFERRED HERE BY SUPPORT ORGANIZATION (NGO/FBO)<br>(SPECIFY_________)<br>[ ] REFERRED HERE BY RELATIVE<br>[ ] REFERRED HERE BY SCHOOL<br>[ ] REFERRED HERE BY WELFARE OFFICER<br>[ ] REFERRED HERE BY FRIEND/NEIGHBOR<br>[ ] IT WAS THE ONLY OPTION AVAILABLE<br>[ ] OTHER SPECIFY_____________	[ ] AWARE THAT THIS FACILITY CAN TREAT THE PROBLEM<br>[ ] FACILITY IS CLOSE/ NEARBY<br>[ ] ALWAYS GO HERE FOR HEALTH SERVICES<br>[ ] COULD GO HERE WITHOUT OTHERS KNOWING<br>[ ] REFERRED HERE OR ACCOMPANIED BY POLICE<br>[ ] REFERRED HERE BY LOCAL AUTHORITIES<br>[ ] REFERRED HERE BY ANOTHER HEALTH FACILITY (SPECIFY_________)<br>[ ] REFERRED HERE BY SUPPORT ORGANIZATION (NGO/FBO)<br>(SPECIFY_________)<br>[ ] REFERRED HERE BY RELATIVE<br>[ ] REFERRED HERE BY SCHOOL<br>[ ] REFERRED HERE BY WELFARE OFFICER<br>[ ] REFERRED HERE BY FRIEND/NEIGHBOR<br>[ ] IT WAS THE ONLY OPTION AVAILABLE<br>[ ] OTHER SPECIFY_____________	[ ] AWARE THAT THIS FACILITY CAN TREAT THE PROBLEM<br>[ ] FACILITY IS CLOSE/ NEARBY<br>[ ] ALWAYS GO HERE FOR HEALTH SERVICES<br>[ ] COULD GO HERE WITHOUT OTHERS KNOWING<br>[ ] REFERRED HERE OR ACCOMPANIED BY POLICE<br>[ ] REFERRED HERE BY LOCAL AUTHORITIES<br>[ ] REFERRED HERE BY ANOTHER HEALTH FACILITY (SPECIFY_________)<br>[ ] REFERRED HERE BY SUPPORT ORGANIZATION (NGO/FBO)<br>(SPECIFY_________)<br>[ ] REFERRED HERE BY RELATIVE<br>[ ] REFERRED HERE BY SCHOOL<br>[ ] REFERRED HERE BY WELFARE OFFICER<br>[ ] REFERRED HERE BY FRIEND/NEIGHBOR<br>[ ] IT WAS THE ONLY OPTION AVAILABLE<br>[ ] OTHER SPECIFY_____________

QUESTIONS and FILTERS		a.<br>MOST RECENT HEALTH FACILITY VISIT			b.<br>NEXT MOST RECENT HEALTH FACILITY VISIT			c.<br>NEXT MOST RECENT HEALTH FACILITY VISIT		
612	What services did you receive at this health facility visit? READ EACH SERVICE AND RECORD ANSWER									
	**Service**	**YES**	**NO**	**DK**	**YES**	**NO**	**DK**	**YES**	**NO**	**DK**
	1. Screened and counseled for gender-based violence	1	2	8	1	2	8	1	2	8
	2. Physical exam	1	2	8	1	2	8	1	2	8
	3. Mental health exam	1	2	8	1	2	8	1	2	8
	4. Treated for physical injuries	1	2	8	1	2	8	1	2	8
	5. Forensic exam	1	2	8	1	2	8	1	2	8
	6. Forensic evidence collected	1	2	8	1	2	8	1	2	8
	7. PF3 form filled out	1	2	8	1	2	8	1	2	8
	8. Psychosocial counseling	1	2	8	1	2	8	1	2	8
	9. Family planning counseling	1	2	8	1	2	8	1	2	8
	10. HIV counseling	1	2	8	1	2	8	1	2	8
	11. PEP adherence counseling	1	2	8	1	2	8	1	2	8
	12. Pregnancy test	1	2	8	1	2	8	1	2	8
	13. HIV test	1	2	8	1	2	8	1	2	8
	14. STI test	1	2	8	1	2	8	1	2	8
	15. FP method	1	2	8	1	2	8	1	2	8
	16. Emergency contraception	1	2	8	1	2	8	1	2	8
	17. STI treatment	1	2	8	1	2	8	1	2	8
	18. PEP for HIV	1	2	8	1	2	8	1	2	8
	19. Tetanus toxoid vaccine	1	2	8	1	2	8	1	2	8
613	Overall, would you describe the services that you received as excellent, good, fair, poor, or very poor?	EXCELLENT. . . . . 1<br>GOOD. . . . . 2<br>FAIR. . . . . 3<br>POOR. . . . . 4<br>VERY POOR. 5			EXCELLENT. . . . . 1<br>GOOD. . . . . 2<br>FAIR. . . . . 3<br>POOR. . . . . 4<br>VERY POOR. 5			EXCELLENT. . . . . 1<br>GOOD. . . . . 2<br>FAIR. . . . . 3<br>POOR. . . . . 4<br>VERY POOR. 5		

QUESTIONS and FILTERS		a.<br>**MOST RECENT HEALTH FACILITY VISIT**	b.<br>**NEXT MOST RECENT HEALTH FACILITY VISIT**	c.<br>**NEXT MOST RECENT HEALTH FACILITY VISIT**
614	At this visit to the health facility, were you referred to services that are provided <u>outside this facility</u>?	YES . . . . 1<br>NO . . 2 GO TO **FILTER 2**	YES . . . . 1<br>NO . . 2 GO TO **FILTER 2**	YES . . . . 1<br>NO . . 2 GO TO **FILTER 2**
615	What services were you referred to? And did you follow through on this/these referrals and go to these services to which you were referred? READ EACH SERVICE AND RECORD ANSWER			
	a. Psychosocial care	YES, REFERRED AND WENT . . . . 1<br>YES, REFERRED BUT DID NOT GO . . . . 2<br>NOT REFERRED . . . 3	YES, REFERRED AND WENT . . . . 1<br>YES, REFERRED BUT DID NOT GO . . . . 2<br>NOT REFERRED . . . 3	YES, REFERRED AND WENT . . . . 1<br>YES, REFERRED BUT DID NOT GO . . . . 2<br>NOT REFERRED . . . 3
	b. Police	YES, REFERRED AND WENT . . . . 1<br>YES, REFERRED BUT DID NOT GO . . . . 2<br>NOT REFERRED . . . 3	YES, REFERRED AND WENT . . . . 1<br>YES, REFERRED BUT DID NOT GO . . . . 2<br>NOT REFERRED . . . 3	YES, REFERRED AND WENT . . . . 1<br>YES, REFERRED BUT DID NOT GO . . . . 2<br>NOT REFERRED . . . 3
	c. Safe house or shelter	YES, REFERRED AND WENT . . . . 1<br>YES, REFERRED BUT DID NOT GO . . . . 2<br>NOT REFERRED . . . 3	YES, REFERRED AND WENT . . . . 1<br>YES, REFERRED BUT DID NOT GO . . . . 2<br>NOT REFERRED . . . 3	YES, REFERRED AND WENT . . . . 1<br>YES, REFERRED BUT DID NOT GO . . . . 2<br>NOT REFERRED . . . 3
	d. Clinical care at higher level facility	YES, REFERRED AND WENT . . . . 1<br>YES, REFERRED BUT DID NOT GO . . . . 2<br>NOT REFERRED . . . 3	YES, REFERRED AND WENT . . . . 1<br>YES, REFERRED BUT DID NOT GO . . . . 2<br>NOT REFERRED . . . 3	YES, REFERRED AND WENT . . . . 1<br>YES, REFERRED BUT DID NOT GO . . . . 2<br>NOT REFERRED . . . 3
	e. Legal services	YES, REFERRED AND WENT . . . . 1<br>YES, REFERRED BUT DID NOT GO . . . . 2<br>NOT REFERRED . . . 3	YES, REFERRED AND WENT . . . . 1<br>YES, REFERRED BUT DID NOT GO . . . . 2<br>NOT REFERRED . . . 3	YES, REFERRED AND WENT . . . . 1<br>YES, REFERRED BUT DID NOT GO . . . . 2<br>NOT REFERRED . . . 3
	f. Other1: SPECIFY<br>________________	YES, REFERRED AND WENT . . . . 1<br>YES, REFERRED BUT DID NOT GO . . . . 2<br>NOT REFERRED . . . 3	YES, REFERRED AND WENT . . . . 1<br>YES, REFERRED BUT DID NOT GO . . . . 2<br>NOT REFERRED . . . 3	YES, REFERRED AND WENT . . . . 1<br>YES, REFERRED BUT DID NOT GO . . . . 2<br>NOT REFERRED . . . 3
	g. Other2: SPECIFY<br>________________	YES, REFERRED AND WENT . . . . 1<br>YES, REFERRED BUT DID NOT GO . . . . 2<br>NOT REFERRED . . . 3	YES, REFERRED AND WENT . . . . 1<br>YES, REFERRED BUT DID NOT GO . . . . 2<br>NOT REFERRED . . . 3	YES, REFERRED AND WENT . . . . 1<br>YES, REFERRED BUT DID NOT GO . . . . 2<br>NOT REFERRED . . . 3

**QUESTIONS and FILTERS**		**a.**<br>**MOST RECENT HEALTH FACILITY VISIT**	**b.**<br>**NEXT MOST RECENT HEALTH FACILITY VISIT**	**c.**<br>**NEXT MOST RECENT HEALTH FACILITY VISIT**
**FILTER 1**: If any response to Q615 is 2 (REFERRED BUT DIDN’T GO), then ask Q616. Otherwise GO TO **FILTER 2**.				
616	What were the reasons that you didn’t follow through on this referral/these referrals?<br><br>RECORD ALL THAT R MENTIONS; DO NOT READ	[ ] DIDN’T WANT THE SERVICE<br>[ ] COULD’T AFFORD THE SERVICE<br>[ ] DIDN’T HAVE/ COULDN’T AFFORD TRANSPORTATION<br>[ ] DIDN’T HAVE TIME<br>[ ] HAD TO WORK<br>[ ] DIDN’T HAVE CHILDCARE<br>[ ] HAD FAMILY RESPONSIBILITIES/ HOUSEHOLD OBLIGATIONS<br>[ ] HUSBAND/ PARTNER WOUDN’T ALLOW IT<br>[ ] DIDN’T KNOW WHERE TO GO<br>[ ] AFRAID TO BE SEEN/THAT OTHERS WOULD FIND OUT<br>[ ] OTHER<br>SPECIFY:____________	[ ] DIDN’T WANT THE SERVICE<br>[ ] COULD’T AFFORD THE SERVICE<br>[ ] DIDN’T HAVE/ COULDN’T AFFORD TRANSPORTATION<br>[ ] DIDN’T HAVE TIME<br>[ ] HAD TO WORK<br>[ ] DIDN’T HAVE CHILDCARE<br>[ ] HAD FAMILY RESPONSIBILITIES/ HOUSEHOLD OBLIGATIONS<br>[ ] HUSBAND/ PARTNER WOUDN’T ALLOW IT<br>[ ] DIDN’T KNOW WHERE TO GO<br>[ ] AFRAID TO BE SEEN/THAT OTHERS WOULD FIND OUT<br>[ ] OTHER<br>SPECIFY:____________	[ ] DIDN’T WANT THE SERVICE<br>[ ] COULD’T AFFORD THE SERVICE<br>[ ] DIDN’T HAVE/ COULDN’T AFFORD TRANSPORTATION<br>[ ] DIDN’T HAVE TIME<br>[ ] HAD TO WORK<br>[ ] DIDN’T HAVE CHILDCARE<br>[ ] HAD FAMILY RESPONSIBILITIES/ HOUSEHOLD OBLIGATIONS<br>[ ] HUSBAND/ PARTNER WOUDN’T ALLOW IT<br>[ ] DIDN’T KNOW WHERE TO GO<br>[ ] AFRAID TO BE SEEN/THAT OTHERS WOULD FIND OUT<br>[ ] OTHER<br>SPECIFY:____________
**FILTER 2:**		If Q604 >1, then continue with column b; else GO TO SECTION 7	If Q604 >2, then continue with column c; else GO TO SECTION 7	GO TO SECTION 7

Section 7 Participation in GBV community programs within past 12 months

INTERVIEWER: I would now like to ask you about your participation in community activities.

QUESTIONS and FILTERS		CODING CATEGORIES	SKIP TO
➢ Participation in community activities of any kind			
701a	In the past 12 months, did you attend or participate in any kind of group activity, association or organisation?<br><br>IF RESPONSE IS NO OR DON'T KNOW PROBE: This includes organisations like women's or community groups, and religious and political associations.	YES . . . . 1<br><br>NO . . . . 2<br>DON'T KNOW 8	 <br>➢ 702a<br>➢ 702a
701b	What kind(s) of group activity, association or organisation did you attend or participate in?<br><br>DO NOT READ; TICK ALL THAT R MENTIONS	a. [ ] Sports group<br>b. [ ] Music and dance group<br>c. [ ] Women's group/Co-Op<br>d. [ ] Burial society<br>e. [ ] Religious group<br>f. [ ] Political group<br>g. [ ] HIV Care and support group<br>h. [ ] Peer education group<br>i. [ ] Youth group<br>j. [ ] Community Health group<br>k. [ ] Parent-teachers association<br>l. [ ] Sewing group<br>m. [ ] Development committee (e.g. land trusts, water & sanitation committee)<br>n. [ ] Other, specify<br>______________________	
➢ Awareness of and participation in HJFMRI GBV Community Programs			
702a	Have you heard about a programme called the HJFMRI Gender-Based Violence Programme – also known as the HJFMRI GBV Programme?	YES . . . . .1<br>NO 2	 ➢ 703a
702b	Did you participate in a launch of this programme?	YES . . . . .1<br>NO 2	

QUESTIONS and FILTERS		CODING CATEGORIES	SKIP TO
703a	Have you heard about "Men as Partners" or "MAP," which is a group education program that works with men to raise awareness about gender-based violence, to reduce acceptability of gender-based violence, and to engage men in stopping violence against women and children?	YES . . . . 1<br>NO . . . . .2<br>DON'T KNOW. . . 8	 ➢ 704a<br>➢ 704a
703b	Do you personally know any man or men who have participated in the "Men As Partners" programme? If so, how often does he/do they participate?<br><br>*IF R KNOWS MORE THAN ONE MAN WHO PARTICIPATES, ASK HER TO DESCRIBE THE FREQUENCY FOR THE PERSON WHO IS MOST ACTIVELY INVOLVED.*	Know at least one person who participates often . . . . 1<br>Know at least one person who sometimes participates . . . . .2<br>Know at least one person who participated once . . . . .3<br>Don't know anyone who has participated 4	
704a	Have you heard about "Couples Connect," which is a program that works with couples to improve the way women and men in partnerships (e.g. husbands and wives) talk to each other?	YES . . . . .1<br>NO . . . . .2<br>DON'T KNOW. 8	 ➢ 705a<br>➢ 705a
704b	How often have you participated in "Couples Connect"?	OFTEN. . . . . 1<br>SOMETIMES. . . . . .2<br>ONCE. . . . . 3<br>NEVER. . . . . 4<br>DON'T KNOW/<br>DON'T REMEMBER 8	
705a	Have you heard in the past 12 months about community dialogues-- that is, gatherings organized for community members to come together to discuss issues of gender-based violence/GBV or violence against women and children?	YES . . . . 1<br>NO . . . . 2<br>DON'T KNOW. . .8	 ➢ 706a<br>➢ 706a
705b	In the past 12 months, how often have you participated in such dialogues?	OFTEN. . . . . 1<br>SOMETIMES. . . . . 2<br>ONCE. . . . . .3<br>NEVER. . . . . 4<br>DON'T KNOW/<br>DON'T REMEMBER 8	
706a	Are there Community Action Groups in your community that focus on gender-based violence/GBV or violence against women and children?	YES . . . . .1<br>NO . . . . 2<br>DON'T KNOW. . . 8	 ➢ 707a<br>➢ 707a

QUESTIONS and FILTERS		CODING CATEGORIES	SKIP TO
706b	Are you a part of such a Community Action Group? If so, how often have you participated in the past 12 months?	OFTEN. . . . . .1<br>SOMETIMES. . . . . . .2<br>ONCE. . . . . 3<br>NOT PART OF SUCH ACTION GROUP . . . 4<br>DON'T KNOW. 8	
707a	Are you aware of any media campaigns (for example, posters, billboards, radio, or TV messages) during the past 12 months that addressed gender-based violence/GBV or violence against women and children?	YES . . . . .1<br>NO 2	 ➢ 708
707b	Did the campaign have a name or slogan? Can you tell me the name?	R MENTIONED "BE A ROLE MODEL". . . . . .1<br>R MENTIONED NAME(S) OTHER THAN "BE A ROLE MODEL". . . 2<br>R INDICATED THE CAMPAIGN HAS NO NAME . . . . 3<br>R INDICATED IT HAS A NAME, BUT SHE DOESN'T REMEMBER IT 8	 ➢ 708<br>➢ 708<br>➢ 708
707c	How often during the last 12 months have you seen or heard "Be a Role Model" episodes or stories?	OFTEN. . . . . 1<br>SOMETIMES. . . . . 2<br>ONCE. . . . . .3<br>NEVER. . . . . 4<br>DON'T KNOW/<br>DON'T REMEMBER 8	

QUESTIONS and FILTERS		CODING CATEGORIES	SKIP TO
708	I'd now like to ask you about some other community activities that may have **included information about gender-based violence/GBV or violence against women and children**. I'm going to read to you the events and I'd like you to tell me if you participated in them in the last 12 months, and if so, how often.<br><br>READ ALL CATEGORIES AND ASK ABOUT FREQUENCY.		
	a. Events held during special, national and international days (e.g. Nane Nane, Saba Saba, World AIDS Day, International Women's Day, etc)	Participated:<br>OFTEN. . . . . 1<br>SOMETIMES. . . . . .2<br>ONCE (in past 12 mos) . . . . 3<br>NEVER. . . . . 4<br>DON'T KNOW/<br>DON'T REMEMBER 8	 <br><br><br>➢ 708c<br><br>➢ 708c
	b. Was any information on GBV or violence against women and children given out or discussed?	YES . . . . 1<br>NO . . . . 2<br>DON'T KNOW/<br>DON'T REMEMBER 8	
	c. Community sporting events?	Participated:<br>OFTEN. . . . . 1<br>SOMETIMES. . . . . .2<br>ONCE (in past 12 mos) . . . . 3<br>NEVER. . . . . 4<br>DON'T KNOW/<br>DON'T REMEMBER 8	 <br><br><br>➢ 708e<br><br>➢ 708e
	d. Was any information on GBV or violence against women and children given out or discussed?	YES . . . . 1<br>NO . . . . 2<br>DON'T KNOW/<br>DON'T REMEMBER 8	
	e. Street outreach, or outreach activities using boda boda?	Participated:<br>OFTEN. . . . . 1<br>SOMETIMES. . . . . 2<br>ONCE. . . . . .3<br>NEVER. . . . . 4<br>DON'T KNOW/<br>DON'T REMEMBER 8	 <br><br><br>➢ 709<br><br>➢ 709
	f. Was any information on GBV or violence against women and children given out or discussed?	YES . . . . 1<br>NO . . . . 2<br>DON'T KNOW/<br>DON'T REMEMBER 8	

QUESTIONS and FILTERS		CODING CATEGORIES	SKIP TO
709	Have you heard or seen any public messages that target men in bars about stopping violence against women and children? If so, how often in the past 12 months?	OFTEN. . . . . 1<br>SOMETIMES. . . . . 2<br>ONCE. . . . . 3<br>NEVER. . . . . 4<br>DON'T KNOW/<br>DON'T REMEMBER 8	
710	Apart from what we have already discussed, in the last 12 months have you heard about any other activities in your community aimed to stop GBV or violence against women and children?	YES . . . . 1<br><br>a.[ ]SPECIFY ACTIVITY:<br>________________________________<br><br>b.[ ] SPECIFY ACTIVITY:<br>________________________________<br><br>c.[ ] SPECIFY ACTIVITY:<br>________________________________<br><br>d.[ ] SPECIFY ACTIVITY:<br>________________________________<br><br>NO 2	 <br><br><br><br><br><br><br><br><br><br><br><br>➢ Section 8

QUESTIONS and FILTERS		CODING CATEGORIES	SKIP TO
711	How often during the last 12 months have you participated in this/these activities?<br><br>FOR EACH OF THE SPECIFIC ACTIVITIES MENTIONED IN 710 (a-d), MARK THE FREQUENCY OF PARTICIPATION.	a.<br>OFTEN. . . . . 1<br>SOMETIMES. . . . . 2<br>ONCE. . . . . 3<br>NEVER. . . . . 4<br>DON'T KNOW/<br>DON'T REMEMBER. . . 8<br><br>b.<br>OFTEN. . . . . 1<br>SOMETIMES. . . . . 2<br>ONCE. . . . . 3<br>NEVER. . . . . 4<br>DON'T KNOW/<br>DON'T REMEMBER. . . 8<br><br>c.<br>OFTEN. . . . . 1<br>SOMETIMES. . . . . 2<br>ONCE. . . . . 3<br>NEVER. . . . . 4<br>DON'T KNOW/<br>DON'T REMEMBER. . . 8<br><br>d.<br>OFTEN. . . . . 1<br>SOMETIMES. . . . . 2<br>ONCE. . . . . 3<br>NEVER. . . . . 4<br>DON'T KNOW/<br>DON'T REMEMBER. . . 8	

Section 8 GBV knowledge and attitudes, gender norms, and behavior change

INTERVIEWER: Finally, I'd like to ask you about some types of violence in your community and your thoughts about that.

QUESTIONS and FILTERS		CODING CATEGORIES	SKIP TO
➢ Awareness of recent cases in the community and community response			
801a	Have there been any cases in your community within the past 12 months where a woman has been physically or sexually abused by her husband or partner?	YES . . . . .1<br>NO . . . . 2<br>DON'T KNOW 8	 ➢ 802a<br>➢ 802a
801b	How many cases are you aware of?<br><br>RECORD THE NUMBER OF CASES STATED BY THE RESPONDENT.	NUMBER OF CASES<br>[ ] [ ] [ ] [ ]<br><br>Don't Know = 9998	
802a	Have there been any cases in your community within the past 12 months where a child has been sexually abused?	YES . . . . .1<br>NO . . . . 2<br>DON'T KNOW 8	 ➢ 803<br>➢ 803
802b	How many cases are you aware of?<br><br>RECORD THE NUMBER OF CASES STATED BY THE RESPONDENT.	NUMBER OF CASES<br>[ ] [ ] [ ] [ ]<br><br>Don't Know = 9998	
803	In your opinion, in the past 12 months has your community done a good job of responding to cases of husband/partner violence and sexual abuse toward children?	Very good job . . . . .1<br>Good job . . . . 2<br>Average job. . . . . .3<br>Poor job . . . . 4<br>Very poor job . . . . 5<br>Don't know 8	
➢ Awareness of laws/policies and recent actions of local leaders on violence			
804	Are you familiar with (do you know) the Tanzanian laws and policies regarding violence against women and children?	VERY AWARE/ KNOWLEDGEABLE . . . 1<br>SOMEWHAT AWARE. . . . . .2<br>NOT AWARE . . . . 3<br>DON'T KNOW. 8	
805	In the past 12 months, how often have local leaders or officials in your community publically spoken out or taken action to address gender-based violence or violence against women and children?	OFTEN. . . . . 1<br>SOMETIMES. . . . . . 2<br>ONCE. . . . . 3<br>NEVER. . . . . .4<br>DON'T KNOW. 8	

➢ Definition or recognition of VAW (adapted from DHS and CHAMPION study)

806	In your opinion, do you consider it an act of violence against a woman when a woman's husband…	**YES**	**NO**	**DON'T KNOW**
	a. Does something to humiliate her in front of others	1	2	8
	b. Threatens to harm her or someone she is close to	1	2	8
	c. Insults her or makes her feel bad about herself	1	2	8
	d. Pushes, slaps, hits, or kicks her	1	2	8
	e. Threatens to attack her with a knife, gun or other weapon	1	2	8
	f. Physically forces her to have sexual intercourse even when she does not want to	1	2	8
	g. Forces her to do something sexual that she doesn't want to do	1	2	8
	h. Restricts her movement	1	2	8
	i. Kicks her out of the house	1	2	8
	j. Has an extramarital affair	1	2	8
	k. Makes all decisions about how family earnings are spent including the wife's earnings	1	2	8
807	In your opinion, do you consider it an act of violence against a woman when someone other than her husband …	**YES**	**NO**	**DON'T KNOW**
	a. Does something to humiliate her in front of others	1	2	8
	b. Threatens to harm her or someone she is close to	1	2	8
	c. Insults her or makes her feel bad about herself	1	2	8
	d. Pushes, slaps, hits, or kicks her	1	2	8
	e. Threatens to attack her with a knife, gun or other weapon	1	2	8
	f. Physically forces her to have sexual intercourse even when she does not want to	1	2	8
	g. Forces her to do something sexual that she doesn't want to do	1	2	8

➢ Recognition or attitudes of sexual violence against children

		AGREE	PARTIALLY AGREE	DO NOT AGREE
808	I'm going to read some statements to you and I'd like you to tell me if you agree, partially agree, or do not agree.			
	a. A child is sexually abused only when sexual intercourse has taken place	1	2	3
	b. When a child is sexually abused, the abuser is rarely a family member	1	2	3
	c. Only girls are sexually abused	1	2	3
	d. It is not possible for children under 10 years of age to experience sexual abuse	1	2	3
	e. Children from reputable families do not experience sexual abuse	1	2	3

➢ Attitudes about domestic/marital violence (from DHS)

		YES	NO	DON'T KNOW
809	In your opinion, is a husband justified in hitting or beating his wife in the following situations:			
	a. If she goes out without telling her husband/partner?	1	2	8
	b. If she neglects the children?	1	2	8
	c. If she argues with her husband/partner?	1	2	8
	d. If she refuses to have sex with her husband/partner?	1	2	8
	e. If she burns the food?	1	2	8

➢ Gender norms (GEM scale: Violence domain items)

		AGREE	PARTIALLY AGREE	DO NOT AGREE
810	I'm now going to read some statements and I'd like you to tell me if you agree, partially agree, or do not agree.			
	a. There are times when a woman deserves to be beaten.	1	2	3
	b. A woman should tolerate violence to keep her family together.	1	2	3
	c. It is alright for a man to beat his wife if she is unfaithful.	1	2	3
	d. A man can hit his wife if she won't have sex with him.	1	2	3
	e. If someone insults a man, he should defend his reputation with force if he has to.	1	2	3
	f. A man using violence against his wife is a private matter that shouldn't be discussed outside the couple	1	2	3

➢ Gender norms (GEM scale: Domestic chores and daily life domain Items)

		AGREE	PARTIALLY AGREE	DO NOT AGREE
811	*Continue reading statements…*			
	a. Changing diapers, giving a bath, and feeding kids is the mother's responsibility.	1	2	3
	b. A woman's role is taking care of her home and family.	1	2	3
	c. The husband should decide to buy the major household items.	1	2	3
	d. A man should have the final word about decisions in his home.	1	2	3
	e. A woman should obey her husband in all things.	1	2	3

➢ R's behavior change: Taking action		
812a	In the past 12 months, have you started a conversation about gender-based violence/GBV or violence against women or children with anyone?	Yes . . . . 1<br>No . . . . 2 GO TO 813a
812b	With whom did you have this conversation?<br><br>CHECK ALL THAT R MENTIONS.	a. [ ] Your partner(s)<br>b. [ ] Friend<br>c. [ ] Family Member<br>d. [ ] Neighbour or other community member<br>e. [ ] Other, specify ______________________<br>f. [ ] Other, specify ______________________
813a	In the past 12 months, did you personally witness any acts of GBV or violence against a woman or child-- or encounter someone who experienced this violence?	Yes . . . . 1<br>No . . . . 2 GO TO 814
813b	Did you take any action to try to stop it or help the survivor (for any of the times you witnessed it)?	Yes . . . . 1<br>No . . . . 2 GO TO 814
813c	What action(s) did you take?<br><br>DO NOT READ.CHECK ALL THAT R MENTIONS.	a. [ ] Listened to the survivor and offered emotional support<br>b. [ ] Provided information about services and programs designed to help survivors<br>c. [ ] Accompanied the survivor to a clinic or support agency<br>d. [ ] Talked with family members of the survivor about the violence<br>e. [ ] Tried to stop or stopped the violence<br>f. [ ] Reported the incident to authorities<br>g. [ ] Other, specify ___________________________________
814	Do you think differently about gender-based violence/ GBV or violence against women and children today than you did 12 months ago?	Yes . . . . .1<br>No 2
815	If you were to witness such violence today would you be more or less likely to take action than you would have 3 months ago?	MORE LIKELY . . . . .1<br>ABOUT AS LIKELY . . . . . 2<br>LESS LIKELY 3

Section 9 Completion of Interview

901	**INTERVIEWER:**<br><br>We are coming to the end of the interview. There is just one last step.<br><br>I'm going to give you a card. On this card are two pictures. No other information is written on the card. One picture is of a sad face, the other is of a happy face.<br><br>GIVE RESPONDENT THE CARD AND PEN.<br><br>No matter what you have already told me, I would like you to put a mark next to the sad picture if someone hurt **you** physically, touched you sexually, or made you do something sexual that you didn't want to in the last 12 months.<br><br>Please put a mark next to the happy face if this did not happen to you in the last 12 months. Once you have marked the card, please fold it over and put it in this bag. The bag contains similar cards from many other women. This will ensure that I do not know your answer.<br><br>DO NOT LOOK AT THE RESPONSE. ASK RESPONDENT TO PUT The MARKED AND FOLDED CARD INTO A BAG THAT ALSO CONTAINS OTHER COMPLETED CARDS. DO NOT RECORD ANYTHING ON THE CARD.	CARD COMPLETED....1<br><br>CARD NOT COMPLETED....2
902	I have asked you about many difficult things. How has talking about these things made you feel?<br><br>READ OPTIONS	GOOD/BETTER. . . . . 1<br>BAD/WORSE. . . . . .2<br>SAME/ NO DIFFERENCE. . . .3
903	We have now finished the interview. Do you have any comments, or is there anything else you would like to share?<br><br>RECORD COMMENTS.	
904	FINISH<br>I would like to thank you very much for helping us. I appreciate the time you have taken. I realize that these questions may have been difficult for you to answer, but it is only by hearing from women themselves that we can really understand about their health and experiences in life.<br><br>In case you are interested or if you ever hear of another person who needs help related to violence and the things we have been talking about, I'm going to leave you with the name and contact information of a service provider at __________________ Health Center/_________Hospital, who can offer assistance. This person is service provider at this health facility who also is working with us on this study. S/he is aware that we are giving out this information as part of the interview. If you go, you can be assured that s/he will keep anything that you say private. You can go whenever you feel ready to, either soon or later on.<br><br>LEAVE PAPER SLIP WITH NAME OF HEALTH FACILITY IN THE STUDY CLUSTER, NAME OF TATHMINI GBV DATA FOCAL PERSON, AND CONTACT INFORMATION.	

<table>
<tr><td></td><td colspan="2">If you are interested, I can also ask this service provider to contact you directly. If you would like this referral, I will take down your contact information, including your name and a safe place where or private way that s/he or another trained colleague can find you. I will record this information on a separate form, which will not be linked in any way with the interview we have just completed. I will then give this form to my team leader, who will provide the form directly to the service provider. Information you have shared during this interview will not be shared with the service provider.

Would you like me to put you in direct contact with a service provider?
YES…………………….1
NO…………………….2

IF THE RESPONDENT INDICATES ‘YES,’ FILL IN THE REFERRAL FORM, PLACE IT IN THE DESIGNATED/UNMARKED ENVELOPE AND GIVE IT TO THE TEAM LEADER.</td></tr>
<tr><td colspan="2">INTERVIEWER:</td><td>Record time of end of interview: [ ][ ]:[ ] [ ] (Use 24 hr clock)</td></tr>
<tr><td colspan="2"></td><td>Interview completed: _______Yes, complete ______No, incomplete

If interview is incomplete, please give reason (s):

Signature of Interviewer: ____</td></tr>
</table>

ANNEX B-1b.

Household Survey Questionnaire

Endline/Follow-up

English

ID: [ ][ ][ ][ ][ ][ ][ ]

HOUSEHOLD IDENTIFICATION

STUDY CLUSTER NAME _________________________ DISTRICT NAME __________________ CODE: [ ] [ ]

ENUMERATION AREA (EA) NUMBER: [ ] [ ] [ ] [ ] [ ] [ ] [ ] [ ] [ ] [ ] [ ] [ ]

NAME OF WARD _____________________________________ CODE: [ ] [ ] [ ]

VILLAGE/HAMLET/ STREET ______________________________ HOUSE NUMBER (if available) ____________

OTHER INFO TO IDENTIFY HOUSEHOLD (if needed)

____

INTERVIEW PARTICULARS

	1st VISIT	2nd VISIT	3rd VISIT	FINAL VISIT
DATE (DD/MM/YYYY)	_______________	______________	________________	DATE _ _ /_ _ / _ _ _ _
INTERVIEWERS ID	_______________	_______________	_______________	DD/ MM/ YYYY
RESULT*	[ ] [ ]	[ ] [ ]	[ ] [ ]	RESULT* [ ] [ ]
NEXT VISIT, IF SCHEDULED Date:	_______________	_______________		Total Number of visits: [ ]
Time:	______________	_______________		

* RESULT CODES:
01. Interview completed
02. No competent adult in household at time of visit
03. No eligible females living in household
04. Entire household absent for extended period of time
05. Refused by household head/representative
06. Selected participant not present; rescheduled
07. Selected participant not present; could not reschedule
08. Refused by selected female
09. Refused by parent
10. Other specify: ____________________

TOTAL # PERSONS IN HOUSEHOLD [ ][ ]

TOTAL # ELIGIBLE FEMALES AGED 15-49 [ ]

LINE # OF SELECTED ELIGIBLE FEMALE [ ]

STUDY CONSENT SUMMARY

For selected respondents ages 18-49, ADULT INFORMED CONSENT obtained?	YES [ ] NO [ ]
For selected respondents ages 15-17, PARENTAL CONSENT obtained?	YES [ ] NO [ ]
MINOR ASSENT obtained?	YES [ ] NO [ ]

QUESTIONNAIRE REVIEW AND PROCESSING			
TEAM LEADER SIGN OFF<br>NAME ________________<br>DATE _ _/_ _/ _ _ _ _<br>dd /mm/yyyy	QUESTIONNAIRE CHECKED BY<br>NAME<br>___________________<br>DATE _ _/_ _/ _ _ _ _<br>dd /mm/yyyy	OFFICE EDITOR<br>NAME<br>________________<br>DATE _ _/_ _/ _ _ _ _<br>dd /mm/yyyy	ENTERED BY<br>ENTRY 1: ________<br>ENTRY 2: ________

ELIGIBLE FEMALES IN THE HOUSEHOLD		
INTERVIEWER OR TEAM LEADER INSTRUCTIONS:<br><br>For purposes of this study, a household is defined as: A group of individuals who usually live and eat together, whether or not they are related by blood or marriage, with one person, male or female, acknowledged as the head of the household. A household can consist of one person or many persons.<br><br>Several households may reside in one dwelling. If this is the case, randomly select one of the households (writing household labels on pieces of paper, then randomly drawing from a bag).<br><br>Upon reaching the house, ask to speak to the household head. If s/he is not available, ask to speak to any (competent) adult who resides in the household. If such individual is not there, ask for a time when s/he will return.<br><br>Then proceed with <u>Brief Introduction of the Study</u>. Then ask the following questions of the household head/adult.		
1.	Please can you tell me how many people live here, and share food regularly?	TOTAL NUMBER OF<br>PEOPLE IN HOUSEHOLD [ ][ ]
2.	Is the head of the household male or female?	MALE ....1<br>FEMALE 2
3.	What is your relationship to the head of this household?	[ ] [ ] CIRCLE CODE BELOW AND ENTER HERE

CODES

01 HEAD
02 WIFE (PARTNER)
03 DAUGHTER
04 DAUGHTER IN LAW
05 GRANDDAUGHTER
06 MOTHER
07 MOTHER IN LAW
08 SISTER
09 SISTER IN LAW
10 ADOPTED/FOSTER/STEP DAUGHTER

11 HUSBAND(PARTNER)
12 SON
13 SON IN LAW
14 GRAND SON
15 FATHER
16 FATHER IN LAW
17 BROTHER
18 BROTHER IN LAW
19 ADOPTED/FOSTER/STEP SON

20 OTHER RELATIVE, SPECIFY: ______________________________

21 OTHER NON RELATIVE, SPECIFY: ______________________________

	LIST OF FEMALE HOUSEHOLD MEMBERS AGES 15-49 WHO HAVE LIVED IN HH FOR PAST 6 MONTHS	RELATIONSHIP TO HEAD OF HH	AGE
4.	Today I would like to talk to one girl or woman from your household. To enable me to identify with whom I should talk, would you please give me the first names of:<br>• all girls and women ages 15-49 who usually live in your household (and share food) AND<br>• who have lived in the household for the past 6 months	What is the relationship of NAME to the head of the household?<br><br>USE CODES BELOW	How old is NAME?
INTEVIEWER INSTRUCTIONS: List eligible females in the order given to you.			
**LINE #**	**NAME**	**CODE**	**AGE (YEARS)**
**1**			
**2**			
**3**			
**4**			
**5**			
**6**			
**7**			
**8**			

CODES

01	HEAD	07	MOTHER IN LAW
02	WIFE (PARTNER)	08	SISTER
03	DAUGHTER	09	SISTER IN LAW
04	DAUGHTER IN LAW	10	ADOPTED/FOSTER/STEP DAUGHTER
05	GRANDDAUGHTER	11	OTHER RELATIVE, SPECIFY:_____________________________
06	MOTHER	12	OTHER NON RELATIVE, SPECIFY:_________________________

RANDOM SELECTION OF PARTICIPANT

INTERVIEWER INSTRUCTIONS:

- Identify the last digit of the **ID Number**. Go to this **row** in the table below.
- Obtain the total **number of eligible women** from the listing above. Go to this column number.
- Follow the selected row and column to the cell where they meet and **circle the number** in the cell. This is the line number of the female to be selected for the interview (from table above).
- Write the name and line number of the selected female in the space below the table.

**LAST DIGIT OF THE ID NUMBER**	**TOTAL NUMBER OF ELIGIBLE WOMEN IN HOUSEHOLD**							
	**1**	**2**	**3**	**4**	**5**	**6**	**7**	**8**
**0**	1	2	2	4	3	6	5	4
**1**	1	1	3	1	4	1	6	5
**2**	1	2	1	2	5	2	7	6
**3**	1	1	2	3	1	3	1	7
**4**	1	2	3	4	2	4	2	8
**5**	1	1	1	1	3	5	3	1
**6**	1	2	2	2	4	6	4	2
**7**	1	1	3	3	5	1	5	3
**8**	1	2	1	4	1	2	6	4
**9**	1	1	2	1	2	3	7	5

NAME OF SELECTED WOMAN_____________________ LINE NUMBER OF SELECTED WOMAN [ ]

AGE VERIFICATION

Interviewer instructions:
Ask to speak with the selected girl/woman. Ask her age and date of birth and record:

Reported age: _ _ **Date of birth: _ _/_ _/_ _ _ _** **Calculated age: _ _**

- If reported or calculated age is 20 or younger:
– Ask to see written documentation. This could include birth certificate, clinic card, passport, religion card, ID card, or other official document.
– If documentation indicates she is aged of 15-17, continue with Parental Consent process. If documentation indicates she is aged 18+, continue with Adult Informed Consent.
– If no documentation is available to verify age, then remove the name of this person from the list of eligible female members and move up all other names by one row. Repeat the random selection process, and then age verification, as indicated by reported age.

[ ] Tick here if you had to repeat the random selection because of no age documents

- If reported or calculated age is age 21-49, continue with the Adult Informed Consent process.

If the selected girl/woman is not available, reschedule a visit. If her age on the roster of eligible females is 20 or younger, before you leave attempt to verify her age through written documentation with others in the household.

RESPONDENT QUESTIONNAIRE
**Introduction**
INTERVIEWER: As you have just learned, the overall purpose of this study is to measure the effect of a set of programs and services delivered in health facilities and in the community to improve the health and well-being of women and children. I am going to ask you a series of questions and then will record your answers on this form. The interview will take from one to one and one half hours. As we noted in the consent form, you may find some questions that I will ask to be sensitive. You do not have to respond to any question that makes you uncomfortable. You may end the interview at any time. We will only begin the interview once we have ensured we are in a private place and you are comfortable. If anyone enters the room, we will pause the interview and look for another private space, if necessary, to ensure your comfort in moving forward with the interview.<br><br>RECORD START TIME OF INTERVIEW: [ ] [ ]:[ ] [ ] (use 24 hour clock)
**Section 1 Respondent's background and her children**

QUESTIONS and FILTERS		CODING CATEGORIES	SKIP TO
INTERVIEWER: If you don't mind, I'd like to start by asking you about and you, your family, and your household.			
➢ Household characteristics			
001	What is the main source of drinking water for members of your household?	PIPED WATER . . . . 1<br>*PIPED INTO DWELLING, PIPED INTO YARD/PLOT, PUBLIC TAP, NEIGHBOUR'S TAP*<br>WATER FROM OPEN WELL. . . . . .2<br>*OPEN WELL IN DWELLING, OPEN WELL IN YARD/PLOT, OPEN PUBLIC WELL, NEIGHBOUR'S OPEN WELL*<br>WATER FROM COVERED WELL OR BOREHOLE . . . . 3<br>*PROTECTED WELL IN DWELLING, PROTECTED WELL IN YARD/PLOT, PROTECTED PUBLIC WELL, NEIGHBOUR'S BOREHOLE*<br>SURFACE WATER . . . . .4<br>*SPRING, RIVER/STREAM, POND/LAKE, DAM*<br>RAINWATER . . . . .5<br>TANKER TRUCK . . . . 6<br>WATER VENDOR . . . . 7<br>BOTTLED WATER . . . . .8<br>OTHER __________________________ 9	
002	What kind of toilet facility do members of your household usually use?	FLUSH/POUR FLUSH TO: PIPED SEWER SYSTEM, PIPED	

QUESTIONS and FILTERS		CODING CATEGORIES	SKIP TO
		SEPTIC TANK, PIPED PIT LATRINE, OR ELSEWHERE . . . . 1<br>PIT LATRINE . . . . 2<br>COMPOSTING TOILET/ECOSAN . . . . 3<br>BUCKET . . . . 4<br>NO FACILITY/BUSH/FIELD . . . . 5<br>OTHER . . . . .6<br>(SPECIFY) ____________________________	
003	Does your household have electricity?	YES . . . . 1<br>NO 2	
➢ Respondent's Age			
101	In which month and year were you born?	MONTH. . . . . [ ] [ ]<br>DON'T KNOW MONTH . . . . 98<br><br>YEAR . . . . [ ] [ ] [ ] [ ]<br>DON'T KNOW YEAR 9 9 9 8	
102	How old were you at your last birthday?<br><br>COMPARE AGE WITH Q101 AND CORRECT AGE IF NECESSARY	AGE IN COMPLETED YEARS. . . . . . . [ ] [ ]	
➢ Respondent's Literacy/Education			
103	Are you able to read and write?	YES . . . . 1<br>NO 2	
104	Have you ever attended school?	YES . . . . 1<br>NO 2	 ➢ 107
105	What is the highest level of school you attended?	PRE-PRIMARY/NURSERY . . . . 0<br>PRIMARY . . . . 1<br>POST-PRIMARY TRAINING . . . . 2<br>SECONDARY . . . . 3<br>POST-SECONDAY TRAINING . . . . 4<br>UNIVERSITY . . . . .5<br>DON'T KNOW 8	 <br><br><br><br><br>➢ 107
106	What is the highest grade/form/year that you completed at that level?<br>IF COMPLETED LESS THAN ONE YEAR AT THAT LEVEL, RECORD '00'	GRADE/FORM/YEAR. . . . . [ ] [ ]<br><br>DON'T KNOW. 98	
➢ Respondent's Work and Property			
107	As you know, some women take up jobs for which they are paid in cash or kind. Others sell things, have a small business or work on the family farm or	YES . . . . 1<br>NO 2	 ➢ 111

QUESTIONS and FILTERS		CODING CATEGORIES	SKIP TO
	in the family business. Aside from your own housework, have you done any of these things or any other work at any time during the past 12 months?		
108	What is your occupation-- that is, what kind of work did you mainly do in the past 12 months?	c) RECORD IN WORDS WHAT R SAYS:<br>______________________________<br><br>d) CODE LATER:<br>Professional/technical/managerial. . . . . . 1<br>Clerical. . . . . 2<br>Sales and services. . . . . 3<br>Skilled manual. . . . . 4<br>Unskilled manual .5<br>Domestic service. 6<br>Agriculture. . . . . 7<br>Student . . . . .8<br>Other 9	
109	Did you do this work throughout the year, or did you work seasonally, or only once in a while?	THROUGHOUT THE YEAR . . . . 1<br>SEASONALLY . . . . 2<br>ONCE IN A WHILE 3	
110	Were you paid in cash or in kind for this work, or were you not paid at all?<br><br>By “in kind” I mean when payment is made in goods, commodities or services -- instead of cash.	CASH ONLY . . . . 1<br>CASH AND IN KIND . . . . 2<br>IN KIND ONLY . . . . .3<br>NOT PAID 4	
111	Do you own this or any other house either alone or jointly with someone else?	OWNS ALONE . . . . 1<br>OWNS JOINTLY . . . . 2<br>DOES NOT OWN 3	
112	Do you own any land either alone or jointly with someone else?	OWNS ALONE . . . . 1<br>OWNS JOINTLY . . . . 2<br>DOES NOT OWN 3	
➢ Births and Children			
INTERVIEWER: Now I would like to ask about the births you have had during your life.			
113	c) Are you currently pregnant or have you been pregnant in the past 12 months?	YES. . . . . 1<br>NO . . . . .2<br>DON’T KNOW 8	
	d) Have you ever given birth?	YES . . . . 1<br>NO 2	 ➢ 120

QUESTIONS and FILTERS		CODING CATEGORIES		SKIP TO
114	Do you have any sons or daughters to whom you have given birth who are now living with you?	YES . . . . 1<br>NO 2		➢ 116
115	How many sons live with you?<br>And how many daughters live with you?<br><br>IF NONE, RECORD '00'.	c) SONS AT HOME . . . .<br>d) DAUGHTERS AT HOME	[ ][ ]<br>[ ][ ]	
116	Do you have any sons or daughters to whom you have given birth who are alive but do not live with you?	YES . . . . 1<br>NO 2		➢ 118
117	How many sons are alive but do not live with you?<br>And how many daughters are alive but do not live with you?<br>IF NONE, RECORD '00'.	c) SONS ELSEWHERE. . . . . . .<br>d) DAUGHTERS ELSEWHERE. .	[ ][ ]<br>[ ][ ]	
118	Have you ever given birth to a boy or girl who was born alive but later died?	YES . . . . 1<br>NO 2		➢ 120
119	How many children have died?	NUMBER OF CHILDREN WHO HAVE DIED<br>. . . . . [ ] [ ]<br>DON'T KNOW. 98		
➢ Partner Status: Current and in past 12 months				
120	What is your current marital status?	Married. . . . . .1<br>Living with a man as if married . . . . . . 2<br>Widowed. . . . . .3<br>Separated. . . . . 4<br>Divorced. . . . . 5<br>Never married/never lived with a man. 6		➢ 124<br>➢ 124<br><br><br><br>➢ 122
121	<u>At any time in the past 12 months</u>, were you married or living together with a man as if married?	Yes, married or living with a man as if married . . . . 1<br>No, not married or living with a man 2		
122	Do you currently have a steady boyfriend even if you did not live together or have sex?	YES . . . . 1<br>NO 2		➢ 124
123	<u>At any time in the past 12 months</u>, have you had a steady boyfriend even if you did not live together or have sex?	Yes, had boyfriend in past 12 mos. . . . . 1<br>No, did not have boyfriend in past 12 mos. . . . 2		
**SUMMARY**<br>**Interviewer instructions: Review responses to Q120-123 and code the following 2 variables. These variables will be used at the start of Section 3.**				
**124**	**Partner in past 12 months?**	YES. 1		

QUESTIONS and FILTERS		CODING CATEGORIES	SKIP TO
		If ANY of the following conditions are met:<br>Q120 = 1 or 2<br>Q121=1<br>Q122=1<br>Q123=1<br>NO . . . . 2<br>If NONE of the conditions above are met	
**125**	**Currently have a partner?**	YES. . . . . 1<br>If ANY of the following conditions are met:<br>Q120 = 1 or 2<br>Q122=1<br>NO . . . . 2<br>If NONE of the conditions above are met	

Section 2 Respondent's health, health behaviors, and sexual history

INTERVIEWER: Now I would like to ask you some questions about your health and some other important aspects of a woman's life relating to health.

QUESTIONS and FILTERS		CODING CATEGORIES			SKIP TO
➢ Health and use of health services					
201	In general, would you describe your overall health as excellent, good, fair, poor, or very poor?	EXCELLENT. . . . . 1<br>GOOD. . . . . 2<br>FAIR. . . . . 3<br>POOR. . . . . 4<br>VERY POOR. 5			
202	In the past 12 months, did you have, or feel, the need for health services for yourself?	YES. . . . . 1<br>NO. 2			
203	In the past 12 months, did you visit a dispensary, health center, or hospital to obtain health services for yourself?	YES. . . . . 1<br>NO. 2			➢ 205
204	In the past 12 months, did a health care provider ever ask you about your experiences with violence?	YES. . . . . 1<br>NO. . . . . 2<br>DON'T KNOW/DON'T REMEMBER. 8			
205	What types of HIV services are available to women in your community?<br><br>READ EACH RESPONSE BELOW AND MARK RESPONSE.	**Available**	**Not available**	**Don't Know**	
	i. HIV testing and counseling for herself	1	2	8	

QUESTIONS and FILTERS		CODING CATEGORIES			SKIP TO
	j. HIV counseling and testing for couples (i.e., with her partner)	1	2	8	
	k. PMTCT	1	2	8	
	l. Antiretroviral treatment	1	2	8	
	m. Other clinical services such as medical treatment for opportunistic infections or other conditions that result from having AIDS	1	2	8	
	n. HIV post-exposure prophylaxis (PEP)	1	2	8	
	o. Education/prevention information	1	2	8	
	p. Psychosocial support/support group	1	2	8	
206	I don't want to know the results, but have you ever been tested to see if you have the AIDS virus?	YES . . . . 1<br>NO . . . . 2<br>DON'T KNOW. 8			 ➢ 209<br>➢ 209
207	When was the last time you were tested?	Within the past 12 MONTHS . . . . 1<br>13 - 24 MONTHS AGO. . . . . 2<br>MORE 2 YEARS AGO. . . . . 3<br>Don't know 8			
208	Again, I don't want to know the results, but did you get the results of the test?	YES . . . . 1<br>NO . . . . 2<br>DON'T KNOW. 8			
INTERVIEWER: Now I am going to ask you some questions that you may find to be sensitive. Please remember that everything you tell me today will be kept confidential and you can skip any question you are not comfortable answering.					
➢ Alcohol and substance use					
209	In the past 12 months, did you ever drink alcohol?	YES . . . . 1<br>NO 2			 ➢ 212
210	How often did you drink to the point of being drunk: often, sometimes, only once, or never?<br><br>By drunk, I mean feeling high, or tipsy, or losing control of your senses.	OFTEN . . . . .1<br>SOMETIMES . . . . 2<br>ONCE IN PAST 12 MOS. . . . . 3<br>NEVER . . . . .4<br>DON'T KNOW. 8			
211	With whom did you usually drink?<br><br>[TICK ALL THAT THE RESPONDENT MENTIONS; DO NOT READ]	[ ] a. ALONE<br>[ ] b. WITH HUSBAND/PARTNER<br>[ ] c. WITH BOYFRIEND<br>[ ] d. WITH IN-LAWS<br>[ ] e. WITH OWN FAMILY MEMBERS<br>[ ] f. WITH FEMALE FRIENDS<br>[ ] g. WITH MALE FRIENDS<br>[ ] h. OTHER: SPECIFY ___________________			

QUESTIONS and FILTERS		CODING CATEGORIES	SKIP TO
212	In the past 12 months, did you ever smoke marijuana?	YES . . . . 1<br>NO 2	 ➢ 215
213	How often did you smoke marijuana to the point of feeling high: often, sometimes, only once or never?	OFTEN . . . . .1<br>SOMETIMES . . . . 2<br>ONCE IN PAST 12 MOS. . . . . 3<br>NEVER . . . . .4<br>DON'T KNOW. 8	
214	With whom did you usually smoke marijuana?<br><br>[TICK ALL THAT THE RESPONDENT MENTIONS; DO NOT READ]	[ ] a. ALONE<br>[ ] b. WITH HUSBAND/PARTNER<br>[ ] c. WITH BOYFRIEND<br>[ ] d. WITH IN-LAWS<br>[ ] e. WITH OWN FAMILY MEMBERS<br>[ ] f. WITH FEMALE FRIENDS<br>[ ] g. WITH MALE FRIENDS<br>[ ] h. OTHER: SPECIFY ___________________	
215	At any time in your life, as a child or as an adult, has anyone ever forced you in any way to have sexual intercourse or perform any other sexual acts?	YES . . . . 1<br>NO . . . . 2<br>DID NOT ANSWER. 9	
216	How old were you when you had sexual intercourse for the very first time?	AGE IN YEARS . . . . [ ] [ ]<br>DON'T KNOW . . . . 98<br>Never had sexual intercourse. 00	 <br>➢ GO TO FILTER BELOW
217	The first time you had sexual intercourse, would you say that you had it because you wanted to, or because you were forced to have it against your will?	WANTED TO. . . . . 1<br>FORCED TO . . . . 2<br>DID NOT ANSWER. 9	
218	In total, with how many different people have you had sexual intercourse with in the last 12 months?	Total number of sexual partners<br>in last 12 months [ ] [ ] [ ]<br>DON'T KNOW . . . . 998<br>DID NOT ANSWER. 999	

FILTER	
**INTERVIEWER FILTER:** INTIMATE PARTNER IN THE PAST 12 MONTHS? See Q124	
If Q124 = YES, CONTINUE WITH SECTION 3	IF Q124=NO, GO TO SECTION 5

Section 3 Intimate partner characteristics

INSTRUCTIONS TO INTERVIEWER:

THE QUESTIONS BELOW SHOULD BE ASKED FOR UP TO 3 INTIMATE PARTNERS THAT RESPONDENT (R) HAS HAD IN THE PAST 12 MONTHS.

BEGIN BY ASKING ABOUT CURRENT PARTNER, IF SHE HAS ONE (Q125=YES), or MOST RECENT PARTNER IF SHE CURRENTLY HAS NO PARTNER. THE RESPONDENT MAY HAVE CONCURRENT PARTNERS, e.g., CURRENTLY MARRIED AND A STEADY BOYFRIEND. WE HAVEN'T ASKED ABOUT THIS PREVIOUSLY, BUT THE FOLLOWING QUESTIONS ALLOW FOR IT. IN THIS CASE, YOU CAN REFER TO A PRIMARY PARTNER. HOWEVER, THE ORDER IN WHICH THE PARTNERS ARE ASKED ABOUT, DOESN'T MATTER.

ASK QUESTIONS VERTICALLY FROM Q301 THROUGH Q424 (SECTION 4).
THEN FOLLOW INSTRUCTIONS IN 424, BEGINNING AGAIN WITH Q301 IF R HAS HAD MORE THAN ONE INTIMATE PARTNER IN THE PAST 12 MONTHS.

INTERVIEWER: Next, I would like to ask you some questions about your current (or recent) male partner. Again, please let me assure you that your answers will be kept completely confidential and will not be told to anyone. If we should come to any question that you don't want to answer, just let me know and we will go on to the next question. You told me previously, that you currently have a partner/had a partner in the past year…

QUESTIONS and FILTERS		a) CURRENT, MOST RECENT, OR PRIMARY PARTNER	b) OTHER PARTNER (1)	c) OTHER PARTNER (2)
301	What is the relationship of this partner to you?	HUSBAND. . . . . 1<br>LIVE-IN PARTNER. . .2<br>EX-HUSBAND . . . . . .3<br>EX-LIVE IN PARTNER . . . . 4<br>STEADY BOYFRIEND (NOT LIVING WITH RESPONDENT). . . . . 5<br>EX-STEADY BOYFRIEND 6	HUSBAND. . . . . 1<br>LIVE-IN PARTNER. . .2<br>EX-HUSBAND . . . . . .3<br>EX-LIVE IN PARTNER . . . . 4<br>STEADY BOYFRIEND (NOT LIVING WITH RESPONDENT). . . . . 5<br>EX-STEADY BOYFRIEND 6	HUSBAND. . . . . 1<br>LIVE-IN PARTNER. . .2<br>EX-HUSBAND . . . . . 3<br>EX-LIVE IN PARTNER . . . . 4<br>STEADY BOYFRIEND (NOT LIVING WITH RESPONDENT). . . . . 5<br>EX-STEADY BOYFRIEND 6
302	Approximately how old is/was this partner compared to you?	YOUNGER THAN YOU ARE . . . . 1<br>SAME AGE AS YOU ARE . . . . .2<br>1-9 YEARS OLDER . . . 3<br>10+ YEARS OLDER. . . 4<br><br>DON'T KNOW 8	YOUNGER THAN YOU ARE . . . . 1<br>SAME AGE AS YOU ARE . . . . .2<br>1-9 YEARS OLDER . . . 3<br>10+ YEARS OLDER. . . 4<br><br>DON'T KNOW 8	YOUNGER THAN YOU ARE . . . . 1<br>SAME AGE AS YOU ARE . . . . .2<br>1-9 YEARS OLDER . . . 3<br>10+ YEARS OLDER. . . 4<br><br>DON'T KNOW 8
303	Did he ever attend school?	YES . . . . 1<br>NO . . . . 2 / DON'T KNOW. .8 — Go to 306	YES . . . . 1<br>NO . . . . 2 / DON'T KNOW. .8 — Go to 306	YES . . . . 1<br>NO . . . . 2 / DON'T KNOW. .8 — Go to 306
304	What is the highest level of school he attended?	PRE-PRIMARY/ NURSERY . . . . 0<br>PRIMARY . . . . 1<br>POST-PRIMARY TRAINING . . . . 2<br>SECONDARY . . . . . . 3<br>POST-SECONDAY TRAINING . . . . .4<br>UNIVERSITY . . . . 5<br>DON'T KNOW . . . . . . 8<br>[GO TO 306]	PRE-PRIMARY/ NURSERY . . . . 0<br>PRIMARY . . . . 1<br>POST-PRIMARY TRAINING . . . . 2<br>SECONDARY . . . . . . 3<br>POST-SECONDAY TRAINING . . . . .4<br>UNIVERSITY . . . . 5<br>DON'T KNOW . . . . . . 8<br>[GO TO 306]	PRE-PRIMARY/ NURSERY . . . . 0<br>PRIMARY . . . . 1<br>POST-PRIMARY TRAINING . . . . 2<br>SECONDARY . . . . . . 3<br>POST-SECONDAY TRAINING . . . . .4<br>UNIVERSITY . . . . 5<br>DON'T KNOW . . . . . . 8<br>[GO TO 306]
305	What is the highest grade/form/year he completed at that level?<br><br>IF COMPLETED LESS THAN ONE YEAR AT THAT LEVEL, RECORD '00'	GRADE/FORM/YEAR . . . . [ ] [ ]<br><br>DON'T KNOW 98	GRADE/FORM/YEAR . . . . [ ] [ ]<br><br>DON'T KNOW 98	GRADE/FORM/YEAR . . . . [ ] [ ]<br><br>DON'T KNOW 98

QUESTIONS and FILTERS		a) CURRENT, MOST RECENT, OR PRIMARY PARTNER	b) OTHER PARTNER (1)	c) OTHER PARTNER (2)
306	What is/was his occupation? That is, what kind of work does/did he mainly do?	3) RECORD IN WORDS WHAT R SAYS:<br>______________________<br><br>4) CODELATER:<br>NOT WORKING . . . . .0<br>Professional/technical/ managerial. . . . . . 1<br>Clerical. . . . . .2<br>Sales and services . . . 3<br>Skilled manual. . . . . 4<br>Unskilled manual . . . . 5<br>Domestic service. . . . 6<br>Agriculture. . . . . 7<br>Student . . . . 8<br>R doesn't know 9	3) RECORD IN WORDS WHAT R SAYS:<br>______________________<br><br>4) CODE:<br>NOT WORKING . . . . .0<br>Professional/technical/ managerial. . . . . . 1<br>Clerical. . . . . .2<br>Sales and services . . . 3<br>Skilled manual. . . . . 4<br>Unskilled manual . . . . 5<br>Domestic service. . . . 6<br>Agriculture. . . . . 7<br>Student . . . . 8<br>R doesn't know 9	3) RECORD IN WORDS WHAT R SAYS:<br>______________________<br><br>4) CODE:<br>NOT WORKING . . . . .0<br>Professional/technical/ managerial. . . . . . 1<br>Clerical. . . . . .2<br>Sales and services . . . 3<br>Skilled manual. . . . . 4<br>Unskilled manual . . . . 5<br>Domestic service. . . . 6<br>Agriculture. . . . . 7<br>Student . . . . 8<br>R doesn't know 9
307	Does/did he have any other partners, a wife or wives, or does/did he live with another woman/other women as if married?	YES . . . . 1<br>NO . . . . 2<br>DON'T KNOW 8	YES . . . . 1<br>NO . . . . 2<br>DON'T KNOW 8	YES . . . . 1<br>NO . . . . 2<br>DON'T KNOW 8
308	In the past year, did your partner ever drink alcohol?	YES . . . . 1<br>NO. . . . . 2 / DON'T KNOW.8 — GO TO 311	YES . . . . 1<br>NO. . . . . 2 / DON'T KNOW.8 — GO TO 311	YES . . . . 1<br>NO. . . . . 2 / DON'T KNOW. 8 — GO TO 311
309	How often did he drink to the point of being drunk: often, sometimes, only once, or never?<br><br>By drunk, I mean feeling high, or tipsy, or losing control of your senses.	OFTEN . . . . .1<br>SOMETIMES . . . . 2<br>ONCE in past 12 mos. 3<br>NEVER . . . . .4<br><br>DON'T KNOW. 8	OFTEN . . . . .1<br>SOMETIMES . . . . 2<br>ONCE in past 12 mos. 3<br>NEVER . . . . .4<br><br>DON'T KNOW. 8	OFTEN . . . . .1<br>SOMETIMES . . . . 2<br>ONCE in past 12 mos. 3<br>NEVER . . . . .4<br><br>DON'T KNOW. 8

QUESTIONS and FILTERS		a) CURRENT, MOST RECENT, OR PRIMARY PARTNER		b) OTHER PARTNER (1)		c) OTHER PARTNER (2)	
310	With whom did he usually drink?<br><br>[TICK ALL THAT THE RESPONDENT MENTIONS; DO NOT READ]	[ ] a. ALONE<br>[ ] b. WITH RESPONDENT<br>[ ] c. WITH GIRLFRIEND<br>[ ] d. WITH IN-LAWS<br>[ ] e. WITH OWN FAMILY MEMBERS<br>[ ] f. WITH FEMALE FRIENDS<br>[ ] g. WITH MALE FRIENDS<br>[ ] h. OTHER: SPECIFY<br>___________________		[ ] a. ALONE<br>[ ] b. WITH RESPONDENT<br>[ ] c. WITH GIRLFRIEND<br>[ ] d. WITH IN-LAWS<br>[ ] e. WITH OWN FAMILY MEMBERS<br>[ ] f. WITH FEMALE FRIENDS<br>[ ] g. WITH MALE FRIENDS<br>[ ] h. OTHER: SPECIFY<br>___________________		[ ] a. ALONE<br>[ ] b. WITH RESPONDENT<br>[ ] c. WITH GIRLFRIEND<br>[ ] d. WITH IN-LAWS<br>[ ] e. WITH OWN FAMILY MEMBERS<br>[ ] f. WITH FEMALE FRIENDS<br>[ ] g. WITH MALE FRIENDS<br>[ ] h. OTHER: SPECIFY<br>___________________	
311	In the past 12 months, did your partner ever smoke marijuana?	YES 1		YES 1		YES 1	
		NO. . . . . 2<br>DON'T KNOW.8	GO TO 314	NO. . . . . 2<br>DON'T KNOW.8	GO TO 314	NO. . . . . 2<br>DON'T KNOW. 8	GO TO 314
312	How often did he smoke marijuana to the point of feeling high: often, sometimes, only once or never?	OFTEN . . . . .1<br>SOMETIMES . . . . 2<br>ONCE . . . . 3<br>NEVER . . . . 4<br>DON'T KNOW. 8		OFTEN . . . . .1<br>SOMETIMES . . . . 2<br>ONCE . . . . 3<br>NEVER . . . . 4<br>DON'T KNOW. 8		OFTEN . . . . .1<br>SOMETIMES . . . . 2<br>ONCE . . . . 3<br>NEVER . . . . 4<br>DON'T KNOW. 8	
313	With whom did he usually smokemarijuana?<br><br>[RECORD ALL THAT THE RESPONDENT MENTIONS; DO NOT READ]	[ ] a. ALONE<br>[ ] b. WITH RESPONDENT<br>[ ] c. WITH GIRLFRIEND<br>[ ] d. WITH IN-LAWS<br>[ ] e. WITH OWN FAMILY MEMBERS<br>[ ] f. WITH FEMALE FRIENDS<br>[ ] g. WITH MALE FRIENDS<br>[ ] h. OTHER: SPECIFY<br>___________________		[ ] a. ALONE<br>[ ] b. WITH RESPONDENT<br>[ ] c. WITH GIRLFRIEND<br>[ ] d. WITH IN-LAWS<br>[ ] e. WITH OWN FAMILY MEMBERS<br>[ ] f. WITH FEMALE FRIENDS<br>[ ] g. WITH MALE FRIENDS<br>[ ] h. OTHER: SPECIFY<br>___________________		[ ] a. ALONE<br>[ ] b. WITH RESPONDENT<br>[ ] c. WITH GIRLFRIEND<br>[ ] d. WITH IN-LAWS<br>[ ] e. WITH OWN FAMILY MEMBERS<br>[ ] f. WITH FEMALE FRIENDS<br>[ ] g. WITH MALE FRIENDS<br>[ ] h. OTHER: SPECIFY<br>___________________	
314	Have you had sexual intercourse with this partner?	YES, in past 12 mos . . . . 1<br><br>Yes, but more than 12 mos ago . . . . 2<br>GO TO SECTION 4<br><br>No, never had sex with this partner. . . . . 3<br>GO TO Section 4		YES, in past 12 mos . . . . 1<br><br>Yes, but more than 12 mos ago . . . . 2<br>GO TO Section 4<br><br>No, never had sex with this partner. . . . . 3<br>GO TO Section 4		YES, in past 12 mos . . . . 1<br><br>Yes, but more than 12 mos ago . . . . 2<br>GO TO Section 4<br><br>No, never had sex with this partner. . . . . 3<br>GO TO Section 4	
315	Approximately how often did you have sexual	OFTEN<br>(2-3 times/week) 1		OFTEN<br>(2-3 times/week) 1		OFTEN<br>(2-3 times/week) 1	

QUESTIONS and FILTERS		a) CURRENT, MOST RECENT, OR PRIMARY PARTNER		b) OTHER PARTNER (1)		c) OTHER PARTNER (2)	
	intercourse with this person <u>in the last 12 months</u>?<br><br>READ CATEGORIES	SOMETIMES (few times/mo). . . . . 2<br>RARELY. . . . . .3<br>ONCE. . . . . 4<br>DON'T KNOW. 8		SOMETIMES (few times/mo). . . . . 2<br>RARELY. . . . . .3<br>ONCE. . . . . 4<br>DON'T KNOW. 8		SOMETIMES (few times/mo). . . . . 2<br>RARELY. . . . . .3<br>ONCE. . . . . 4<br>DON'T KNOW. 8	
316	The last time you had intercourse with this person, was a condom used?	YES. 1		YES. 1		YES. 1	
		NO. . . . . . . 2<br>DON'T KNOW . . . 8	GO TO SECTION 4	NO. . . . . . . 2<br>DON'T KNOW . . . 8	GO TO SECTION 4	NO. . . . . . . 2<br>DON'T KNOW . . . 8	GO TO SECTION 4
317	<u>In the past 12 months</u>, did you use a condom every time you had sexual intercourse with this person?	YES. . . . . 1<br>NO. . . . . 2<br>DON'T KNOW 8		YES. . . . . 1<br>NO. . . . . 2<br>DON'T KNOW 8		YES. . . . . 1<br>NO. . . . . 2<br>DON'T KNOW 8	

Section 4 IPV in the past 12 months

INTERVIEWER: Read this only the first time/for first partner. I am now going to ask you about some situations that happen to some women. I'd like you to think back over the past 12 months and please tell me if they happened to you. You may find some of these questions to be sensitive. Please remember if we should come to any question that you do not want to answer, just let me know and we will go on to the next question. I want to remind you that if you prefer, we can stop the interview at any time.

QUESTIONS and FILTERS		a) CURRENT, MOST RECENT, OR PRIMARY PARTNER	b) OTHER PARTNER (1)	c) OTHER PARTNER (2)
**INTERVIEWER**: So continuing with the partner we were just discussing, please let me know how often, if at all, this happened to you in the past year.				
➢ Controlling partner behavior				
401	In the last 12 months, was your partner jealous or angry if you talked to other men—often, sometimes, just once, or not at all?	OFTEN. . . . . 1<br>SOMETIMES. . . . . . . 2<br>ONCE. . . . . 3<br>NEVER. . . . . 4<br>DON'T KNOW . . . . . 8<br>NO ANSWER 9	OFTEN. . . . . 1<br>SOMETIMES. . . . . . . 2<br>ONCE. . . . . 3<br>NEVER. . . . . 4<br>DON'T KNOW . . . . . 8<br>NO ANSWER 9	OFTEN. . . . . 1<br>SOMETIMES. . . . . . . 2<br>ONCE. . . . . 3<br>NEVER. . . . . 4<br>DON'T KNOW . . . . . 8<br>NO ANSWER 9
402	Did he accuse you of being unfaithful?	OFTEN. . . . . 1<br>SOMETIMES. . . . . . . 2<br>ONCE. . . . . 3<br>NEVER. . . . . 4<br>DON'T KNOW . . . . . 8<br>NO ANSWER 9	OFTEN. . . . . 1<br>SOMETIMES. . . . . . . 2<br>ONCE. . . . . 3<br>NEVER. . . . . 4<br>DON'T KNOW . . . . . 8<br>NO ANSWER 9	OFTEN. . . . . 1<br>SOMETIMES. . . . . . . 2<br>ONCE. . . . . 3<br>NEVER. . . . . 4<br>DON'T KNOW . . . . . 8<br>NO ANSWER 9
403	Did he try to isolate you from meeting your female friends?	OFTEN. . . . . 1<br>SOMETIMES. . . . . . . 2<br>ONCE. . . . . 3<br>NEVER. . . . . 4<br>DON'T KNOW . . . . . 8<br>NO ANSWER 9	OFTEN. . . . . 1<br>SOMETIMES. . . . . . . 2<br>ONCE. . . . . 3<br>NEVER. . . . . 4<br>DON'T KNOW . . . . . 8<br>NO ANSWER 9	OFTEN. . . . . 1<br>SOMETIMES. . . . . . . 2<br>ONCE. . . . . 3<br>NEVER. . . . . 4<br>DON'T KNOW . . . . . 8<br>NO ANSWER 9
404	Did he try to limit your contact with your family?	OFTEN. . . . . 1<br>SOMETIMES. . . . . . . 2<br>ONCE. . . . . 3<br>NEVER. . . . . 4<br>DON'T KNOW . . . . . 8<br>NO ANSWER 9	OFTEN. . . . . 1<br>SOMETIMES. . . . . . . 2<br>ONCE. . . . . 3<br>NEVER. . . . . 4<br>DON'T KNOW . . . . . 8<br>NO ANSWER 9	OFTEN. . . . . 1<br>SOMETIMES. . . . . . . 2<br>ONCE. . . . . 3<br>NEVER. . . . . 4<br>DON'T KNOW . . . . . 8<br>NO ANSWER 9
405	Did he generally insist on knowing where you were at all times?	OFTEN. . . . . 1<br>SOMETIMES. . . . . . . 2<br>ONCE. . . . . 3<br>NEVER. . . . . 4<br>DON'T KNOW . . . . . 8<br>NO ANSWER 9	OFTEN. . . . . 1<br>SOMETIMES. . . . . . . 2<br>ONCE. . . . . 3<br>NEVER. . . . . 4<br>DON'T KNOW . . . . . 8<br>NO ANSWER 9	OFTEN. . . . . 1<br>SOMETIMES. . . . . . . 2<br>ONCE. . . . . 3<br>NEVER. . . . . 4<br>DON'T KNOW . . . . . 8<br>NO ANSWER 9
406	Did he try to control your use of money?	OFTEN. . . . . 1<br>SOMETIMES. . . . . . . 2<br>ONCE. . . . . 3<br>NEVER. 4	OFTEN. . . . . 1<br>SOMETIMES. . . . . . . 2<br>ONCE. . . . . 3<br>NEVER. 4	OFTEN. . . . . 1<br>SOMETIMES. . . . . . . 2<br>ONCE. . . . . 3<br>NEVER. 4

QUESTIONS and FILTERS		a) CURRENT, MOST RECENT, OR PRIMARY PARTNER	b) OTHER PARTNER (1)	c) OTHER PARTNER (2)
		DON'T KNOW . . . . . 8<br>NO ANSWER 9	DON'T KNOW . . . . . 8<br>NO ANSWER 9	DON'T KNOW . . . . . 8<br>NO ANSWER 9
407	Would you say that the money you earned in the past 12 months was more than what your partner earned, less than what he earned, or about the same?	MORE THAN HE EARNED. . . . . 1<br>LESS THAN HE EARNED. . . . . 2<br>ABOUT THE SAME . . 3<br>DON'T KNOW 8	MORE THAN HE EARNED. . . . . 1<br>LESS THAN HE EARNED. . . . . 2<br>ABOUT THE SAME . . 3<br>DON'T KNOW 8	MORE THAN HE EARNED. . . . . 1<br>LESS THAN HE EARNED. . . . . 2<br>ABOUT THE SAME . . 3<br>DON'T KNOW 8
➢ Experience of Emotional violence				
408	**In the last 12 months**:<br>Did he say or do anything to humiliate you in front of others?	OFTEN. . . . . 1<br>SOMETIMES. . . . . . . 2<br>ONCE. . . . . 3<br>NEVER. . . . . 4<br>DON'T KNOW . . . . . 8<br>NO ANSWER 9	OFTEN. . . . . 1<br>SOMETIMES. . . . . . . 2<br>ONCE. . . . . 3<br>NEVER. . . . . 4<br>DON'T KNOW . . . . . 8<br>NO ANSWER 9	OFTEN. . . . . 1<br>SOMETIMES. . . . . . . 2<br>ONCE. . . . . 3<br>NEVER. . . . . 4<br>DON'T KNOW . . . . . 8<br>NO ANSWER 9
409	Did he threaten to hurt or harm you or someone close to you?	OFTEN. . . . . 1<br>SOMETIMES. . . . . . . 2<br>ONCE. . . . . 3<br>NEVER. . . . . 4<br>DON'T KNOW . . . . . 8<br>NO ANSWER 9	OFTEN. . . . . 1<br>SOMETIMES. . . . . . . 2<br>ONCE. . . . . 3<br>NEVER. . . . . 4<br>DON'T KNOW . . . . . 8<br>NO ANSWER 9	OFTEN. . . . . 1<br>SOMETIMES. . . . . . . 2<br>ONCE. . . . . 3<br>NEVER. . . . . 4<br>DON'T KNOW . . . . . 8<br>NO ANSWER 9
410	Did he insult you or make you feel bad about yourself?	OFTEN. . . . . 1<br>SOMETIMES. . . . . . . 2<br>ONCE. . . . . 3<br>NEVER. . . . . 4<br>DON'T KNOW . . . . . 8<br>NO ANSWER 9	OFTEN. . . . . 1<br>SOMETIMES. . . . . . . 2<br>ONCE. . . . . 3<br>NEVER. . . . . 4<br>DON'T KNOW . . . . . 8<br>NO ANSWER 9	OFTEN. . . . . 1<br>SOMETIMES. . . . . . . 2<br>ONCE. . . . . 3<br>NEVER. . . . . 4<br>DON'T KNOW . . . . . 8<br>NO ANSWER 9
➢ Experience of Physical violence				
411	Did he push you, shake you, or throw something at you?	OFTEN. . . . . 1<br>SOMETIMES. . . . . . . 2<br>ONCE. . . . . 3<br>NEVER. . . . . 4<br>DON'T KNOW . . . . . 8<br>NO ANSWER 9	OFTEN. . . . . 1<br>SOMETIMES. . . . . . . 2<br>ONCE. . . . . 3<br>NEVER. . . . . 4<br>DON'T KNOW . . . . . 8<br>NO ANSWER 9	OFTEN. . . . . 1<br>SOMETIMES. . . . . . . 2<br>ONCE. . . . . 3<br>NEVER. . . . . 4<br>DON'T KNOW . . . . . 8<br>NO ANSWER 9
412	Did he Slap you?	OFTEN. . . . . 1<br>SOMETIMES. . . . . . . 2<br>ONCE. . . . . 3<br>NEVER. . . . . 4<br>DON'T KNOW . . . . . 8<br>NO ANSWER 9	OFTEN. . . . . 1<br>SOMETIMES. . . . . . . 2<br>ONCE. . . . . 3<br>NEVER. . . . . 4<br>DON'T KNOW . . . . . 8<br>NO ANSWER 9	OFTEN. . . . . 1<br>SOMETIMES. . . . . . . 2<br>ONCE. . . . . 3<br>NEVER. . . . . 4<br>DON'T KNOW . . . . . 8<br>NO ANSWER 9

QUESTIONS and FILTERS		a) CURRENT, MOST RECENT, OR PRIMARY PARTNER	b) OTHER PARTNER (1)	c) OTHER PARTNER (2)
413	Did he Twist your arm or pull your hair?	OFTEN. . . . . 1<br>SOMETIMES. . . . . . . 2<br>ONCE. . . . . 3<br>NEVER. . . . . 4<br>DON'T KNOW . . . . . 8<br>NO ANSWER 9	OFTEN. . . . . 1<br>SOMETIMES. . . . . . . 2<br>ONCE. . . . . 3<br>NEVER. . . . . 4<br>DON'T KNOW . . . . . 8<br>NO ANSWER 9	OFTEN. . . . . 1<br>SOMETIMES. . . . . . . 2<br>ONCE. . . . . 3<br>NEVER. . . . . 4<br>DON'T KNOW . . . . . 8<br>NO ANSWER 9
414	Did he punch you with his fist or with something that could hurt you?	OFTEN. . . . . 1<br>SOMETIMES. . . . . . . 2<br>ONCE. . . . . 3<br>NEVER. . . . . 4<br>DON'T KNOW . . . . . 8<br>NO ANSWER 9	OFTEN. . . . . 1<br>SOMETIMES. . . . . . . 2<br>ONCE. . . . . 3<br>NEVER. . . . . 4<br>DON'T KNOW . . . . . 8<br>NO ANSWER 9	OFTEN. . . . . 1<br>SOMETIMES. . . . . . . 2<br>ONCE. . . . . 3<br>NEVER. . . . . 4<br>DON'T KNOW . . . . . 8<br>NO ANSWER 9
415	Did he kick you, drag you or beat you up?	OFTEN. . . . . 1<br>SOMETIMES. . . . . . . 2<br>ONCE. . . . . 3<br>NEVER. . . . . 4<br>DON'T KNOW . . . . . 8<br>NO ANSWER 9	OFTEN. . . . . 1<br>SOMETIMES. . . . . . . 2<br>ONCE. . . . . 3<br>NEVER. . . . . 4<br>DON'T KNOW . . . . . 8<br>NO ANSWER 9	OFTEN. . . . . 1<br>SOMETIMES. . . . . . . 2<br>ONCE. . . . . 3<br>NEVER. . . . . 4<br>DON'T KNOW . . . . . 8<br>NO ANSWER 9
416	Did he try to choke you or burn you on purpose?	OFTEN. . . . . 1<br>SOMETIMES. . . . . . . 2<br>ONCE. . . . . 3<br>NEVER. . . . . 4<br>DON'T KNOW . . . . . 8<br>NO ANSWER 9	OFTEN. . . . . 1<br>SOMETIMES. . . . . . . 2<br>ONCE. . . . . 3<br>NEVER. . . . . 4<br>DON'T KNOW . . . . . 8<br>NO ANSWER 9	OFTEN. . . . . 1<br>SOMETIMES. . . . . . . 2<br>ONCE. . . . . 3<br>NEVER. . . . . 4<br>DON'T KNOW . . . . . 8<br>NO ANSWER 9
417	Did he threaten or attack you with a knife, gun, or any other weapon?	OFTEN. . . . . 1<br>SOMETIMES. . . . . . . 2<br>ONCE. . . . . 3<br>NEVER. . . . . 4<br>DON'T KNOW . . . . . 8<br>NO ANSWER 9	OFTEN. . . . . 1<br>SOMETIMES. . . . . . . 2<br>ONCE. . . . . 3<br>NEVER. . . . . 4<br>DON'T KNOW . . . . . 8<br>NO ANSWER 9	OFTEN. . . . . 1<br>SOMETIMES. . . . . . . 2<br>ONCE. . . . . 3<br>NEVER. . . . . 4<br>DON'T KNOW . . . . . 8<br>NO ANSWER 9
➢ Experience of Physical sexual violence				
418	Did he physically force you to have sexual intercourse with him even when you did not want to?	OFTEN. . . . . 1<br>SOMETIMES. . . . . . . 2<br>ONCE. . . . . 3<br>NEVER. . . . . 4<br>DON'T KNOW . . . . . 8<br>NO ANSWER 9	OFTEN. . . . . 1<br>SOMETIMES. . . . . . . 2<br>ONCE. . . . . 3<br>NEVER. . . . . 4<br>DON'T KNOW . . . . . 8<br>NO ANSWER 9	OFTEN. . . . . 1<br>SOMETIMES. . . . . . . 2<br>ONCE. . . . . 3<br>NEVER. . . . . 4<br>DON'T KNOW . . . . . 8<br>NO ANSWER 9
419	Did he force you to do anything sexual that you did not want to?	OFTEN. . . . . 1<br>SOMETIMES. . . . . . . 2<br>ONCE. . . . . 3<br>NEVER. . . . . 4<br>DON'T KNOW . . . . . 8<br>NO ANSWER 9	OFTEN. . . . . 1<br>SOMETIMES. . . . . . . 2<br>ONCE. . . . . 3<br>NEVER. . . . . 4<br>DON'T KNOW . . . . . 8<br>NO ANSWER 9	OFTEN. . . . . 1<br>SOMETIMES. . . . . . . 2<br>ONCE. . . . . 3<br>NEVER. . . . . 4<br>DON'T KNOW . . . . . 8<br>NO ANSWER 9

QUESTIONS and FILTERS		a) CURRENT, MOST RECENT, OR PRIMARY PARTNER	b) OTHER PARTNER (1)	c) OTHER PARTNER (2)
➢ Experience of severe Physical violence				
**INTERVIEWER**: Did the following ever happen in the past 12 months as a result of what this partner did to you?				
420	You had cuts, bruises, or aches?	OFTEN. . . . . 1<br>SOMETIMES. . . . . . . 2<br>ONCE. . . . . 3<br>NEVER. . . . . 4<br>DON'T KNOW . . . . . 8<br>NO ANSWER 9	OFTEN. . . . . 1<br>SOMETIMES. . . . . . . 2<br>ONCE. . . . . 3<br>NEVER. . . . . 4<br>DON'T KNOW . . . . . 8<br>NO ANSWER 9	OFTEN. . . . . 1<br>SOMETIMES. . . . . . . 2<br>ONCE. . . . . 3<br>NEVER. . . . . 4<br>DON'T KNOW . . . . . 8<br>NO ANSWER 9
421	You had eye injuries, sprains, dislocations, or burns?	OFTEN. . . . . 1<br>SOMETIMES. . . . . . . 2<br>ONCE. . . . . 3<br>NEVER. . . . . 4<br>DON'T KNOW . . . . . 8<br>NO ANSWER 9	OFTEN. . . . . 1<br>SOMETIMES. . . . . . . 2<br>ONCE. . . . . 3<br>NEVER. . . . . 4<br>DON'T KNOW . . . . . 8<br>NO ANSWER 9	OFTEN. . . . . 1<br>SOMETIMES. . . . . . . 2<br>ONCE. . . . . 3<br>NEVER. . . . . 4<br>DON'T KNOW . . . . . 8<br>NO ANSWER 9
422	You had deep wounds, broken bones, broken teeth, or any other serious injury?	OFTEN. . . . . 1<br>SOMETIMES. . . . . . . 2<br>ONCE. . . . . 3<br>NEVER. . . . . 4<br>DON'T KNOW . . . . . 8<br>NO ANSWER 9	OFTEN. . . . . 1<br>SOMETIMES. . . . . . . 2<br>ONCE. . . . . 3<br>NEVER. . . . . 4<br>DON'T KNOW . . . . . 8<br>NO ANSWER 9	OFTEN. . . . . 1<br>SOMETIMES. . . . . . . 2<br>ONCE. . . . . 3<br>NEVER. . . . . 4<br>DON'T KNOW . . . . . 8<br>NO ANSWER 9
➢ Perpetration of Physical violence				
423	In the past 12 months, did you ever hit, slap, kick, or do anything else to physically hurt your partner at times when he was not already beating or physically hurting you?	OFTEN. . . . . 1<br>SOMETIMES. . . . . . . 2<br>ONCE. . . . . 3<br>NEVER. . . . . 4<br>DON'T KNOW . . . . . 8<br>NO ANSWER 9	OFTEN. . . . . 1<br>SOMETIMES. . . . . . . 2<br>ONCE. . . . . 3<br>NEVER. . . . . 4<br>DON'T KNOW . . . . . 8<br>NO ANSWER 9	OFTEN. . . . . 1<br>SOMETIMES. . . . . . . 2<br>ONCE. . . . . 3<br>NEVER. . . . . 4<br>DON'T KNOW . . . . . 8<br>NO ANSWER 9
**FILTER:** Other partners in past 12 months?				
424	Apart from (this person, these two people) we have just discussed, in the past 12 months did you have any other male partners (i.e., husband, live-in partner, ex-husband, ex-live in partner, boyfriend, ex-boyfriend)?	YES . . . . .1<br>GO BACK TO 301 and ask Q301 through Q424 for second partner; RECORD RESPONSES IN COLUMN 2<br><br>No 2	YES . . . . .1<br>GO BACK TO 301 and ask Q301 through Q424 for third partner; RECORD RESPONSES IN COLUMN 3<br><br>No 2	GO TO SECTION 5

Section 5 Physical and sexual violence from others in past 12 months

INTERVIEWER: I would now like to ask you about types of violence that you may have experienced from others, (IF SECTIONS 3 AND 4 WERE ASKED, THEN ADD: besides the partners we have just been discussing).

QUESTIONS and FILTERS		CODING CATEGORIES	SKIP TO
➢ Physical violence			
501	In the past 12 months, has anyone [other than the partner/partners we have just talked about (husband, live-in partner, steady boyfriend)] hit, slapped, kicked or done anything else to hurt you physically?	YES. . . . . 1<br>NO . . . . 2<br>NO ANSWER 9	 ➢ 503<br>➢ 503
502	Who has hurt you in this way? DO NOT READ CATEGORIES, BUT TICK ALL THAT R MENTIONS; USE ONLY ONE CATEGORY FOR EACH PERSON MENTIONED.<br>THEN ASK ABOUT FREQUENCY FOR THOSE TICKED:<br>Did this happen often, sometimes or only once in the past 12 months?		
	a. [ ] MEMBER OF OWN FAMILY BY BIRTH	OFTEN. . . . . 1<br>SOMETIMES. . . . . . . 2<br>ONCE. 3	
	b. [ ] IN-LAW/MEMBER OF PARTNER'S FAMILY	OFTEN. . . . . 1<br>SOMETIMES. . . . . . . 2<br>ONCE. 3	
	c. [ ] FORMER HUSBAND/LIVE-IN PARTNER R HAD MORE THAN 12 MOS AGO	OFTEN. . . . . 1<br>SOMETIMES. . . . . . . 2<br>ONCE. 3	
	d. [ ] FORMER BOYFRIEND R HAD MORE THAN 12 MOS AGO	OFTEN. . . . . 1<br>SOMETIMES. . . . . . . 2<br>ONCE. 3	
	e. [ ] TEACHER	OFTEN. . . . . 1<br>SOMETIMES. . . . . . . 2<br>ONCE. 3	
	f. [ ] EMPLOYER/SOMEONE AT WORK	OFTEN. . . . . 1<br>SOMETIMES. . . . . . . 2<br>ONCE. 3	
	g. [ ] POLICE/SOLDIER	OFTEN. . . . . 1<br>SOMETIMES. . . . . . . 2<br>ONCE. 3	
	h. [ ] NEIGHBOR/SOMEONE IN COMMUNITY	OFTEN. . . . . 1<br>SOMETIMES. . . . . . . 2<br>ONCE. 3	
	i. [ ] STRANGER	OFTEN. . . . . 1<br>SOMETIMES. . . . . . . 2<br>ONCE. 3	
	j. [ ] OTHER- SPECIFY_____________	OFTEN. . . . . 1<br>SOMETIMES. . . . . . . 2<br>ONCE. 3	
➢ Forced sexual intercourse			

QUESTIONS and FILTERS		CODING CATEGORIES	SKIP TO
503	In the past 12 months, has anyone other than the partners we have already talked about [husband, live-in partner, steady boyfriend] forced you to have sexual intercourse against your will?	YES. . . . . 1<br>NO . . . . 2<br>NO ANSWER 9	 ➢505<br>➢505
504	Who has forced you to have sexual intercourse? DO NOT READ CATEGORIES, BUT TICK ALL THAT R MENTIONS; USE ONLY ONE CATEGORY FOR EACH PERSON MENTIONED.<br><br>THEN ASK ABOUT FREQUENCY FOR THOSE TICKED:<br>Did this happen often, sometimes or only once in the past 12 months?		
	a. [ ] FATHER	OFTEN. . . . . 1<br>SOMETIMES. . . . . . . 2<br>ONCE. 3	
	b. [ ] OTHER BIRTH RELATIVE	OFTEN. . . . . 1<br>SOMETIMES. . . . . . . 2<br>ONCE. 3	
	c. [ ] STEP-FATHER	OFTEN. . . . . 1<br>SOMETIMES. . . . . . . 2<br>ONCE. 3	
	d. [ ] INLAW	OFTEN. . . . . 1<br>SOMETIMES. . . . . . . 2<br>ONCE. 3	
	e. [ ] FORMER HUSBAND/PARTNER/BOYFRIEND R HAD MORE THAN 12 MOS AGO	OFTEN. . . . . 1<br>SOMETIMES. . . . . . . 2<br>ONCE. 3	
	f. [ ] OWN FRIEND/ACQUAINTANCE	OFTEN. . . . . 1<br>SOMETIMES. . . . . . . 2<br>ONCE. 3	
	g. [ ] FRIEND OF FAMILY OR FAMILY MEMBER	OFTEN. . . . . 1<br>SOMETIMES. . . . . . . 2<br>ONCE. 3	
	h. [ ] TEACHER	OFTEN. . . . . 1<br>SOMETIMES. . . . . . . 2<br>ONCE. 3	
	i. [ ] EMPLOYER/SOMEONE AT WORK	OFTEN. . . . . 1<br>SOMETIMES. . . . . . . 2<br>ONCE. 3	
	j. [ ] POLICE/SOLDIER	OFTEN. . . . . 1<br>SOMETIMES. . . . . . . 2<br>ONCE. 3	
	k. [ ] PRIEST/PASTOR/RELIGIOUS LEADER	OFTEN. . . . . 1<br>SOMETIMES. . . . . . . 2<br>ONCE. 3	
	l. [ ] NEIGHBOR/SOMEONE IN COMMUNITY	OFTEN. . . . . 1<br>SOMETIMES. 2	

QUESTIONS and FILTERS		CODING CATEGORIES	SKIP TO
		ONCE. 3	
	m. [ ] STRANGER	OFTEN. . . . . 1<br>SOMETIMES. . . . . . . 2<br>ONCE. 3	
	n. [ ] OTHER- SPECIFY_____________	OFTEN. . . . . 1<br>SOMETIMES. . . . . . . 2<br>ONCE. 3	
505	As far as you know, did your father ever beat your mother?	YES . . . . .1<br>NO . . . . .2<br>DON'T KNOW. . .8<br>NO ANSWER . . . 9	

SUMMARY<br>Interviewer instructions: Review responses to Q411-417 and Q501 and code the following 2 variables.		
**506**	**Experienced at least one form of physical violence in the past 12 months?**	YES. . . . . 1<br>If ANY of the following conditions are met:<br>Q411=1, 2 or 3<br>Q412=1, 2 or 3<br>Q413=1, 2 or 3<br>Q414=1, 2 or 3<br>Q415=1, 2 or 3<br>Q416=1, 2 or 3<br>Q417=1, 2 or 3<br>Q501=1<br>NO/DON'T KNOW/NO ANSWER. . . . . 2<br>If NONE of the conditions above are met
**507**	**Experienced at least one form of sexual violence in the past 12 months?**	YES. . . . . 1<br>If ANY of the following conditions are met:<br>Q418 = 1, 2 or 3<br>Q419=1, 2 or 3<br>Q503=1<br>NO/DON'T KNOW/NO ANSWER . . . . 2<br>If NONE of the conditions above are met
**FILTER**		
**If Q506 =1 OR Q507=1 then CONTINUE WITH SECTION 6**<br>**If Q506=2 AND Q507=2 then GO TO SECTION 7**		

Section 6 Use of services for IPV or other GBV within past 12 months

INTERVIEWER: You have just shared with me that you recently experienced an act or situation that I'm going to refer to as "gender-based violence." I would now like to ask you about any actions you took as a result of this experience.

QUESTIONS and FILTERS		CODING CATEGORIES	SKIP TO
601	During the last 12 months, did you ever seek help to stop any type of this gender-based violence you experienced -- or seek care or treatment for the consequences of violence you experienced?	YES, SOUGHT HELP . . . . .1<br>NO, DID NOT SEEK HELP 2	 ➢ SECTION 7
602	Where and who did you go to for help in the past 12 months?<br><br>DO NOT READ; TICK ALL THAT R MENTIONS	a. [ ] OWN FAMILY MEMBER<br>b. [ ] IN-LAWS<br>c. [ ] HUSBAND/PARTNER<br>d. [ ] FORMER PARTNER<br>e. [ ] BEST MAN/BEST WOMEN<br>f. [ ] FRIEND<br>g. [ ] NEIGHBOUR<br>h. [ ] RELIGIOUS LEADER<br>i. [ ] LOCAL OFFICIAL<br>j. [ ] POLICE<br>k. [ ] LAWYER/LEGAL SERVICES<br>l. [ ] NGO/FBO<br>m. [ ] WELFARE OFFICE<br>n. [ ] SCHOOL OFFICIAL<br>o. [ ] SAFE HOUSE/SHELTER<br>p. [ ] HEALTH FACILITY<br>q. [ ] OTHER: SPECIFY<br>____________________	
603	Where/who did you go to first for help?<br><br>RECORD LETTER (a. – q.) OF RESPONSE FROM Q602	WENT TO FIRST [ ]	
604	How many times in the past 12 months did you visit a <u>health facility</u> to get care, treatment, or support for the gender-based violence you experienced?	NUMBER OF VISITS . . . [ ] [ ] [ ]<br><br>IF GREATER THAN 1, BEGIN BY ASKING Q605 ABOUT THE MOST RECENT VISIT AND CONTINUE ASKING ABOUT UP TO 3 VISITS	IF "0," SKIP TO SECTION 7

QUESTIONS and FILTERS		a.<br>MOST RECENT HEALTH FACILITY VISIT	b.<br>NEXT MOST RECENT HEALTH FACILITY VISIT	c.<br>NEXT MOST RECENT HEALTH FACILITY VISIT
**INTERVIEWER:** I am now going to ask you some questions about this visit/these visits, starting with your most recent visit.				
605	Was the health facility a dispensary, health centre, or hospital?	Dispensary. . . . . 1<br>Health centre . . . . . . 2<br>Hospital . . . . .3<br>Other. . . . . 4<br>Specify: ___________	Dispensary. . . . . 1<br>Health centre . . . . . . 2<br>Hospital . . . . .3<br>Other. . . . . .4<br>Specify: ___________	Dispensary. . . . . 1<br>Health centre . . . . . . 2<br>Hospital . . . . .3<br>Other. . . . . 4<br>Specify: ___________
606	What is the name of the health facility?	Facility name:<br>____________________	Facility name:<br>____________________	Facility name:<br>____________________
607	FILTER:<br>DO NOT ASK THIS QUESTION FOR COLUMN a. ASK ONLY FOR COLUMNS b. AND c., AS APPROPRIATE.<br><br>Was this visit for the same incident of violence as your previous visit to a health facility?	X	YES, SAME INCIDENT . . . . .1<br>NO, DIFFERENT INCIDENT. 2	YES, SAME INCIDENT . . . . .1<br>NO, DIFFERENT INCIDENT. 2
608	What types of services were you seeking or being referred to?<br><br>DO NOT READ; TICK ALL THAT R MENTIONS	a [ ] TREATMENT FOR PHYSICAL INJURIES<br>b [ ] TREATMENT FOR RAPE (OR SEXUAL VIOLENCE)<br>c [ ] PSYCHOSOCIAL COUNSELING/SUPPORT<br>d [ ] TEST FOR SEXUALLY TRANSMITTED INFECTION (STI)<br>e [ ] TREATMENT FOR STI<br>f [ ] PREGNANCY TEST<br>g [ ] FAMILY PLANNING<br>h [ ] EMERGENCY CONTRACEPTION<br>i [ ] HIV TEST<br>j [ ] HIV PEP<br>k [ ] PEP ADHERENCE<br>l [ ] FORENSIC EXAM<br>m [ ] REFERRAL TO ANOTHER SERVICE<br>n [ ] OTHER, Specify:<br>_______________	[ ] TREATMENT FOR PHYSICAL INJURIES<br>[ ] TREATMENT FOR RAPE (OR SEXUAL VIOLENCE)<br>[ ] PSYCHOSOCIAL COUNSELING/SUPPORT<br>[ ] TEST FOR SEXUALLY TRANSMITTED INFECTION (STI)<br>[ ] TREATMENT FOR STI<br>[ ] PREGNANCY TEST<br>[ ] FAMILY PLANNING<br>[ ] EMERGENCY CONTRACEPTION<br>[ ] HIV TEST<br>[ ] HIV PEP<br>[ ] PEP ADHERENCE<br>[ ] FORENSIC EXAM<br>[ ] REFERRAL TO ANOTHER SERVICE<br>[ ] OTHER, Specify:<br>_______________	[ ] TREATMENT FOR PHYSICAL INJURIES<br>[ ] TREATMENT FOR RAPE (OR SEXUAL VIOLENCE)<br>[ ] PSYCHOSOCIAL COUNSELING/SUPPORT<br>[ ] TEST FOR SEXUALLY TRANSMITTED INFECTION (STI)<br>[ ] TREATMENT FOR STI<br>[ ] PREGNANCY TEST<br>[ ] FAMILY PLANNING<br>[ ] EMERGENCY CONTRACEPTION<br>[ ] HIV TEST<br>[ ] HIV PEP<br>[ ] PEP ADHERENCE<br>[ ] FORENSIC EXAM<br>[ ] REFERRAL TO ANOTHER SERVICE<br>[ ] OTHER, Specify:<br>_______________

QUESTIONS and FILTERS		a.<br>MOST RECENT HEALTH FACILITY VISIT	b.<br>NEXT MOST RECENT HEALTH FACILITY VISIT	c.<br>NEXT MOST RECENT HEALTH FACILITY VISIT
609	FILTER: Is the category "Treatment for rape (Sexual Violence) ticked in Q608?	YES . . . . 1<br>NO . . . . 2<br>GO TO 611	YES . . . . 1<br>NO . . . . 2<br>GO TO 611	YES . . . . 1<br>NO . . . . 2<br>GO TO 611
610	Did you go to the health facility within 72 hours of the rape/sexual violence incident?	YES . . . . 1<br>NO . . . . 2<br>DON'T KNOW 8	YES . . . . 1<br>NO . . . . 2<br>DON'T KNOW 8	YES . . . . 1<br>NO . . . . 2<br>DON'T KNOW 8
611	How or why did you choose or go to this facility?<br><br>DO NOT READ; TICK ALL THAT R MENTIONS<br>PROBE: If you were referred, who referred you?	a [ ] AWARE THAT THIS FACILITY CAN TREAT THE PROBLEM<br>b [ ] FACILITY IS CLOSE/ NEARBY<br>c [ ] ALWAYS GO HERE FOR HEALTH SERVICES<br>d [ ] COULD GO HERE WITHOUT OTHERS KNOWING<br>e [ ] REFERRED HERE OR ACCOMPANIED BY POLICE<br>f [ ] REFERRED HERE BY LOCAL AUTHORITIES<br>g [ ] REFERRED HERE BY ANOTHER HEALTH FACILITY (SPECIFY_________)<br>h [ ] REFERRED HERE BY SUPPORT ORGANIZATION (NGO/FBO)<br>(SPECIFY_________)<br>i [ ] REFERRED HERE BY RELATIVE<br>j [ ] REFERRED HERE BY SCHOOL<br>k [ ] REFERRED HERE BY WELFARE OFFICER<br>l [ ] REFERRED HERE BY FRIEND/NEIGHBOR<br>m [ ] IT WAS THE ONLY OPTION AVAILABLE<br>n [ ] OTHER SPECIFY_____________	[ ] AWARE THAT THIS FACILITY CAN TREAT THE PROBLEM<br>[ ] FACILITY IS CLOSE/ NEARBY<br>[ ] ALWAYS GO HERE FOR HEALTH SERVICES<br>[ ] COULD GO HERE WITHOUT OTHERS KNOWING<br>[ ] REFERRED HERE OR ACCOMPANIED BY POLICE<br>[ ] REFERRED HERE BY LOCAL AUTHORITIES<br>[ ] REFERRED HERE BY ANOTHER HEALTH FACILITY (SPECIFY_________)<br>[ ] REFERRED HERE BY SUPPORT ORGANIZATION (NGO/FBO)<br>(SPECIFY_________)<br>[ ] REFERRED HERE BY RELATIVE<br>[ ] REFERRED HERE BY SCHOOL<br>[ ] REFERRED HERE BY WELFARE OFFICER<br>[ ] REFERRED HERE BY FRIEND/NEIGHBOR<br>[ ] IT WAS THE ONLY OPTION AVAILABLE<br>[ ] OTHER SPECIFY_____________	[ ] AWARE THAT THIS FACILITY CAN TREAT THE PROBLEM<br>[ ] FACILITY IS CLOSE/ NEARBY<br>[ ] ALWAYS GO HERE FOR HEALTH SERVICES<br>[ ] COULD GO HERE WITHOUT OTHERS KNOWING<br>[ ] REFERRED HERE OR ACCOMPANIED BY POLICE<br>[ ] REFERRED HERE BY LOCAL AUTHORITIES<br>[ ] REFERRED HERE BY ANOTHER HEALTH FACILITY (SPECIFY_________)<br>[ ] REFERRED HERE BY SUPPORT ORGANIZATION (NGO/FBO)<br>(SPECIFY_________)<br>[ ] REFERRED HERE BY RELATIVE<br>[ ] REFERRED HERE BY SCHOOL<br>[ ] REFERRED HERE BY WELFARE OFFICER<br>[ ] REFERRED HERE BY FRIEND/NEIGHBOR<br>[ ] IT WAS THE ONLY OPTION AVAILABLE<br>[ ] OTHER SPECIFY_____________

QUESTIONS and FILTERS		a. MOST RECENT HEALTH FACILITY VISIT			b. NEXT MOST RECENT HEALTH FACILITY VISIT			c. NEXT MOST RECENT HEALTH FACILITY VISIT		
612	What services did you receive at this health facility visit? READ EACH SERVICE AND RECORD ANSWER									
	**Service**	**YES**	**NO**	**DK**	**YES**	**NO**	**DK**	**YES**	**NO**	**DK**
	20. Screened and counseled for gender-based violence	1	2	8	1	2	8	1	2	8
	21. Physical exam	1	2	8	1	2	8	1	2	8
	22. Mental health exam	1	2	8	1	2	8	1	2	8
	23. Treated for physical injuries	1	2	8	1	2	8	1	2	8
	24. Forensic exam	1	2	8	1	2	8	1	2	8
	25. Forensic evidence collected	1	2	8	1	2	8	1	2	8
	26. PF3 form filled out	1	2	8	1	2	8	1	2	8
	27. Psychosocial counseling	1	2	8	1	2	8	1	2	8
	28. Family planning counseling	1	2	8	1	2	8	1	2	8
	29. HIV counseling	1	2	8	1	2	8	1	2	8
	30. PEP adherence counseling	1	2	8	1	2	8	1	2	8
	31. Pregnancy test	1	2	8	1	2	8	1	2	8
	32. HIV test	1	2	8	1	2	8	1	2	8
	33. STI test	1	2	8	1	2	8	1	2	8
	34. FP method	1	2	8	1	2	8	1	2	8
	35. Emergency contraception	1	2	8	1	2	8	1	2	8
	36. STI treatment	1	2	8	1	2	8	1	2	8
	37. PEP for HIV	1	2	8	1	2	8	1	2	8
	38. Tetanus toxoid vaccine	1	2	8	1	2	8	1	2	8
613	Overall, would you describe the services that you received as excellent, good, fair, poor, or very poor?	EXCELLENT. . . . . 1<br>GOOD. . . . . 2<br>FAIR. . . . . 3<br>POOR. . . . . 4<br>VERY POOR. 5			EXCELLENT. . . . . 1<br>GOOD. . . . . 2<br>FAIR. . . . . 3<br>POOR. . . . . 4<br>VERY POOR. 5			EXCELLENT. . . . . 1<br>GOOD. . . . . 2<br>FAIR. . . . . 3<br>POOR. . . . . 4<br>VERY POOR. 5		

QUESTIONS and FILTERS		a.<br>MOST RECENT HEALTH FACILITY VISIT	b.<br>NEXT MOST RECENT HEALTH FACILITY VISIT	c.<br>NEXT MOST RECENT HEALTH FACILITY VISIT
614	At this visit to the health facility, were you referred to services that are provided <u>outside this facility</u>?	YES . . . . 1<br>NO . . 2 GO TO **FILTER 2**	YES . . . . 1<br>NO . . 2 GO TO **FILTER 2**	YES . . . . 1<br>NO . . 2 GO TO **FILTER 2**
615	What services were you referred to? And did you follow through on this/these referrals and go to these services to which you were referred? READ EACH SERVICE AND RECORD ANSWER			
	h. Psychosocial care	YES, REFERRED AND WENT . . . . 1<br>YES, REFERRED BUT DID NOT GO . . . . 2<br>NOT REFERRED . . . 3	YES, REFERRED AND WENT . . . . 1<br>YES, REFERRED BUT DID NOT GO . . . . 2<br>NOT REFERRED . . . 3	YES, REFERRED AND WENT . . . . 1<br>YES, REFERRED BUT DID NOT GO . . . . 2<br>NOT REFERRED . . . 3
	i. Police	YES, REFERRED AND WENT . . . . 1<br>YES, REFERRED BUT DID NOT GO . . . . 2<br>NOT REFERRED . . . 3	YES, REFERRED AND WENT . . . . 1<br>YES, REFERRED BUT DID NOT GO . . . . 2<br>NOT REFERRED . . . 3	YES, REFERRED AND WENT . . . . 1<br>YES, REFERRED BUT DID NOT GO . . . . 2<br>NOT REFERRED . . . 3
	j. Safe house or shelter	YES, REFERRED AND WENT . . . . 1<br>YES, REFERRED BUT DID NOT GO . . . . 2<br>NOT REFERRED . . . 3	YES, REFERRED AND WENT . . . . 1<br>YES, REFERRED BUT DID NOT GO . . . . 2<br>NOT REFERRED . . . 3	YES, REFERRED AND WENT . . . . 1<br>YES, REFERRED BUT DID NOT GO . . . . 2<br>NOT REFERRED . . . 3
	k. Clinical care at higher level facility	YES, REFERRED AND WENT . . . . 1<br>YES, REFERRED BUT DID NOT GO . . . . 2<br>NOT REFERRED . . . 3	YES, REFERRED AND WENT . . . . 1<br>YES, REFERRED BUT DID NOT GO . . . . 2<br>NOT REFERRED . . . 3	YES, REFERRED AND WENT . . . . 1<br>YES, REFERRED BUT DID NOT GO . . . . 2<br>NOT REFERRED . . . 3
	l. Legal services	YES, REFERRED AND WENT . . . . 1<br>YES, REFERRED BUT DID NOT GO . . . . 2<br>NOT REFERRED . . . 3	YES, REFERRED AND WENT . . . . 1<br>YES, REFERRED BUT DID NOT GO . . . . 2<br>NOT REFERRED . . . 3	YES, REFERRED AND WENT . . . . 1<br>YES, REFERRED BUT DID NOT GO . . . . 2<br>NOT REFERRED . . . 3
	m. Other1: SPECIFY<br>____________________	YES, REFERRED AND WENT . . . . 1<br>YES, REFERRED BUT DID NOT GO . . . . 2<br>NOT REFERRED . . . 3	YES, REFERRED AND WENT . . . . 1<br>YES, REFERRED BUT DID NOT GO . . . . 2<br>NOT REFERRED . . . 3	YES, REFERRED AND WENT . . . . 1<br>YES, REFERRED BUT DID NOT GO . . . . 2<br>NOT REFERRED . . . 3
	n. Other2: SPECIFY<br>____________________	YES, REFERRED AND WENT 1	YES, REFERRED AND WENT 1	YES, REFERRED AND WENT 1

QUESTIONS and FILTERS		a.<br>MOST RECENT HEALTH FACILITY VISIT	b.<br>NEXT MOST RECENT HEALTH FACILITY VISIT	c.<br>NEXT MOST RECENT HEALTH FACILITY VISIT
		YES, REFERRED BUT DID NOT GO . . . . 2<br>NOT REFERRED . . . 3	YES, REFERRED BUT DID NOT GO . . . . 2<br>NOT REFERRED . . . 3	YES, REFERRED BUT DID NOT GO . . . . 2<br>NOT REFERRED . . . 3
**FILTER 1**: If any response to Q615 is 2 (REFERRED BUT DIDN'T GO), then ask Q616. Otherwise GO TO **FILTER 2**.				
616	What were the reasons that you didn't follow through on this referral/these referrals?<br><br>TICK ALL THAT R MENTIONS; DO NOT READ	a [ ] DIDN'T WANT THE SERVICE<br>b [ ] COULD'T AFFORD THE SERVICE<br>c [ ] DIDN'T HAVE/ COULDN'T AFFORD TRANSPORTATION<br>d [ ] DIDN'T HAVE TIME<br>e [ ] HAD TO WORK<br>f [ ] DIDN'T HAVE CHILDCARE<br>g [ ] HAD FAMILY RESPONSIBILITIES/ HOUSEHOLD OBLIGATIONS<br>h [ ] HUSBAND/ PARTNER WOUDN'T ALLOW IT<br>i [ ] DIDN'T KNOW WHERE TO GO<br>j [ ] AFRAID TO BE SEEN/THAT OTHERS WOULD FIND OUT<br>k [ ] OTHER<br>SPECIFY:____________	[ ] DIDN'T WANT THE SERVICE<br>[ ] COULD'T AFFORD THE SERVICE<br>[ ] DIDN'T HAVE/ COULDN'T AFFORD TRANSPORTATION<br>[ ] DIDN'T HAVE TIME<br>[ ] HAD TO WORK<br>[ ] DIDN'T HAVE CHILDCARE<br>[ ] HAD FAMILY RESPONSIBILITIES/ HOUSEHOLD OBLIGATIONS<br>[ ] HUSBAND/ PARTNER WOUDN'T ALLOW IT<br>[ ] DIDN'T KNOW WHERE TO GO<br>[ ] AFRAID TO BE SEEN/THAT OTHERS WOULD FIND OUT<br>[ ] OTHER<br>SPECIFY:____________	[ ] DIDN'T WANT THE SERVICE<br>[ ] COULD'T AFFORD THE SERVICE<br>[ ] DIDN'T HAVE/ COULDN'T AFFORD TRANSPORTATION<br>[ ] DIDN'T HAVE TIME<br>[ ] HAD TO WORK<br>[ ] DIDN'T HAVE CHILDCARE<br>[ ] HAD FAMILY RESPONSIBILITIES/ HOUSEHOLD OBLIGATIONS<br>[ ] HUSBAND/ PARTNER WOUDN'T ALLOW IT<br>[ ] DIDN'T KNOW WHERE TO GO<br>[ ] AFRAID TO BE SEEN/THAT OTHERS WOULD FIND OUT<br>[ ] OTHER<br>SPECIFY:____________
**FILTER 2:**		If Q604 >1, then continue with column b; else GO TO SECTION 7	If Q604 >2, then continue with column c; else GO TO SECTION 7	GO TO SECTION 7

Section 7 Participation in GBV community programs

INTERVIEWER: I would now like to ask you about your participation in community activities.

QUESTIONS and FILTERS		CODING CATEGORIES	SKIP TO
➢ Participation in community activities of any kind			
701a	In the past two years, did you attend or participate in any kind of group activity, association or organisation?<br><br>IF RESPONSE IS NO OR DON'T KNOW PROBE: This includes organisations like women's or community groups, and religious and political associations.	YES . . . . 1<br>NO . . . . 2<br>DON'T KNOW 8	 ➢ 702a<br>➢ 702a
701b	What kind(s) of group activity, association or organisation did you attend or participate in?<br><br>DO NOT READ; TICK ALL THAT R MENTIONS	[ ] Sports group<br>[ ] Music and dance group<br>[ ] Women's group/Co-Op<br>[ ] Burial society<br>[ ] Religious group<br>[ ] Political group<br>[ ] HIV Care and support group<br>[ ] Peer education group<br>[ ] Youth group<br>[ ] Community Health group<br>[ ] Parent-teachers association<br>[ ] Sewing group<br>[ ] Development committee (e.g. land trusts, water & sanitation committee)<br>[ ] Other, specify<br>______________________	
➢ Awareness of and participation in HJFMRI GBV Community Programs			
702a	Have you heard about a programme called the WRP/HJFMRI Gender-Based Violence Programme – also known as the HJFMRI GBV Programme or AMKA SASA?	YES . . . . .1<br>NO 2	 ➢ 703a
702b	Did you participate in a launch of this programme?	YES . . . . .1<br>NO 2	

QUESTIONS and FILTERS		CODING CATEGORIES	SKIP TO
703a	Have you heard about a group education program that works with women and men to raise awareness about gender, gender-based violence, to reduce acceptability of gender-based violence, and to engage men in stopping violence against women and children?	YES . . . . 1<br>NO . . . . .2<br>DON'T KNOW. . . 8	 ➢ 704a<br>➢ 704a
703b	Have you participated in one or more of these group education sessions on gender and GBV?	YES, PARTICIPATED . . . . 1<br>HAVE NEVER PARTICIPATED. . . . . .2<br>Don't know. 8	➢ 704a<br>➢ 704a
703c	How many group education sessions on gender and GBV did you participate in?	[ ] [ ]	
704a	Have you heard about "Couples Connect," which is a program that works with couples to improve the way women and men in partnerships (e.g. husbands and wives) talk to each other?	YES . . . . .1<br>NO . . . . .2<br>DON'T KNOW. 8	 ➢ 705a<br>➢ 705a
704b	How often have you participated in "Couples Connect"?	OFTEN. . . . . 1<br>SOMETIMES. . . . . .2<br>ONCE. . . . . 3<br>NEVER. . . . . 4<br>DON'T KNOW/<br>DON'T REMEMBER 8	
705a	Have you heard about community dialogues--that is, gatherings organized for community members to come together to discuss issues of gender-based violence/GBV or violence against women and children?	YES . . . . 1<br>NO . . . . 2<br>DON'T KNOW. . .8	 ➢ 706a<br>➢ 706a

QUESTIONS and FILTERS		CODING CATEGORIES	SKIP TO
705b	How often have you participated in such dialogues?	OFTEN. . . . . 1<br>SOMETIMES. . . . . 2<br>ONCE. . . . . .3<br>NEVER. . . . . 4<br>DON'T KNOW/<br>DON'T REMEMBER 8	
706a	Is there a GBV Coordination Committee in your community that focuses on gender-based violence/GBV or violence against women and children?	YES . . . . .1<br>NO . . . . 2<br>DON'T KNOW. . . 8	➢ 707a<br>➢ 707a
706b	What do members of the GBV Coordination Committee do?<br>DO NOT READ THE LIST. CHECK ALL RESPONSES THAT R MENTIONS.	a. [ ] Help GBV survivors get to the health facility<br>b. [ ] Take GBV cases to the police<br>c. [ ] Settle GBV cases<br>d. [ ] Educate the community about GBV<br>e. [ ] OTHER<br>specify ______________________	
707a	Are you aware of any current or recent media campaigns (for example, posters, billboards, radio, or TV messages) that address gender-based violence/GBV or violence against women and children?	YES . . . . .1<br>NO 2	➢ 708
707b	Does/did the campaign or campaigns have a name or slogan? Can you tell me the name(s)?<br><br>DO NOT READ THE RESOPNSES; TICK THE APPROPRIATE BOXES.	a. [ ] R MENTIONED "AMKA SASA"<br><br>b. [ ] R MENTIONED "BE A ROLE MODEL"<br><br>c. [ ] R MENTIONED NAME(S) OTHER THAN "AMKA SASA" OR "BE A ROLE MODEL"<br><br>d. [ ] R INDICATED THE CAMPAIGN HAS NO NAME e. [ ] R INDICATED IT HAS A NAME,<br>BUT SHE DOESN'T<br>REMEMBER IT	➢
707c			

QUESTIONS and FILTERS		CODING CATEGORIES	SKIP TO
708	I'd now like to ask you about some other community activities that may have **included information about gender-based violence/GBV or violence against women and children**. I'm going to read to you the events and I'd like you to tell me if you participated in them in the past two years, and if so, how often.<br><br>READ ALL CATEGORIES AND ASK ABOUT FREQUENCY.		
	g. Events held during special, national and international days (e.g. Nane Nane, Saba Saba, World AIDS Day, International Women's Day, etc)	Participated:<br>OFTEN. . . . . 1<br>SOMETIMES. . . . . .2<br>ONCE (in past 2 yrs) . . . . 3<br>NEVER. . . . . 4<br>DON'T KNOW/<br>DON'T REMEMBER 8	 <br><br><br>➢ 708c<br><br>➢ 708c
	h. Was any information on GBV or violence against women and children given out or discussed?	YES . . . . 1<br>NO . . . . 2<br>DON'T KNOW/<br>DON'T REMEMBER 8	
	i. Community sporting events?	Participated:<br>OFTEN. . . . . 1<br>SOMETIMES. . . . . .2<br>ONCE (in past 2 yrs) . . . . 3<br>NEVER. . . . . 4<br>DON'T KNOW/<br>DON'T REMEMBER 8	 <br><br><br>➢ 708e<br><br>➢ 708e
	j. Was any information on GBV or violence against women and children given out or discussed?	YES . . . . 1<br>NO . . . . 2<br>DON'T KNOW/<br>DON'T REMEMBER 8	
	k. Street outreach, or outreach activities using boda boda?	Participated:<br>OFTEN. . . . . 1<br>SOMETIMES. . . . . 2<br>ONCE (in past 2 yrs) . . . . . . .3<br>NEVER. . . . . 4<br>DON'T KNOW/<br>DON'T REMEMBER 8	 <br><br><br>➢ 709<br><br>➢ 709
	l. Was any information on GBV or violence against women and children given out or discussed?	YES . . . . 1<br>NO . . . . 2<br>DON'T KNOW/<br>DON'T REMEMBER 8	

QUESTIONS and FILTERS		CODING CATEGORIES	SKIP TO
709	Have you heard or seen any public messages that target men in bars about stopping violence against women and children? If so, how often?	OFTEN. . . . . 1<br>SOMETIMES. . . . . 2<br>ONCE. . . . . 3<br>NEVER. . . . . 4<br>DON'T KNOW/<br>DON'T REMEMBER 8	
709a	In your community, are there community educators or community volunteers who help survivors of GBV get to services they may need?	YES . . . . 1<br>NO . . . . 2<br>DON'T KNOW 8	➢710<br>➢ 710
709c	What services do they refer or take them to?<br><br>DO NOT READ THE LIST. CHECK ALL RESPONSES THAT R MENTIONS.	a. [ ] Health facility<br>b. [ ] Police<br>c. [ ] Social welfare<br>d. [ ] Ward leader or other local official<br>e. [ ] Religious leader<br>f. [ ] Other<br>Specify: ______________________	
710	Apart from what we have already discussed, in the last two years have you heard about any other activities in your community aimed to stop GBV or violence against women and children?	YES . . . . 1<br><br>a.[ ]SPECIFY ACTIVITY:<br>______________________________<br><br>b.[ ] SPECIFY ACTIVITY:<br>______________________________<br><br>c.[ ] SPECIFY ACTIVITY:<br>______________________________<br><br>d.[ ] SPECIFY ACTIVITY:<br>______________________________<br><br>NO 2	➢ Section 8

QUESTIONS and FILTERS		CODING CATEGORIES	SKIP TO
711	How often have you participated in this/these activities?<br><br>FOR EACH OF THE SPECIFIC ACTIVITIES MENTIONED IN 710 (a-d), MARK THE FREQUENCY OF PARTICIPATION.	a.<br>OFTEN. . . . . 1<br>SOMETIMES. . . . . 2<br>ONCE. . . . . 3<br>NEVER. . . . . 4<br>DON'T KNOW/<br>DON'T REMEMBER. . . 8<br><br>b.<br>OFTEN. . . . . 1<br>SOMETIMES. . . . . 2<br>ONCE. . . . . 3<br>NEVER. . . . . 4<br>DON'T KNOW/<br>DON'T REMEMBER. . . 8<br><br>c.<br>OFTEN. . . . . 1<br>SOMETIMES. . . . . 2<br>ONCE. . . . . 3<br>NEVER. . . . . 4<br>DON'T KNOW/<br>DON'T REMEMBER. . . 8<br><br>d.<br>OFTEN. . . . . 1<br>SOMETIMES. . . . . 2<br>ONCE. . . . . 3<br>NEVER. . . . . 4<br>DON'T KNOW/<br>DON'T REMEMBER. . . 8	

Section 8 GBV knowledge and attitudes, gender norms, and behavior change

INTERVIEWER: Finally, I'd like to ask you about some types of violence in your community and your thoughts about that.

QUESTIONS and FILTERS		CODING CATEGORIES	SKIP TO
➢ Awareness of recent cases in the community and community response			
801a	Have there been any cases in your community within the past 12 months where a woman has been physically or sexually abused by her husband or partner?	YES . . . . .1<br>NO . . . . 2<br>DON'T KNOW 8	 ➢ 802a<br>➢ 802a
801b	How many cases are you aware of?<br><br>RECORD THE NUMBER OF CASES STATED BY THE RESPONDENT.	NUMBER OF CASES<br>[ ] [ ] [ ] [ ]<br><br>Don't Know = 9998	
802a	Have there been any cases in your community within the past 12 months where a child has been sexually abused?	YES . . . . .1<br>NO . . . . 2<br>DON'T KNOW 8	 ➢ 803<br>➢ 803
802b	How many cases are you aware of?<br><br>RECORD THE NUMBER OF CASES STATED BY THE RESPONDENT.	NUMBER OF CASES<br>[ ] [ ] [ ] [ ]<br><br>Don't Know = 9998	
803	In your opinion, in the past 12 months has your community done a good job of responding to cases of husband/partner violence and sexual abuse toward children?	Very good job . . . . .1<br>Good job . . . . 2<br>Average job. . . . . .3<br>Poor job . . . . 4<br>Very poor job . . . . 5<br>Don't know 8	
➢ Awareness of laws/policies and recent actions of local leaders on violence			
804	Are you familiar with (do you know) the Tanzanian laws and policies regarding violence against women and children?	VERY AWARE/ KNOWLEDGEABLE . . . 1<br>SOMEWHAT AWARE. . . . . .2<br>NOT AWARE . . . . 3<br>DON'T KNOW. 8	
805	In the past 12 months, how often have local leaders or officials in your community publically spoken out or taken action to address gender-based violence or violence against women and children?	OFTEN. . . . . 1<br>SOMETIMES. . . . . . 2<br>ONCE. . . . . 3<br>NEVER. . . . . .4<br>DON'T KNOW. 8	

➢ Definition or recognition of VAW (adapted from DHS and CHAMPION study)				
806	In your opinion, do you consider it an act of violence against a woman when a woman's husband…	**YES**	**NO**	**DON'T KNOW**
	a. Does something to humiliate her in front of others	1	2	8
	b. Threatens to harm her or someone she is close to	1	2	8
	c. Insults her or makes her feel bad about herself	1	2	8
	d. Pushes, slaps, hits, or kicks her	1	2	8
	e. Threatens to attack her with a knife, gun or other weapon	1	2	8
	f. Physically forces her to have sexual intercourse even when she does not want to	1	2	8
	g. Forces her to do something sexual that she doesn't want to do	1	2	8
	h. Restricts her movement	1	2	8
	i. Kicks her out of the house	1	2	8
	j. Has an extramarital affair	1	2	8
	k. Makes all decisions about how family earnings are spent including the wife's earnings	1	2	8
807	In your opinion, do you consider it an act of violence against a woman when someone other than her husband …	**YES**	**NO**	**DON'T KNOW**
	a. Does something to humiliate her in front of others	1	2	8
	b. Threatens to harm her or someone she is close to	1	2	8
	c. Insults her or makes her feel bad about herself	1	2	8
	d. Pushes, slaps, hits, or kicks her	1	2	8
	e. Threatens to attack her with a knife, gun or other weapon	1	2	8
	f. Physically forces her to have sexual intercourse even when she does not want to	1	2	8
	g. Forces her to do something sexual that she doesn't want to do	1	2	8

➢ Recognition or attitudes of sexual violence against children				
808	I'm going to read some statements to you and I'd like you to tell me if you agree, partially agree, or do not agree.	**AGREE**	**PARTIALLY AGREE**	**DO NOT AGREE**
	a. A child is sexually abused only when sexual intercourse has taken place	1	2	3
	b. When a child is sexually abused, the abuser is rarely a family member	1	2	3
	c. Only girls are sexually abused	1	2	3
	d. It is not possible for children under 10 years of age to experience sexual abuse	1	2	3
	e. Children from reputable families do not experience sexual abuse	1	2	3

➢ Attitudes about domestic/marital violence (from DHS)				
809	In your opinion, is a husband justified in hitting or beating his wife in the following situations:	**YES**	**NO**	**DON'T KNOW**
	a. If she goes out without telling her husband/partner?	1	2	8
	b. If she neglects the children?	1	2	8
	c. If she argues with her husband/partner?	1	2	8
	d. If she refuses to have sex with her husband/partner?	1	2	8
	e. If she burns the food?	1	2	8

➢ Gender norms (GEM scale: Violence domain items)				
810	I'm now going to read some statements and I'd like you to tell me if you agree, partially agree, or do not agree.	**AGREE**	**PARTIALLY AGREE**	**DO NOT AGREE**
	a. There are times when a woman deserves to be beaten.	1	2	3
	b. A woman should tolerate violence to keep her family together.	1	2	3
	c. It is alright for a man to beat his wife if she is unfaithful.	1	2	3
	d. A man can hit his wife if she won't have sex with him.	1	2	3
	e. If someone insults a man, he should defend his reputation with force if he has to.	1	2	3
	f. A man using violence against his wife is a private matter that shouldn't be discussed outside the couple	1	2	3

➢ Gender norms (GEM scale: Domestic chores and daily life domain Items)				
811	*Continue reading statements…*	**AGREE**	**PARTIALLY AGREE**	**DO NOT AGREE**
	a. Changing diapers, giving a bath, and feeding kids is the mother's responsibility.	1	2	3
	b. A woman's role is taking care of her home and family.	1	2	3
	c. The husband should decide to buy the major household items.	1	2	3
	d. A man should have the final word about decisions in his home.	1	2	3
	e. A woman should obey her husband in all things.	1	2	3

➢ R's behavior change: Taking action		
812a	In the past 12 months, have you started a conversation about gender-based violence/GBV or violence against women or children with anyone?	Yes . . . . 1<br>No . . . . 2 GO TO 813a
812b	With whom did you have this conversation?<br><br>CHECK ALL THAT R MENTIONS.	[ ] Your partner(s)<br>[ ] Friend<br>[ ] Family Member<br>[ ] Neighbour or other community member<br>[ ] Other, specify ______________________<br>[ ] Other, specify ______________________
813a	In the past 12 months, did you personally witness any acts of GBV or violence against a woman or child-- or encounter someone who experienced this violence?	Yes . . . . 1<br>No . . . . 2 GO TO 814
813b	Did you take any action to try to stop it or help the survivor (for any of the times you witnessed it)?	Yes . . . . 1<br>No . . . . 2 GO TO 814
813c	What action(s) did you take?<br><br>DO NOT REDA.CHECK ALL THAT R MENTIONS.	[ ] Listened to the survivor and offered emotional support<br>[ ] Provided information about services and programs designed to help survivors<br>[ ] Accompanied the survivor to a clinic or support agency<br>[ ] Talked with family members of the survivor about the violence<br>[ ] Tried to stop or stopped the violence<br>[ ] Reported the incident to authorities<br>[ ] Other, specify<br>____________________________________
814	Do you think differently about gender-based violence/ GBV or violence against women and children today than you did 12 months ago?	Yes . . . . .1<br>No 2
815	If you were to witness such violence today would you be more or less likely to take action than you would have 3 months ago?	MORE LIKELY . . . . .1<br>ABOUT AS LIKELY . . . . . 2<br>LESS LIKELY 3

Section 9 Completion of Interview

901	**INTERVIEWER:**<br>We are coming to the end of the interview. There is just one last step.<br>I'm going to give you a card. On this card are two pictures. No other information is written on the card. One picture is of a sad face, the other is of a happy face.<br>GIVE RESPONDENT THE CARD AND PEN.<br>No matter what you have already told me, I would like you to put a mark next to the sad picture if someone hurt **you** physically, touched you sexually, or made you do something sexual that you didn't want to in the last 12 months.<br>Please put a mark next to the happy face if this did not happen to you in the last 12 months. Once you have marked the card, please fold it over and put it in this bag. The bag contains similar cards from many other women. This will ensure that I do not know your answer.<br><br>DO NOT LOOK AT THE RESPONSE. ASK RESPONDENT TO PUT The MARKED AND FOLDED CARD INTO A BAG THAT ALSO CONTAINS OTHER COMPLETED CARDS. DO NOT RECORD ANYTHING ON THE CARD.	CARD COMPLETED....1<br><br>CARD NOT COMPLETED....2
902	I have asked you about many difficult things. How has talking about these things made you feel?<br><br>READ OPTIONS	GOOD/BETTER. . . . . 1<br>BAD/WORSE. . . . . .2<br>SAME/ NO DIFFERENCE. . . .3
903	We have now finished the interview. Do you have any comments, or is there anything else you would like to share?<br>RECORD COMMENTS.	
904	FINISH<br>I would like to thank you very much for helping us. I appreciate the time you have taken. I realize that these questions may have been difficult for you to answer, but it is only by hearing from women themselves that we can really understand about their health and experiences in life.<br><br>In case you are interested or if you ever hear of another person who needs help related to violence and the things we have been talking about, I'm going to leave you with the name and contact information of a service provider at _________________ Health Center/_________Hospital, who can offer assistance. This person is service provider at this health facility who also is working with us on this study. S/he is aware that we are giving out this information as part of the interview. If you go, you can be assured that s/he will keep anything that you say private. You can go whenever you feel ready to, either soon or later on.<br><br>LEAVE PAPER SLIP WITH NAME OF HEALTH FACILITY IN THE STUDY CLUSTER, NAME OF TATHMINI GBV DATA FOCAL PERSON, AND CONTACT INFORMATION.	

	If you are interested, I can also ask this service provider to contact you directly. If you would like this referral, I will take down your contact information, including your name and a safe place where or private way that s/he or another trained colleague can find you. I will record this information on a separate form, which will not be linked in any way with the interview we have just completed. I will then give this form to my team leader, who will provide the form directly to the service provider. Information you have shared during this interview will not be shared with the service provider.<br><br>Would you like me to put you in direct contact with a service provider?<br>YES…………………….1<br>NO…………………….2<br><br>IF THE RESPONDENT INDICATES 'YES,' FILL IN THE REFERRAL FORM, PLACE IT IN THE DESIGNATED/UNMARKED ENVELOPE AND GIVE IT TO THE TEAM LEADER.
**INTERVIEWER:**	Record time of end of interview: [ ][ ]:[ ] [ ] (Use 24 hr clock)
	Interview completed: _______Yes, complete ______No, incomplete<br><br>If interview is incomplete, please give reason (s):<br><br><br><br>Signature of Interviewer: ____

Annex B-2.

Key informant interview guide: Community leaders/gate-keepers

Key Informant Interview Guide: Community Leaders/Gatekeepers

Background

Key informant interviews will be conducted with community leaders and/or gatekeepers in the six intervention clusters in order to understand their own knowledge and attitude regarding GBV, gather opinions about their experiences in handling GBV cases. Exploring whether these leaders also believe attitudes have changed in the community during the study period will be another focus of the interviews. These leaders and gatekeepers also will be asked for their opinions about the strengths and weaknesses of the comprehensive GBV program, and for their suggestions for ongoing improvements to the program, as well as about any community-led actions to address GBV that they have witnessed and/or participated in due to the implementation of the program.

Knowledge and experience of GBV

- When you hear the words "gender-based violence" or GBV what does it mean to you?
    - Probe for them to describe/define GBV

- One definition of GBV is "violence involving men and women, in which the female is usually the victim; and which is derived from unequal power relationships between men and women. GBV occurs in many forms, including but not limited to intimate partner violence (IPV), domestic violence, sexual violence, and femicide or the killing of women because of their gender by males.'
    - Based on this definition, please describe any instances/forms of GBV that you have witnessed in the community.
    - Please share with me an incidence of GBV that you experienced

Knowledge of GBV services

- If a woman (or man, or child) experiences GBV, what services/organizations do you know of in your community that they may go to? What services/help do these organizations provide? Where are these services/organizations located in your community?

- What do people accessing these services say about them? What makes it easy for people to take advantage of these services? What makes it difficult for community members to take advantage of these services? What are some of the gaps, e.g. areas where services aren't available in your community?

- Please tell me about someone you know who who utilized these services. What can you tell us about their experiences with these services?

Norms and Attitudes about gender and GBV

- In your opinion, what are some circumstances in which a husband is justified in hitting or beating his wife/partner?
    - Probe on:

- If she goes out without telling him?
    - If she neglects the children?
    - If she argues with him?
    - If she refuses to have sex with him?
    - If she burns the food?

- In general, for what reasons/under what circumstances are men most likely to hit or beat their wives/partners?
    - Are there times of the year this is more likely to happen? E.g. harvesting time?
    - What is the role of alcohol/marijuana use in GBV?
    - What do women do to "provoke" men?

- In general, is it ok for women to refuse/resist sex with their husbands/partners? If not, is it ok for men to force/demand their wives to have sex, even if she has said no?

- If a woman does experience violence, what should she do?
    - Probe:
    - Tolerate it to keep the family together?
    - Discuss it with family members to garner support to stop the violence
    - Report it to police/local authorities?
    - Seek medical care/social support?
    - Leave her husband?

- What can you tell us about sexual violence against children in your community?
    - Does it happen? Where/why?
    - Who is perpetrating it/who are the victims?
    - What can be done to protect children from this/are there services to help survivors of this?

Knowledge and effects of Comprehensive GBV Program
Since [January 2013], there has been a program in your community designed to address issues of GBV. Through this program, services have been rolled out at hospitals and clinics to support survivors of GBV and there have also been activities rolled out at the community-level designed to prevent the perpetration of GBV.

- Are you aware of this/these program/s? If yes, what can you tell me about them?
    - Probe on: what services, where? Has the respondent had any interaction with the program? Does he or she know anyone who participated in the program?

- Have you witnessed or participated in any changes in the community, or community-led actions (e.g. neighbors stopping other neighbors engaging in GBV) against GBV, based on this program?

- What effects if any has this program had on you personally, either in your own thinking or behavior related to GBV, and/or more generally in your ideas about women or how you interact with your spouse/partner?

Respondent's Role in the Community as it Relates to GBV

- How would you describe your job/role in the community?
- In terms of your job/role in the community, what support to survivors of GBV that you have provided?
    - what kind of support did you provide?
    - How did you handle GBV cases?
    - What are some examples of things that have changed since the comprehensive program has been put in place?
    - Also in terms of your job/role in the community, how have you used this role to influence others in your community on issues related to GBV? How have you used this role to influence policies in your community on issues related to GBV?
    - If so, in what ways?
    - What, have been some outcomes from your influence?

Is there anything else regarding GBV in your community that you would like to share with me?

Annex B-3

Key Informant Interviews with Participants of the Prevention and Community Programs

Key Informant Interview Guide: Participants of the Prevention and Community Programs

1. Introduction

Thank you for taking time to speak with me today. My name is ____________, and I look forward to our discussion. You have been invited to participate in this discussion because of your engagement in a program (or programs) within your community designed to address gender-based violence (GBV). The overall purpose of our discussion today is to help the study team learn about your experience with this program, and if and how this experience has influenced any of your beliefs and behaviors about GBV. We hope this information will help improve programs and services in the future.

Please remember that anything you share with me will be kept confidential. We will tape record the conversation, but we will not use your name on the tape, nor will we connect your name to any of the information collected. At any time, for any reason, you can stop the interview or decline to answer a question. The discussion will take about one hour.

Do you have any questions for me before we begin (pause for questions)?

2. **Knowledge of, and experience with the GBV program (of the study)**

Since [January 2014], there has been a program in your community designed to address issues of gender-based violence (GBV). Through this program, services have been rolled out in health facilities in your area to support survivors of GBV. There have also been activities started at the community-level to raise awareness about the situation of GBV and to stop perpetration of GBV. You have been selected to be interviewed because you have participated in at least one of these programs. I am going to start the discussion by asking you about your experiences with the program, and then move on to discuss your own feelings about and experiences with GBV.

Please tell me about your experience with this/these program/s:

<u>Note to interviewer</u>: Please probe on the following items related to the participants experience with the program:

- What do you know about the program?
- What interaction have you had with the program?
- What parts of the program have you participated in?
  How frequently have you participated in the program over the last 12 months?
- Who else close to you (e.g., family member or friend) has participated in the program? What was their role in the program?

I'd like to know about the effects/impact that this program has had on you personally:

- Please describe if and how these programs have changed your own thinking or attitudes about thegender roles of women and men in your household and your community.
- Please describe if and how these changes have effected how you interact with your partner or others in your life?

I would also like to know about the effects/impact this program may have had on others participating in the program, or the community overall.

- Please tell me about any changes or actions related to GBV that you've witnessed among others who have participated in the program or in the community overall.
  - How do you think the program has influenced these changes or actions?
  - What do you think about these changes?
- Please tell me about incidents of GBV in your community
  - Have you witnessed any incident of violence against a woman or child in your area?
  - If yes, can you describe what happened in this (or the most recent) incident recent you witnessed?
  - Are such incidents common in your area? Have you noticed that there's been an increase or decrease in the numbers over the past 12 months?
- [Note to interviewer: Probe to find out if violence is associated with other human behavior such as drug use, insubordination of women, children maltreatment, etc.]
- Have any local community measures been taken in addressing such incidents?
  - [Note to the interviewer: If, yes, please probe on the details of the actions and when this took place.]

2. Norms and attitudes about gender and GBV

Now I would like to ask some more specific questions about your feelings and ideas about gender and GBV.

In your opinion, what are some circumstances, if any, under which it is ok for a man/husband to hit or beat his partner/wife?

Note to interviewer: Please probe on the following types of circumstances that might be considered acceptable reasons for men/husbands to hit or beat or otherwise abuse their partners:

- if she goes out without telling him?
- if she neglects the children?
- if she argues with him?
- if she refuses to have sex with him?
- if she burns the food?

From your experience, for what reasons/under what circumstances are men most likely to hit or beat their wives/partners?

Note to interviewer: Please probe on the following types of reasons/circumstances

- What role does alcohol or marijuana use play in these episodes?
- Do women "provoke" men? If yes, what is it that they do?
- Are there times of the year when this is more likely to happen? Probe on: harvesting time, holidays, when men return from working away from home, etc.

In general, how do you feel about wives/women refusing or resisting sex with their husbands/partners?

What do you think about men forcing/demanding their wives/partners to have sex, if she has said no? What about couples who are not married, but are in a steady relationship?

If a woman does experience violence, what do you think she should she do?

Note to interviewer: Please probe on the following things the respondent might highlight:

- tolerate the violence/keep silent
- discuss it with family members
- report it to police/local authorities
- seek medical care/social support

For all possibilities, ask why a woman should do these things. What might be some of the challenges?

Is leaving her husband an option? Please tell me why or why not.

3. Response to GBV against women/adults

What do families do in case of violence between a man/husband and woman/wife (intimate partner violence)?

Note to interviewer: Please probe on details of measures taken including:

- whether reporting to police is among the options for addressing IPV
- how follow-up takes place for any of the situations where a case is reported
- awareness of the GBV program and what it does to support survivors of GBV

If you became aware that a woman in your neighborhood has been abused, what would you do? What if you know the abuser?

[Note to interviewer: Please probe to find out whether interviewee will become supportive or neglect the case and the reasons for doing whatever s/he has decided to do.]

Given all that we have discussed, have any of your thoughts or feelings changed about these issues based on what you have learned or experienced through your participation with the GBV Program?

4. Violence against Children

What can you tell me about violence against children in your community?

- In your opinion, what are some circumstances, if any, under which it is ok for a parent or other adult to hit or beat a child?
- From your experience, for what reasons/under what circumstances are adults most likely to hit or beat children?
- Other than beating, what else do parents in your community do to punish children who have made a mistake?

Are you aware of any incidents of sexual relations between a child (under age18) and an adult, in your community?

<u>Note to interviewer</u>: If yes, please probe on the following:

- What was the incident(s)?
- How did you hear about this?
- How often have you heard of such incidents? When did it/they happen?
- Could you describe the perpetrators of child sexual abuse? (With probing whenever something interesting is mentioned)

<u>Note to interviewer:</u> An example of an interesting issue might be that the respondent reports a neighbor's child was sexually abused recently by a family friend. Please ask to find out how, where, and when this took place and the circumstance and the actions taken by the child/parents or other community members to address it.

5. Response to GBV among children/minors

Have any local community measures been taken in addressing such incidents?

<u>Note to the interviewer</u>: If, yes, please probe on the details of the actions and when this took place.

What do families do in case of a sexual abuse incident in the family?

<u>Note to interviewer</u>: Please probe on details of measures taken including:

- whether reporting to police is among the options for addressing sexual violence against minors
- how follow-up takes place for any of the situations where a case is reported
- awareness of the GBV program and what it does to support sexually abused children and their families

If you became aware that a child in your neighborhood has been sexually abused, what would you do? What if you know the abuser?

<u>Note to interviewer:</u> Please probe to find out whether interviewee will become supportive or neglect the case and the reasons for doing whatever he has decided to do.]

In your opinion, what are some circumstances, if any, under which it is ok for an adult (someone over 18) to engage in sexual activity with a child or minor (under 18)?

Given all that we have discussed, have any of your thoughts or feelings changed about these issues based on what you have learned or experienced through your participation with the GBV Program?

6. Experience of GBV

Now I am going to ask you some questions about your own experiences with gender-based violence. Please remember that everything we talk about here today will be kept confidential, and that you do not need answer any questions/discuss any topics that you are not comfortable discussing.

Please describe any instances/forms of GBV, including sexual violence against children that you have directly witnessed or observed.

Note to interviewer, please probe: where was this? How long ago was this? What happened? Who was the victim? Who was the perpetrator? What was the outcome?

Have you ever personally experienced/been the victim of GBV, as an adult or a child? If so, what can you tell me about it?
Note to interviewer, please probe: where was this? How long ago was this? What happened? Who was the perpetrator? What was the outcome?

Have you ever engaged in GBV; that is, done any of these things to another person (either an adult or a child)? If so, what can you tell me about it?
Note to interviewer, please probe: Where was this? How long ago was this? Please tell me what happened

7. Knowledge of and experience with GBV services

I would like to finish up our discussion by asking your opinions about support in your community for people who have experienced GBV.

If a woman (or man, or child) experiences GBV, where can she/he go for help/support? Please describe to me the type of help/services that are offered.

Note to interviewer: Please ask for names of facilities and organizations.

Are these services easy to use? If yes, why is that? If not, please tell me what makes it difficult for people in your community to use these services?

Please also describe any types of services that are missing. Are there some parts of the community where no GBV services are available?

Do you know people who have used these GBV services? Have you personally used these services? If yes, what can you tell me about their/your experiences with these services?

Do you have any suggestions on how these programs could be improved? Are there other types of programs and services that are missing/needed? If so, what?

Wrap up

What else you would like to share with me regarding GBV in your community?

What else would you like to share with me about the GBV program?

Thank you very much for participating in this discussion.

Annex B-4

Health facility GBV register

English

CODES:

Column 3:
1 = Father & Mother
2 = Father only
3 = Mother only
4 = Father & step mother
5 = Mother & step father
6 = Uncle
7 = Aunty
8 = Brother
9 = Sister
10 = Another relative
11= Neighbour
12 = Alone
13 = Partner

Column 16:
1= Self
2 = Family
3 = Police
4 = Local Official
5 = School
6 = Welfare office
7 = NGO/FBO
8 = Good samaritan/neighbour
9 = Other Health facility
10 = Other
NA = Not first encounter

Column 17:
1= RCH
2 = CTC
3 = OPD
4 = IPD
5 = Dental
6 = Mental
7 = Other
NA = First encounter

Column 8:
1= Orphan
2 = Street Child
3 = Both
NA = Not orphan or street child

Column 15:
1 = Single/Child
2 = Married
3 = Widowed
4 = Divorced
5 = Separated

Column 26:
1= RCH
2 = CTC
3 = OPD
4 = IPD
5 = Dental
6 = Mental
7= Other
NA = Referals not given

TATHMINI GBV - FACILITY REGISTER FOR SERVICES FOR GENDER BASED VIOLENCE AND VIOLENCE AGAINST CHILDREN

THIS REGISTER CONTAIN THE CLIENT LOG FOR:
[ ][ ] Month [2][0][1][ ] Year

Department/Unit ______________________

Health Facility Name ______________________

District Name ______________________

Start New Register on 1st day of each calender month

PAGE _____ OF _____ PAGES FOR THIS MONTH

INSTRUCTIONS:
PLEASE COMPLETE REGISTER FOR ALL CLIENTS (ADULTS AND CHILDREN) SEEKING/RECEIVING GBV/VAC RELATED SERVICES IN YOUR DEPARTMENT.

If more than 10 clients are seen in this month, start a new page. Indicate the page number in the space above. Continue with consecutive numbering in column 1 (e.g. starting new page with 11, 21, 31, etc.). At the end of the month staple together all pages for the month for this department

1	2	3	4	5	6	7	8	9	10	11	12	13	14	15	16	17	18			19				20	21			22					23	24			25					26	27						28
S/N	Client Name	Who are you living with? - SEE CODES	Height (cms)	Weight (Kgs)	Main Source of income - Job	How many times have you given birth?	Vulnerable person? - SEE CODES	Name of street/village chairperson	Client ID or GBV Card #	Date of client visit (DD-MM-YY)	Place client stays Ward Village/Street	Sex of client: ME= Male ; KE = Female	Month & Year of birth (MM-YY)	Marital status - SEE CODES	If this is client's first encounter at this facility for GBV/VAC services. Cleint Refered to facility by - SEE CODES	If client previously was seen at this facility for GBV/VAC services write Dept last seen - SEE CODES	Type of exam conducted √ = Conducted H = Not conducted			Type of violence √ = Yes H = No				For sexual violence case. Cient arrived at the facility within 72 hours? - 1= YES, 2 = NO, 3 = Don't know	Forensic Exam conducted √ = Conducted H = Not conducted			Counselling provided √ = Provided H = Not provided					Medical tretament provided for injuries: 1= YES, 2 = NO, NA =No injuries	Test Performed √ = Performed H = Not performed			Propylaxis/ treatment given √ = Given H = Not given					Referrals made to other DEPARTMENT at this facility Write name of Dept client was referred to - SEE CODES	Referrals made to services outside the facility √ = Referrals made H = Referrals not made						Provider Initials
																	GBV screening and counselling	Assess physical state	Assess mental state	Sexual	Physical	Emotional	Neglect		Forensic Exam conducted	Evidence collected	PF3 form filled	Psycosocial	Pregnancy/Family Planning	HIV and HIV test	PEP adherence	Other		Pregnancy test	HIV test	STI test	Family Planning Method	Emergency Contraception	PEP for HIV	STI	Tetanus Toxoid		Psychosocial care	Police	Safe house or shelter	Clinical care at higher facility	Legal Services	Others	

Annex B-5.

Health Facility Audit Tool

Health Facility Audit for GBV Services

Part I. Interview with Facility Heads

GENERAL INFORMATION, INTRODUCTION, PERMISSION

Health Facility (name): _________________________________	Type of Facility: 1) Hospital 2)Health Centre
Facility ID No: ____	District: 1=Mbeya City 2=Mbeya Rural 3=Rungwe 4=Ileje 5=Chunya 6=Mbozi 7=Mbarali 8=Kyela
Date (dd/mm/yy) ___/___/___	Name of Interviewer: ____

INSTRUCTIONS TO INTERVIEWER: Heads of facility should be interviewed individually and in private when they have agreed to give us time on a particular day. Please make clear that you are seeking information on factors related to the facility's readiness and capacity to respond to gender-based violence (GBV) in order to improve services for survivors of GBV, but that you are not evaluating the performance of the individual facility or of its individual managers and providers.

**Read the following greeting to interviewee:**<br>*Hello…… My name is ………………………… I am representing Tathmini GBV, a project that is conducting an evaluation in Mbeya of comprehensive facility and community based activities to prevent and respond to GBV. As part of this work, we are collecting information on the staffing, services offered, infrastructure at facilities and practices of facility providers. We will be conducting this facility assessment now at baseline, again 6 months from now, and again at the end of the study (in approximately 18 months). I would like to ask you some questions about this facility.*<br><br>*There is no risk if you agree to participate in this assessment. Rather, it could benefit you by helping us to improve services in Tanzania. All the information that you give to me will be kept confidential; your name will not be used and you will not be identified in any way in any report of publication resulting from this assessment. Your current and future position at this facility will not be affected in any way. If you agree to participate, this interview should take approximately 90 minutes to complete. Your participation is absolutely voluntary and there is no penalty for refusing to take part. You are free to ask any questions; you may refuse to be in this assessment; you may refuse to answer any question in the interview; and you may stop the interview at any point.*
If you have any complaint about the way you have been treated during the interview or any possible harm, please contact:<br>*Professor Mainen Moshi, Director of Research and Publications, Muhimbili University of Health and Allied Sciences*<br>*Phone number: **+255 222 150 302/6.***<br>[Write down the name and phone number on a piece of paper and leave it with the interviewee]
Do you have any questions for me? 1= Yes 2= No<br>If yes, write down the questions and address the ones that you can.
Would you like to participate in this interview? 1= Yes 2= No

→ If the facility head agrees to continue, begin the interview with Section 1.

→ If the facility head does not agree to continue, tick below "refused interview," and if possible, provide a reason why the interview was refused. Thank him/her for his/her time.

Interview completed: 1= Yes, complete 2= No, incomplete 3= Refused interview<br><br>If interview refused (give reason):<br><br><br><br><br>Signature of interviewer: ____

Be sure to return HERE at the end of the interview to indicate whether the survey is complete.

1. CATCHMENT AREA, STAFFING, GBV SERVICES

Introduction and Definition of GBV

Read the following:

I'd like to begin by asking you about the services you currently provide for gender-based violence, or GBV. When I use the term "GBV" in this interview, I am referring to the definition in the Ministry of Health and Social Welfare, GBV management guidelines, 2011. The guidelines define GBV as "an umbrella term for any act, omission, or conduct that is perpetuated against a person's will and that is based on socially ascribed differences (gender) between males and females. In this context, GBV includes but is not limited to sexual violence, physical violence and harmful traditional practices, and economic and social violence. The term refers to violence that targets individuals or groups on the basis of their being female or male."

Some of the many examples of GBV include:

- *Sexual violence, including sexual exploitation/abuse and forced prostitution*
- *Physical violence by an intimate or dating partner*
- *Psychological abuse, including threats and intimidation*
- *Forced/early marriage*
- *Harmful traditional practices such as female genital mutilation, honor killings, widow inheritance, and others*

**GBV patient care and patient follow-up at this facility**
Do you have a protocol or systematic procedures for caring for GBV survivors? Please describe how you provide services.<br><br><br><br>Probe: Does this include systematic procedures for providing care to GBV survivors following their initial visit to this facility?

Interviewer: *I am going to ask you more details about the GBV services you provide, but I'd like to get some general information about the facility first.*

Q#	Question	Response	
101	What is the size of the population served by this facility?		
102a	What wards are served by this facility?	*List them:*	
102b	What towns/villages are served by this facility?	*List them:*	
103	How many staff work at this facility?<br>*Ask about each cadre below and write number in column (1); if none record "0"*<br>*When finished listing staff under column (1), go to Q104.*	**(1)**<br>**Number of staff**<br>(Don't know=888)	**(2)**<br>**Number who have completed national GBV training**<br>(Don't know=888)
	a) Obs/Gyn		
	b) Surgeon		
	c) Urologist		
	d) Psychiatrist		
	e) Psychologist		
	f) Pediatrician		
	g) Pathologists		
	h) Medical Doctor		
	i) Assistant Medical Officer (Psychiatrist)		
	j) Assistant Medical Officer		
	k) Clinical officer		
	l) Clinical officers (counselors)		
	m) Assistant Clinical Officer		
	n) Nursing officer		
	o) Nurse Counselors (VTC/PITC)		
	p) Nurse (psychiatrist)		
	q) Enrolled Nurses		
	r) Nurse midwives		
	s) Nursing assistants/Medical Attendants		

Q#	Question	Response	
	t) HIV/AIDS counselors		
	u) Laboratory technologist		
	v) Laboratory technicians		
	w) Laboratory Assistants		
	x) Health information officers/MTUHA		
	y) Pharmacists		
	z) Pharmaceutical technicians		
	aa) Pharmaceutical assistant		
	bb) Community health workers		
	cc) Social workers		
	dd) Social welfare officer		
	ee) Others (specify:______________________)		

Q#	Question	Response
104	Have any staff completed the 12-day national GBV training?	1= Yes<br>*Then ask which staff and Indicate in column (2) above the numbers by cadre*<br>2= No<br>8= Don't know
105	Have any staff received GBV training other than the 12-day national training?	1= Yes<br>2= No (skip to 201)<br>8= Don't know (skip to 201)
106	What organization or organizations provided that training?	*List all:*

2. STANDARD OPERATING PROCEDURES AND CONFIDENTIALITY for GBV clients

Q#	Question	Response
201	Do you have any written SOPs, protocols, or flow charts that remind providers how to care for a survivor of sexual violence or other kinds of GBV?	1= Yes [have some or all of these]<br>2= No [do not have any of these] (skip to Q204)<br>8= Don't know (skip to Q204)
202	Do you have the National Policy Guidelines for health sector response to and prevention of GBV 2011 and the National Management Guidelines for Health sector response to and prevention of GBV?	1= Yes, have both<br>2= Have national policy guidelines only<br>3= Have national management guidelines only<br>4= No, do not have either<br>8=Don't know

Q#	Question	Response
203	Do you have separate written SOPs, protocols or flow charts that remind providers how to care for child survivors of violence (VAC)?	1= Yes, have separate materials for VAC<br>2= No<br>8= Don't know
204	Does this facility have a system in place to protect patient confidentiality for GBV patients?	1= Yes<br>2= No (skip to Q206)<br>8= Don't know (skip to Q206)
205	If yes, please describe the system<br>***Probe:***<br>• *What does this facility write on?*<br>• *Patient's files?*<br>• *Use names, numbers or both?*<br>• *Some facilities use coding systems. For example, the facility staff assigns patients an identity code made up of letters and/or numbers to use on medical files and referral forms in place of the patient's name.*	*Record what the respondent says*:
206	How many staff at the facility has access to the identifying information of GBV clients?	No. with access:<br>888=Don't know
207	Are GBV client files kept in locked filing cabinets?	1= Yes<br>2= No (skip to Q209)<br>8= Don't know (skip to Q209)
208	If yes, how many staff has access to the locked filing cabinets?	No. with access:<br>888=Don't know
209	An informed consent process involves counseling the patient on what steps the provider recommends taking, asking for and addressing the patient's questions and concerns, and receiving her/his agreement to proceed. **Are providers at this facility required to obtain informed consent from the patient <u>before</u>** …	
	a) Conducting a clinical examination?	1=Yes<br>2=No<br>8=Don't know
	b) Collecting forensic evidence?	1=Yes<br>2=No<br>8=Don't know
	c) Performing an HIV test?	1=Yes<br>2=No<br>8=Don't know
	d) Passing on any of his or her information to another facility when referring a patient?	1= Yes<br>2= No<br>8= Don't know

Q#	Question	Response
210	Are providers and other staff at this facility required to sign a Code of Conduct that addresses patient confidentiality?	1= Yes, providers and staff<br>2= Yes, providers only<br>3= Yes, staff only<br>4= No [no one is required]<br>8= Don't know
211	Does this facility record and report the numbers of patients who come in as a result of GBV?	1= Yes<br>2= No (skip to 301)<br>8= Don't know (skip to 301)
212	If yes, which register or form is used?	*Record name of form:*
213	To whom does the facility report the numbers of patients who come in as a result of GBV?	
	a) Government: DMO, RMO, or MoH	1= Yes<br>2= No<br>8= Don't know
	b) NGO(s)	1= Yes, specify: ______________________<br>2= No<br>8= Don't know
	c) Other	1= Yes, specify: ______________________<br>2= No<br>8= Don't know

3. SERVICES OFFERED to GBV CLIENTS

Interviewer: *I am now going to list components of GBV services and I'd like you to tell me if these are currently offered to GBV clients at this facility. If you don't know, please say "don't know."*

	For each service below, mark an X in the appropriate column	**1= Yes, always offered**	**2= Yes,** sometimes offered	**3 = No, never offered**	**4= Don't know**
301	I'd like to begin with **Medical Services.** Do you…				
	a) Receive and triage GBV clients for appropriate care				
	b) Provide initial counseling, ensuring confidentiality<br>*If counseling is offered, but it's not confidential, note that.*				
	c) Take history using a checklist				
	d) Undertake exam using a checklist				

	For each service below, mark an X in the appropriate column	1= Yes, always offered	2= Yes, sometimes offered	3 = No, never offered	4= Don't know
	e) Request and perform baseline investigations				
	f) Manage minor injuries				
	g) Manage major injuries				
	h) Offer HIV test at initial visit				
	i) Provide Post Exposure prophylaxis (PEP)				
	j) Provide Emergency Contraception (EC)				
	k) Provide Tetanus Toxoid Vaccination (TT vaccination)				
	l) Treat STIs				
	m) Provide Post Abortion Care				
	n) Conduct HIV test 3-months and 6 months (post-sexual rape)				
	o) Manage HIV and AIDS				
	p) Manage anemia				
	q) Offer Obstetric Care				
302	Next I'll ask you about **Psychosocial Services.** Do you…				
	a) Provide Basic counseling/reassurance				
	b) Provide advanced counseling (e.g., for anxiety, trauma, depressive disorders, etc)				
	c) Provide PEP adherence counseling				
	d) Provide social services (e.g., shelter, basic needs, transport, etc)				
	e) Link the survivor with other GBV services				
	f) Follow up with the survivor				
303	With regard to **Forensic Services,** do you…				
	a) Document and collect history and findings for medico-legal purposes				
	b) Complete PF3 forms				
	c) Collect samples and send them for DNA analysis				
	d) Serve as a factual/expert witness in the court of law				
304	With regard to **Referrals and Linkages Services,** do you				
	a) Refer complications to other health facilities that have the capabilities				

<table>
<tr><th colspan="2">For each service below, mark an X in the appropriate column</th><th>1= Yes, always offered</th><th>2= Yes, sometimes offered</th><th>3 = No, never offered</th><th>4= Don't know</th></tr>
<tr><td></td><td>b) Educate, orient, and provide information about other GBV services</td><td></td><td></td><td></td><td></td></tr>
<tr><td></td><td>c) Consult a qualified person with a legal mandate to serve as an expert witness in court during the initial encounter with a GBV survivor</td><td></td><td></td><td></td><td></td></tr>
<tr><td>305</td><td colspan="5">Is the facility prepared to make the following types of referrals for survivors of GBV, if needed and desired by the survivor?</td></tr>
<tr><td></td><td>a) Referral for legal assistance?</td><td colspan="4">1= Yes, where:____
2= No
8= Don't know</td></tr>
<tr><td></td><td>b) Referral to a shelter or safe house?</td><td colspan="4">1= Yes, where:____
2= No
8= Don't know</td></tr>
<tr><td></td><td>c) Referral for psychosocial counseling?</td><td colspan="4">1= Yes, where:____
2= No
8= Don't know</td></tr>
<tr><td></td><td>d) Referral for community reintegration?</td><td colspan="4">1= Yes, where:____
2= No
8= Don't know</td></tr>
<tr><td></td><td>e) Any other type of service?</td><td colspan="4">1= Yes, where/for what service:
____
____
2= No
8= Don't know</td></tr>
</table>

4. FACILITY NEEDS

Q#	Question	Response
401	Do providers at this facility have the equipment, supplies, and infrastructure necessary to provide adequate medical care to GBV survivors?	1= Yes (skip to 403)<br>2= No<br>8= Don't know (skip to 403)
402	If no, what additional equipment, supplies, and infrastructure are needed to provide adequate care to GBV survivors?<br>*List all mentioned*	

403	Do providers at this facility have the knowledge and skills necessary to provide adequate care to GBV survivors?	1= Yes (skip to Q405)<br>2= No<br>8= Don't know (skip to Q405)
404	If no, what additional knowledge and skills do you feel providers need to provide adequate care to GBV survivors?<br>*List all mentioned*	
405	What are the challenges that this facility faces in responding to GBV?<br>*List all mentioned*	
406	Do you have suggestions for improving this facility's response to GBV?<br>*List all mentioned*	
407	Do you have any questions for me?<br>*List all mentioned*	

Closing
Interviewer: *Thank you very much for answering these questions. We appreciate your time and honesty.*

Part II Observational checklist for GBV Services

Instructions for Interviewer: For each item, indicate your observation as follows:
Y = Yes, item is available
N = No, item is not available
DK= Don't know (for those questions asked of Facility Head)
NA = Not applicable

Verify your observations with the Facility Head as you walk through the facility.

**General**<br>*Visit and record your observations for each department. If the department doesn't exist, draw a line down the column.*		**OPD**	**RCH**	**CTC**	**IPD**	**Mental Health**
1	Is there a private room (with 4 walls and a door) for examination of GBV survivors? (*Skip to Q3 if response is No or DK)*					
2	Is the private room available 24 hours? (*answered by the Facility Head, not observation*)					
3	Is there a toilet in the same corridor as the examination room?					
4	Is there a bath/shower available for use by the rape survivor after the examination?					
5	Is there hot water in the health care facility?					
6	Is the bed linen changed after each patient is examined? (*Answered by the Facility Head, not observation*)					
In the rooms in the departments where GBV examinations routinely take place:		**OPD**	**RCH**	**CTC**	**IPD**	**Mental Health**
7	Is there a working telephone?					
8	Is there an examination couch?					
9	Is there a working angle lamp?					
10	Is there a speculum?					
11	Is there a vaginal retractor?					
12	Is there a colposcope?					
13	Are there examination gloves?					
14	Is there a sharps container?					
15	Is there a lockable cupboard for the storage of forensic/medico-legal evidence?					

**General**<br>*Visit and record your observations for each department. If the department doesn't exist, draw a line down the column.*		**OPD**	**RCH**	**CTC**	**IPD**	**Mental Health**
16	Is there a lockable medical supply cabinet?					
17	Is there a screen for examination of patients?					
18	Is there a mackintosh on the examination bed?					
19	Are there patient gowns?					
20	Are there sanitary towels?					
21	Is there emergency clothing?					
22	Is there a consent form for the examination?					
23	Is there a pre-packaged rape kit?					
24	Are there swabs?					
25	Are there vaginal swabs?					
26	Are there blood tubes?					
27	Are there urine bottles?					
28	Are there sheets of paper?					
29	Are there paper bags?					
30	Are there written SOPs for management of GBV survivors?					
31	Are there flow charts?					
32	Is there a copy of the National Management Guidelines for Health sector response to and prevention of GBV?					
33	Are there leaflets and hand-outs on support services for rape survivors, such as NGOs?					
34	Are there forms to record and report the numbers of patients who come in as a result of GBV?					

**In the Pharmacy/Dispensing Area**		**Indicate: Y=Yes N=No**
35	Is there a pregnancy test kit?	
36	Is there a HIV rapid test?	
37	Are there emergency contraceptives?	
38	Are there STI prophylaxis/treatment?	
39	Is there post-exposure prophylaxis to prevent HIV?	
40	Is there analgesia?	
41	Are there tranquilizers?	
42	Are there anti-emetics?	

**Laboratory**		**Indicate: Y=Yes N=No**
43	Pregnancy test kits	
44	Microscopes	
45	Culture and sensitivity testing	

46	HIV test kits	
47	DNA tests	
48	VDRL	
49	Hepatitis B surface antigen tests	
50	FBP	
51	Blood grouping and Cross matching	
52	Blood chemistry	

NOTE TO INTERVIEWER: PLEASE BE SURE TO RETURN TO **PAGE 2**, and INDICATE WHETHER THE SURVEY WAS COMPLETED. BE SURE YOU SIGN THE SURVEY AND COMPLETE ALL GENERAL INFORMATION ON PAGE 1.

Annex B-6.

Key informant interview guide: Service providers

(English)

ID: [S] [P] - [ ] [ ] [ ] [ ] [ ] [ ]

Health Facility Assessment Part III: Interview with Service Providers

Health Facility (name): ________________________ Date (dd/mm/yy) __/__/__

Type of Facility(*mark with* X) : ____ Hospital ____Health Centre

Interviewer ID: [ ] [ ] [ ] [ ] START TIME: [ ] [ ]:[ ] [ ] END TIME [ ] [ ] :[ ] [ ]

Data collection phase (*mark with* X): ____ Baseline _____ 6-month follow-up ______ Endline

GENERAL INFORMATION, INTRODUCTION, PERMISSION

INSTRUCTIONS TO INTERVIEWER

1) Introduce yourself

Hello…... My name is ....…. I am representing Tathmini GBV, a project funded by USAID that is conducting an evaluation in Mbeya of comprehensive facility- and community-based interventions to prevent and respond to gender-based violence (GBV). I am seeking information on factors related to the facility's readiness and capacity to respond to GBV in order to improve services for survivors of GBV. I am not evaluating the performance of the individual facility or of its individual managers and staff. This is a continuation of the facility assessment that we started last February when we interviewed the facility manager and conducted an inventory of departments. This portion includes interviews with service providers. It is the last component of the facility assessment. The study team is conducting this facility assessment at baseline (now- before the interventions begin), again 6 months from now, and again at the end of the study, which will be in approximately 18 months.

2) Obtain the provider's informed consent: Read the informed consent, and if s/he agrees, ask her/him to sign it. If s/he chooses not to sign the informed consent form, DO NOT PROCEED with the interview. If provider consents, look for a private place to interview her/him.

Before you start the interview, ask provider if he/she has any questions for you: ___ Yes ___ No

If yes, write the questions below. Answer the ones you can and record your answers. If there are questions that you can't answer, tell the provider that you will forward them to the study coordinator, who will get back to her/him with answers.

→ Begin the interview by asking Q.1.0. For each item, please answer each question by circling the correct response code or writing in the information.

→ Please complete the box below at the end of the interview to indicate whether the survey is complete.

Interview completed: ___Yes, complete ___ No, incomplete

If interview is incomplete, give reason(s): ____

Signature of interviewer: ______________________________

SECTION 1: PROVIDER'S BACKGROUND			
**Q#**	**Question**	**Response**	**Skip patterns**
**1.0**	Sex<br><br>**<u>Observe provider sex; don't ask.</u>**	1= Male<br>2= Female	
Interviewer: *Thank you for agreeing to participate. First, I would like to ask you few questions about yourself.*			
**1.1**	e) What is your job title?<br><br>**<u>Note: If not a specialist, skip to 1.2</u>**	1=Specialist →<br>2= Medical Officer<br>3=Assistant Medical Officer<br>4=Clinical officer<br>5=Nursing officer<br>6=Nurse Midwife<br>7=Pharmacist<br>8=Lab technologist<br>9=Lab Assistant<br>10=HIV Counselor<br>11= Social welfare<br>88=Other Specify___________	**Ask 1.1b**<br><br>**skip to 1.2**
	f) If you are a specialist, what is your specialty?	1=OBGyn<br>2=Surgeon<br>3=Pediatrician<br>4=Orthopedics<br>5=Urologist<br>6=Physician/Internal Medicine<br>88=Other Specify___________	
**1.2**	In what year did you begin working in health services?	[ ][ ][ ][ ] Year<br>8888=Don't remember/Don't know	
**1.3**	In what year did you begin working in this facility?	[ ][ ][ ][ ] Year<br>8888=Don't remember/Don't know	

SECTION 2: PROVIDERS KNOWLEDGE AND ATTITUDES ON GBV

Interviewer: *Now I would like to move and ask you questions regarding your knowledge on Gender Based Violence*

Q#	Question	Response	Skip pattern
**2.0**	a) Do you know what the term "Gender Based Violence" means?	1=Yes<br>2=No →	**Read the GBV definition below**
	b) If YES, could you please describe to me what Gender Based Violence means?		

<u>Read the definition below regardless of the response to Q 2.0</u>.

Definition of GBV:

When I use, the term gender-based violence, or GBV, in this interview, I am referring to the definition in the Ministry of Health and Social Welfare, GBV management guidelines, 2011. The guidelines define GBV as "an umbrella term for any act, omission, or conduct that is perpetrated against a person's will and that is based on socially ascribed differences (gender) between males and females. In this context, GBV includes but is not limited to sexual violence, physical violence and harmful traditional practices, and economic and social violence. The term refers to violence that targets individuals or groups on the basis of their being female or male."

Some examples of GBV include:

- *Sexual violence, including sexual exploitation/abuse and forced prostitution*
- *Physical violence by an intimate or dating partner*
- *Psychological abuse, including threats and intimidation*
- *Forced/early marriage*
- *Harmful traditional practices such as female genital mutilation, honor killings, widow inheritance, and others*

Q#	Question	Response		
2.1	In your opinion, is a husband justified in hitting or beating his wife in the following situations:	**YES**	**NO**	**DON'T KNOW**
	f. If she goes out (of the house) without telling her husband/partner?	1	2	8
	g. If she neglects the children?	1	2	8
	h. If she argues with her husband/partner?	1	2	8
	i. If she refuses to have sex with her husband/partner?	1	2	8
	j. If she burns the food?	1	2	8

Q#	Question	Response	Skip pattern
**2.2**	Imagine that a woman comes into the facility and reports that she was raped, but she does not want to disclose what happened to anyone except you, her medical care provider. In a private meeting, her husband asks you what happened.<br>In your opinion, should you tell her husband that she was raped?	1=Yes<br>2=No<br>8=Don't know	
**2.3**	a) If a man has sexual intercourse with a woman who says "no" but she does not physically resist, has he committed rape?	1=Yes<br>2=No<br>8=Don't know	
	b) If a husband has sexual intercourse with his wife who says "no" but she doesn't resist, has he committed rape?	1=Yes<br>2=No<br>8=Don't know	
**2.4**	a) How many times, if any, have you attended a training workshop on providing services for survivors of GBV?<br>IF PROVIDER SAYS "Don't Know," ASK HER TO GIVE HER BEST ESTIMATE	______ # of times<br>If 0 (no training) ⟶	**Skip to Q2.5**
	b) Which organization or institution organized/led the most recent training you attended?	1=Ministry of Health<br>2=Walter Reed Program (WRP)/HJMRI<br>3=MOH and WRP/HJMRI<br>4=Other, specify:<br>______________<br>8=Don't know	
	c) In what year did that <u>most recent</u> training take place?	**[ ][ ][ ][ ]** Year<br>8888=Don't remember/Don't know	
	d) How many days was the <u>most recent</u> the training?	**[ ][ ]** # of days<br>88=Don't remember/Don't know	

	e) Were the following topics covered in the training(s)? **<u>Read each topic, circle the answer the provider gives.</u>**	**Yes**	**No**	**Don't know**
	1) Definition of GBV	1	2	8
	2) Causes of GBV	1	2	8
	3) Consequences of GBV	1	2	8
	4) International standards for the care of GBV survivors	1	2	8
	5) National SOPs for the care of GBV survivors	1	2	8
	6) Providing psychosocial support/reassurance to GBV survivors	1	2	8
	7) Clinical management of rape	1	2	8
	8) Collection & documentation of clinical evidence of rape	1	2	8
	9) Confidentiality	1	2	8
	10) Consent	1	2	8
	11) Legal requirements for providers	1	2	8
	12) Special considerations when GBV survivors are children/minors	1	2	8
	13) Other *(specify)*: ____________________	1	2	8
**2.5**	How would you rate your familiarity with Tanzania laws on GBV?<br><br>**<u>Read options</u>**	1=Very good<br>2=Good<br>3= Average<br>4=Poor<br>5=Very Poor		
**2.6**	What Tanzania laws on GBV do you know?			

SECTION 3: STANDARD OPERATING PROCEDURES AND CONFIDENTIALITY

Interviewer: *Now I am moving into another set of questions - about standard operating procedures, or SOPs, and confidentiality - with GBV services in mind.*

Q#	Question	Response	Skip pattern
**3.0**	a) Are there specific providers in this facility who are assigned to respond to sexual violence and other forms of GBV?	1=Yes<br>2=No →<br>8= Don't know →	 **skip to 3.1**<br>**skip to 3.1**
	b) If YES, are you one of those providers?	1=Yes<br>2=No<br>8= Don't know	
**3.1**	1) Do you know the roles and tasks a service provider needs to carry out when a GBV survivor comes to the facility for medical care?	1=Yes<br>2=No →<br>8= Don't know →	 **skip to 3.3a**<br>**skip to 3.3a**
	2) If YES, can you please describe those roles and tasks?<br>**<u>Do not read. If R mentions the role/task, circle 1. If not mentioned, circle 2.</u>**		

	Mentioned	Not mentioned
1) Ensure safety of the survivor	1	2
2) Ensure confidentiality	1	2
3) Respect, wishes, rights and dignity of survivor	1	2
4) Obtain consent for specific procedures and services	1	2
5) Show empathy, be discrete, friendly and compassionate	1	2
6) Provide correct information to the survivor	1	2
7) Handle evidence according to guidelines to safeguard chain of evidence	1	2
8) In collaboration with other service providers ensure physical protection and safety and minimize further suffering	1	2
9) Keep written information safe and confidential at all times	1	2
10) Provide counseling and referral for further medical care and services	1	2
11) Serve as expert witness in court if required	1	2
12) Conduct advocacy meetings on prevention and response in the community	1	2
13) Monitor and evaluate GBV and VAC services	1	2
14) Liaise with other sectors to promote prompt management	1	2
15) Sensitize other health and social workers on prevention and response to GBV	1	2
16) Provide psychosocial support	1	2

	17) Promote continuity of care and follow up	1	2
	18) Other, specify: ____________________	1	2

Q#	Question	Response	
3.2	How did you learn what to do when a GBV survivor of comes to the facility for medical care?<br><br>**Do not read. If R mentions the item, circle 1. If not mentioned, circle 2.**	**Mentioned**	**Not mentioned**
	a. Through written SOPs	1	2
	b. Through written job description	1	2
	c. Through verbal explanation by manager or other person	1	2
	d. From training/other external instruction	1	2
	e. I learned on my own	1	2
	f. Other, specify: ____________________	1	2

Q#	Question	Response	Skip patterns
3.3	a) Do you have any written SOPs, protocols, or flow charts on how to care for a GBV survivor?	1=Yes<br>2=No →<br>8= Don't know →	 **skip to 3.4a**<br>**skip to 3.4a**
	b) If YES, kindly show me the SOPs, protocols, flow charts or other similar documents that guide care of GBV survivors<br>**Circle appropriate response for each item below**	**Shown**	**Not shown**
	1) SOPs	1	2
	2) National Policy Guidelines for health sector response to and prevention of GBV 2011	1	2
	3) National Management Guidelines for Health sector response to and prevention of GBV	1	2
	4) Flow charts	1	2
	5) Others, specify: ____________________	1	2
3.4	I'd now like to ask you some questions about what you may do at this facility to protect the confidentiality of GBV clients.		
	a) Please describe the record system the facility uses to document and store information about GBV clients.		
	b) Do you think this record system keeps the client information confidential?	1=YES<br>2= NO<br>8= Don't know	

	c) Are GBV client files kept in locked filing cabinets?	1= Yes<br>2= No →<br>8= Don't know →	 **skip to 3.5**<br>**skip to 3.5**
	d) If files are kept in locked cabinets, who has access/ permission to access the files? **Read the options below**.		
	1) GBV focal person?	1= Yes 2= No 8= Don't know	
	2) Facility Manager?	1= Yes 2= No 8= Don't know	
	3) Clinicians?	1= Yes 2= No 8= Don't know	
	4) Registry/records staff?	1= Yes 2= No 8= Don't know	
	5) Social Workers?	1= Yes 2= No 8= Don't know	
	6) Others, specify ___________	1= Yes 2= No 8= Don't know	

Q#	Question	Response		
**3.5**	An informed consent process involves counseling the client on steps the provider recommends taking, asking for and addressing the client's questions and concerns, and receiving her/his agreement to proceed.<br>**Are providers at this facility required to obtain informed consent from the client <u>before</u>** …			
	**<u>Read each item. Circle the response that is given.</u>**	**Yes**	**No**	**Don't know**
	a) Conducting a clinical examination?	1	2	8
	b) Collecting forensic evidence? (if service not offered, draw line across row)	1	2	8
	c) Performing an HIV test?	1	2	8
	d) Passing on any of his or her information to another facility when referring a client?	1	2	8
	e) Other specify ___________________________	1	2	8
	f) Other specify ___________________________	1	2	8

Q#	Question	Response	Skip patterns
**3.6**	Have you ever signed a code of conduct to protect client confidentiality since you started working here?	1= Yes<br>2= No<br>8= Don't know	
**3.7**	a) Does this facility record and report the numbers of clients who come in as a result of GBV (monitoring data)?	1= Yes<br>2= No →<br>8= Don't know →	 **skip to 3.8**<br>**skip to 3.8**
	b) If yes, which register or form is used to record GBV clients?	1 = Tathmini GBV Facility Register<br>2= GBV Register from MOHSW guidelines	

		3= Both Tathmini and MOHSW registers<br>8 = Other, Specify:<br>______________________________
**3.8**	Is a GBV client given the option of being attended by a male or female service provider—should s/he have a preference for the sex of the provider?	1= Yes, always<br>2= Yes, sometimes<br>3= No<br>8= Don't know

SECTION 4: SERVICES OFFERED TO GBV CLIENTS

Interviewer: *Now I am moving into another set of questions, which are about services offered to GBV clients.*

Q#	Question	Response			
**4.0**	Does this facility offer services for clients (adults and children) who arrive reporting:<br><br>**Read each item and response options. Circle response given.**	**Yes, services always available**	**Yes, services sometimes available**	**No, never offered**	**Don't know**
	r) Rape	1	2	3	8
	s) Other forms of sexual violence (for example, abusive sexual contact or touching of any person without his or her consent; or forced prostitution)	1	2	3	8
	t) Gender-based physical violence	1	2	3	8
	u) Emotional violence	1	2	3	8
	v) Neglect (of children)	1	2	3	8
	w) Other, specify: ____________________	1	2	3	8
	x) Other, specify: ____________________	1	2	3	8
	y) Other, specify: ____________________	1	2	3	8

Q#	Question	Response	
**4.1**	Would you please describe the steps or actions you would take if a client who had just been raped arrived at this facility?<br>**Do not read the responses. If R mentions the item, circle 1. If not mentioned, circle 2.**	**Mentioned**	**Not mentioned**
	a) Provide psychosocial support/comfort the client (e.g., explaining that they are not at fault for experiencing GBV)	1	2
	b) Assure the client of confidentiality	1	2
	c) Inform client of his/her right to accept or refuse any of the services offered	1	2
	d) Refer the client to another provider at the same facility for examination and care	1	2
	e) Ask the client to describe what happened	1	2
	f) Ask the client about any symptoms and relevant medical history (e.g., HIV status)	1	2
	g) Perform a physical examination	1	2
	h) Treat any wounds or injuries, if applicable	1	2
	i) Test for pregnancy, if applicable	1	2
	j) Prescribe emergency contraception, if applicable	1	2
	k) Offer HIV test, if applicable	1	2
	l) Provide HIV post-exposure prophylaxis, if applicable	1	2
	m) Test for other STIs, if applicable	1	2
	n) Treat STIs, if applicable	1	2
	o) Administer other vaccines, if applicable (e.g., tetanus, Hepatitis B)	1	2
	p) Inform the client of his or her legal right to press charges against the perpetrator, if applicable	1	2
	q) Complete a medical certificate (PF3 form) documenting the rape, according to the client's wishes.	1	2
	r) Discuss client's safety (e.g., if they are in danger of continuing GBV)	1	2
	s) In cases of domestic violence, ask whether children in the family are at risk of violence	1	2
	t) Assess whether the client may be at risk of depression or suicide	1	2
	u) Refer the client for other services, according to the client's needs and wishes	1	2
	v) Collect forensic evidence, if applicable	1	2
	w) Other (specify): ____________________	1	2
	x) Other (specify): ____________________	1	2
	y) Other (specify): ____________________	1	2

Q#	Question	Response	
**4.2**	Would you please describe the actions you would take if a client who had experienced physical gender-based violence arrived at this facility?<br>**Do not read the responses. If R mentions the item, circle 1. If not mentioned, circle 2.**	**Mentioned**	**Not mentioned**
	a) Provide psychosocial support/comfort the client (e.g., explaining that they are not at fault for experiencing GBV)	1	2
	b) Assure the client of confidentiality	1	2
	c) Inform client of his/her right to accept or refuse any of the services offered	1	2
	d) Refer the client to another provider at the same facility for examination and care	1	2
	e) Ask the client to describe what happened	1	2
	f) Ask the client about any symptoms and relevant medical history (e.g., HIV status)	1	2
	g) Perform a physical examination	1	2
	h) Treat any wounds or injuries, if applicable	1	2
	i) Offer HIV test, if applicable	1	2
	j) Establish if sexual violence was experienced	1	2
	k) Administer other vaccines, if applicable (e.g., tetanus, Hepatitis B)	1	2
	l) Inform the client of his or her legal right to press charges against the perpetrator, if applicable	1	2
	m) Complete a police certificate (PF3 form), according to the client's wishes.	1	2
	n) Discuss client's safety (e.g., if they are in danger of continuing GBV)	1	2
	o) In cases of domestic violence, ask whether children in the family are at risk of violence	1	2
	p) Assess whether the client may be at risk of depression or suicide	1	2
	q) Refer the client for other services, according to the client's needs and wishes	1	2
	r) Other (specify): ______________________________	1	2
	s) Other (specify): ______________________________	1	2
	t) Other (specify): ______________________________	1	2

Q#	Question	Response	Skip patterns
**4.3**	a) Are you familiar with the approach to providing emergency contraceptive services to someone who has been raped?	1= Yes<br>2= No →	**skip to 4.3c**
	b) If yes, please explain the steps.<br>**<u>Do not read the responses. If R mentions the item, circle 1. If not mentioned, circle 2. If provider gives incorrect response, record under Other.</u>**	**Mentioned**	**Not mentioned**
	1) Ask a contraceptive method she is currently using	1	2
	2) Test for pregnancy	1	2
	3) If pregnant, provide or refer to ANC and PMTCT services	1	2
	4) If not pregnant, provide EC within 120 hours (5 days)	1	2
	5) Ask her to come back for pregnancy test again after 6 weeks	1	2
	6) Other, specify: ______________________	1	2
	7) Other, specify: ______________________	1	2
	8) Other, specify: ______________________	1	2
	c) Have there been any stock outs of emergency contraception over the past three months?	1= Yes<br>2= No<br>8= Don't know	
	d) Have there been any stock outs of pregnancy test kits over the past three months?	1= Yes<br>2= No<br>8= Don't know	
**4.4**	a) Are you familiar with the approach to providing HIV PEP to someone who has been raped?	1= Yes<br>2= No →	**skip to 4.4c**
	b) If yes, please explain the steps or actions to take.<br>**<u>Do not read the responses. If R mentions the item, circle 1. If not mentioned, circle 2.</u>**	**Mentioned**	**Not mentioned**
	1) Provide PEP within 72 hours	1	2
	2) Provide counseling before and after HIV testing	1	2
	3) If HIV positive, provide referral to CTC	1	2
	4) If HIV negative, provide PEP according to the guideline	1	2
	5) Provide brief explanation of PEP, it's positive & negative effects	1	2
	6) Other, specify: ______________________	1	2

Q#	Question	Response	Skip patterns
	7) Other, specify: ______________________________	1	2
	c) Have there been any stock outs of HIV PEP over the past three months?	1= Yes<br>2= No<br>8= Don't know	
	d) Have there been any stock outs of HIV test kits over the past three months?	1= Yes<br>2= No<br>8= Don't know	

Q#	Question	Response
**4.5**	a) Have you ever taken samples for forensic evidence for a GBV client?	1= Yes<br>2= No<br>8= Don't know
	b) Have you ever filled out a police certificate (PF3 form) for a client to use as evidence that she or he experienced physical or sexual violence from an intimate partner?	1= Yes<br>2= No<br>8= Don't know
	c) Is this facility consistently stocked with the materials to take samples for medico-legal purposes (that is, medical evidence collected for legal purposes)?	1= Yes<br>2= No<br>8= Don't know
**4.6**	a) Does this facility provide PITC (provider initiated testing and counseling for HIV) services?	1= Yes<br>2= No<br>8= Don't know
	b) Does this facility provide HIV couples counseling?	1= Yes<br>2= No<br>8= Don't know
**4.7**	Does this facility refer GBV clients to other services if they are needed and can't be provided at this facility? For example… **<u>Read each type of referral below</u>.**	
	a) Referral to a higher level health facility?	1= Yes, where:____________________<br>______________________________<br>2= No<br>8= Don't know

Q#	Question		Response
	b) Referral for legal assistance?		1= Yes, where:______________ ____________________ 2= No 8= Don't know
	c) Referral to a shelter or safe house?		1= Yes, where:______________ ____________________ 2= No 8= Don't know
	d) Referral for psychosocial counseling?		1= Yes, where:______________ ____________________ 2= No 8= Don't know
	e) Other, Specify ____________________		1= Yes, where:______________ ____________________ 2= No 8= Don't know
	f) Other, Specify ____________________		1= Yes, where:______________ ____________________ 2= No 8= Don't know

SECTION 5: SEXUAL VIOLENCE AGAINST CHILDREN

Interviewer: *Now I would like to move and ask you some questions about services provided at the facility for children who experience violence.*

Q#	Question	Response	Skip pattern
**5.0**	*a)* Do you know the meaning of the term "Child Sexual Violence"?	1=Yes<br>2=No →	**Read the definition below**
	b) If YES, could you please tell me the definition?		
	**<u>Read the definition below regardless of the response to Q 5.0</u>.**		

	**CHILD SEXUAL VIOLENCE Definition:**<br>Child sexual violence is the involvement of a child (in Tanzania, defined as a person under the age 18) in sexual activity that he or she does not fully comprehend, is unable to give informed consent to, or for which the child is not developmentally prepared and cannot give consent, or that violates the laws or social taboos of society. Child sexual abuse is evidenced by this activity between a child and an adult or another child who by age or development is in a relationship of responsibility, trust, or power, the activity being intended to gratify or satisfy the needs of the other person.<br><br>This may include but is not limited to:<br>• the inducement or coercion of a child to engage in any unlawful sexual activity,<br>• the exploitative use of a child in prostitution or other unlawful sexual practices, and<br>• the exploitative use of children in pornographic performance and materials. (WHO 1999)

Q#	Question	Response		
5.1	I'm going to read some statements to you and I'd like you to tell me if you agree, partially agree, or do not agree.	AGREE	PARTIALLY AGREE	DO NOT AGREE
	a) A child is sexually abused only when sexual intercourse has taken place	1	2	3
	b) When a child is sexually abused, the abuser is rarely a family member	1	2	3
	c) Only girls are sexually abused	1	2	3
	d) It is not possible for children under 10 years of age to experience sexual abuse	1	2	3
	e) Children from reputable families do not experience sexual abuse	1	2	3

Q#	Question	Response	Skip Pattern
**5.2**	Imagine that a girl 14 years of age comes into the facility with her mother and reports that she is pregnant. Would you (or another appropriate service provider) conduct an examination to assess whether she may have experienced sexual violence?	1=Yes<br>2=No<br>8=Don't know	
**5.3**	If a girl wears a short dress that leaves her body parts half naked, is a man justified in forcing her to have sexual intercourse?	1=Yes<br>2=No<br>8=Don't know	
**5.4**	a) Have you ever been trained to care for children who have experienced sexual violence?	1= Yes<br>2= No →<br>8= Don't know	**skip to 5.5**
	b) Which organization or institution organized/led the most recent training you attended?	1=Ministry of Health<br>2=Walter Reed Program/HJMRI<br>3= MOH and WRP/HJMRI<br>4=Other, specify: ____________<br>8=Don't know	

Q#	Question	Response	Skip Pattern
**5.5**	a) Are cases of sexual violence among children seen at this facility?	1= Yes<br>2= No →<br>8= Don't know	**skip to 5.6**
	b) Based on your experience of providing services here, what is the most common age of children who are seen here for sexual violence?<br><br>RECORD WHAT PROVIDER SAYS:<br>____________________________________	1= Under 5 years<br>2= 5-9 years<br>3=10-14 years<br>4=15-18 years<br>8=Don't know	
	c) Based on your experience, what are some of the complaints brought by children who have experienced **sexual** violence? What is the most common complaint? **Record what the respondent says.**		

Q#	Question	Response	
**5.6**	How would you recognize that a child has experienced sexual violence?<br><br>**Do not read the responses. If R mentions the item, circle 1. If not mentioned, circle 2.**	**Mentioned**	**Not Mentioned**
	a) Injuries in reproductive parts/private parts	1	2
	b) Watery discharge in private parts	1	2
	c) Not being able to hold urine or stool	1	2
	d) Pain when urinating	1	2
	e) PV bleeding	1	2
	f) Urinary tract infection	1	2
	g) STIs and PIDs	1	2
	h) Child being pregnant	1	2
	i) Delayed milestones	1	2
	j) Poor performance in school	1	2

	k) Other: Specify<br>____	1	2
	l) Other: Specify:<br>____	1	2
	m) Other: Specify:<br>____	1	2
**5.7**	Based on your experience, what are the most important actions to take to care for a child who has experienced sexual violence?<br>**Do not read the responses. If R mentions the item, circle 1. If not mentioned, circle 2.**	**Mentioned**	**Not mentioned**
	a) Ensure a child-friendly management and age-appropriate environment.	1	2
	b) Ensure that a parent/guardian is present at all times if the child so wishes (while also ensuring that the parent/guardian does not present a threat to the child).	1	2
	c) Always prepare the child on what to expect, including ensuring that the child understands what is going to happen.	1	2
	d) For young children below age 18 years, obtain the parent/guardian's consent on their behalf.	1	2
	e) Ensure safeties' of the child by placing her/him to the fit person or institution	1	2
	f) Ensure protection of the child when in the legal systems as a witness or victim	1	2
	g) Provide psychosocial support to the child	1	2
	h) Ensure confidentiality	1	2
	i) Other, SPECIFY: ______________________________________	1	2
	j) Other, SPECIFY: ______________________________________	1	2
	k) Other, SPECIFY: ______________________________________	1	2
**5.8**	Do you have any written SOPs, protocols or flow charts that focus specifically on how to care for children who have experienced sexual violence – apart from those that focus on adults?	1=Yes<br>2=No<br>8= Don't know	

SECTION 6: SUGGESTIONS

Interviewer: *We are now coming to the end of our interview.*

Q#	Question	Response	Skip pattern

**6.0**	a) Do you feel you have the knowledge and skills necessary to provide adequate care to adult and child GBV clients?	1= Yes →<br>2= No<br>8= Don't know	**skip to 6.1**
	b) What additional knowledge and skills do you feel providers need to provide adequate care to GBV clients? <u>List all mentioned.</u>		
**6.1**	a) In your opinion, do providers at this facility have the equipment, supplies, and infrastructure necessary to provide adequate medical care to adult and child GBV clients?	1= Yes →<br>2= No	**skip to 6.2**
	b) What additional equipment, supplies, and infrastructure are needed to provide adequate care to GBV clients? <u>List all mentioned.</u>		
**6.2**	What are the challenges, if any, that this facility faces in responding to GBV? <u>List all mentioned.</u>		
**6.3**	Do you have any final suggestions for improving this facility's response to GBV? <u>List all mentioned</u>		
**6.4**	Do you have any questions for me? <u>List all mentioned</u>		
**Interviewer:** *Thank you very much for answering these questions. We appreciate your time and honesty.*			

NOTE TO INTERVIEWER: PLEASE BE SURE TO RETURN TO PAGE 1, and INDICATE WHETHER THE SURVEY WAS COMPLETED. BE SURE YOU SIGN THE SURVEY AND COMPLETE ALL GENERAL INFORMATION ON PAGE 1.

Annex B-7.

Key informant interview guide: Program managers

Key Informant Interview Guide: Program Managers

7. Introduction

Thank you for taking time to speak with me today. My name is ____________, and I look forward to our discussion. You have been invited to participate in this discussion because of your role as a program manager of the WRP/HJFMRI GBV program. The overall purpose of our discussion today is to help the study team learn about the roll out and implementation of the program and your experience with the program. We hope this information will help improve GBV programs and services in the future.

Please remember that anything you share with me will be kept confidential. We will tape record the conversation, but we will not use your name on the tape, nor will we connect your name to any of the information collected. At any time, for any reason, you can stop the interview or decline to answer a question. The discussion will take about one hour.

Do you have any questions for me before we begin (pause for questions)?

8. **Your roles and responsibilities with the WRP/HJFMRI GBV program**

I am going to start the discussion by asking you about your roles and responsibilities with the program.

Please tell me about what you do that is related to the GBV program:

Note to interviewer: Please probe on the following items:

- What is your job title? To whom do you report?
- When did you start working for the program?
- What are your main responsibilities—and what do you do on a day-to-day basis?
- Do you interact regularly with other partner organizations? If so which ones and in what capacity?
- Do you work as part of a team? If so, which team?
- Do you provide services or training directly to program participants? If so, to whom?

2. Description of the overall program (or component that you manage) and workplan

Now I would like you tell me in more detail about the program component that you manage.

Note to interviewer: Please probe on the following items:

- Do you follow a workplan for your activities? (ask for a copy, if available) Who developed the workplan?
- Have you met the targets and deadlines of your workplan?
- Please describe the GBV interventions in detail – e.g., what is the intervention? Who participates? How many? How often?
- Who is your target population?
- What geographic or community areas does your program cover?

- Are there other similar programs in the communities where you work? IF so, what are they? Do you coordinate/collaborate with them, and, if so, how?
- What is your budget for this program component? Is this sufficient funding for your workplan? Are funds available when you need them?

9. Implementation successes

What aspects of your program have been most successful?

Note to interviewer: Please probe on the following:

- How do you measure success?
- Do you feel that the program is making a difference in participants' lives? If so, how?
- Do you feel that the program is making a difference in your community? If so, how?
- Can you please give some examples of cases that have benefited from your program?
- What do you think contributed to these successes?

10. Implementation challenges

What aspects of your program have been the most challenging?

Note to interviewer: Please probe on the following:

- Have these been challenges all along—or do they come and go?
- How do you recognize problems? Do you know the cause of the problems?
- What steps have you taken to resolve the problems? Has this worked?
- What else do you need, if anything, to help resolve problems?

11. Recommendations

Based on your experiences, what recommendations do you have for others who manage or offer similar programs (or would like to)?

Note to interviewer: Please probe on the following:

- How important is this program component vis a vis the other components of the comprehensive GBV program?
- What can you do to improve your program?
- What can others do to help improve your program?
- What lessons have you learned that you would like to share with others?

12. Wrap up

What else you would like to share with me regarding GBV in your community? What else would you like to share with me about the GBV program?

Thank you very much for participating in this discussion.
