# Supplementary material for: Cluster randomized trial of comprehensive gender-based violence programming delivered through the HIV/AIDS program platform in Mbeya Region, Tanzania: Tathmini GBV study
Source: PLoS One. 2018 Dec 6;13(12):e0206074. doi: 10.1371/journal.pone.0206074 (PMC6283609; doi:10.1371/journal.pone.0206074)
Supplement: S2 File — (PDF) [file pone.0206074.s003.pdf]

Project SEARCH Task Order 9: HOUSEHOLD SURVEY

BASELINE

CHUO KIKUU CHA AFYA NA SAYANSI
SHIRIKISHI CHA MUHIMBILI

CONFIDENTIAL

ID: [ ] [ ] [ ] [ ] [ ] [ ] [ ]

ID: [ ][ ][ ][ ][ ][ ][ ]

HOUSEHOLD IDENTIFICATION

STUDY CLUSTER NAME __________________________ DISTRICT NAME ___________________ CODE: [ ] [ ]

ENUMERATION AREA (EA) NUMBER: [ ] [ ] [ ] [ ] [ ] [ ] [ ] [ ] [ ] [ ] [ ] [ ]

NAME OF WARD _______________________________________ CODE: [ ] [ ] [ ]

VILLAGE/HAMLET/ STREET ________________________________ HOUSE NUMBER (if available) ____________

OTHER INFO TO IDENTIFY HOUSEHOLD (if needed)
____

INTERVIEW PARTICULARS

	1st VISIT	2nd VISIT	3rd VISIT	FINAL VISIT
DATE (DD/MM/YYYY)	______________	______________	______________	DATE _ _ /_ _ / _ _ _ _ DD/ MM/ YYYY
INTERVIEWERS ID	______________	______________	______________	
RESULT*	[ ] [ ]	[ ] [ ]	[ ] [ ]	RESULT* [ ] [ ]
NEXT VISIT, IF SCHEDULED Date:	______________	______________		Total Number of visits: [ ]
Time:	______________	______________		

* RESULT CODES:
01. Interview completed
02. No competent adult in household at time of visit
03. No eligible females living in household
04. Entire household absent for extended period of time
05. Refused by household head/representative
06. Selected participant not present; rescheduled
07. Selected participant not present; could not reschedule
08. Refused by selected female
09. Refused by parent
10. Other specify: ____________________

TOTAL # PERSONS IN HOUSEHOLD [ ][ ]

TOTAL # ELIGIBLE FEMALES AGED 15-49 [ ]

LINE # OF SELECTED ELIGIBLE FEMALE [ ]

STUDY CONSENT SUMMARY

For selected respondents ages 18-49, ADULT INFORMED CONSENT obtained?	YES [ ] NO [ ]
For selected respondents ages 15-17, PARENTAL CONSENT obtained?	YES [ ] NO [ ]
MINOR ASSENT obtained?	YES [ ] NO [ ]

QUESTIONNAIRE REVIEW AND PROCESSING			
TEAM LEADER SIGN OFF<br>NAME ________________<br>DATE _ _/_ _/ _ _ _ _<br>dd /mm/yyyy	QUESTIONNAIRE CHECKED BY<br>NAME<br>___________________<br>DATE _ _/_ _/ _ _ _ _<br>dd /mm/yyyy	OFFICE EDITOR<br>NAME<br>________________<br>DATE _ _/_ _/ _ _ _ _<br>dd /mm/yyyy	ENTERED BY<br>ENTRY 1: ________<br>ENTRY 2: ________

ID: [ ] [ ] [ ] [ ] [ ] [ ] [ ]

ELIGIBLE FEMALES IN THE HOUSEHOLD

INTERVIEWER OR TEAM LEADER INSTRUCTIONS:

For purposes of this study, a household is defined as: A group of individuals who usually live and eat together, whether or not they are related by blood or marriage, with one person, male or female, acknowledged as the head of the household. A household can consist of one person or many persons.

Several households may reside in one dwelling. If this is the case, randomly select one of the households (writing household labels on pieces of paper, then randomly drawing from a bag).

Upon reaching the house, ask to speak to the household head. If s/he is not available, ask to speak to any (competent) adult who resides in the household. If such individual is not there, ask for a time when s/he will return.

Then proceed with <u>Brief Introduction of the Study</u>. Then ask the following questions of the household head/adult.

1.	Please can you tell me how many people live here, and share food regularly?	TOTAL NUMBER OF<br>PEOPLE IN HOUSEHOLD [ ][ ]
2.	Is the head of the household male or female?	MALE ....1<br>FEMALE 2
3.	What is your relationship to the head of this household?	[ ] [ ] CIRCLE CODE BELOW AND ENTER HERE

CODES

01 HEAD		
02 WIFE (PARTNER)	11 HUSBAND(PARTNER)	20 OTHER RELATIVE, SPECIFY: ____________________________
03 DAUGHTER	12 SON	
04 DAUGHTER IN LAW	13 SON IN LAW	
05 GRANDDAUGHTER	14 GRAND SON	21 OTHER NON RELATIVE, SPECIFY: ____________________________
06 MOTHER	15 FATHER	
07 MOTHER IN LAW	16 FATHER IN LAW	
08 SISTER	17 BROTHER	
09 SISTER IN LAW	18 BROTHER IN LAW	
10 ADOPTED/FOSTER/STEP DAUGHTER	19 ADOPTED/FOSTER/STEP SON	

ID: [ ][ ][ ][ ][ ][ ][ ]

	LIST OF FEMALE HOUSEHOLD MEMBERS AGES 15-49 WHO HAVE LIVED IN HH FOR PAST 6 MONTHS	RELATIONSHIP TO HEAD OF HH	AGE
4.	Today I would like to talk to one girl or woman from your household. To enable me to identify with whom I should talk, would you please give me the first names of:<br>• all girls and women ages 15-49 who usually live in your household (and share food) AND<br>• who have lived in the household for the past 6 months	What is the relationship of NAME to the head of the household?<br><br>USE CODES BELOW	How old is NAME?
INTEVIEWER INSTRUCTIONS: List eligible females in the order given to you.			
**LINE #**	**NAME**	**CODE**	**AGE (YEARS)**
**1**			
**2**			
**3**			
**4**			
**5**			
**6**			
**7**			
**8**			

CODES

01 HEAD	07 MOTHER IN LAW
02 WIFE (PARTNER)	08 SISTER
03 DAUGHTER	09 SISTER IN LAW
04 DAUGHTER IN LAW	10 ADOPTED/FOSTER/STEP DAUGHTER
05 GRANDDAUGHTER	11 OTHER RELATIVE, SPECIFY:____________________________
06 MOTHER	12 OTHER NON RELATIVE, SPECIFY:________________________

ID: [ ][ ][ ][ ][ ][ ][ ]

RANDOM SELECTION OF PARTICIPANT

INTERVIEWER INSTRUCTIONS:

- Identify the last digit of the **ID Number**. Go to this **row** in the table below.
- Obtain the total **number of eligible women** from the listing above. Go to this column number.
- Follow the selected row and column to the cell where they meet and **circle the number** in the cell. This is the line number of the female to be selected for the interview (from table above).
- Write the name and line number of the selected female in the space below the table.

**LAST DIGIT OF THE ID NUMBER**	**TOTAL NUMBER OF ELIGIBLE WOMEN IN HOUSEHOLD**							
	**1**	**2**	**3**	**4**	**5**	**6**	**7**	**8**
**0**	1	2	2	4	3	6	5	4
**1**	1	1	3	1	4	1	6	5
**2**	1	2	1	2	5	2	7	6
**3**	1	1	2	3	1	3	1	7
**4**	1	2	3	4	2	4	2	8
**5**	1	1	1	1	3	5	3	1
**6**	1	2	2	2	4	6	4	2
**7**	1	1	3	3	5	1	5	3
**8**	1	2	1	4	1	2	6	4
**9**	1	1	2	1	2	3	7	5

NAME OF SELECTED WOMAN___________________ LINE NUMBER OF SELECTED WOMAN [ ]

AGE VERIFICATION

Interviewer instructions:
Ask to speak with the selected girl/woman. Ask her age and date of birth and record:

Reported age: _ _ **Date of birth: _ _/_ _/_ _ _ _** **Calculated age: _ _**

- If reported or calculated age is 20 or younger:
  - Ask to see written documentation. This could include birth certificate, clinic card, passport, religion card, ID card, or other official document.
  - If documentation indicates she is aged of 15-17, continue with Parental Consent process.
    If documentation indicates she is aged 18+, continue with Adult Informed Consent.
  - If no documentation is available to verify age, then remove the name of this person from the list of eligible female members and move up all other names by one row. Repeat the random selection process, and then age verification, as indicated by reported age.

  [ ] Tick here if you had to repeat the random selection because of no age documents

- If reported or calculated age is age 21-49, continue with the Adult Informed Consent process.

  If the selected girl/woman is not available, reschedule a visit. If her age on the roster of eligible females is 20 or younger, before you leave attempt to verify her age through written documentation with others in the household.

ID: [ ][ ][ ][ ][ ][ ][ ]

DODOSO YA MHOJIWA

Utangulizi

MHOJAJI:

Kama ulivyojifunza, dhumuni la tafiti hii ni kupima uzito wa programu na huduma zinazotolewa kwenye vituo vya afya na katika jamii kwa ajili ya kuleta maendeleo katika afya ya wanawake na watoto. Nitakuuliza maswali na kurekodi majibu yake kwenye hii fomu. Mahojiano yatachukua takribani saa 1 – 11/2. Kama tulivyoainisha kwenye fomu ya idhini, unaweza kukuta baadhi ya maswali ni ya undani zaidi. Si lazima ujibu swali lolote litalofanya ujisikie vibaya. Na pia unaweza sitisha mahojiano muda wowote. Tutaanza mahojiano pindi tu tuko sehemu ya faragha na yenye utulivu. Mtu yeyote atakapoingia tutasimamisha mahojiano na kutafuta sehemu nyingine yenye utulivu kama italazimu, ili kuhakikisha unaridhika kuendelea na mahojiano.

NAKILI MUDA WA KUANZA MAHOJIANO: [ ] [ ]:[ ] [ ] (tumia saa 24)

Kipengele cha 1: Taarifa za awali za mhojiwa na watoto wake

MASWALI NA CHUJAJI		UFUNGUO WA MAKUNDI	NENDA
MHOJAJI: Kama hutojali, ningependa kuanza kwa kuuliza maswali kuhusu wewe, familia na kaya yako.			
➢ **Sifa za kaya**			
001	Nini chanzo kikuu cha maji ya kunywa kwa ajili ya wanakaya wako?	MAJI YA BOMBA . . . . .1<br>*YANAYOFIKA KWENYE MAKAZI, KWENYE KIWANJA/UWAZI, BOMBA LA JUMUIYA, BOMBA LA JIRANI*<br>MAJI KUTOKA KISIMA CHA WAZI . . . . 2<br>*KATIKA MAKAZI, KATIKA KIWANJA/MAKAZI, CHA JUMUIYA, AU CHA JIRANI*<br>MAJI KUTOKA KISIMA KILICHOFUNIKWA . . . . . 3<br>*KINACHOLINDWA KATIKA MAKAZI, KWENYE UWAZI/KIWANJA, KINACHOLINDWA CHA JUMUIYA, AU CHA JIRANI*<br>MAJI YA WAZI . . . . 4<br>*MFEREJI, KIJITO, BWAWA/ZIWA,LAMBO*<br>MAJI YA MVUA . . . . 5<br>MAJI YA MALORI . . . . 6<br>MCHUUZI WA MAJI . . . . .7<br>MAJI YA CHUPA . . . . 8<br>NYINGINE_______________________________ 9	
002	Ni aina gani ya choo ambacho kaya yako huwa inatumia?	CHA KUFLASHI/KUFLASH KWA KUMIMINA KWENDA KWENYE: TANKI LA MAJI TAKA, CHOO CHA SHIMO AU VINGINEVYO . . . . 1<br>CHOO CHA SHIMO . . . . 2<br>COMPOSTING TOILET/ECOSAN . . . . 3<br>NDOO . . . . .4<br>HAKUNA CHOO/KICHAKA/UWANJA . . . . 5<br>NYINGINE . . . . .9<br>(TAJA) ___________________________________	
003	Je kaya yenu ina umeme?	NDIO . . . . .1<br>HAPANA. 2	

ID: [ ][ ][ ][ ][ ][ ][ ]

MASWALI NA CHUJAJI		UFUNGUO WA MAKUNDI	NENDA
➢ **Umri wa Mhojiwa**			
101	Umezaliwa mwezi na mwaka gani?	MWEZI. . . . . [ ] [ ]<br>SIFAHAMU MWEZI. . . . . 9 8<br>MWAKA. . . . . [ ][ ][ ][ ]<br>SIFAHAMU MWAKA. 9 9 9 8	
102	Ulikuwa na umri gani kwenye siku yako yako ya kuzaliwa ya mwisho?<br>**FANANISHA UMRI NA Q 101 KISHA SAHIHISHA UMRI KAMA KUNA ULAZIMA**	UMRI KWA HESABU YA MIAKA. . . . . [ ] [ ]	
➢ **Elimu/Taaluma ya Mhojiwa**			
103	Unaweza kusoma na kuandika?	NDIO . . . . 1<br>HAPANA. 2	
104	Je, umewahi kusoma shule?	NDIO . . . . 1<br>HAPANA. 2	➢ 107
105	Je, ni kiwango gani cha juu cha Elimu ulichowahi kufikia?	ELIMU YA AWALI . . . . .0<br>ELIMU YA MSINGI . . . . 1<br>ELIMU YA MAFUNZO BAADA YA ELIMU YA MSINGI . . . . .2<br>ELIMU YA SEKONDARI . . . . 3<br>ELIMU YA MAFUNZO BAADA YA SEKONDARI . .4<br>CHUO KIKUU . . . . 5<br>SIJUI 8	➢ 107
106	Je, ni kiwango gani ulihitimu katika hatua hiyo?<br>**KAMA ALIHITIMU KATIKA MUDA USIOZIDI MWAKA MMOJA KATIKA HATUA HIYO NAKILI '00'.**	DARASA/KIDATO/MWAKA . . . . [ ] [ ]<br>SIJUI. 9 8	
➢ **Kazi ya Mhojiwa na umilikaji**			
107	Kama unavyofahamu, kuna wanawake ambao hufanya kazi na kulipwa kwa vitu au fedha. Wengine huuza vitu, kuwa na biashara ndogondogo au hufanya kazi katika shamba la familia au katika biashara ya familia.katika miezi 12 iliyopita, tofauti na shughuli zako za nyumbani, umefanya shughuli yoyote au kitu	NDIO . . . . .1<br>HAPANA 2	➢ 111

ID: [ ][ ][ ][ ][ ][ ][ ]

MASWALI NA CHUJAJI		UFUNGUO WA MAKUNDI	NENDA
	chochote ambacho umelipwa kifedha au kwa vitu?		
108	Je, una kazi gani--Namaanisha ni aina ipi mahsusi ya kazi unayofanya?	**a) NAKILI ATACHOKISEMA:** ____________<br>**b) CODE BAADAE NA KIONGOZI WA TIMU:**<br>Kitaalam/Ufundi/Utawala. . . .. .1<br>Ukarani. . . . . 2<br>Bidhaa na Huduma. . . .. 3<br>Utendaji wenye maarifa . . . . .4<br>Utendaji usio Maarifa . . . . .5<br>Kazi za Jamii. . ..…. . . .. .6<br>Mkulima. . . . . 7<br>Mwanafunzi . . . . .8<br>"R" Hajui kundi 9	
109	Je, wewe hufanya kazi siku zote katika mwaka au hufanya kazi kwa msimu au mara moja baada ya muda?	SIKU ZOTE KATIKA MWAKA . . . . .1<br>KWA MSIMU . . . . .2<br>MARA MOJA KWA MUDA. 3	
110	Je, unalipwa/unapata kwa fedha taslimu au kwa hali kwenye hii kazi unayofanya au hulipwi kabisa?<br><br>Kwa hali namaanisha malipo yanayotolewa kwa mali, bidhaa au huduma -- tofauti na fedha	FEDHA TASLIMU TU. . . . . .1<br>FEDHA TASLIMU NA HALI . . . . .2<br>KWA HALI TU . . . . 3<br>SILIPWI 4	
111	Je unamiliki hii nyumba au nyumba nyingine yoyote peke yako au pamoja na mtu mwingine?	NAMILIKI PEKE YANGU . . . . 1<br>TUNAMILIKI PAMOJA . . . . .2<br>SIMILIKI. 3	
112	Je unamiliki ardhi yoyote, peke yako au pamoja na mtu mwingine?	NAMILIKI PEKE YANGU . . . . 1<br>TUNAMILIKI PAMOJA . . . . 2<br>SIMILIKI 3	
➢ **Uzao na watoto**			
MHOJAJI: Sasa ningependa kukuuliza kuhusu mara zote ulizojifungua katika maisha yako.			
113	a) Je, una ujauzito kwa sasa au uwewahi kubeba ujauzito ndani ya miezi 12 iliyopita?	NDIO . . . . .1<br>HAPANA . . . . 2<br>SIJUI 8	
	b) Umeishawahi kujifungua?	NDIO . . . . .1<br>HAPANA 2	➢ 120 (HAPANA)

ID: [ ][ ][ ][ ][ ][ ][ ]

MASWALI NA CHUJAJI		UFUNGUO WA MAKUNDI		NENDA
114	Je una watoto wowote uliojifungua unaoishi nao?	NDIO . . . . 1<br>HAPANA 2		➢ 116
115	Watoto wangapi wa kiume unaoishi nao? Na, watoto wangapi wa kike unaoishi nao?<br><br>**KAMA HAKUNA, NAKILI '00'**	a) WA KIUME NYUMBANI<br>b) WA KIKE NYUMBANI	[ ][ ]<br>[ ][ ]	
116	Je, una watoto wowote wa kiume au wa kike uliojifungua walio hai usioishi nao?	NDIO . . . . 1<br>HAPANA 2		➢ 118
117	Je, watoto wangapi wa kiume wanaoishi ambao huishi nao? Na, una watoto wangapi wa kike wanaoishi ambao huishi nao?<br>**KAMA HAKUNA, NAKILI '00'**	a) WA KIUME KWENGINE . . . . .<br>b) WA KIKE KWENGINE	[ ][ ]<br>[ ][ ]	
118	Umeshawahi kujifungua mtoto wa kiume au wa kike ambaye alikuwa mzima baadaye kufariki?	NDIO . . . . 1<br>HAPANA 2		➢ 120
119	Watoto wangapi wamefariki?	IDADI YA WATOTO WALIOFARIKI . . . . . [ ] [ ]<br>SIJUI 9 8		
➢ **Kuwa na Mshirika/Mpenzi:Sasa na ndani ya miezi 12 iliyopita**				
120	Hali yako ya ndoa sasa hivi ikoje?	Nimeolewa. . . . . .1<br>Naishi na mwanaume kama tumeoana . . . . . . .2<br>Mjane. . . . . .3<br>Tumetengana . . . . 4<br>Mtalikiwa . . . . .5<br>Sijawahi kuolewa/Kuishi na mwanaume 6		➢ 124<br>➢ 124<br><br><br><br>➢ 122
121	<u>Je, katika miezi 12 iliyopita</u>, umeolewa, umeishi na mwanaume kama mmeoana?	Ndio, nimeolewa au ninaishi na mwanaume kama tumeoana. . . . . 1<br>Hapana, sijaolewa au kuishi na mwanaume . . . 2		
122	Je una rafiki wa kiume ambaye unamchukulia kama mpenzi wako hata kama hamuishi pamoja au hamjamiiani?	NDIO . . . . 1<br>HAPANA 2		➢ 124
123	<u>Katika muda wowote ndani ya miezi 12 iliyopita</u>, Je umekuwa na rafiki wa kiume ambaye	Ndio nimekuwa na mpenzi kwenye miezi 12 iliyopita 1		

ID: [ ][ ][ ][ ][ ][ ][ ]

MASWALI NA CHUJAJI		UFUNGUO WA MAKUNDI	NENDA
	unamchukulia kama mpenzi wako hata kama hamuishi pamoja au hamjamiiani?	Hapana, sikuwahi kuwa na mpenzi kwenye miezi kumi na mbili iliyopita.. 2	

UFUPISHO<br>**Maelekezo kwa Mhojaji: Angalia majibu ya maswali na. 120-123 na nakili taarifa 2 zifuatazo. Taarifa hizi zitatumika kuanzia mwanzo wa kipengele cha 3.**			
**124**	**Mpenzi kwa miezi 12 iliyopita?**	**NDIO . . . . .1**<br>Kama masharti yoyote kati ya yafuatayo yamefikiwa:<br>Na. 120 = 1 au 2<br>Na. 121=1<br>Na. 122=1<br>Na. 123=1<br><br>**HAPANA . . . . 2**<br>Kama HAKUNA sharti hapo juu lililofikiwa	
**125**	**Mpenzi sasa?**	**NDIO . . . . .1**<br>Kama masharti yoyote kati ya yafuatayo yamefikiwa:<br>Na. 120 = 1 au 2<br>Na. 122=1<br><br>**HAKUNA . . . . 2**<br>Kama HAKUNA sharti hapo juu lililofikiwa	

ID: [ ] [ ] [ ] [ ] [ ] [ ] [ ]

Kipengele cha 2: Afya ya Mhojiwa, tabia za afya, na historia ya kujamiiana

MHOJAJI:

Sasa ningependa kukuuliza maswali kuhusiana na afya yako na sehemu nyingine muhimu kuhusiana na maisha ya mwanamke yanavyohusiana na afya.

MASWALI NA CHUJAJI		UFUNGUO WA MAKUNDI	NENDA
➢ **Afya na utumiaji wa Huduma za Afya**			
201	Kwa ujumla afya yako unaweza kuiweka kundi lipi, nzuri sana, nzuri, wastani, dhoofu, dhoofu sana?	NZURI SANA . . . . . 1<br>NZURI . . . . . 2<br>WASTANI . . . . . 3<br>DHOOFU . . . . . 4<br>DHOOFU SANA 5	
202	Katika miezi 12 iliyopita, ulipata hitaji ya kupata huduma ya afya kwa ajili yako?	NDIO . . . . . 1<br>HAPANA 2	
203	Katika miezi 12 iliyopita, umetafuta huduma zozote kutoka zahanati, kituo cha afya, au hospitali kwa ajili yako?	NDIO . . . . . 1<br>HAPANA 2	 ➢ 205
204	Katika miezi 12 iliyopita, je mtoa huduma aliwahi kukuuliza kama unakabiliana na vitendo vya kikatili?	NDIO . . . . . 1<br>HAPANA . . . . . 2<br>SIJUI/SIKUMBUKI 8	

		Zipo	Hazipo	Sijui	
205	Ni aina gani ya huduma za VVU zinazopatikana kwa wanawake katika eneo lako?<br>**SOMA KILA JIBU HAPA CHINI NA ZUNGUSHIA MSIMBO SAHIHI**				
	a. Upimaji VVU na Ushauri	1	2	8	
	b. Upimaji na ushauri wa VVU kwa mwenza	1	2	8	
	c. Kukinga maambukizi kutoka kwa mama kwenda kwa Mtoto	1	2	8	
	d. Matibabu ya dawa za kupunguza makali ya VVU	1	2	8	
	e. Matibabu mengine kama vile tiba za afya kwa ajili ya magonjwa nyemelezi au hali nyingine zinazotokana na kuwa na UKIMWI	1	2	8	
	f. Tiba kinga ya VVU mara baada ya maambukizi (PEP)	1	2	8	
	g. Elimu/Taarifa za kujikinga	1	2	8	
	h. Tiba saidizi ya kiakili au kijamii/Kikundi cha usaidizi wa kiakili na kijamii	1	2	8	

206	Sitaki kufahamu majibu, lakini je, umewahi kupima kuona kama umeambukizwa VVU?	NDIO . . . . . 1<br>HAPANA . . . . . 2<br>SIJUI 8	 ➢ 209<br>➢ 209
207	Je, mara ya mwisho umepima lini?	CHINI YA MIEZI 12 ILIYOPITA. . . . . 1<br>MIEZI 12 - 24 ILIYOPITA. . . . . 2<br>ZAIDI YA MIAKA 2 ILIYOPITA . . . . . 3<br>SIJUI 8	

ID: [ ][ ][ ][ ][ ][ ][ ]

208	Tena, sitaki kufahamu majibu, lakini je, ulipewa majibu?	NDIO . . . . .1<br>HAPANA . . . . .2<br>SIJUI 8	
MHOJAJI:<br>Sasa naelekea kukuuliza maswali ambayo unaweza kujisikia ni ya undani zaidi. Tafadhali kumbuka kuwa yote utakayoniambia leo yatatunzwa kwa siri na unaweza kuruka swali lolote usilojisikia huru kulijibu.			
➢ **Matumizi ya pomble na Vilevi**			
209	Ndani ya miezi 12 iliyopita umewahi kunywa pombe?	NDIO . . . . .1<br>HAPANA 2	➢ 212
210	Je, ni mara ngapi unakunywa na kufikia hatua ya kulewa, mara zote, mara chache tu, au sijawahi?<br>Kwa kulewa, namaanisha kujisikia kulewa au kuchangamka, au kupoteza uwezo wako wa kutambua.	MARA ZOTE . . . . 1<br>MARA CHACHE . . . . .2<br>MARA MOJA KATIKA MIEZI 12. . . . . .3<br>SIJAWAHI . . . . .4<br>SIJUI 8	
211	Huwa unakunywa na nani?<br><br>**[NAKILI YOTE MHOJIWA ATAKAYOJIBU; USISOME]**	[ ] a. PEKE YANGU<br>[ ] b. PAMOJA NA MUME WANGU/MPENZI<br>[ ] c. NA RAFIKI YANGU WA KIUME<br>[ ] d. NA WAKWE<br>[ ] e. NA WANAFAMILIA WANGU<br>[ ] f. NA RAFIKI ZANGU WA KIKE<br>[ ] g. NA RAFIKI ZANGU WA KIUME<br>[ ] h. VINGINEVYO: TAJA __________________	
212	Ndani ya miezi 12 iliyopita, Je, umewahi kuvuta sigara au kutumia bangi?	NDIO . . . . .1<br>HAPANA 2	➢ 215
213	Je, ni mara ngapi ulivuta sigara au bangi mpaka kufikia kulewa: mara zote, mara chache tu, au sijawahi?	MARA ZOTE . . . . 1<br>MARA CHACHE . . . . .2<br>MARA MOJA KATIKA MIEZI 12. . . . . .3<br>SIJAWAHI . . . . .4<br>SIJUI 8	
214	Huwa unavuta sigara au bangi na nani?<br><br>**[NAKILI YOTE MHOJIWA ATAKAYOJIBU; USISOME]**	[ ] a. PEKE YANGU<br>[ ] b. PAMOJA NA MUME WANGU/MPENZI<br>[ ] c. NA RAFIKI YANGU WA KIUME<br>[ ] d. NA WAKWE<br>[ ] e. NA WANAFAMILIA WANGU<br>[ ] f. NA RAFIKI ZANGU WA KIKE<br>[ ] g. NA RAFIKI ZANGU WA KIUME<br>[ ] h. VINGINEVYO: TAJA __________________	

ID: [ ][ ][ ][ ][ ][ ][ ]

215	Katika muda wowote katika maisha yako (kama mtoto au mtu mzima, kuna mtu yeyote ameishawahi kukulazimisha kwa namna yoyote kufanya naye ngono au kufanya naye tendo lolote la kingono?	NDIO . . . . .1<br>HAPANA . . . . .2<br>HAKUJIBU 9	
216	Ulikuwa na umri gani ulipofanya ngono kwa mara ya kwanza?	UMRI KATIKA MIAKA . . . . [ ] [ ]<br>SIJUI . . . . 98<br>Sijawahi kufanya ngono 0 0	➢ **CHUJAJI HAPO CHINI**
217	Kwa mara ya kwanzia ulivyofanya ngono, unaweza kusema ulifanya kwa sababu ulitaka kufanya au ulilazimishwa kufanya bila ridhaa yako?	NILITAKA . . . . 1<br>NILILAZIMISHWA . . . . 2<br>HAKUJIBU 9	
218	Kwa ujumla, Je umefanya ngono na watu tofauti wangapi katika ya miezi 12 iliyopita?	Idadi ya wapenzi miezi 12 iliyopita [ ] [ ] [ ]<br>SIJUI . . . . 9 9 8<br>HAKUJIBU 9 9 9	

**CHUJAJI**	
**CHUJAJI YA MHOJAJI:**<br>MWENZI/MWANDANI KATIKA MIEZI 12 ILIYOPITA? **Angalia swali na. 124**	
Kama swali Na. **124 = NDIO**,<br>ENDELEA **KIPENGELE CHA 3**	Kama swali Na. **124=HAPANA**,<br>**NENDA KIPENGELE CHA 5**

ID: [ ] [ ] [ ] [ ] [ ] [ ]

Kipengele cha 3 : Tabia za MPENZI/MWANDANI

MAELEKEZO KWA MHOJAJI:

MASWALI YAFUATAYO YAULIZWE KWA MPAKA MPENZI/MWANDANI WATATU AMBAO MHOJIWA AMEKUWA NAO KATIKA MIEZI 12 ILIYOPITA.

ANZA KWA KUULIZA KUHUSU MWANDANI/MPENZI WA SASA KAMA ANAYE, (Swali Na. 125=NDIYO), AU MWANDANI/MPENZI WA HIVI KARIBUNI. MHOJIWA (R) ANAWEZA KUWA NA WANDANI/WAPENZI KWA MPIGO kwa mfano, YUKO NDANI YA NDOA ILA PIA ANA RAFIKI WA KIUME. HATUJA ULIZA HILI HAPO NYUMA ILA MASWALI YANAYOFUATIA YANARUHUSU KUHOJI. KWA HALI HIYO UNAWEZA KUSEMA KUWA NI MWENZI WA MSINGI. HATA HIVYO MTIRIRIKO WA JINSI YA KUHOJI WANDANI/WAPENZI SI TATIZO.

ULIZA MASWALI MOJA KWA MOJA KUANZIA 301 MPAKA 424 (KIPENGELE CHA NNE).
KISHA FUATA MAELEKEZO KATIKA SWALI LA 424, KWA KUANZA TENA NA 301 KAMA MHOJIWA AMEKUWA NA ZAIDI YA MPENZI/MWANDANI MMOJA KATIKA MIEZI 12 ILIYOPITA.

MHOJAJI:

Kinachofuata nitakuuliza maswali kuhusiana na mwandani/mpenzi wako wa kiume wa sasa. Tena, ningependa kukuhakikishia kuwa majibu yako yatatunzwa kwa usiri mkubwa na hayataelezwa kwa mtu yeyote. Kama tukifikia swali lolote ambalo usingependa kujibu na tutaweza kuendelea na swali linalofuata. Umenieleza awali kuwa kwa sasa una mwandani/mpenzi au kwa mwaka uliopita ulikuwa una mpenzi/mwandani…

MASWALI NA CHUJAJI		a) WA SASA, HIVI PUNDE, AU MPENZI WA MSINGI		b) MPENZI MWINGINE (1)		c) MPENZI MWINGINE (2)	
301	Nini uhusiano wako na mwandani/mpenzi wako kwako?	MUME . . . . 1<br>MPENZI MNAISHI PAMOJA. . . . . 2<br>MUME WA ZAMANI . . . . 3<br>MPENZI WA ZAMANI WA KUISHI PAMOJA . . . . .4<br>RAFIKI WA KIUME (ASIYEISHI NA MHOJIWA) . . . . .5<br>RAFIKI WA KIUME WA ZAMANI 6		MUME . . . . 1<br>MPENZI MNAISHI PAMOJA. . . . . 2<br>MUME WA ZAMANI . . . . 3<br>MPENZI WA ZAMANI WA KUISHI PAMOJA . . . . .4<br>RAFIKI WA KIUME (ASIYEISHI NA MHOJIWA) . . . . .5<br>RAFIKI WA KIUME WA ZAMANI 6		MUME . . . . 1<br>MPENZI MNAISHI PAMOJA. . . . . 2<br>MUME WA ZAMANI . . . . 3<br>MPENZI WA ZAMANI WA KUISHI PAMOJA . . . . .4<br>RAFIKI WA KIUME (ASIYEISHI NA MHOJIWA) . . . . .5<br>RAFIKI WA KIUME WA ZAMANI 6	
302	Kwa kukadiria alikuwa na umri gani kulinganisha na wewe?	MDOGO KUKUZIDI . . . . . 1<br>UNALINGANA NAYE . . . . 2<br>MWAKA 1-9 ZAIDI . . . . . 3<br>MIAKA 10+ ZAIDI . . . . . . 4<br>SIJUI 8		MDOGO KUKUZIDI . . . . . 1<br>UNALINGANA NAYE . . . . 2<br>MWAKA 1-9 ZAIDI . . . . . 3<br>MIAKA 10+ ZAIDI . . . . . . 4<br>SIJUI 8		MDOGO KUKUZIDI . . . . . 1<br>UNALINGANA NAYE . . . . 2<br>MWAKA 1-9 ZAIDI . . . . . 3<br>MIAKA 10+ ZAIDI . . . . . . 4<br>SIJUI 8	
303	Amewahi kusoma shule?	NDIO 1		NDIO 1		NDIO 1	
		HAPANA . . 2<br>SIJUI 8	➢ 306<br>➢ 306	HAPANA . . 2<br>SIJUI 8	➢ 306<br>➢ 306	HAPANA . . 2<br>SIJUI 8	➢ 306<br>➢ 306

ID: [ ][ ][ ][ ][ ][ ][ ]

MASWALI NA CHUJAJI		a) WA SASA, HIVI PUNDE, AU MPENZI WA MSINGI	b) MPENZI MWINGINE (1)	c) MPENZI MWINGINE (2)
304	Je, ni hatua gani ya juu ya elimu amefikia?	ELIMU YA AWALI . . . . . . 0<br>ELIMU YA MSINGI . . . . . .1<br>ELIMU YA MAFUNZO BAADA YA ELIMU YA MSINGI . . . . 2<br>ELIMU YA SEKONDARI . . 3<br>ELIMU YA MAFUNZO BAADA YA EMILU YA SEKONDARI . . . . .4<br>CHUO KIKUU . . . . 5<br>SIJUI . . . . 8<br>(NENDA Q 306)	ELIMU YA AWALI . . . . . . 0<br>ELIMU YA MSINGI . . . . . .1<br>ELIMU YA MAFUNZO BAADA YA ELIMU YA MSINGI . . . . 2<br>ELIMU YA SEKONDARI . . 3<br>ELIMU YA MAFUNZO BAADA YA EMILU YA SEKONDARI . . . . .4<br>CHUO KIKUU . . . . 5<br>SIJUI . . . . 8<br>(NENDA Q 306)	ELIMU YA AWALI . . . . . . 0<br>ELIMU YA MSINGI . . . . . .1<br>ELIMU YA MAFUNZO BAADA YA ELIMU YA MSINGI . . . . 2<br>ELIMU YA SEKONDARI . . 3<br>ELIMU YA MAFUNZO BAADA YA EMILU YA SEKONDARI . . . . .4<br>CHUO KIKUU . . . . 5<br>SIJUI . . . . 8<br>(NENDA Q 306)
305	Je, ni kiwango (darasa/mwaka) gani cha juu alikamilisha hatua hiyo?<br><br>**KAMA AMEKAMILISHA CHINI YA MWAKA MMOJA NAKILI '00'**	DARASA/KIDATO/MWAKA . . . .[ ] [ ]<br><br>SIJUI 9 8	DARASA/KIDATO/MWAKA . . . .[ ] [ ]<br><br>SIJUI 9 8	DARASA/KIDATO/MWAKA . . . . [ ] [ ]<br><br>SIJUI 9 8
306	Anafanya kazi gani? Nikimaanisha aina gani halisi ya kazi anayofanya?	**1) NAKILI ATACHOKISEMA:**<br>_________________<br><br>**2) YA KUKODI BAADAE:**<br>Kitaalam/Ufundi/Utawala. . . . . 1<br>Ukarani . . . . 2<br>Bidhaa na huduma . .3<br>Utendaji wenye maarifa . . . . 4<br>Utendaji usio Maarifa . . . . .5<br>Kazi za Jamii . . . . 6<br>Mkulima . . . . 7<br>Mwanafunzi . . . . 8<br>**"R"** hajui 9	**1) NAKILI ATACHOKISEMA:**<br>_________________<br><br>**2) YA KUKODI BAADAE:**<br>Kitaalam/Ufundi/Utawala. . . . . 1<br>Ukarani . . . . 2<br>Bidhaa na huduma . .3<br>Utendaji wenye maarifa . . . . 4<br>Utendaji usio Maarifa . . . . .5<br>Kazi za Jamii . . . . 6<br>Mkulima . . . . 7<br>Mwanafunzi . . . . 8<br>**"R"** hajui 9	**1) NAKILI ATACHOKISEMA:**<br>_________________<br><br>**2) YA KUKODI BAADAE:**<br>Kitaalam/Ufundi/Utawala. . . . . 1<br>Ukarani . . . . 2<br>Bidhaa na huduma . .3<br>Utendaji wenye maarifa . . . . 4<br>Utendaji usio Maarifa . . . . .5<br>Kazi za Jamii . . . . 6<br>Mkulima . . . . 7<br>Mwanafunzi . . . . 8<br>**"R"** hajui 9

ID: [ ][ ][ ][ ][ ][ ][ ]

MASWALI NA CHUJAJI		a) WA SASA, HIVI PUNDE, AU MPENZI WA MSINGI		b) MPENZI MWINGINE (1)		c) MPENZI MWINGINE (2)	
307	Je, ana/aliwahi kuwa na wake wengine au anaishi/aliwahi kuishi na wanawake wengine kama vile wameoana?	NDIO . . . . .1<br>HAPANA . . . . .2<br>SIJUI 8		NDIO . . . . .1<br>HAPANA . . . . .2<br>SIJUI 8		NDIO . . . . .1<br>HAPANA . . . . .2<br>SIJUI 8	
308	Ndani ya mwaka mmoja uliopita, je mpenzi wako alishawahi kunywa pombe?	NDIO 1		NDIO 1		NDIO 1	
		HAPANA . . . . 2<br>SIJUI 8	➢ 311<br>➢ 311	HAPANA . . . . 2<br>SIJUI 8	➢ 311<br>➢ 311	HAPANA . . . . 2<br>SIJUI 8	➢ 311<br>➢ 311
309	Je, ni mara ngapi anakunywa na kufikia hatua ya kulewa, mara zote, mara chache tu, au hajawahi?<br><br>Kwa kulewa, namaanisha kujisikia kulewa au kuchangamka, au kupoteza uwezo wake wa kutambua.	MARA ZOTE . . . . .1<br>MARA CHACHE . . . . 2<br>MARA MOJA KATIKA MIEZI 12 ILIOPITA . . . . . .3<br>SIJAWAHI . . . . .4<br>SIJUI. 8		MARA ZOTE . . . . .1<br>MARA CHACHE . . . . 2<br>MARA MOJA KATIKA MIEZI 12 ILIOPITA . . . . . .3<br>SIJAWAHI . . . . .4<br>SIJUI. 8		MARA ZOTE . . . . .1<br>MARA CHACHE . . . . 2<br>MARA MOJA KATIKA MIEZI 12 ILIOPITA . . . . . . 3<br>SIJAWAHI . . . . .4<br>SIJUI. 8	
310	Huwa anakunywa na nani?<br><br>**[NAKILI YOTE MHOJIWA ATAKAYOJIBU; USISOME]**	[ ] a. PEKE YAKE<br>[ ] b. NA MHOJIWA<br>[ ] c. NA RAFIKI WA KIKE<br>[ ] d. NA WAKWE<br>[ ] e. NA WANAFAMILIA WAKE<br>[ ] f. NA RAFIKI ZAKE WA KIKE<br>[ ] g. NA MARAFIKI ZAKE WA KIUME<br>[ ] h. NYINGINE: TAJA<br>___________________		[ ] a. PEKE YAKE<br>[ ] b. NA MHOJIWA<br>[ ] c. NA RAFIKI WA KIKE<br>[ ] d. NA WAKWE<br>[ ] e. NA WANAFAMILIA WAKE<br>[ ] f. NA RAFIKI ZAKE WA KIKE<br>[ ] g. NA MARAFIKI ZAKE WA KIUME<br>[ ] h. NYINGINE: TAJA<br>___________________		[ ] a. PEKE YAKE<br>[ ] b. NA MHOJIWA<br>[ ] c. NA RAFIKI WA KIKE<br>[ ] d. NA WAKWE<br>[ ] e. NA WANAFAMILIA WAKE<br>[ ] f. NA RAFIKI ZAKE WA KIKE<br>[ ] g. NA MARAFIKI ZAKE WA KIUME<br>[ ] h. NYINGINE: TAJA<br>___________________	

ID: [ ][ ][ ][ ][ ][ ][ ]

MASWALI NA CHUJAJI		a) WA SASA, HIVI PUNDE, AU MPENZI WA MSINGI		b) MPENZI MWINGINE (1)		c) MPENZI MWINGINE (2)	
311	Kwa mwaka uliopita, mpenzi wako alishawahi kuvuta sigara au kutumia bangi?	NDIO 1		NDIO 1		NDIO 1	
		HAPANA . . . . 2<br>SIJUI 8	➢ 314<br>➢ 314	HAPANA . . . . 2<br>SIJUI 8	➢ 314<br>➢ 314	HAPANA . . . . 2<br>SIJUI 8	➢ 314<br>➢ 314
312	Je, ni mara ngapi huvuta sigara au bangi mpaka kufikia kulevya: mara zote, mara chache tu, au hajawahi?	MARA ZOTE . . . . 1<br>MARA CHACHE . . . . 2<br>MARA MOJA KATIKA MIEZI 12. . . . . .3<br>HAJAWAHI . . . . .4<br>SIJUI. 8		MARA ZOTE . . . . 1<br>MARA CHACHE . . . . 2<br>MARA MOJA KATIKA MIEZI 12. . . . . .3<br>HAJAWAHI . . . . .4<br>SIJUI. 8		MARA ZOTE . . . . 1<br>MARA CHACHE . . . . 2<br>MARA MOJA KATIKA MIEZI 12. . . . . 3<br>HAJAWAHI . . . . .4<br>SIJUI. 8	
313	Huwa anavuta sigara au kutumia bangi na nani?<br><br>[NAKILI YOTE MHOJIWA ATAKAYOJIBU; USISOME]	[ ] a. PEKE YAKE<br>[ ] b. NA MHOJIWA<br>[ ] c. NA RAFIKI WA KIKE<br>[ ] d. NA WAKWE<br>[ ] e. NA WANAFAMILIA WAKE<br>[ ] f. NA RAFIKI ZAKE WA KIKE<br>[ ] g. NA MARAFIKI ZAKE WA KIUME<br>[ ] h. NYINGINE: TAJA<br>__________________		[ ] a. PEKE YAKE<br>[ ] b. NA MHOJIWA<br>[ ] c. NA RAFIKI WA KIKE<br>[ ] d. NA WAKWE<br>[ ] e. NA WANAFAMILIA WAKE<br>[ ] f. NA RAFIKI ZAKE WA KIKE<br>[ ] g. NA MARAFIKI ZAKE WA KIUME<br>[ ] h. NYINGINE: TAJA<br>__________________		[ ] a. PEKE YAKE<br>[ ] b. NA MHOJIWA<br>[ ] c. NA RAFIKI WA KIKE<br>[ ] d. NA WAKWE<br>[ ] e. NA WANAFAMILIA WAKE<br>[ ] f. NA RAFIKI ZAKE WA KIKE<br>[ ] g. NA MARAFIKI ZAKE WA KIUME<br>[ ] h. NYINGINE: TAJA<br>__________________	
314	Umeshawahi kufanya ngono na mpenzi huyu?	NDIO, katika miezi 12 iliyopita . . . . 1<br><br>NDIO, lakini zaidi ya miezi 12 iliyopita. . . . . 2<br>**(NENDA KIPENGELE CHA 4)**<br><br>HAPANA, sijawahi kufanya mapenzi na mpenzi huyu . . . . 3<br>**(NENDA KIPENGELE CHA 4)**		NDIO, katika miezi 12 iliyopita . . . . 1<br><br>NDIO, lakini zaidi ya miezi 12 iliyopita. . . . . 2<br>**(NENDA KIPENGELE CHA 4)**<br><br>HAPANA, sijawahi kufanya mapenzi na mpenzi huyu . . . . 3<br>**(NENDA KIPENGELE CHA 4)**		NDIO, katika miezi 12 iliyopita . . . . 1<br><br>NDIO, lakini zaidi ya miezi 12 iliyopita. . . . . 2<br>**(NENDA KIPENGELE CHA 4)**<br><br>HAPANA, sijawahi kufanya mapenzi na mpenzi huyu . . . . 3<br>**(NENDA KIPENGELE CHA 4)**	
315	Kwa kukadiria ni mara ngapi, mlifanya ngono na mtu huyu katika miezi 12 iliyopita?<br><br>SOMA MAKUNDI	MARA KWA MARA (mara 2-3 /wiki) . . . . 1<br><br>MARA CHACHE (mara kadhaa/mwezi) . . 2<br><br>MARA MOJAMOJA . . . . . 3<br>MARA MOJA . . . . 4<br>SIJUI 8		MARA KWA MARA (mara 2-3 /wiki) . . . . 1<br><br>MARA CHACHE (mara kadhaa/mwezi) . . 2<br><br>MARA MOJAMOJA . . . . . 3<br>MARA MOJA . . . . 4<br>SIJUI 8		MARA KWA MARA (mara 2-3 /wiki) . . . . 1<br><br>MARA CHACHE (mara kadhaa/mwezi) . . 2<br><br>MARA MOJAMOJA . . . . . 3<br>MARA MOJA . . . . 4<br>SIJUI 8	

ID: [ ][ ][ ][ ][ ][ ][ ]

<table>
<tr><th colspan="2">MASWALI NA CHUJAJI</th><th colspan="2">a) WA SASA, HIVI PUNDE, AU MPENZI WA MSINGI</th><th colspan="2">b) MPENZI MWINGINE (1)</th><th colspan="2">c) MPENZI MWINGINE (2)</th></tr>
<tr><td rowspan="2">316</td><td rowspan="2">Mara ya mwisho kufanya ngono na huyu mpenzi wako mlitumia kondomu?</td><td colspan="2">NDIO . . . . 1</td><td colspan="2">NDIO . . . . 1</td><td colspan="2">NDIO . . . . 1</td></tr>
<tr><td>HAPANA . . 2
SIJUI . . . . . 8</td><td>(NENDA KIPENGELE CHA 4)</td><td>HAPANA . . 2
SIJUI . . . . . 8</td><td>(NENDA KIPENGELE CHA 4)</td><td>HAPANA . . 2
SIJUI . . . . . 8</td><td>(NENDA KIPENGELE CHA 4)</td></tr>
<tr><td>317</td><td><u>Katika miezi 12 iliyopita</u>, mlikuwa mnatumia kondomu kila mfanyapo ngono na mtu huyu?</td><td colspan="2">NDIO 1
HAPANA 2
SIJUI . . . . 8</td><td colspan="2">NDIO 1
HAPANA 2
SIJUI . . . . 8</td><td colspan="2">NDIO 1
HAPANA 2
SIJUI . . . . 8</td></tr>
</table>

ID: [ ] [ ] [ ] [ ] [ ] [ ] [ ]

Kipengele cha 4 : Ukatili toka kwa mpenzi/mwandani (IPV) katika miezi 12

MHOJAJI:

Soma hii mara ya kwanza tu/kwa mpenzi huyu. Sasa ningependa kukuuliza maswali kuhusu hali ambazo zinawatokea baadhi ya wanawake. Nakuomba uweze kukumbuka ndani ya miezi 12 iliyopita na tafadhali nieleze kama yamewahi kukutokea. Kuna baadhi ya maswali yanaweza kuwa ya undani sana, kama tutafikia swali lolote ambalo usingependa kulijibu, naomba unitaarifu na nitaliruka na kuendelea na maswali mengine. Na pia ningependa kukumbusha kuwa, kama ukipenda muda wowote unaweza kusimamisha mahojiano.

MASWALI na CHUJAJI		a) WA SASA, HIVI PUNDE, AU MPENZI WA MSINGI	b) MPENZI MWINGINE (1)	c) MPENZI MWINGINE (2)
**MHOJAJI**:<br>Tukiendelea kuongea kuhusiana na mpenzi/mshirika tuliyekuwa tukimuongelea, tafadhali naomba unitaarifu mara ngapi kwa namna yoyote kama yafuatayo yamewahi kukutokea ndani ya mwaka mmoja ulioisha.				
➢ **Tabia ya kutawala ya Mpenzi**				
401	Katika miezi 12 iliyopita, Je mpenzi wako amekuwa na wivu au hasira pale ulipoongea na wanaume wengine?, mara zote, mara chache tu, au hajawahi?	MARA NYINGI . . . . . . .1<br>MARA CHACHE. . . . . . 2<br>MARA MOJA . . . . . . .3<br>HAJAWAHI. . . . . .4<br>SIJUI . . . . 8<br>HAKUNA JIBU 9	MARA NYINGI . . . . . . .1<br>MARA CHACHE. . . . . . 2<br>MARA MOJA . . . . . . .3<br>HAJAWAHI. . . . . .4<br>SIJUI . . . . 8<br>HAKUNA JIBU 9	MARA NYINGI . . . . . . .1<br>MARA CHACHE. . . . . . 2<br>MARA MOJA . . . . . . .3<br>HAJAWAHI. . . . . . . .4<br>SIJUI . . . . 8<br>HAKUNA JIBU 9
402	Amewahi kukulaumu kuwa wewe sio muaminifu?	MARA NYINGI . . . . . . .1<br>MARA CHACHE. . . . . . 2<br>MARA MOJA . . . . . . .3<br>HAJAWAHI. . . . . .4<br>SIJUI . . . . 8<br>HAKUNA JIBU 9	MARA NYINGI . . . . . . .1<br>MARA CHACHE. . . . . . 2<br>MARA MOJA . . . . . . .3<br>HAJAWAHI. . . . . .4<br>SIJUI . . . . 8<br>HAKUNA JIBU 9	MARA NYINGI . . . . . . .1<br>MARA CHACHE. . . . . . 2<br>MARA MOJA . . . . . . .3<br>HAJAWAHI. . . . . .4<br>SIJUI . . . . 8<br>HAKUNA JIBU 9
403	Amewahi kukuzuia kukutana na rafiki zako wa kike?	MARA NYINGI . . . . . . .1<br>MARA CHACHE. . . . . . 2<br>MARA MOJA . . . . . . .3<br>HAJAWAHI. . . . . .4<br>SIJUI . . . . 8<br>HAKUNA JIBU 9	MARA NYINGI . . . . . . .1<br>MARA CHACHE. . . . . . 2<br>MARA MOJA . . . . . . .3<br>HAJAWAHI. . . . . .4<br>SIJUI . . . . 8<br>HAKUNA JIBU 9	MARA NYINGI . . . . . . .1<br>MARA CHACHE. . . . . . 2<br>MARA MOJA . . . . . . .3<br>HAJAWAHI. . . . . .4<br>SIJUI . . . . 8<br>HAKUNA JIBU 9
404	Alijaribu kukuzuia kuwasiliana na familia yako?	MARA NYINGI . . . . . . .1<br>MARA CHACHE. . . . . 2<br>MARA MOJA . . . . . . .3<br>HAJAWAHI. . . . . .4<br>SIJUI . . . . 8<br>HAKUNA JIBU 9	MARA NYINGI . . . . . . .1<br>MARA CHACHE. . . . . . 2<br>MARA MOJA . . . . . . .3<br>HAJAWAHI. . . . . .4<br>SIJUI . . . . 8<br>HAKUNA JIBU 9	MARA NYINGI . . . . . . .1<br>MARA CHACHE. . . . . . 2<br>MARA MOJA . . . . . . .3<br>HAJAWAHI. . . . . .4<br>SIJUI . . . . 8<br>HAKUNA JIBU 9

ID: [ ][ ][ ][ ][ ][ ]

MASWALI na CHUJAJI		a) WA SASA, HIVI PUNDE, AU MPENZI WA MSINGI	b) MPENZI MWINGINE (1)	c) MPENZI MWINGINE (2)
405	Kwa ujumla, je alisisitiza kujua ulipo muda wote?	MARA NYINGI . . . . . 1<br>MARA CHACHE . . . . . 2<br>MARA MOJA . . . . . . .3<br>HAJAWAHI . . . . 4<br>SIJUI . . . . 8<br>HAKUNA JIBU 9	MARA NYINGI . . . . . . .1<br>MARA CHACHE . . . . . .2<br>MARA MOJA . . . . . . .3<br>HAJAWAHI . . . . . . 4<br>SIJUI . . . . 8<br>HAKUNA JIBU 9	MARA NYINGI . . . . . . .1<br>MARA CHACHE . . . . . .2<br>MARA MOJA . . . . . . .3<br>HAJAWAHI . . . . 4<br>SIJUI . . . . 8<br>HAKUNA JIBU 9
406	Alijaribu kutawala matumizi yako ya pesa?	MARA NYINGI . . . . . . .1<br>MARA CHACHE . . . . . . 2<br>MARA MOJA . . . . . . .3<br>HAJAWAHI . . . . 4<br>SIJUI . . . . 8<br>HAKUNA JIBU 9	MARA NYINGI . . . . . . .1<br>MARA CHACHE . . . . . .2<br>MARA MOJA . . . . . . .3<br>HAJAWAHI . . . . 4<br>SIJUI . . . . 8<br>HAKUNA JIBU 9	MARA NYINGI . . . . . . .1<br>MARA CHACHE . . . . . .2<br>MARA MOJA . . . . . . .3<br>HAJAWAHI . . . . 4<br>SIJUI . . . . 8<br>HAKUNA JIBU 9
407	Je, unaweza kusema pesa uliyopata katika miezi 12 iliyopita, ni zaidi ya pesa mpenzi wako aliyopata, ni pungufu ya aliyopata mpenzi wako, au karibu sawasawa?	ZAIDI YAKE . . . . 1<br>PUNGUFU YAKE . . . .2<br>KARIBU SAWASAWA 3<br>SIJUI. 8	ZAIDI YAKE . . . . 1<br>PUNGUFU YAKE . . . .2<br>KARIBU SAWASAWA 3<br>SIJUI. 8	ZAIDI YAKE . . . . 1<br>PUNGUFU YAKE . . . .2<br>KARIBU SAWASAWA 3<br>SIJUI. 8
➢	**Kujisikia kunyanyaswa kihisia**			
408	Katika miezi 12 iliyopita:<br>Husema au hufanya vitu vya kukudhalilisha mbele ya watu wengine?	MARA NYINGI . . . . . . .1<br>MARA CHACHE . . . . . . 2<br>MARA MOJA . . . . . . .3<br>HAJAWAHI . . . . 4<br>SIJUI . . . . 8<br>HAKUNA JIBU 9	MARA NYINGI . . . . . . .1<br>MARA CHACHE . . . . . . 2<br>MARA MOJA . . . . . . .3<br>HAJAWAHI . . . . 4<br>SIJUI . . . . 8<br>HAKUNA JIBU 9	MARA NYINGI . . . . . . .1<br>MARA CHACHE . . . . . . 2<br>MARA MOJA . . . . . . .3<br>HAJAWAHI . . . . 4<br>SIJUI . . . . 8<br>HAKUNA JIBU 9
409	Ametishia kukuumiza au kukudhuru, au kumuumiza mtu wa jirani yako?	MARA NYINGI . . . . . . .1<br>MARA CHACHE . . . . . . 2<br>MARA MOJA . . . . . . .3<br>HAJAWAHI . . . . 4<br>SIJUI . . . . 8<br>HAKUNA JIBU 9	MARA NYINGI . . . . . . .1<br>MARA CHACHE . . . . . . 2<br>MARA MOJA . . . . . . .3<br>HAJAWAHI . . . . 4<br>SIJUI . . . . 8<br>HAKUNA JIBU 9	MARA NYINGI . . . . . . .1<br>MARA CHACHE . . . . . . 2<br>MARA MOJA . . . . . . .3<br>HAJAWAHI . . . . 4<br>SIJUI . . . . 8<br>HAKUNA JIBU 9
410	Alikutukana au kukufanya ujisikie vibaya?	MARA NYINGI . . . . . . .1<br>MARA CHACHE . . . . . . 2<br>MARA MOJA . . . . . . .3<br>HAJAWAHI . . . . 4<br>SIJUI . . . . 8<br>HAKUNA JIBU 9	MARA NYINGI . . . . . . .1<br>MARA CHACHE . . . . . . 2<br>MARA MOJA . . . . . . .3<br>HAJAWAHI . . . . 4<br>SIJUI . . . . 8<br>HAKUNA JIBU 9	MARA NYINGI . . . . . . .1<br>MARA CHACHE . . . . . . 2<br>MARA MOJA . . . . . . .3<br>HAJAWAHI . . . . 4<br>SIJUI . . . . 8<br>HAKUNA JIBU 9
➢	**Hali ya kujisikia kunyanyaswa kivitendo**			
411	Alishawahi kukusukuma, kukutikisa, au kukurushia kitu?	MARA NYINGI . . . . . . .1<br>MARA CHACHE . . . . . . 2<br>MARA MOJA . . . . . . .3<br>HAJAWAHI . . . . 4<br>SIJUI 8	MARA NYINGI . . . . . . .1<br>MARA CHACHE . . . . . . 2<br>MARA MOJA . . . . . . .3<br>HAJAWAHI . . . . 4<br>SIJUI 8	MARA NYINGI . . . . . . .1<br>MARA CHACHE . . . . . . 2<br>MARA MOJA . . . . . . .3<br>HAJAWAHI . . . . 4<br>SIJUI 8

ID: [ ][ ][ ][ ][ ][ ][ ]

MASWALI na CHUJAJI		a) WA SASA, HIVI PUNDE, AU MPENZI WA MSINGI	b) MPENZI MWINGINE (1)	c) MPENZI MWINGINE (2)
		HAKUNA JIBU 9	HAKUNA JIBU 9	HAKUNA JIBU 9
412	Kukupiga makofi?	MARA NYINGI . . . . . . .1<br>MARA CHACHE . . . . . . 2<br>MARA MOJA . . . . . . .3<br>HAJAWAHI . . . . 4<br>SIJUI . . . . 8<br>HAKUNA JIBU 9	MARA NYINGI . . . . . . .1<br>MARA CHACHE . . . . . . 2<br>MARA MOJA . . . . . . .3<br>HAJAWAHI . . . . 4<br>SIJUI . . . . 8<br>HAKUNA JIBU 9	MARA NYINGI . . . . . . .1<br>MARA CHACHE . . . . . . 2<br>MARA MOJA . . . . . . .3<br>HAJAWAHI . . . . 4<br>SIJUI . . . . 8<br>HAKUNA JIBU 9
413	Alikukunja mkono au kukuvuta nywele?	MARA NYINGI . . . . . . .1<br>MARA CHACHE . . . . . . 2<br>MARA MOJA . . . . . . .3<br>HAJAWAHI . . . . 4<br>SIJUI . . . . 8<br>HAKUNA JIBU 9	MARA NYINGI . . . . . . .1<br>MARA CHACHE . . . . . . 2<br>MARA MOJA . . . . . . .3<br>HAJAWAHI . . . . 4<br>SIJUI . . . . 8<br>HAKUNA JIBU 9	MARA NYINGI . . . . . . .1<br>MARA CHACHE . . . . . . 2<br>MARA MOJA . . . . . . .3<br>HAJAWAHI . . . . 4<br>SIJUI . . . . 8<br>HAKUNA JIBU 9
414	Alikupiga ngumi au alikupiga na kitu kinachoweza kukuumiza?	MARA NYINGI . . . . . . .1<br>MARA CHACHE . . . . . . 2<br>MARA MOJA . . . . . . .3<br>HAJAWAHI . . . . 4<br>SIJUI . . . . 8<br>HAKUNA JIBU 9	MARA NYINGI . . . . . . .1<br>MARA CHACHE . . . . . . 2<br>MARA MOJA . . . . . . .3<br>HAJAWAHI . . . . 4<br>SIJUI . . . . 8<br>HAKUNA JIBU 9	MARA NYINGI . . . . . . .1<br>MARA CHACHE . . . . . . 2<br>MARA MOJA . . . . . . .3<br>HAJAWAHI . . . . 4<br>SIJUI . . . . 8<br>HAKUNA JIBU 9
415	Alikupiga teke, alikuburuza au alikuchapa?	MARA NYINGI . . . . . . .1<br>MARA CHACHE . . . . . . 2<br>MARA MOJA . . . . . . .3<br>HAJAWAHI . . . . 4<br>SIJUI . . . . 8<br>HAKUNA JIBU 9	MARA NYINGI . . . . . . .1<br>MARA CHACHE . . . . . . 2<br>MARA MOJA . . . . . . .3<br>HAJAWAHI . . . . 4<br>SIJUI . . . . 8<br>HAKUNA JIBU 9	MARA NYINGI . . . . . . .1<br>MARA CHACHE . . . . . . 2<br>MARA MOJA . . . . . . .3<br>HAJAWAHI . . . . 4<br>SIJUI . . . . 8<br>HAKUNA JIBU 9
416	Alikukaba au alikuunguza makusudi?	MARA NYINGI . . . . . . .1<br>MARA CHACHE . . . . . . 2<br>MARA MOJA . . . . . . .3<br>HAJAWAHI . . . . 4<br>SIJUI . . . . 8<br>HAKUNA JIBU 9	MARA NYINGI . . . . . . .1<br>MARA CHACHE . . . . . . 2<br>MARA MOJA . . . . . . .3<br>HAJAWAHI . . . . 4<br>SIJUI . . . . 8<br>HAKUNA JIBU 9	MARA NYINGI . . . . . . .1<br>MARA CHACHE . . . . . . 2<br>MARA MOJA . . . . . . .3<br>HAJAWAHI . . . . 4<br>SIJUI . . . . 8<br>HAKUNA JIBU 9
417	Alikutishia au alikuvamia na kisu, bunduki au silaha yoyote?	MARA NYINGI . . . . . . .1<br>MARA CHACHE . . . . . . 2<br>MARA MOJA . . . . . . .3<br>HAJAWAHI . . . . 4<br>SIJUI . . . . 8<br>HAKUNA JIBU 9	MARA NYINGI . . . . . . .1<br>MARA CHACHE . . . . . . 2<br>MARA MOJA . . . . . . .3<br>HAJAWAHI . . . . 4<br>SIJUI . . . . 8<br>HAKUNA JIBU 9	MARA NYINGI . . . . . . .1<br>MARA CHACHE . . . . . . 2<br>MARA MOJA . . . . . . .3<br>HAJAWAHI . . . . 4<br>SIJUI . . . . 8<br>HAKUNA JIBU 9
➢	**Hali ya kujisikia kunyanyasika kingono**			
418	Alikulazimisha kwa nguvu kufanya ngono naye hata kama ulikuwa hutaki?	MARA NYINGI . . . . . . .1<br>MARA CHACHE . . . . . . 2<br>MARA MOJA . . . . . . .3<br>HAJAWAHI . . . . 4<br>SIJUI . . . . 8<br>HAKUNA JIBU 9	MARA NYINGI . . . . . . .1<br>MARA CHACHE . . . . . . 2<br>MARA MOJA . . . . . . .3<br>HAJAWAHI . . . . 4<br>SIJUI . . . . 8<br>HAKUNA JIBU 9	MARA NYINGI . . . . . . .1<br>MARA CHACHE . . . . . . 2<br>MARA MOJA . . . . . . .3<br>HAJAWAHI . . . . 4<br>SIJUI . . . . 8<br>HAKUNA JIBU 9
419	Alikulazimisha kufanya vitendo vyovyote vya kingono ambavyo hukutaka?	MARA NYINGI . . . . . . .1<br>MARA CHACHE . . . . . . 2<br>MARA MOJA . . . . . . .3<br>HAJAWAHI . . . . 4<br>SIJUI 8	MARA NYINGI . . . . . . .1<br>MARA CHACHE . . . . . . 2<br>MARA MOJA . . . . . . .3<br>HAJAWAHI . . . . 4<br>SIJUI 8	MARA NYINGI . . . . . . .1<br>MARA CHACHE . . . . . . 2<br>MARA MOJA . . . . . . .3<br>HAJAWAHI . . . . 4<br>SIJUI 8

ID: [ ][ ][ ][ ][ ][ ][ ]

MASWALI na CHUJAJI		a) WA SASA, HIVI PUNDE, AU MPENZI WA MSINGI	b) MPENZI MWINGINE (1)	c) MPENZI MWINGINE (2)
		HAKUNA JIBU 9	HAKUNA JIBU 9	HAKUNA JIBU 9
➢ **Hali ya kujisikia ukatili wa zaidi wa kimaumbile**				
**MHOJAJI**:<br>Je lolote kati ya haya yafuatayo yameshawahi kukutokea ndani ya miezi 12 iliyopita kutokana na vitendo vya mpenzi wako alivyokufanyia				
420	Ulichanika, chubuka au kupata maumivu?	MARA NYINGI . . . . . . .1<br>MARA CHACHE . . . . . . 2<br>MARA MOJA . . . . . . .3<br>HAJAWAHI . . . . 4<br>SIJUI . . . . 8<br>HAKUNA JIBU 9	MARA NYINGI . . . . . . .1<br>MARA CHACHE . . . . . . 2<br>MARA MOJA . . . . . . .3<br>HAJAWAHI . . . . 4<br>SIJUI . . . . 8<br>HAKUNA JIBU 9	MARA NYINGI . . . . . . .1<br>MARA CHACHE . . . . . . 2<br>MARA MOJA . . . . . . .3<br>HAJAWAHI . . . . 4<br>SIJUI . . . . 8<br>HAKUNA JIBU 9
421	Uliumia macho, kuteguka, kutenguka, au kuungua?	MARA NYINGI . . . . . . .1<br>MARA CHACHE . . . . . . 2<br>MARA MOJA . . . . . . .3<br>HAJAWAHI . . . . 4<br>SIJUI . . . . 8<br>HAKUNA JIBU 9	MARA NYINGI . . . . . . .1<br>MARA CHACHE . . . . . . 2<br>MARA MOJA . . . . . . .3<br>HAJAWAHI . . . . 4<br>SIJUI . . . . 8<br>HAKUNA JIBU 9	MARA NYINGI . . . . . . .1<br>MARA CHACHE . . . . . . 2<br>MARA MOJA . . . . . . .3<br>HAJAWAHI . . . . 4<br>SIJUI . . . . 8<br>HAKUNA JIBU 9
422	Ulipata majeraha ya ndani, ulivunjika, uling'oka au kuvunjika jino, kuvunjika mifupa au majeraha yoyote makubwa?	MARA NYINGI . . . . . . .1<br>MARA CHACHE . . . . . . 2<br>MARA MOJA . . . . . . .3<br>HAJAWAHI . . . . 4<br>SIJUI . . . . 8<br>HAKUNA JIBU 9	MARA NYINGI . . . . . . .1<br>MARA CHACHE . . . . . . 2<br>MARA MOJA . . . . . . .3<br>HAJAWAHI . . . . 4<br>SIJUI . . . . 8<br>HAKUNA JIBU 9	MARA NYINGI . . . . . . .1<br>MARA CHACHE . . . . . . 2<br>MARA MOJA . . . . . . .3<br>HAJAWAHI . . . . 4<br>SIJUI . . . . 8<br>HAKUNA JIBU 9
➢ **Vichocheo vya kunyanyaswa kwa vitendo**				
423	Ndani ya miezi 12 iliyopita, Je, wewe umewahi kumpiga, kumsukuma, kumpiga teke au kufanya kitendo chochote kumuumiza mpenzi wako kipindi bado hajawahi kukupiga au kukuujeruhi?	MARA NYINGI . . . . . . .1<br>MARA CHACHE . . . . . . 2<br>MARA MOJA . . . . . . .3<br>HAJAWAHI . . . . 4<br>SIJUI . . . . 8<br>HAKUNA JIBU 9	MARA NYINGI . . . . . . .1<br>MARA CHACHE . . . . . . 2<br>MARA MOJA . . . . . . .3<br>HAJAWAHI . . . . 4<br>SIJUI . . . . 8<br>HAKUNA JIBU 9	MARA NYINGI . . . . . . .1<br>MARA CHACHE . . . . . . 2<br>MARA MOJA . . . . . . .3<br>HAJAWAHI . . . . 4<br>SIJUI . . . . 8<br>HAKUNA JIBU 9
**CHUJAJI:** Wapenzi wengine katika miezi 12 iliyopita?				
424	Tofauti na huyu mtu (hawa watu wawili) ambaye/ambayo tumewaongelea, umefanya ngono na mtu mwingine yeyote ndani ya miezi 12 iliyopita (kama vile,	NDIO. . . . . .1<br>RUDI SWALI Na. 301 na uliza kuanzia swali la 301 mpaka 424 kwa mpenzi wa pili; NAKILI MAJIBU KWENYE SAFU YA PILI	NDIO. . . . . .1<br>RUDI SWALI Na. 301 na uliza kuanzia swali la 301 mpaka 424 kwa mpenzi wa pili; NAKILI MAJIBU KWENYE SAFU YA PILI	**NENDA KIPENGELE CHA 5**

ID: [ ][ ][ ][ ][ ][ ][ ]

MASWALI na CHUJAJI		a) WA SASA, HIVI PUNDE, AU MPENZI WA MSINGI	b) MPENZI MWINGINE (1)	c) MPENZI MWINGINE (2)
	mume, mpenzi unayeishi naye, mpenzi wa kuishi naye wa zamani, rafiki wa kiume, rafiki wa kiume wa zamani)?	Hapana 2	Hapana 2	

Kipengele cha 5: Ukatili wa kimaumbile na kingono katika miezi 12 iliyopita

MHOJAJI:

Sasa ningependa kukuuliza kuhusiana na aina ya unyanyasaji ambao unaweza kuwa umeupata kutoka kwa wengine, (KAMA KIPENGELE CHA 3 NA 4 KILIULIZWA, BASI ONGEZA: tofauti na wapenzi tuliokwisha waongelea)

MASWALI NA CHUJAJI		UFUNGUO WA MAKUNDI	NENDA
➢ **Ukatili wa kimaumbile**			
501	Katika miezi 12 iliyopita, kuna mtu mwingine yoyote [tofauti na mpenzi/wapenzi tuliokwisha waongelea (mume, mpenzi wa kuishi naye, rafiki wa kiume) alikupiga ngumi, kofi, teke au alikufanyia kitu chochote ili kukudhuru?	NDIO . . . . .1<br>HAPANA . . . . .2<br>HAKUNA JIBU 9	 ➢ 503<br>➢ 503
502	Je, ni nani aliyekuumiza katika namna hiyo? USISOME MAKUNDI, LAKINI WEKEA ALAMA YA TIKI "√" KWA YOTE ATAKAYOTAJA MHOJIWA.<br>TUMIA KUNDI MOJA TU KWA KILA MTU ATAKAYETAJWA. KISHA ULIZA KUHUSU IDADI KWA ULIOWEKEA ALAMA: Je, hii ilitokea mara nyingi, mara chace au mara moja katika miezi 12 iliyopita?		
	a. [ ] NDUGU WA KUZALIWA NAYE	MARA NYINGI .. . . 1<br>MARA CHACHE. . . . . 2<br>MARA MOJA. 3	
	b. [ ] MKWE/NDUGU WA MPENZI	MARA NYINGI . . . . 1<br>MARA CHACHE. . . . . 2<br>MARA MOJA. 3	
	c. [ ] MUME WA ZAMANI/MPENZI WA KUISHI NAYE ZAIDI YA MIEZI 12 ILIYOPITA	MARA NYINGI . . . . 1<br>MARA CHACHE. . . . . 2<br>MARA MOJA. 3	
	d. [ ] RAFIKI WA KIUME WA ZAMANI WA ZAIDI YA MIEZI 12 ILIYOPITA	MARA NYINGI . . . . 1<br>MARA CHACHE. . . . . 2<br>MARA MOJA. 3	

ID: [ ][ ][ ][ ][ ][ ][ ]

MASWALI NA CHUJAJI		UFUNGUO WA MAKUNDI	NENDA
	e. [ ] MWALIMU	MARA NYINGI . . . . 1<br>MARA CHACHE. . . . . 2<br>MARA MOJA. 3	
	f. [ ] MWAJIRI/MTU KAZINI	MARA NYINGI . . . . 1<br>MARA CHACHE. . . . . 2<br>MARA MOJA. 3	
	g. [ ] ASKARI/MWANAJESHI	MARA NYINGI . . . . 1<br>MARA CHACHE. . . . . 2<br>MARA MOJA. 3	
	h. [ ] JIRANI/MKAZI WA MTAANI	MARA NYINGI . . . . 1<br>MARA CHACHE. . . . . 2<br>MARA MOJA. 3	
	i. [ ] MTU NISIYE MFAHAMU	MARA NYINGI . . . . 1<br>MARA CHACHE. . . . . 2<br>MARA MOJA. 3	
	j. [ ] MWINGINE, TAJA_____________	MARA NYINGI . . . . 1<br>MARA CHACHE. . . . . 2<br>MARA MOJA. 3	
➢ **Kulazimishwa kufanya ngono**			
503	Katika miezi 12 iliyopita, kuna mtu mwingine yoyote [tofauti na mpenzi/wapenzi tuliokwisha waongelea (mume, mpenzi wa kuishi naye, rafiki wa kiume) amekulazimisha kufanya ngono bila wewe kuridhia?	NDIO . . . . .1<br>HAPANA . . . . .2<br>HAKUNA JIBU 9	 ➢505<br>➢505
504	Je, ni nani aliyekulazimisha kufanya naye ngono? USISOME MAKUNDI, LAKINI WEKEA ALAMA YA TIKI “√” ATAKAYOTAJA MHOJIWA; TUMIA KUNDI MOJA TU KWA KILA MTU ATAKAYETAJWA<br><br>KISHA ULIZA KUHUSU IDADI KWA ULIOWEKEA ALAMA:<br>Je, hii ilitokea mara nyingi, mara chace au mara moja katika miezi 12 iliyopita?		
	a. [ ] BABA	MARA NYINGI . . . . 1<br>MARA CHACHE. . . . . 2<br>MARA MOJA. 3	
	b. [ ] NDUGU MWINGINE WA KUZALIWA NAYE	MARA NYINGI . . . . 1<br>MARA CHACHE. . . . . 2<br>MARA MOJA. 3	
	c. [ ] BABA WA KAMBO	MARA NYINGI . . . . 1<br>MARA CHACHE. . . . . 2<br>MARA MOJA. 3	

ID: [ ][ ][ ][ ][ ][ ][ ]

MASWALI NA CHUJAJI		UFUNGUO WA MAKUNDI	NENDA
	d. [ ] MKWE	MARA NYINGI . . . . 1<br>MARA CHACHE. . . . . 2<br>MARA MOJA. 3	
	e. [ ] MUME WA ZAMANI/MPENZI WA KUISHI NAYE ZAIDI YA MIEZI 12 ILIYOPITA	MARA NYINGI . . . . 1<br>MARA CHACHE. . . . . 2<br>MARA MOJA. 3	
	f. [ ] RAFIKI YANGU/MSHIRIKA	MARA NYINGI . . . . 1<br>MARA CHACHE. . . . . 2<br>MARA MOJA. 3	
	g. [ ] RAFIKI WA FAMILIA AU MWANAFAMILIA	MARA NYINGI . . . . 1<br>MARA CHACHE. . . . . 2<br>MARA MOJA. 3	
	h. [ ] MWALIMU	MARA NYINGI . . . . 1<br>MARA CHACHE. . . . . 2<br>MARA MOJA. 3	
	i. [ ] MWAJIRI/MTU KAZINI	MARA NYINGI . . . . 1<br>MARA CHACHE. . . . . 2<br>MARA MOJA. 3	
	j. [ ] ASKARI/MWANAJESHI	MARA NYINGI . . . . 1<br>MARA CHACHE. . . . . 2<br>MARA MOJA. 3	
	k. [ ] PADRI/MCHUNGAJI/KIONGOZI WA DINI	MARA NYINGI . . . . 1<br>MARA CHACHE. . . . . 2<br>MARA MOJA. 3	
	l. [ ] JIRANI/MKAZI WA MTAANI	MARA NYINGI . . . . 1<br>MARA CHACHE. . . . . 2<br>MARA MOJA. 3	
	m. [ ] MTU NISIYE MFAHAMU	MARA NYINGI . . . . 1<br>MARA CHACHE. . . . . 2<br>MARA MOJA. 3	
	n. [ ] MWINGINE, TAJA_____________	MARA NYINGI . . . . 1<br>MARA CHACHE. . . . . 2<br>MARA MOJA. 3	
505	Kwa ambavyo utaweza kukumbuka, je baba yako aliwahi kumpiga mama yako?	NDIO . . . . 1<br>HAPANA . . . . .2<br>SIJUI . . . . 8<br>HANA JIBU 9	

ID: [ ][ ][ ][ ][ ][ ][ ]

UFUPISHO<br>Maelekezo kwa mhojaji: Tazama tena majibu ya maswali 411-417 na 501 na nakili taarifa zifuatazo.		
**506**	**Kukutwa na walau aina moja ya ukatili wa kimaumbile katika miezi 12 iliyopita?**	**NDIO** . . . . **1**<br>Kama LOLOTE kati ya masharti yafuatayo yamefuatwa:<br>Swali na. 411 = 1, 2 or 3<br>Swali na. 412=1, 2 or 3<br>Swali na. 413=1, 2 or 3<br>Swali na. 414=1, 2 or 3<br>Swali na. 415=1, 2 or 3<br>Swali na. 416=1, 2 or 3<br>Swali na. 417=1, 2 or 3<br>Swali na. 501=1<br><br>**HAPANA/SIJUI/HAKUNA JIBU** . . . . **2**<br>Kama HAKUNA sharti kati ya hayo hapo juu lililofuatwa
**507**	**Kukutwa na walau aina moja ya unyanyasaji wa kingono katika miezi 12 iliyopita?**	**NDIO** . . . . **1**<br>Kama LOLOTE kati ya masharti yafuatayo yamefuatwa:<br>Swali na. 418 = 1, 2 or 3<br>Swali na. 419=1, 2 or 3<br>Swali na. 503=1<br><br>**HAPANA/SIJUI/HAKUNA JIBU** . . . . **2**<br>Kama HAKUNA sharti kati ya hayo hapo juu lililofuatwa
**CHUJAJI**		
**Kama Swali na. 506 =1 AU Swali na. 507=1 endelea na KIPENGELE CHA 6**<br>**Kama Swali na. 506=2 NA Swali na. 507=2 basi NENDA KIPENGELE CHA 7**		

ID: [ ][ ][ ][ ][ ][ ]

Kipengele cha 6: Matumizi ya Huduma za IPV katika miezi 12 iliyopita

MHOJAJI:
Umemaliza kunishirikisha kuwa umewahi kupatwa na matukio au hali ambazo ningependa kuzitambua kama "ukatili wa kijinsia". Ningependa kukuuliza kuhusiana na hatua zozote ulizochukua kutokana na matukio hayo.

MASWALI na CHUJAJI		UFUNGUO WA MAKUNDI	NENDA
601	Ndani ya miezi 12 iliyopita, je umewahi kutafuta msaada ili kuzuia ukatili wa kijinsia huo uliopitia –au kutafuta matibabu kwa ajili ya matokeo ya matukio hayo?	NDIO, NILITAFUTA MSAADA. . . . . .1<br>HAPANA, SIJATAFUTA MSAADA 2	 ➢ **Kipengele cha 7**
602	Je, wapi na nani ulimwendea kwa ajili ya msaada, katika miezi 12 iliyopita?<br><br>**USIMSOMEE; WEKA ALAMA KWA YOTE ATAKAYOTAJA**	a. [ ] MWANAFAMILIA MWENZANGU<br>b. [ ] WAKWE<br>c. [ ] MUME/MPENZI<br>d. [ ] MPENZI WA ZAMANI<br>e. [ ] RAFIKI WA KIUME/KIKE<br>f. [ ] RAFIKI<br>g. [ ] JIRANI<br>h. [ ] KIONGOZI WA DINI<br>i. [ ] KIONGOZI WA SERIKALI<br>j. [ ] ASKARI<br>k. [ ] MWANASHERIA/HUDUMA ZA SHERIA<br>l. [ ] ASASI YA KIRAIA/KIDINI<br>m. [ ] OFISI YA USTAWI<br>n. [ ] VIONGOZI WA SHULE<br>o. [ ] MAKAZI SALAMA<br>p. [ ] KITUO CHA AFYA<br>q. [ ] NYINGINE: TAJA<br>____________________	
603	Wapi/Nani ulimwendea kwa msaada wa kwanza?<br><br>**NAKILI HERUFI MOJA (a. – q.) YA MAJIBU HAPO JUU TOKA SWALI LA 602**	NILIENDA KWANZA . . . . [ ]	
604	Ndani ya miezi 12 iliyopita, umewahi kutembelea kituo cha afya kwa ajili ya kupata huduma, matibabu au kupata msaada kutokana na ukatili wa kijinsia ulioupata?	IDADI YA MAHUDHURIO . . . . . [ ] [ ] [ ]<br><br>**KAMA NI ZAIDI YA 1, ANZA KWA KUULIZA SWALI LA 605, KUHUSU HUDHURIO LA JIRANI ZAIDI NA ENDELEA KUULIZA MPAKA MAHUDHURIO 3**	**Kama "0," ruka mpaka kipengele cha 7**

ID: [ ][ ][ ][ ][ ][ ][ ]

MASWALI na CHUJAJI		a.<br>HUDHURIO LA KITUO CHA AFYA LA JIRANI ZAIDI	b.<br>HUDHURIO LA KITUO CHA AFYA LA PILI KWA UJIRANI ZAIDI	c.<br>HUDHURIO LA AFYA LINALOFUATIA KWA UJIRANI ZAIDI
**MHOJAJI:**<br>Sasa naelekea kukuuliza maswali kuhusiana na hayo mahudhurio kwa kuanzia na yale ya karibu kabisa.				
605	Je, kituo ulichoenda ni zahanati, kituo cha afya au hospitali.	Zahanati . . . . 1<br>Kituo cha afya . . . . . . .2<br>Hospitali . . . . 3<br>Nyingine . . . . .4<br>Taja: _______________	Zahanati . . . . 1<br>Kituo cha afya . . . . . . .2<br>Hospitali . . . . 3<br>Nyingine . . . . .4<br>Taja: _______________	Zahanati . . . . 1<br>Kituo cha afya . . . . . . .2<br>Hospitali . . . . 3<br>Nyingine . . . . .4<br>Taja: _______________
606	Je, kituo kilikuwa kinaitwaje?	1) Jina la kituo:<br>____________________	1) Jina la kituo:<br>____________________	1) Jina la kituo:<br>_____________________
607	CHUJAJI:<br>USIULIZE HILI SWALI KWA SAFU a. ULIZA TU KAMA INAFAA KWA SAFU b and c.<br>Je mahudhurio haya ilikuwa ni sababu ya matukio ya unyanyasaji kama mahudhurio yako megine katika kituo cha afya	X	NDIO, TUKIO LILE LILE . . . . .1<br>HAPANA, TUKIO TOFAUTI 2	NDIO, TUKIO LILE LILE . . . . 1<br>HAPANA, TUKIO TOFAUTI. . . . 2

ID: [ ][ ][ ][ ][ ][ ][ ]

MASWALI na CHUJAJI		a. HUDHURIO LA KITUO CHA AFYA LA JIRANI ZAIDI	b. HUDHURIO LA KITUO CHA AFYA LA PILI KWA UJIRANI ZAIDI	c. HUDHURIO LA AFYA LINALOFUATIA KWA UJIRANI ZAIDI
608	Ni aina gani ya huduma ulizofuata<br><br>USIMSOMEE; NAKILI YOTE MHOJIWA ATAKAYOTAJA	[ ] MATIBABU YA MAJERAHA YA KIMWILI<br>[ ] MATIBABU YA KUBAKWA (AU UKATILI WA KINGONO)<br>[ ] MSAADA WA KISAIKOLOJIA NA KIJAMII<br>[ ] VIPIMO VYA MAGONJWA YA ZINAA<br>[ ] MATIBABU YA MAGONJWA YA ZINAA<br>[ ] KIPIMO CHA MIMBA<br>[ ] UZAZI WA MPANGO<br>[ ] KINGA YADHARURA YA MIMBA<br>[ ] KIPIMO CHA UKIMWI<br>[ ] KINGA YA KUZUIA MAAMBUKIZI YA VVU (PEP)<br>[ ]UZINGATIAJI WA PEP<br>[ ] VIPIMO/HUDUMA ZA KIMAHAKAMA<br>[ ] RUFAA KWENDA KITUO KINGINE<br>[ ] NYINGINE, TAJA: ________________	[ ] MATIBABU YA MAJERAHA YA KIMWILI<br>[ ] MATIBABU YA KUBAKWA (AU UKATILI WA KINGONO)<br>[ ] MSAADA WA KISAIKOLOJIA NA KIJAMII<br>[ ] VIPIMO VYA MAGONJWA YA ZINAA<br>[ ] MATIBABU YA MAGONJWA YA ZINAA<br>[ ] KIPIMO CHA MIMBA<br>[ ] UZAZI WA MPANGO<br>[ ] KINGA YADHARURA YA MIMBA<br>[ ] KIPIMO CHA UKIMWI<br>[ ] KINGA YA KUZUIA MAAMBUKIZI YA VVU (PEP)<br>[ ]UZINGATIAJI WA PEP<br>[ ] VIPIMO/HUDUMA ZA KIMAHAKAMA<br>[ ] RUFAA KWENDA KITUO KINGINE<br>[ ] NYINGINE, TAJA: ________________	[ ] MATIBABU YA MAJERAHA YA KIMWILI<br>[ ] MATIBABU YA KUBAKWA (AU UKATILI WA KINGONO)<br>[ ] MSAADA WA KISAIKOLOJIA NA KIJAMII<br>[ ] VIPIMO VYA MAGONJWA YA ZINAA<br>[ ] MATIBABU YA MAGONJWA YA ZINAA<br>[ ] KIPIMO CHA MIMBA<br>[ ] UZAZI WA MPANGO<br>[ ] KINGA YADHARURA YA MIMBA<br>[ ] KIPIMO CHA UKIMWI<br>[ ] KINGA YA KUZUIA MAAMBUKIZI YA VVU (PEP)<br>[ ]UZINGATIAJI WA PEP<br>[ ] VIPIMO/HUDUMA ZA KIMAHAKAMA<br>[ ] RUFAA KWENDA KITUO KINGINE<br>[ ] NYINGINE, TAJA: ________________
609	CHUJAJI: JE ULITIKI MATIBABU YA KUBAKWA (UKATILI WA KINGONO) kwenye swali la 608	NDIO . . . . 1<br><br>HAPANA . . . . 2<br>(nenda swali la 611)	NDIO . . . . .1<br><br>HAPANA . . . . .2<br>(nenda swali la 611)	NDIO . . . . 1<br><br>HAPANA . . . . 2<br>(nenda swali la 611)
610	Je ulienda kwenye kituo cha afya ndani ya masaa 72 baada ya kubakwa, (ukatili wa ngono)?	NDIO . . . . 1<br>HAPANA . . . . 2<br>SIJUI 8	NDIO . . . . 1<br>HAPANA . . . . 2<br>SIJUI 8	NDIO . . . . 1<br>HAPANA . . . . 2<br>SIJUI 8

ID: [ ][ ][ ][ ][ ][ ][ ]

MASWALI na CHUJAJI		a. HUDHURIO LA KITUO CHA AFYA LA JIRANI ZAIDI	b. HUDHURIO LA KITUO CHA AFYA LA PILI KWA UJIRANI ZAIDI	c. HUDHURIO LA AFYA LINALOFUATIA KWA UJIRANI ZAIDI
611	Kwa nini ulichagua au ulichaguaje kwenda katika kituo niki?<br><br>**USIMSOMEE; NAKILI YOTE MHOJIWA ATAKAYOTAJA OMBA UFAFANUZI: Kama ulipata rufaa, ni nani aliyekupa hiyo rufaa?**	[ ] NILIKUWA NAFAHAMU PANATOLEWA HUDUMA HIZO KWENYE HICHO KITUO<br>[ ] KITUO KIPO JIRANI NA NINAPO ISHI<br>[ ] NI MAHALI NILIPO KWENDA KWA HUDUMA NYINGINE ZA AFYA<br>[ ] NINAWEZA KUPAFIKIA BILA WATU WENGINE KUFAHAMU<br>[ ] NILIPATA RUFAA HAPA AU NILIPELEKWA NA POLISI<br>[ ] NILIPATA RUFAA KUTOKA KWA VIONGOZI WA SERIKALI ZA MITAA<br>[ ]NILIPEWA RUFAA KUTOKA HOSPITALI NYINGNE (TAJA_______)<br>[ ]NILIELEKEZWA NA ASASI YA KIRAIA (TAJA_______)<br>[ ]NILIELEKEZWA NA NDUGU NDUGU YANGU<br>[ ] NILIELEKEZWA HAPA NA SHULE<br>[ ] NILIELEKEZWA NA AFISA USTAWI<br>[ ] NILIELEKEZWA NA RAFIKI/JIRANI<br>[ ] NDIO UAMUZI PEKEE NILIOKUWA NAO<br>[ ] NYINGINE (TAJA_________)	[ ] NILIKUWA NAFAHAMU PANATOLEWA HUDUMA HIZO KWENYE HICHO KITUO<br>[ ] KITUO KIPO JIRANI NA NINAPO ISHI<br>[ ] NI MAHALI NILIPO KWENDA KWA HUDUMA NYINGINE ZA AFYA<br>[ ] NINAWEZA KUPAFIKIA BILA WATU WENGINE KUFAHAMU<br>[ ] NILIPATA RUFAA HAPA AU NILIPELEKWA NA POLISI<br>[ ] NILIPATA RUFAA KUTOKA KWA VIONGOZI WA SERIKALI ZA MITAA<br>[ ]NILIPEWA RUFAA KUTOKA HOSPITALI NYINGNE (TAJA_______)<br>[ ]NILIELEKEZWA NA ASASI YA KIRAIA (TAJA_______)<br>[ ]NILIELEKEZWA NA NDUGU NDUGU YANGU<br>[ ] NILIELEKEZWA HAPA NA SHULE<br>[ ] NILIELEKEZWA NA AFISA USTAWI<br>[ ] NILIELEKEZWA NA RAFIKI/JIRANI<br>[ ] NDIO UAMUZI PEKEE NILIOKUWA NAO<br>[ ] NYINGINE (TAJA_________)	[ ] NILIKUWA NAFAHAMU PANATOLEWA HUDUMA HIZO KWENYE HICHO KITUO<br>[ ] KITUO KIPO JIRANI NA NINAPO ISHI<br>[ ] NI MAHALI NILIPO KWENDA KWA HUDUMA NYINGINE ZA AFYA<br>[ ] NINAWEZA KUPAFIKIA BILA WATU WENGINE KUFAHAMU<br>[ ] NILIPATA RUFAA HAPA AU NILIPELEKWA NA POLISI<br>[ ] NILIPATA RUFAA KUTOKA KWA VIONGOZI WA SERIKALI ZA MITAA<br>[ ]NILIPEWA RUFAA KUTOKA HOSPITALI NYINGNE (TAJA______)<br>[ ]NILIELEKEZWA NA ASASI YA KIRAIA (TAJA_________)<br>[ ]NILIELEKEZWA NA NDUGU NDUGU YANGU<br>[ ] NILIELEKEZWA HAPA NA SHULE<br>[ ] NILIELEKEZWA NA AFISA USTAWI<br>[ ] NILIELEKEZWA NA RAFIKI/JIRANI<br>[ ] NDIO UAMUZI PEKEE NILIOKUWA NAO<br>[ ] NYINGINE (TAJA_________)

ID: [ ][ ][ ][ ][ ][ ][ ]

MASWALI na CHUJAJI		a. HUDHURIO LA KITUO CHA AFYA LA JIRANI ZAIDI			b. HUDHURIO LA KITUO CHA AFYA LA PILI KWA UJIRANI ZAIDI			c. HUDHURIO LA AFYA LINALOFUATIA KWA UJIRANI ZAIDI		
612	Ni huduma gani ulipata katika kituo hiki?	SOMA KILA HUDUMA NA NAKILI MAJIBU								
	**Huduma**	**NDIO**	**HPN**	**SJUI**	**NDIO**	**HPN**	**SIJUI**	**NDIO**	**HPN**	**SIJUI**
	1. Kupimwa na kupata ushauri wa ukatili wa kijinsia	1	2	8	1	2	8	1	2	8
	2. Vipimo vya mwili	1	2	8	1	2	8	1	2	8
	3. Vipimo vya afya ya akili	1	2	8	1	2	8	1	2	8
	4. Matibabu ya majeraha	1	2	8	1	2	8	1	2	8
	5. Vipimo vya kimahakama	1	2	8	1	2	8	1	2	8
	6. Vipimo vya kimahakama vilichukuliwa	1	2	8	1	2	8	1	2	8
	7. Kujaza fomu ya polisi ya PF3	1	2	8	1	2	8	1	2	8
	8. Ushauri wa kisaikolojia	1	2	8	1	2	8	1	2	8
	9. Ushauri wa afya ya uzazi	1	2	8	1	2	8	1	2	8
	10. Ushauri kuhusu VVU	1	2	8	1	2	8	1	2	8
	11. Ushauri wa uzingatiaji wa PEP	1	2	8	1	2	8	1	2	8
	12. Kipimo cha mimba	1	2	8	1	2	8	1	2	8
	13. Kipimo cha VVU	1	2	8	1	2	8	1	2	8
	14. Kipimo cha magonjwa ya zinaa	1	2	8	1	2	8	1	2	8
	15. Njia za uzazi wa mpango	1	2	8	1	2	8	1	2	8
	16. Kukinga mimba kwa dharura	1	2	8	1	2	8	1	2	8
	17. Matibabu ya magonjwa ya zinaa	1	2	8	1	2	8	1	2	8
	18. PEP kwa ajili ya VVU	1	2	8	1	2	8	1	2	8

ID: [ ][ ][ ][ ][ ][ ][ ]

MASWALI na CHUJAJI		a. HUDHURIO LA KITUO CHA AFYA LA JIRANI ZAIDI			b. HUDHURIO LA KITUO CHA AFYA LA PILI KWA UJIRANI ZAIDI			c. HUDHURIO LA AFYA LINALOFUATIA KWA UJIRANI ZAIDI		
	19. Kinga ya pepopunda	1	2	8	1	2	8	1	2	8

MASWALI na CHUJAJI		a. HUDHURIO LA KITUO CHA AFYA LA JIRANI ZAIDI	b. HUDHURIO LA KITUO CHA AFYA LA PILI KWA UJIRANI ZAIDI	c. HUDHURIO LA AFYA LINALOFUATIA KWA UJIRANI ZAIDI
613	Kwa ujumla, unaweza kuongeleaje huduma uliyopata? Nzuri sana, nzuri, wastani, mbaya, mbaya sana?	NZURI SANA. . . . . . . 1<br>NZURI. . . . . .2<br>WASTANI . . . . 3<br>MBAYA . . . . 4<br>MBAYA SANA. 5	NZURI SANA. . . . . . . 1<br>NZURI. . . . . .2<br>WASTANI . . . . 3<br>MBAYA . . . . 4<br>MBAYA SANA. 5	NZURI SANA. . . . . . 1<br>NZURI. . . . . .2<br>WASTANI . . . . 3<br>MBAYA . . . . 4<br>MBAYA SANA. 5
614	Katika matembezi haya, je ulipewa rufaa kwa ajili ya huduma nyingine nje ya kituo hiki?	NDIO . . . . .1<br>HAPANA . . . . 2<br>(NENDA **CHUJAJI Na.2)**	NDIO . . . . .1<br>HAPANA . . . . 2<br>(NENDA **CHUJAJI Na.2)**	NDIO . . . . .1<br>HAPANA . . . . .2<br>(NENDA **CHUJAJI Na.2)**
615	Huduma gani ulizoelekezwa? Je ulifuatilia hizi rufaa na kwenda kwenye huduma hizo ulizoelekezwa? SOMA KILA HUDUMA NA NAKILI JIBU			
	a. Uangalizi wa kisaikolojia	NDIO, NILIELEKEZWA NA NIKAENDA . . . . .1<br>NDIO NILIELEKEZWA, ILA SIKWENDA . . . . .2<br>SIKUELEKEZWA 3	NDIO, NILIELEKEZWA NA NIKAENDA . . . . .1<br>NDIO NILIELEKEZWA, ILA SIKWENDA . . . . .2<br>SIKUELEKEZWA 3	NDIO, NILIELEKEZWA NA NIKAENDA . . . . .1<br>NDIO NILIELEKEZWA, ILA SIKWENDA . . . . .2<br>SIKUELEKEZWA 3
	b. Polisi	NDIO, NILIELEKEZWA NA NIKAENDA . . . . .1<br>NDIO NILIELEKEZWA, ILA SIKWENDA . . . . .2<br>SIKUELEKEZWA 3	NDIO, NILIELEKEZWA NA NIKAENDA . . . . .1<br>NDIO NILIELEKEZWA, ILA SIKWENDA . . . . .2<br>SIKUELEKEZWA 3	NDIO, NILIELEKEZWA NA NIKAENDA . . . . .1<br>NDIO NILIELEKEZWA, ILA SIKWENDA . . . . .2<br>SIKUELEKEZWA 3
	c. Makazi salama	NDIO, NILIELEKEZWA NA NIKAENDA . . . . .1<br>NDIO NILIELEKEZWA, ILA SIKWENDA . . . . .2<br>SIKUELEKEZWA 3	NDIO, NILIELEKEZWA NA NIKAENDA . . . . .1<br>NDIO NILIELEKEZWA, ILA SIKWENDA . . . . .2<br>SIKUELEKEZWA 3	NDIO, NILIELEKEZWA NA NIKAENDA . . . . .1<br>NDIO NILIELEKEZWA, ILA SIKWENDA . . . . .2<br>SIKUELEKEZWA 3
	d. Matibabu katika kituo cha afya cha juu zaidi	NDIO, NILIELEKEZWA NA NIKAENDA . . . . .1<br>NDIO NILIELEKEZWA, ILA SIKWENDA . . . . .2<br>SIKUELEKEZWA 3	NDIO, NILIELEKEZWA NA NIKAENDA . . . . .1<br>NDIO NILIELEKEZWA, ILA SIKWENDA . . . . .2<br>SIKUELEKEZWA 3	NDIO, NILIELEKEZWA NA NIKAENDA . . . . .1<br>NDIO NILIELEKEZWA, ILA SIKWENDA . . . . .2<br>SIKUELEKEZWA 3
	e. Huduma za kisheria	NDIO, NILIELEKEZWA NA NIKAENDA . . . . .1<br>NDIO NILIELEKEZWA, ILA SIKWENDA 2	NDIO, NILIELEKEZWA NA NIKAENDA . . . . .1<br>NDIO NILIELEKEZWA, ILA SIKWENDA 2	NDIO, NILIELEKEZWA NA NIKAENDA . . . . .1<br>NDIO NILIELEKEZWA, ILA SIKWENDA 2

ID: [ ][ ][ ][ ][ ][ ][ ]

<table>
<tr><th colspan="2">MASWALI na CHUJAJI</th><th>a.
HUDHURIO LA KITUO CHA AFYA LA JIRANI ZAIDI</th><th>b.
HUDHURIO LA KITUO CHA AFYA LA PILI KWA UJIRANI ZAIDI</th><th>c.
HUDHURIO LA AFYA LINALOFUATIA KWA UJIRANI ZAIDI</th></tr>
<tr><td></td><td></td><td>SIKUELEKEZWA . . . .3</td><td>SIKUELEKEZWA . . . .3</td><td>SIKUELEKEZWA . . . .3</td></tr>
<tr><td></td><td>f. Nyingine1: TAJA _______________</td><td>NDIO, NILIELEKEZWA NA NIKAENDA 1
NDIO NILIELEKEZWA, ILA SIKWENDA 2
SIKUELEKEZWA . . . .3</td><td>NDIO, NILIELEKEZWA NA NIKAENDA 1
NDIO NILIELEKEZWA, ILA SIKWENDA 2
SIKUELEKEZWA . . . .3</td><td>NDIO, NILIELEKEZWA NA NIKAENDA 1
NDIO NILIELEKEZWA, ILA SIKWENDA 2
SIKUELEKEZWA . . . .3</td></tr>
<tr><td></td><td>g. Nyingine2: TAJA _______________</td><td>NDIO, NILIELEKEZWA NA NIKAENDA 1
NDIO NILIELEKEZWA, ILA SIKWENDA 2
SIKUELEKEZWA . . . .3</td><td>NDIO, NILIELEKEZWA NA NIKAENDA 1
NDIO NILIELEKEZWA, ILA SIKWENDA 2
SIKUELEKEZWA . . . .3</td><td>NDIO, NILIELEKEZWA NA NIKAENDA 1
NDIO NILIELEKEZWA, ILA SIKWENDA 2
SIKUELEKEZWA . . . .3</td></tr>
<tr><td colspan="5">CHUJAJI 1:
Kama jibu lolote la Q615 ni 2 (NILIELEKEZWA LAKINI SIKWENDA), hivyo uliza Q616. Vinginevyo nenda CHUJAJI Na 2.</td></tr>
<tr><td>616</td><td>Je, ni sababu gani zilifanya usiende kwenye huduma hizi?

NAKILI YOTE ATAKAYOSEMA; USIMSOMEE</td><td>[ ]SIKUZIHITAJI HIZO HUDUMA
[ ] ZIKUWEZA KUMUDU
[ ] SIKUWA/SIKUWEZA KUMUDU USAFIRI
[ ] SIKUWA NA MUDA
[ ] NILIKUWA NA KAZI
[ ] SIKUWA NA WA KUNISAIDIA WATOTO
[ ] NILIKUWA NA MAJUKUMU YA FAMILIA/KAYA
[ ]MUME/MPENZI ASINGERUHUSU
[ ] SIKUJUA PA KWENDA
[ ] NILIOGOPA KUONEKANA/WENGINE KUGUNDUA
[ ] NYINGINE
TAJA:____________</td><td>[ ]SIKUZIHITAJI HIZO HUDUMA
[ ] ZIKUWEZA KUMUDU
[ ] SIKUWA/SIKUWEZA KUMUDU USAFIRI
[ ] SIKUWA NA MUDA
[ ] NILIKUWA NA KAZI
[ ] SIKUWA NA WA KUNISAIDIA WATOTO
[ ] NILIKUWA NA MAJUKUMU YA FAMILIA/KAYA
[ ]MUME/MPENZI ASINGERUHUSU
[ ] SIKUJUA PA KWENDA
[ ] NILIOGOPA KUONEKANA/WENGINE KUGUNDUA
[ ] NYINGINE
TAJA:____________</td><td>[ ]SIKUZIHITAJI HIZO HUDUMA
[ ] ZIKUWEZA KUMUDU
[ ] SIKUWA/SIKUWEZA KUMUDU USAFIRI
[ ] SIKUWA NA MUDA
[ ] NILIKUWA NA KAZI
[ ] SIKUWA NA WA KUNISAIDIA WATOTO
[ ] NILIKUWA NA MAJUKUMU YA FAMILIA/KAYA
[ ]MUME/MPENZI ASINGERUHUSU
[ ] SIKUJUA PA KWENDA
[ ] NILIOGOPA KUONEKANA/WENGINE KUGUNDUA
[ ] NYINGINE
TAJA:____________</td></tr>
<tr><td colspan="2">CHUJAJI Na 2:</td><td>Kama swali 604>1, basi endelea na safu b; vinginevyo NENDA KIPENGELE CHA 7</td><td>Kama swali 604>2, basi endelea na safu c; vinginevyo NENDA KIPENGELE CHA 7</td><td>NENDA KIPENGELE CHA 7</td></tr>
</table>

ID: [ ][ ][ ][ ][ ][ ][ ]

Kipengele cha 7: Ushiriki katika miradi ya ukatili wa jinsia katika jamii katika ya miezi 12 iliyopita

MHOJAJI: Sasa ningependa kukuuliza kuhusu ushiriki wako katika shughuli za kijamii.

MASWALI na CHUJAJI		UFUNGUO WA MAKUNDI	NENDA
➢ Ushiriki katika shughuli za kijamii za kila aina			
701a	Je uliwahi kuhudhuria au kushiriki shughuli za makundi, asasi au jumuiya za kijamii?<br><br>KAMA JIBU HAPANA AU SIJUI, DADISI: Hii inahusisha Jumuiya kama za wanawake, au jumuiya za kijamii, jumuiya za kidini au jumuiya za kisiasa.	NDIO . . . . 1<br>HAPANA . . . . 2<br>SIJUI 8	 ➢ 702a<br>➢ 702a
701b	Je, ni aina gani ya shughuli za kijamii, asasi au jumuiya za kijamii ulizoshiriki?<br><br>**USIMSOMEE; NAKILI YOTE ATAKAYOTAJA**	a. [ ] kikundi cha michezo<br>b. [ ] kikundi cha muziki na kucheza<br>c. [ ] kikundi cha wanawake<br>d. [ ] kikundi cha mazishi<br>e. [ ] kikundi cha dini<br>f. [ ] kikundi cha siasa<br>g. [ ] kikundi cha huduma ya VVU<br>h. [ ] kikundi cha kuelimisha rika<br>i. [ ] Kikundi cha vijana<br>j. [ ] Kikindi cha Afya ya Jamii<br>k. [ ] Asasi ya wazazi na waalimu<br>l. [ ] Kikundi cha ushonaji<br>m. [ ] Kamati ya Maendeleo (mfano; ardhi, kamati ya maji na usafi)<br>n. [ ] Nyingine, Taja ______________________	
➢ Uelewa na ushiriki katika program za jumuiya za HJFMRI/WRP GBV			
702a	Je umewahi kusikia programu inayoitwa HJFMRI/WRP – programu ya ukatili wa kijinsia pia inajulikana kama programu ya GBV ya HJMRI?	NDIO . . . . 1<br>HAPANA 2	 ➢ 703a
702b	Je, ulishiriki katika uanzishwaji wa programu hii?	NDIO . . . . 1<br>HAPANA 2	

ID: [ ][ ][ ][ ][ ][ ][ ]

MASWALI na CHUJAJI		UFUNGUO WA MAKUNDI	NENDA
703a	Je umewahi kusikia “Men as Partners” au “MAP”, ambalo ni kundi linalofanya kazi na wanaume kuongeza uelewa kuhusu ukatili wa kijinsia, kupunguza ukubali wa ukatili wa kijinsia, na kuwashirikisha wanaume katika kusitisha ukatili dhidi ya wanawake na watoto?	NDIO . . . . 1<br>HAPANA . . . . .2<br>SIJUI 8	 ➢ 704a<br>➢ 704a
703b	Je, wewe binafsi unafahamu mwanaume au wanaume wowote ambao wameshiriki katika programu ya “Men As Partners”? Kama ndiyo ni mara ngapi wanashiriki?<br><br>*KAMA MHOJIWA ANAFAHAMU MWANAUME ZAIDI YA MMOJA ANAYESHIRIKI, ULIZA AKUELEZE KIASI CHA USHIRIKI WA MTU ANAYESHIRIKI ZAIDI*	Anamfahamu walau mtu mmoja anayeshiriki mara kwa mara. . . . . .1<br><br>Anamfahamu walau mtu mmoja anayeshiriki mara chache. . . . . 2<br><br>Anamfahamu walau mtu mmoja anayeshiriki mara moja. . . . . .3<br><br>Hamfahamu mtu aliyeshiriki. 4	
704a	Je umewahi kusikia kuhusu“Couples Connect,” ambayo ni programu ambayo inafanya kazi na wenza/wachumba kuboresha jinsi wanaume na wanawake wanavyohusiana (kwa mfano waume na wake) katika kuwasiliana?	NDIO . . . . .1<br>HAPANA . . . . 2<br>SIJUI 8	 ➢ 705a<br>➢ 705a
704b	Ni mara ngapi umeshiriki katika “Couples Connect”?	MARA NYINGI . . . . .1<br>MARA CHACHE . . . . 2<br>MARA MOJA . . . . 3<br>SIJAWAHI . . . . .4<br>SIJUI/SIKUMBUKI 8	
705a	Katika miezi 12 iliyopita je umewahi kusikia majadiliano ya kijamii—kwamba, mikutaniko iliyoitishwa na jamii kwa ajili ya wanajumuiya kukaa pamoja kujadili masuala ya ukatili wa kijinsia au ukatili dhidi ya wanawake na watoto?	NDIO . . . . .1<br>HAPANA . . . . 2<br>SIJUI 8	 ➢ 706a<br>➢ 706a
705b	Katika miezi 12 iliyopita, ni mara ngapi umeshiriki katika majadiliano kama hayo?	MARA NYINGI . . . . .1<br>MARA CHACHE . . . . 2<br>MARA MOJA . . . . 3<br>SIJAWAHI . . . . .4<br>SIJUI/SIKUMBUKI 8	

ID: [ ][ ][ ][ ][ ][ ][ ]

MASWALI na CHUJAJI		UFUNGUO WA MAKUNDI	NENDA
706a	Je, kuna vikundi vya kijamii vinavyojihusisha na ukatili wa kijinsia au ukatili dhidi ya wanawake na watoto?	NDIO . . . . .1<br>HAPANA . . . . 2<br>SIJUI 8	 ➢ 707a<br>➢ 707a
706b	Je, wewe ni mmojawapo wa wanakikundi kma hicho? kama ndivyo ni mara ngapi umeshiriki katika miezi 12 iliyopita?	MARA NYINGI . . . . .1<br>MARA CHACHE . . . . 2<br>MARA MOJA . . . . 3<br>SIJAWAHI .4<br>SIJUI/SIKUMBUKI 8	
707a	Je unafahamu kampeni zozote za vyombo vya habari (kama vile, matangazo, mashindano, redio, au ujumbe wa TV), katika miezi 12 iliyopita inayoelezea kuhusu ukatili wa kijinsia au ukatili dhidi ya wanawake na watoto?	NDIO . . . . 1<br>HAPANA 2	 ➢ 708
707b	Je kampeni hiyo ilikuwa na jina au msemo wowote? Je unaweza kunieleza jina?	MHOJIWA KATAMKA "KUWA MFANO WA KUIGWA". . . . . .1<br><br>MHOJIWA ALITAMKA MAJINA MENGINE TOFAUTI NA "KUWA MFANO WA KUIGWA". . . . . 2<br><br>MHOJIWA ALITAMKA KAMPENI HAINA JINA . . . . .3<br><br>MHOJIWA ALITAMKA KUWA INA JINA, ILA HALIKUMBUKII 8	 <br>➢ 708<br>➢ 708<br>➢ 708
707c	Katika miezi 12 iliyopita ni mara ngapi umeona au umesikia vipindi au simulizi za "Kuwa mfano wa kuigwa"?	MARA NYINGI . . . . 1<br>MARA CHACHE . . . . 2<br>MARA MOJA . . . . 3<br>SIJAWAHI . . . . .4<br>SIJUI/SIKUMBUKI 8	
708	Sasa ningependa kukuuliza kuhusiana na shughuli nyingine za kijamii ambazo zinaweza kuwa **zimehusisha taarifa dhidi ya ukatili wa kijinsia au ukatili kwa wanawake na watoto.** Nitasoma matukio na ningependa wewe kunieleza kama ulishiriki katika miezi 12 iliyopita, na kama ulishiriki ni mara ngapi.<br>SOMA MAKUNDI YOTE NA ULIZA KUHUSU NI MARA NGAPI.		

ID: [ ][ ][ ][ ][ ][ ][ ]

MASWALI na CHUJAJI		UFUNGUO WA MAKUNDI	NENDA
	a. Matukio yanayoendeshwa wakati wa matukio maalum, ya kitaifa na kimataifa (kwa mfano, NaneNane, SabaSaba, Siku ya UKIMWI duniani, Siku ya Wanawake Duniani n.k)	Ameshiriki:<br>MARA NYINGI. . . . . 1<br>MARA CHACHE . . . . 2<br>MARA MOJA (Katika miezi 12) . . . .3<br>SIJAWAHI. . . . . .4<br>SIJUI/SIKUMBUKI 8	 <br><br><br>➢ 708c<br>➢ 708c
	b. Je, kuna taarifa yoyote kuhusiana na ukatili wa kijinsia au ukatili dhidi ya wanawake ilitolewa au kujadiliwa?	NDIO . . . . .1<br>HAPANA . . . . .2<br>SIJUI/SIKUMBUKI 8	
	c. Shughuli za kimichezo za kijamii?	Ameshiriki:<br>MARA NYINGI. . . . . 1<br>MARA CHACHE . . . . 2<br>MARA MOJA (Katika miezi 12) .. . . .3<br>SIJAWAHI. . . . . .4<br>SIJUI/SIKUMBUKI 8	 <br><br><br>➢ 708e<br>➢ 708e
	d. Je, kuna taarifa yoyote kuhusiana na ukatili wa kijinsia au ukatili dhidi ya wanawake ilitolewa au kujadiliwa?	NDIO . . . . .1<br>HAPANA . . . . .2<br>SIJUI/SIKUMBUKI 8	
	e. Shughuli za kijamii au shughuli za kijamii kwa kutumia bodaboda?	Ameshiriki:<br>MARA NYINGI. . . . . 1<br>MARA CHACHE . . . . 2<br>MARA MOJA (Katika miezi 12) .. . . .3<br>SIJAWAHI. . . . . .4<br>SIJUI/SIKUMBUKI 8	 <br><br><br>➢ 709<br>➢ 709
	f. Je, kuna taarifa yoyote kuhusiana na ukatili wa kijinsia au ukatili dhidi ya wanawake ilitolewa au kujadiliwa?	NDIO . . . . .1<br>HAPANA . . . . .2<br>SIJUI/SIKUMBUKI 8	
709	Je, umewahi kusikia au kuona ujumbe wa kijumuiya unao walenga wanaume katika vilabu vya pombe kuhusiana na kuacha ukatili dhidi ya wanawake na watoto? Kama ndivyo, mara ngapi ndani ya miezi 12 iliyopita?	MARA NYINGI. . . . . 1<br>MARA CHACHE . . . . 2<br>MARA MOJA (Katika miezi 12) . . . .3<br>SIJAWAHI. . . . . .4<br>SIJUI/SIKUMBUKI 8	

ID: [ ][ ][ ][ ][ ][ ][ ]

MASWALI na CHUJAJI		UFUNGUO WA MAKUNDI	NENDA
710	Tofauti na tulivyoongea tayari, katika miezi 12 iliyopita, je umesikia kuhusiana na shughuli zingine zozote katika jamii yako zinazolenga kusitisha ukatili wa kijinsia au ukatili dhidi ya wanawake na watoto?	NDIO . . . . 1<br><br>a.[ ]TAJA SHUGHULI: _____________<br><br>b.[ ] TAJA SHUGHULI: ____________<br><br>c.[ ] TAJA SHUGHULI: ____________<br><br>d.[ ] TAJA SHUGHULI: ____________<br><br>HAKUNA 2	 <br><br><br><br><br><br><br><br><br>➢**Kipengele cha 8**
711	Katika miezi 12 iliyopita ni mara ngapi umeshiriki katika shughuli hizo?<br><br>**KWA KILA SHUGHULI ILIYOTAJWA KATIKA SWALI 710 (a-d), NAKILI MARA NGAPI AMESHIRIKI**.	a.<br>MARA NYINGI. . . . . 1<br>MARA CHACHE . . . . 2<br>MARA MOJA . . . . .3<br>SIJAWAHI. . . . . .4<br>SIJUI/SIKUMBUKI . . . . 8<br><br>b.<br>MARA NYINGI. . . . . 1<br>MARA CHACHE . . . . 2<br>MARA MOJA . . . . .3<br>SIJAWAHI. . . . . .4<br>SIJUI/SIKUMBUKI .. . . 8<br><br>c.<br>MARA NYINGI. . . . . 1<br>MARA CHACHE . . . . 2<br>MARA MOJA . . . . .3<br>SIJAWAHI. . . . . .4<br>SIJUI/SIKUMBUKI . . . . 8<br><br>d.<br>MARA NYINGI. . . . . 1<br>MARA CHACHE . . . . 2<br>MARA MOJA . . . . .3<br>SIJAWAHI. . . . . .4<br>SIJUI/SIKUMBUKI 8	

ID: [ ][ ][ ][ ][ ][ ][ ]

Kipengele cha 8 : Uelewa na mtazamo, mila za kijinsia, na tabia za mabadiliko

MHOJAJI:

Sasa ningependa kukuuliza kuhusu baadhi ya ukatili katika jamii yako na mawazo yako kuhusiana na hayo.

MASWALI na CHUJAJI		UFUNGUO WA MAKUNDI	NENDA
➢ Uelewa wa matukio katika jamii na majibu ya Jumuiya.			
801a	Je kumewahi kuwa matukio yoyote katika jamii unayoishi ndani ya miezi 12 iliyopita ambapo mwanamke amekatiliwa kingono ama kijinsia na mume wake au mwenzi wake?	NDIO . . . . 1<br>HAPANA . . . . 2<br>SIJUI 8	 ➢ 802a<br>➢ 802a
801b	Ni matukio mangapi unayoyafahamu?<br><br>**NAKILI IDADI YA MATUKIO KAMA YALIVYOSEMWA NA MHOJIWA.**	IDADI YA MATUKIO [ ] [ ] [ ] [ ]<br><br>SIJUI 9 9 9 8	
802a	Je kumewahi kuwa na matukio yoyote katika jamii unayoishi ndani ya miezi 12 iliyopita ambapo mtoto amekatiliwa kingono?	NDIO . . . . 1<br>HAPANA . . . . 2<br>SIJUI 8	 ➢ 803<br>➢ 803
802b	Ni matukio mangapi unayoyafahamu?<br><br>**NAKILI IDADI YA MATUKIO KAMA YALIVYOSEMWA NA MHOJIWA.**	IDADI YA MATUKIO [ ] [ ] [ ] [ ]<br><br>SIJUI 9 9 9 8	
803	Kwa maoni yako, katika miezi 12 iliyopita, je jumuiya yako imefanya kazi nzuri katika kushughulikia matukio ya ukatili kutoka kwa wanaume/wenzi na ukatili wa kingono kwa watoto?	Kazi nzuri sana . . . . 1<br>Kazi nzuri . . . . 2<br>Kazi ya wastani . . . . 3<br>Kazi hafifu .4<br>Kazi hafifu sana . . . . 5<br>Sijui 8	
➢ Uelewa wa Sheria/Sera na hatua za hivi karibuni za viongozi wa serikali za mtaa kuhusu Ukatili			
804	Je, unafahamu sheria zozote za Tanzaniaau sera dhidi ya ukatili kwa wanawake na watoto?	NAFAHAMU/NAELEWA SANA . . . . . .1<br>WASTANI. . . . . .2<br>SIFAHAMU . . . . 3<br>SIJUI. 8	
805	Katika miezi 12 iliyopita, ni mara ngapi viongozi wa serikali za mitaa wameongea kwa uwazi au wamechukua hatua kushughulikia suala la ukatili wa kijinsia au ukatili dhidi ya wanawake au watoto?	MARA NYINGI. . . . . .1<br>MARA CHACHE. . . . . 2<br>MARA MOJA. . . . . 3<br>HAWAJAWAHI. . . . . 4<br>SIJUI. 8	

ID: [ ] [ ] [ ] [ ] [ ] [ ] [ ] [ ]

➢ Maelezo au utambuzi wa Unyanyasaji dhidi ya wanawake (Imetoholewa kwenye DHS na utafiti wa CHAMPION)				
806	Kwa maoni yako, je, unachukulia hali hizi ni unyanyasaji dhidi ya mwanamke kama mume wake...	**NDIO**	**HPN**	**SIJUI**
	a. Akifanya kitu kumdhalilisha mbele ya wengine	1	2	8
	b. Akitishia kumdhuru au kumdhuru mtu wa jirani	1	2	8
	c. Akimtukana au akimfanya ajisikie vibaya	1	2	8
	d. Akimsukuma, kumpiga makofi, kumpiga ngumi au teke	1	2	8
	e. Akitishia kumdhuru kwa kisu, bunduki au silaha yoyote	1	2	8
	f. Akimlazimisha kwa nguvu, kufanya naye ngono hata kama akiwa hataki	1	2	8
	g. Akimlazimisha kufanya kitu fulani cha kingono ambacho hataki kufanya	1	2	8
	h. Akizuia matembezi yake	1	2	8
	i. Akimfukuza nyumbani	1	2	8
	j. Akiwa na mapenzi nje ya ndoa	1	2	8
	k. Akiwa anafanya maamuzi yote kuhusiana na jinsi mapato ya familia yanavyotumika ikiwa ni pamoja na mapato ya mke wake	1	2	8
807	Kwa maoni yako, unachukulia haya kama unyanyasaji dhidi ya mwanamke kama mtu mwingine tofauti na mume wake...	**NDIO**	**HPN**	**SIJUI**
	a. Akifanya kitu kumdhalilisha mbele ya wengine	1	2	8
	b. Akitishia kumdhuru au kumdhuru mtu wa jirani	1	2	8
	c. Akimtukana au akimfanya ajisikie vibaya	1	2	8
	d. Akimsukuma, kumpiga makofi, kumpiga ngumi au teke	1	2	8
	e. Akitishia kumdhuru kwa kisu, bunduki au silaha yoyote	1	2	8
	f. Akimlazimisha kwa nguvu, kufanya naye ngono hata kama akiwa hataki	1	2	8
	g. Akimlazimisha kufanya kitu fulani cha kingono ambacho hataki kufanya	1	2	8

ID: [ ][ ][ ][ ][ ][ ][ ]

➢ Maelezo au utambuzi wa Unyanyasaji dhidi ya watoto				
808	Nina kwenda kusoma baadhi ya kauli na ningependa uniambie kama wewe unakubaliana na hiyo kauli, unakubaliana kidogo, au hukubaliani.	NAKUBALI	NAKUBALI KIDOGO	SIKUBALI
	a. Mtoto anakua amekatiliwa kingono pale tu kitendo cha kujamiiana kimefanyika	1	2	3
	b. Mtoto anapokuwa amekatiliwa kingono, mara chache aliyemkatili ni mmoja wa familia	1	2	3
	c. Ni watoto wa kike tu ndio wanakatiliwa kingono	1	2	3
	d. Haiwezekani watoto wenye umri chini ya miaka 10 kufanyiwa ukatili wa kingono (sexually abused)	1	2	3
	e. Watoto wanaotoka kwenye familia zinazojiweza hawana uzoefu na ukatili wa kingono	1	2	3
➢ Mitazamo kuhusu ukatili ndani ya ndoa/majumbani (kutoka DHS)				
809	Katika mtazamo wako, je mume anaruhusiwa kumpiga mke wake katika matukio yafuatayo:	**NDIO**	**HPN**	**SIJUI**
	a. Kama akitoka bila kumpa taarifa mumewe/mpenzi wake?	1	2	8
	b. Kama akitelekeza watoto?	1	2	8
	c. Kama akibishana na mumewe/mpenzi wake?	1	2	8
	d. Kama akikataa kufanya ngono na mumewe/mpenzi wake?	1	2	8
	e. Kama akiunguza chakula?	1	2	8
➢ Mila kuhusu jinsia (Kipimo cha GEM: Ukatili na vidokezo vyake)				
810	Sasa nitasoma sentensi na ningependa unieleze kama unakubaliana nazo, unakubaliana kidogo au hukubaliani nazo.	NAKUBALI	NAKUBALI KIDOGO	SIKUBALI
	a. Kuna muda mwanamke anastahili kupigwa.	1	2	3
	b. Mwanamke anabidi kuvumilia unyanyasaji ili kutunza familia yake pamoja.	1	2	3
	c. Ni sawa kwa mwanaume kumpiga mke wake kama si mwaminifu.	1	2	3
	d. Mwanaume anaweza kumpiga mke wake kama anakataa kufanya naye ngono.	1	2	3
	e. Kama mtu akimtukana mwanaume, lazima alinde heshima yake hata kama ni kwa kutumia nguvu.	1	2	3
	f. Mwanaume anayetumia nguvu kwa mkewe ni suala binafsi ambalo halitakiwi kujadiliwa nje yao wao wawili.	1	2	3
➢ Mila kuhusu jinsia (Kipimo cha GEM: Shughuli za ndani na umilikaji vitu)				
811	a. Kubadili nepi, kumwosha, na kumlisha mtoto ni jukumu la mama.	1	2	3
	b. Jukumu la mwanamke ni kuitunza familia yake.	1	2	3
	c. Mume ndio anatakiwa kuamua kufanya manunuzi ya vitu muhimu vya kaya.	1	2	3
	d. Mwanaume ndio anatakiwa awe na uamuzi wa mwisho kuhusiana na maamuzi nyumbani.	1	2	3

ID: [ ][ ][ ][ ][ ][ ][ ]

	e. Mwanamke anatakiwa kumuheshimu mume wake katika kila kitu.		1	2	3
➢ Mabadiliko ya tabia ya Mhojiwa: Kuchukua hatua.					
812a	Katika miezi 12 iliyopita, je umeanzisha mazungumzo juu ya ukatili wa kijinsia au ukatili dhidi ya wanawake na watoto na mtu yeyote?	Ndio . . . . 1<br>Hapana 2	➢ 813a		
812b	Ulifanya haya mazungumzo na nani?<br><br>NAKILI YOTE ATAKAYOTAJA.	a. [ ] Mpenzi/wapenzi<br>b. [ ] Rafiki<br>c. [ ] Mwanafamilia<br>d. [ ] Jirani au mwanajumuiya<br>e. [ ] Mwingine, taja ______________________<br>f. [ ] Mwingine, taja ______________________			
813a	Katika miezi 12 iliyopita, je wewe mwenyewe umeshashuhudia vitendo vyovyote vya ukatili wa kijinsia au ukatili dhidi ya wanawake au watoto au umeshawahi kukutana na mtu ambaye amefanyiwa huu ukatili?	Ndio . . . . 1<br>Hapana 2	➢ 814		
813b	Je, ulichukua hatua yoyote kuzuia au kumsaidia mhanga? (Kwa mara zozote ulizoshuhudia)?	Ndio . . . . 1<br>Hapana 2	➢ 814		
813c	Ni hatua gani ulizochukua?<br><br>**NAKILI YOTE ATAKAYOTAJA, USISOME!.**	a. [ ] Nilimsikiliza mhanga na kumpatia usaidizi wa kihisia.<br>b. [ ] Nilimpatia taarifa kuhusu huduma na programu kwa ajili ya wahanga.<br>c. [ ] Nilimsindikiza mhanga kwenda kliniki au kwa ajenti wa usaidizi.<br>d. [ ] Niliongea na wanafamilia wa mhanga kuhusiana na unyanyasaji.<br>e. [ ] Nilijaribu kuzuia au nilizuia unyanyasaji<br>f. [ ] Nilitoa taarifa kwenye mamlaka<br>g. [ ] Nyingine, taja ______________________			
814	Je una mtazamo tofauti <u>sasa</u> kuhusu ukatili wa kijinsia au ukatili dhidi ya watoto na wanawake tofauti na ulivyokuwa unafikiria miezi 12 iliyopita?	Ndio . . . . .1<br>Hapana 2			
815	Kama ungeshududia unyanyasaji leo je ungekuwa na uwezo zaidi, au usingekuwa na uwezo wa kuchukua hatua kama ambavyo ungekuwa miezi 3 iliyopita?	UWEZEKANO MKUBWA . . . . 1<br>VILEVILE TU . . . . 2<br>UWEZEKANO MDOGO 3			

ID: [ ][ ][ ][ ][ ][ ][ ]

Kipengele cha 9: Kumaliza Mahojiano

901	**MHOJAJI:**<br>Tunaelekea mwisho wa mahojiano yetu. Kuna hatua moja tu ya mwisho.<br>Nitakupatia kadi. Katika kadi hii kuna picha mbili. Hakuna taarifa nyingine yoyote iliyopo kwenye kadi hii. Picha moja ni ya furaha na picha nyingine ni ya huzuni.<br>**MPE MHOJIWA KADI NA KALAMU.**<br>Bila kujali kile ulichoniambia, ningependa uweke alama kwenye picha isiyo na furaha kama kuna mtu alikuumiza **WEWE** kimaumbile, alikugusa kingono/kimapenzi, au alikulazimisha kufanya kitu chochote cha kingono/kimapenzi ambacho hukuridhia katika miezi 12 iliyopita.<br>Tafadhali weka alama kwenye picha yenye furaha kama hili halikukutokea katika miezi 12 iliyopita. Ukisha weka alama kwenye kadi, ikunje na kisha iweke ndani ya mfuko huu. Mfuko huu una kadi sawasawa na hii kutoka kwa wanawake wengi wengine. Hii itahakikisha sitajua jibu lako.<br>USIANGALIE JIBU, MWOMBE MHOJIWA KUWEKA ALAMA NA KUWEKA KADI ILIYOKUNJWA KWENYE MFUKO ULIO NA KADI NYINGINE. USIWEKE ALAMA YOYOTE KWENYE KADI	KADI IMEKAMILIKA……..1<br><br>KADI HAIJAKAMILIKA…..2
902	Nimekuuliza mambo mengi magumu. Je, kuongelea mambo haya kumekufanya ujisikie vipi?<br><br>**SOMA MAJIBU**	VIZURI/VIZURI ZAIDI . . . . 1<br>VIBAYA/VIBAYA ZAIDI . . . . .2<br>VILEVILE/ HAKUNA TOFAUTI 3
903	Sasa tumemaliza mahojiano. Je una maoni yoyote, au kuna chochote ambacho ungependa kunishirikisha?<br>**NAKILI MAONI**	

ID: [ ][ ][ ][ ][ ][ ][ ]

904	**HITIMISHO**<br><br>Ningependa kukushukuru sana kwa kutusaidia. Na ninashukuru pia kwa muda uliojitoa. Ninatambua kuwa maswali haya yameweza kuwa magumu kwako, lakini ni kwa kusikia tu kutoka kwa wanawake wenyewe ndio tunaweza kuelewa uhalisia wa afya yao na uzoefu wa maisha yao.<br><br>Kwa mfano kama utakuwa na nia au kama ukisikia kuhusiana na mtu mwingine atakayehitaji msaada kuhusiana na masuala ya unyanyasaji na vitu ambavyo tumekuwa tukiongele, nitakuachia jina na mawasiliano ya mtoa huduma katika kituo afya cha __________________ au Hospitali ya Center/_______________ambaye atakupa usaidizi. Mtu huyu ni mtoa huduma katika kituo hiki cha afya ambaye pia anafanya kazi na sisi katika utafiti huu. Yeye anatambua kuwa tunatoa taarifa hii kama sehemu ya Mahojiano. Kama ukienda unahakikishiwa atatunza chochote utakachosema atakitunza kwa usiri. Unaweza kwenda muda wowote utakapojisikia uko tayari, hivi karibuni hata baadaye.<br><br>**MWACHIE KARATASI YENYE JINA LA KITUO CHA AFYA KATIKA KUNDI LA UTAFITI, JINA LA MHUSIKA WA TATHMINI GBV WA ENEO HILO, NA TAARIFA YA MAWASILIANO**.<br><br>Kama ungependa pia, ninaweza kumwomba mhudumu huyu wa afya awasiliane nawe moja kwa moja. Na kama ungependa uelekezo/rufaa hii, nitachukua taarifa ya mawasiliano yako, pamoja na jina lako na sehemu salama au faragha ambapo ungependa mtaalamu mwingine aweze kukukuta. Nitanakili taarifa hii kwenye karatasi tofauti, ambayo haitahusishwa kwa namna yoyote na mahojiano tuliyomaliza. Na nitampa taarifa hizi kiongozi wangu ambaye ataifikisha karatasi hii kwa mtoa huduma. Taarifa uliyonishirikisha kwenye mahojiano haitatolewa kwa mtoa huduma.<br><br>Je ungependa nikukutanishe moja kwa moja na Mtoa huduma?<br>NDIO……………………1<br>HAPANA………………2<br><br>**KAMA MHOJIWA AKISEMA ‘NDIO’, JAZA KARATASI YA RUFAA, IWEKE KWENYE BAHASHA ILIYOANDALIWA/ISIYO NA ALAMA NA MPATIE KIONGOZI WA KIKUNDI.**

**MHOJAJI:**	Nakili muda wa kumaliza mahojiano: Saa [ ][ ]:[ ] [ ] (tumia saa 24)
	Mahojiano yamekamilika: _______Ndio, yamekamilika ______Hapana, Hayajakamilika<br><br>Kama mahojiano hayajakamilika, tafadhali toa sababu:<br><br>**Sahihi ya mhojaji:**<br>____

ID: [ ][ ][ ][ ][ ][ ][ ]

Project SOAR: HOUSEHOLD SURVEY

ENDLINE

CHUO KIKUU CHA AFYA NA SAYANSI
SHIRIKISHI CHA MUHIMBILI

CONFIDENTIAL

ID: [ ] [ ] [ ] [ ] [ ] [ ] [ ]

ID: [ ][ ][ ][ ][ ][ ][ ]

HOUSEHOLD IDENTIFICATION

STUDY CLUSTER NAME ___________________________ DISTRICT NAME ___________________ CODE: [ ] [ ]

ENUMERATION AREA (EA) NUMBER: [ ] [ ] [ ] [ ] [ ] [ ] [ ] [ ] [ ] [ ] [ ] [ ]

NAME OF WARD ____ CODE: [ ] [ ] [ ]

VILLAGE/HAMLET/ STREET ________________________________ HOUSE NUMBER (if available) _____________

OTHER INFO TO IDENTIFY HOUSEHOLD (if needed)
____

INTERVIEW PARTICULARS

	1st VISIT	2nd VISIT	3rd VISIT	FINAL VISIT
DATE (DD/MM/YYYY)<br>INTERVIEWERS ID<br>RESULT*	______________<br>______________<br>[ ] [ ]	______________<br>______________<br>[ ] [ ]	______________<br>______________<br>[ ] [ ]	DATE _ _ /_ _ / _ _ _ _<br>DD/ MM/ YYYY<br>RESULT* [ ] [ ]
NEXT VISIT, IF SCHEDULED<br>Date:<br>Time:	______________<br>______________	______________<br>______________		Total Number of visits: [ ]

* RESULT CODES:
01. Interview completed
02. No competent adult in household at time of visit
03. No eligible females living in household
04. Entire household absent for extended period of time
05. Refused by household head/representative
06. Selected participant not present; rescheduled
07. Selected participant not present; could not reschedule
08. Refused by selected female
09. Refused by parent
10. Other specify: ______________________

TOTAL # PERSONS IN HOUSEHOLD [ ][ ]

TOTAL # ELIGIBLE FEMALES AGED 15-49 [ ]

LINE # OF SELECTED ELIGIBLE FEMALE [ ]

STUDY CONSENT SUMMARY

For selected respondents ages 18-49, ADULT INFORMED CONSENT obtained?	YES [ ] NO [ ]
For selected respondents ages 15-17, PARENTAL CONSENT obtained?	YES [ ] NO [ ]
MINOR ASSENT obtained?	YES [ ] NO [ ]

QUESTIONNAIRE REVIEW AND PROCESSING			
TEAM LEADER SIGN OFF<br>NAME ________________<br>DATE _ _/_ _/ _ _ _ _<br>dd /mm/yyyy	QUESTIONNAIRE CHECKED BY<br>NAME<br>___________________<br>DATE _ _/_ _/ _ _ _ _<br>dd /mm/yyyy	OFFICE EDITOR<br>NAME<br>________________<br>DATE _ _/_ _/ _ _ _ _<br>dd /mm/yyyy	ENTERED BY<br>ENTRY 1: ________<br>ENTRY 2: ________

ID: [ ] [ ] [ ] [ ] [ ] [ ] [ ]

ELIGIBLE FEMALES IN THE HOUSEHOLD

INTERVIEWER OR TEAM LEADER INSTRUCTIONS:

For purposes of this study, a household is defined as: A group of individuals who usually live and eat together, whether or not they are related by blood or marriage, with one person, male or female, acknowledged as the head of the household. A household can consist of one person or many persons.

Several households may reside in one dwelling. If this is the case, randomly select one of the households (writing household labels on pieces of paper, then randomly drawing from a bag).

Upon reaching the house, ask to speak to the household head. If s/he is not available, ask to speak to any (competent) adult who resides in the household. If such individual is not there, ask for a time when s/he will return.

Then proceed with Brief Introduction of the Study. Then ask the following questions of the household head/adult.

1.	Please can you tell me how many people live here, and share food regularly?	TOTAL NUMBER OF<br>PEOPLE IN HOUSEHOLD [ ][ ]
2.	Is the head of the household male or female?	MALE ....1<br>FEMALE 2
3.	What is your relationship to the head of this household?	[ ] [ ] CIRCLE CODE BELOW AND ENTER HERE

CODES

22 HEAD
23 WIFE (PARTNER)
24 DAUGHTER
25 DAUGHTER IN LAW
26 GRANDDAUGHTER
27 MOTHER
28 MOTHER IN LAW
29 SISTER
30 SISTER IN LAW
31 ADOPTED/FOSTER/STEP DAUGHTER

32 HUSBAND(PARTNER)
33 SON
34 SON IN LAW
35 GRAND SON
36 FATHER
37 FATHER IN LAW
38 BROTHER
39 BROTHER IN LAW
40 ADOPTED/FOSTER/STEP SON

41 OTHER RELATIVE, SPECIFY: ____________________________
_

42 OTHER NON RELATIVE, SPECIFY: ____________________________
_

ID: [ ][ ][ ][ ][ ][ ][ ]

	LIST OF FEMALE HOUSEHOLD MEMBERS AGES 15-49 WHO HAVE LIVED IN HH FOR PAST 6 MONTHS	RELATIONSHIP TO HEAD OF HH	AGE
4.	Today I would like to talk to one girl or woman from your household. To enable me to identify with whom I should talk, would you please give me the first names of:<br>• all girls and women ages 15-49 who usually live in your household (and share food) AND<br>• who have lived in the household for the past 6 months	What is the relationship of NAME to the head of the household?<br><br>USE CODES BELOW	How old is NAME?
INTEVIEWER INSTRUCTIONS: List eligible females in the order given to you.			
**LINE #**	**NAME**	**CODE**	**AGE (YEARS)**
**1**			
**2**			
**3**			
**4**			
**5**			
**6**			
**7**			
**8**			

CODES

12 HEAD
13 WIFE (PARTNER)
14 DAUGHTER
15 DAUGHTER IN LAW
16 GRANDDAUGHTER
17 MOTHER
18 MOTHER IN LAW
19 SISTER
20 SISTER IN LAW
21 ADOPTED/FOSTER/STEP DAUGHTER
22 OTHER RELATIVE, SPECIFY:____________________________
23 OTHER NON RELATIVE, SPECIFY:________________________

ID: [ ] [ ] [ ] [ ] [ ] [ ] [ ]

RANDOM SELECTION OF PARTICIPANT

INTERVIEWER INSTRUCTIONS:

- Identify the last digit of the **ID Number**. Go to this **row** in the table below.
- Obtain the total **number of eligible women** from the listing above. Go to this column number.
- Follow the selected row and column to the cell where they meet and **circle the number** in the cell. This is the line number of the female to be selected for the interview (from table above).
- Write the name and line number of the selected female in the space below the table.

**LAST DIGIT OF THE ID NUMBER**	**TOTAL NUMBER OF ELIGIBLE WOMEN IN HOUSEHOLD**							
	**1**	**2**	**3**	**4**	**5**	**6**	**7**	**8**
**0**	1	2	2	4	3	6	5	4
**1**	1	1	3	1	4	1	6	5
**2**	1	2	1	2	5	2	7	6
**3**	1	1	2	3	1	3	1	7
**4**	1	2	3	4	2	4	2	8
**5**	1	1	1	1	3	5	3	1
**6**	1	2	2	2	4	6	4	2
**7**	1	1	3	3	5	1	5	3
**8**	1	2	1	4	1	2	6	4
**9**	1	1	2	1	2	3	7	5

NAME OF SELECTED WOMAN_____________________ LINE NUMBER OF SELECTED WOMAN [ ]

AGE VERIFICATION

Interviewer instructions:
Ask to speak with the selected girl/woman. Ask her age and date of birth and record:

Reported age: _ _ **Date of birth: _ _/_ _/_ _ _ _** **Calculated age: _ _**

- If reported or calculated age is 20 or younger:
  - Ask to see written documentation. This could include birth certificate, clinic card, passport, religion card, ID card, or other official document.
  - If documentation indicates she is aged of 15-17, continue with Parental Consent process.
    If documentation indicates she is aged 18+, continue with Adult Informed Consent.
  - If no documentation is available to verify age, then remove the name of this person from the list of eligible female members and move up all other names by one row. Repeat the random selection process, and then age verification, as indicated by reported age.

  [ ] Tick here if you had to repeat the random selection because of no age documents

- If reported or calculated age is age 21-49, continue with the Adult Informed Consent process.

  If the selected girl/woman is not available, reschedule a visit. If her age on the roster of eligible females is 20 or younger, before you leave attempt to verify her age through written documentation with others in the household.

ID: [ ][ ][ ][ ][ ][ ][ ]

DODOSO YA MHOJIWA

Utangulizi

MHOJAJI:

Kama ulivyojifunza, dhumuni la tafiti hii ni kupima uzito wa programu na huduma zinazotolewa kwenye vituo vya afya na katika jamii kwa ajili ya kuleta maendeleo katika afya ya wanawake na watoto. Nitakuuliza maswali na kurekodi majibu yake kwenye hii fomu. Mahojiano yatachukua takribani saa 1 – 11/2. Kama tulivyoainisha kwenye fomu ya idhini, unaweza kukuta baadhi ya maswali ni ya undani zaidi. Si lazima ujibu swali lolote litalofanya ujisikie vibaya. Na pia unaweza sitisha mahojiano muda wowote. Tutaanza mahojiano pindi tu tuko sehemu ya faragha na yenye utulivu. Mtu yeyote atakapoingia tutasimamisha mahojiano na kutafuta sehemu nyingine yenye utulivu kama italazimu, ili kuhakikisha unaridhika kuendelea na mahojiano.

NAKILI MUDA WA KUANZA MAHOJIANO: [ ] [ ]:[ ] [ ] (tumia saa 24)

Kipengele cha 1: Taarifa za awali za mhojiwa na watoto wake

MASWALI NA CHUJAJI		UFUNGUO WA MAKUNDI	NENDA
MHOJAJI: Kama hutojali, ningependa kuanza kwa kuuliza maswali kuhusu wewe, familia na kaya yako.			
➢ **Sifa za kaya**			
001	Nini chanzo kikuu cha maji ya kunywa kwa ajili ya wanakaya wako?	MAJI YA BOMBA . . . . .1<br>*YANAYOFIKA KWENYE MAKAZI, KWENYE KIWANJA/UWAZI, BOMBA LA JUMUIYA, BOMBA LA JIRANI*<br>MAJI KUTOKA KISIMA CHA WAZI .. . . 2<br>*KATIKA MAKAZI, KATIKA KIWANJA/MAKAZI, CHA JUMUIYA, AU CHA JIRANI*<br>MAJI KUTOKA KISIMA KILICHOFUNIKWA . . . 3<br>*KINACHOLINDWA KATIKA MAKAZI, KWENYE UWAZI/KIWANJA, KINACHOLINDWA CHA JUMUIYA, AU CHA JIRANI*<br>MAJI YA WAZI . . . . .4<br>*MFEREJI, KIJITO, BWAWA/ZIWA,LAMBO*<br>MAJI YA MVUA . . . . 5<br>MAJI YA MALORI . . . . 6<br>MCHUUZI WA MAJI . . . . .7<br>MAJI YA CHUPA . . . . 8<br>NYINGINE_____________________________ 9	
002	Ni aina gani ya choo ambacho kaya yako huwa inatumia?	CHA KUFLASHI/KUFLASH KWA KUMIMINA KWENDA KWENYE: TANKI LA MAJI TAKA, CHOO CHA SHIMO AU VINGINEVYO . . . . 1<br>CHOO CHA SHIMO . . . . 2<br>COMPOSTING TOILET/ECOSAN . . . . 3<br>NDOO . . . . .4<br>HAKUNA CHOO/KICHAKA/UWANJA . .. . 5<br>NYINGINE . . . . .9<br>(TAJA) _________________________________	
003	Je kaya yenu ina umeme?	NDIO . . . . 1<br>HAPANA 2	

ID: [ ][ ][ ][ ][ ][ ][ ]

MASWALI NA CHUJAJI		UFUNGUO WA MAKUNDI	NENDA
➢ **Umri wa Mhojiwa**			
101	Umezaliwa mwezi na mwaka gani?	MWEZI. . . . . [ ] [ ]<br>SIFAHAMU MWEZI. . . . . 9 8<br>MWAKA . . . . [ ][ ][ ][ ]<br>SIFAHAMU MWAKA. 9 9 9 8	
102	Ulikuwa na umri gani kwenye siku yako yako ya kuzaliwa ya mwisho?<br><br>**FANANISHA UMRI NA Q 101 KISHA SAHIHISHA UMRI KAMA KUNA ULAZIMA**	UMRI KWA HESABU YA MIAKA. . . . . [ ] [ ]	
➢ **Elimu/Taaluma ya Mhojiwa**			
103	Unaweza kusoma na kuandika?	NDIO . . . . 1<br>HAPANA. 2	
104	Je, umewahi kusoma shule?	NDIO . . . . 1<br>HAPANA. 2	 ➢ 107
105	Je, ni kiwango gani cha juu cha Elimu ulichowahi kufikia?	ELIMU YA AWALI . . . . .0<br>ELIMU YA MSINGI . . . . 1<br>ELIMU YA MAFUNZO BAADA YA ELIMU YA MSINGI . . . . .2<br>ELIMU YA SEKONDARI . . . . 3<br>ELIMU YA MAFUNZO BAADA YA SEKONDARI .4<br>CHUO KIKUU . . . . 5<br>SIJUI 8	 <br><br><br><br><br>➢ 107
106	Je, ni kiwango gani ulihitimu katika hatua hiyo?<br><br>**KAMA ALIHITIMU KATIKA MUDA USIOZIDI MWAKA MMOJA KATIKA HATUA HIYO NAKILI '00'.**	DARASA/KIDATO/MWAKA . . . . [ ] [ ]<br><br>SIJUI. 9 8	
➢ **Kazi ya Mhojiwa na umilikaji**			
107	Kama unavyofahamu, kuna wanawake ambao hufanya kazi na kulipwa kwa vitu au fedha. Wengine huuza vitu, kuwa na biashara ndogondogo au hufanya kazi katika shamba la familia au katika biashara ya familia. Katika miezi 12 iliyopita, tofauti na shughuli zako za nyumbani, umefanya shughuli	NDIO . . . . 1<br>HAPANA 2	 ➢ 111

ID: [ ][ ][ ][ ][ ][ ][ ]

MASWALI NA CHUJAJI		UFUNGUO WA MAKUNDI	NENDA
	yoyote au kitu chochote ambacho umelipwa fedha au kwa vitu?		
108	Je, una kazi gani--Namaanisha ni aina ipi mahsusi ya kazi umekuwa ukifanya katika kipindi cha miezi 12 iliyopita?	**c) NAKILI ATACHOKISEMA:** ______________________<br>**d) KODI BAADAE**<br>Kitaalam/Ufundi/Utawala. . . . . .1<br>Ukarani. . . . . 2<br>Bidhaa na Huduma . . . . 3<br>Utendaji wenye maarifa . . . . .4<br>Utendaji usio Maarifa . . . . .5<br>Kazi za Jamii. . . . . .6<br>Mkulima. . . . . 7<br>Mwanafunzi . . . . .8<br>"R" Hajui kundi 9	
109	Je, wewe hufanya kazi siku zote katika mwaka au hufanya kazi kwa msimu au mara moja baada ya muda?	SIKU ZOTE KATIKA MWAKA . . . . .1<br>KWA MSIMU . . . . .2<br>MARA MOJA KWA MUDA. 3	
110	Je, unalipwa/unapata kwa fedha taslimu au kwa hali kwenye hii kazi unayofanya au hulipwi kabisa?<br>Kwa hali namaanisha malipo yanayotolewa kwa mali, bidhaa au huduma -- tofauti na fedha	FEDHA TASLIMU TU. . . . . .1<br>FEDHA TASLIMU NA HALI . . . . .2<br>KWA HALI TU . . . . 3<br>SILIPWI 4	
111	Je unamiliki hii nyumba au nyumba nyingine yoyote peke yako au pamoja na mtu mwingine?	NAMILIKI PEKE YANGU . . . . 1<br>TUNAMILIKI PAMOJA .2<br>SIMILIKI. 3	
112	Je unamiliki ardhi yoyote, peke yako au pamoja na mtu mwingine?	NAMILIKI PEKE YANGU . . . . 1<br>TUNAMILIKI PAMOJA .2<br>SIMILIKI. 3	
➢ **Uzao na watoto**			
MHOJAJI: Sasa ningependa kukuuliza kuhusu mara zote ulizojifungua katika maisha yako.			
113	a) Je, una ujauzito kwa sasa au uwewahi kubeba ujauzito ndani ya miezi 12 iliyopita?	NDIO . . . . .1<br>HAPANA . . .. 2<br>SIJUI 8	
	b) Umeishawahi kujifungua?	NDIO . . . . .1<br>HAPANA . . .. 2<br>SIJUI 8	➢ 120

ID: [ ][ ][ ][ ][ ][ ][ ]

MASWALI NA CHUJAJI		UFUNGUO WA MAKUNDI		NENDA
114	Je una watoto wowote uliojifungua unaoishi nao?	NDIO . . . . .1<br>HAPANA . . .. 2<br>SIJUI 8		➢ 116
115	Watoto wangapi wa kiume unaoishi nao? Na, watoto wangapi wa kike unaoishi nao?<br><br>**KAMA HAKUNA, NAKILI '00'**	a) WA KIUME NYUMBANI<br>b) WA KIKE NYUMBANI	[ ][ ]<br>[ ][ ]	
116	Je, una watoto wowote wa kiume au wa kike uliojifungua walio hai usioishi nao?	NDIO . . . . 1<br>HAPANA 2		➢ 118
117	Je, watoto wangapi wa kiume wanaoishi ambao huishi nao? Na, una watoto wangapi wa kike wanaoishi ambao huishi nao?<br><br>**KAMA HAKUNA, NAKILI '00'**	a) WA KIUME KWENGINE .. . .<br>b) WA KIKE KWENGINE . . . . . .<br>.	[ ][ ]<br>[ ][ ]	
118	Umeshawahi kujifungua mtoto wa kiume au wa kike ambaye alikuwa mzima baadaye kufariki?	NDIO . . . . 1<br>HAPANA 2		➢ 120
119	Watoto wangapi wamefariki?	IDADI YA WATOTO WALIOFARIKI . . . . [ ] [ ]<br>SIJUI 9 8		
➢ **Kuwa na Mshirika/Mpenzi:Sasa na ndani ya miezi 12 iliyopita**				
120	Hali yako ya ndoa sasa hivi ikoje?	Nimeolewa. . . . . .1<br>Naishi na mwanaume kama tumeoana . . . . .2<br>Mjane. . . . . .3<br>Tumetengana . . . . 4<br>Mtalikiwa . . . . .5<br>Sijawahi kuolewa/Kuishi na mwanaume 6		➢ 124<br>➢ 124<br><br><br><br>➢ 122
121	<u>Je, katika miezi 12 iliyopita</u>, umeolewa, umeishi na mwanaume kama mmeoana?	Ndio, nimeolewa au ninaishi na mwanaume kama tumeoana. . . . . 1<br>Hapana, sijaolewa au kuishi na mwanaume . 2		
122	Je una rafiki wa kiume ambaye unamchukulia kama mpenzi wako hata kama hamuishi pamoja au hamjamiiani?	NDIO . . . . 1<br>HAPANA 2		➢ 124

ID: [ ][ ][ ][ ][ ][ ][ ]

MASWALI NA CHUJAJI		UFUNGUO WA MAKUNDI	NENDA
123	Katika muda wowote ndani ya miezi 12 iliyopita, Je umekuwa na rafiki wa kiume ambaye unamchukulia kama mpenzi wako hata kama hamuishi pamoja au hamjamiiani?	Ndio nimekuwa na mpenzi kwenye miezi 12 iliyopita . . . . 1<br><br>Hapana, sikuwahi kuwa na mpenzi kwenye miezi kumi na mbili iliyopita.. 2	

UFUPISHO<br>**Maelekezo kwa Mhojaji: Angalia majibu ya maswali na. 120-123 na nakili taarifa 2 zifuatazo. Taarifa hizi zitatumika kuanzia mwanzo wa kipengele cha 3.**			
**124**	**Mpenzi kwa miezi 12 iliyopita?**	**NDIO . . . . .1**<br>Kama masharti yoyote kati ya yafuatayo yamefikiwa:<br>Na. 120 = 1 au 2<br>Na. 121=1<br>Na. 122=1<br>Na. 123=1<br><br>**HAPANA . . . . 2**<br>Kama HAKUNA sharti hapo juu lililofikiwa	
**125**	**Mpenzi sasa?**	**NDIO . . . . .1**<br>Kama masharti yoyote kati ya yafuatayo yamefikiwa:<br>Na. 120 = 1 au 2<br>Na. 122=1<br><br>**HAKUNA . . . . 2**<br>Kama HAKUNA sharti hapo juu lililofikiwa	

ID: [ ][ ][ ][ ][ ][ ][ ]

Kipengele cha 2: Afya ya Mhojiwa, tabia za afya, na historia ya kujamiiana

MHOJAJI:
Sasa ningependa kukuuliza maswali kuhusiana na afya yako na sehemu nyingine muhimu kuhusiana na maisha ya mwanamke yanavyohusiana na afya.

MASWALI NA CHUJAJI		UFUNGUO WA MAKUNDI	NENDA
➢ **Afya na utumiaji wa Huduma za Afya**			
201	Kwa ujumla afya yako unaweza kuiweka kundi lipi, nzuri sana, nzuri, wastani, dhoofu, dhoofu sana?	NZURI SANA . . . . 1<br>NZURI . . . . 2<br>WASTANI . . . . 3<br>DHOOFU . . . . .4<br>DHOOFU SANA 5	
202	Katika miezi 12 iliyopita, ulipata hitaji ya kupata huduma ya afya kwa ajili yako?	NDIO . . . . .1<br>HAPANA 2	
203	Katika miezi 12 iliyopita, umetafuta huduma zozote kutoka zahanati, kituo cha afya, au hospitali kwa ajili yako?	NDIO . . . . .1<br>HAPANA 2	 ➢ 205
204	Katika miezi 12 iliyopita, je mtoa huduma aliwahi kukuuliza kama unakabiliana na vitendo vya kikatili?	NDIO . . . . .1<br>HAPANA . . . . .2<br>SIJUI/SIKUMBUKI 8	

205	Ni aina gani ya huduma za VVU zinazopatikana kwa wanawake katika eneo lako?<br>**SOMA KILA JIBU HAPA CHINI NA ZUNGUSHIA MSIMBO SAHIHI**	**Zipo**	**Hazipo**	**Sijui**	
	a. Upimaji VVU na Ushauri	1	2	8	
	b. Upimaji na ushauri wa VVU kwa mwenza	1	2	8	
	c. Kukinga maambukizi kutoka kwa mama kwenda kwa Mtoto	1	2	8	
	d. Matibabu ya dawa za kupunguza makali ya VVU	1	2	8	
	e. Matibabu mengine kama vile tiba za afya kwa ajili ya magonjwa nyemelezi au hali nyingine zinazotokana na kuwa na UKIMWI	1	2	8	
	f. Tiba kinga ya VVU mara baada ya maambukizi (PEP)	1	2	8	
	g. Elimu/Taarifa za kujikinga	1	2	8	
	h. Tiba saidizi ya kiakili au kijamii/Kikundi cha usaidizi wa kiakili na kijamii	1	2	8	

206	Sitaki kufahamu majibu, lakini je, umewahi kupima kuona kama umeambukizwa VVU?	NDIO . . .. .1<br>HAPANA . . . . .2<br>SIJUI 8	 ➢ 209<br>➢ 209
207	Je, mara ya mwisho umepima lini?	CHINI YA MIEZI 12 ILIYOPITA .. . . 1<br>MIEZI 12 - 24 ILIYOPITA. . . . . .2<br>ZAIDI YA MIAKA 2 ILIYOPITA . . . . 3<br>SIJUI 8	

ID: [ ][ ][ ][ ][ ][ ][ ]

208	Tena, sitaki kufahamu majibu, lakini je, ulipewa majibu?	NDIO . . . . .1<br>HAPANA . . . . 2<br>SIJUI 8	
MHOJAJI:<br>Sasa naelekea kukuuliza maswali ambayo unaweza kujisikia ni ya undani zaidi. Tafadhali kumbuka kuwa yote utakayoniambia leo yatatunzwa kwa siri na unaweza kuruka swali lolote usilojisikia huru kulijibu.			
➢ **Matumizi ya pomble na Vilevi**			
209	Ndani ya miezi 12 iliyopita umewahi kunywa pombe?	NDIO . . . . .1<br>HAPANA 2	➢ 212
210	Je, ni mara ngapi unakunywa na kufikia hatua ya kulewa, mara zote, mara chache tu, au sijawahi?<br>Kwa kulewa, namaanisha kujisikia kulewa au kuchangamka, au kupoteza uwezo wako wa kutambua.	MARA ZOTE . . . . 1<br>MARA CHACHE . . . . .2<br>MARA MOJA KATIKA MIEZI 12. . . . . .3<br>SIJAWAHI . . . . .4<br>SIJUI 8	
211	Huwa unakunywa na nani?<br><br>**[WEKA ALAMA YA TIKI KWA YOTE MHOJIWA ATAKAYOJIBU; USIMSOMEE]**	[ ] a. PEKE YANGU<br>[ ] b. PAMOJA NA MUME WANGU/MPENZI<br>[ ] c. NA RAFIKI YANGU WA KIUME<br>[ ] d. NA WAKWE<br>[ ] e. NA WANAFAMILIA WANGU<br>[ ] f. NA RAFIKI ZANGU WA KIKE<br>[ ] g. NA RAFIKI ZANGU WA KIUME<br>[ ] h. VINGINEVYO: TAJA __________________	
212	Ndani ya miezi 12 iliyopita, Je, umewahi kuvuta bangi?	NDIO . . . . .1<br>HAPANA 2	➢ 215
213	Je, ni mara ngapi ulivuta bangi mpaka kufikia kulewa: mara zote, mara chache tu, au sijawahi?	MARA ZOTE . . . . 1<br>MARA CHACHE . . . . .2<br>MARA MOJA KATIKA MIEZI 12. . . . . .3<br>SIJAWAHI . . . . .4<br>SIJUI 8	
214	Ni nani ulikuwa unavuta naye bangi?<br><br>**[WEKA ALAMA YA TIKI KWA YOTE MHOJIWA ATAKAYOJIBU; USIMSOMEE]**	[ ] a. PEKE YANGU<br>[ ] b. PAMOJA NA MUME WANGU/MPENZI<br>[ ] c. NA RAFIKI YANGU WA KIUME<br>[ ] d. NA WAKWE<br>[ ] e. NA WANAFAMILIA WANGU<br>[ ] f. NA RAFIKI ZANGU WA KIKE<br>[ ] g. NA RAFIKI ZANGU WA KIUME<br>[ ] h. VINGINEVYO: TAJA __________________	

ID: [ ][ ][ ][ ][ ][ ][ ]

215	Katika muda wowote katika maisha yako (kama mtoto au mtu mzima, kuna mtu yeyote ameishawahi kukulazimisha kwa namna yoyote kufanya naye ngono au kufanya naye tendo lolote la kingono?	NDIO . . . . .1<br>HAPANA . . . . .2<br>HAKUJIBU 9	
216	Ulikuwa na umri gani ulipofanya ngono kwa mara ya kwanza?	UMRI KATIKA MIAKA . . . . [ ] [ ]<br>SIJUI . . . . 98<br>Sijawahi kufanya ngono 0 0	➢ CHUJAJI HAPO CHINI
217	Kwa mara ya kwanza ulivyofanya ngono, unaweza kusema ulifanya kwa sababu ulitaka kufanya au ulilazimishwa kufanya bila ridhaa yako?	NILITAKA . . . . 1<br>NILILAZIMISHWA . . . . 2<br>HAKUJIBU 9	
218	Kwa ujumla, Je umefanya ngono na watu tofauti wangapi katika ya miezi 12 iliyopita?	Idadi ya wapenzi miezi 12 iliyopita [ ] [ ] [ ]<br>SIJUI . . . . 9 9 8<br>HAKUJIBU 9 9 9	

CHUJAJI	
**CHUJAJI YA MHOJAJI:**<br>MWENZI/MWANDANI KATIKA MIEZI 12 ILIYOPITA? **Angalia swali na. 124**	
Kama swali Na. **124 = NDIO**,<br>ENDELEA **KIPENGELE CHA 3**	Kama swali Na. **124=HAPANA**,<br>**NENDA KIPENGELE CHA 5**

ID: [ ] [ ] [ ] [ ] [ ] [ ] [ ]

Kipengele cha 3 : Tabia za MPENZI/MWANDANI

MAELEKEZO KWA MHOJAJI:

MASWALI YAFUATAYO YAULIZWE KWA MPAKA MPENZI/MWANDANI WATATU AMBAO MHOJIWA AMEKUWA NAO KATIKA MIEZI 12 ILIYOPITA.

ANZA KWA KUULIZA KUHUSU MWANDANI/MPENZI WA SASA KAMA ANAYE, (Swali Na. 125=NDIYO), AU MWANDANI/MPENZI WA HIVI KARIBUNI. MHOJIWA (R) ANAWEZA KUWA NA WANDANI/WAPENZI KWA MPIGO kwa mfano, YUKO NDANI YA NDOA ILA PIA ANA RAFIKI WA KIUME. HATUJA ULIZA HILI HAPO NYUMA ILA MASWALI YANAYOFUATIA YANARUHUSU KUHOJI. KWA HALI HIYO UNAWEZA KUSEMA KUWA NI MWENZI WA MSINGI. HATA HIVYO MTIRIRIKO WA JINSI YA KUHOJI WANDANI/WAPENZI SI TATIZO.

ULIZA MASWALI MOJA KWA MOJA KUANZIA 301 MPAKA 424 (KIPENGELE CHA NNE).
KISHA FUATA MAELEKEZO KATIKA SWALI LA 424, KWA KUANZA TENA NA 301 KAMA MHOJIWA AMEKUWA NA ZAIDI YA MPENZI/MWANDANI MMOJA KATIKA MIEZI 12 ILIYOPITA.

MHOJAJI:

Kinachofuata nitakuuliza maswali kuhusiana na mwandani/mpenzi wako wa kiume wa sasa. Tena, ningependa kukuhakikishia kuwa majibu yako yatatunzwa kwa usiri mkubwa na hayataelezwa kwa mtu yeyote. Kama tukifikia swali lolote ambalo usingependa kujibu na tutaweza kuendelea na swali linalofuata. Umenieleza awali kuwa kwa sasa una mwandani/mpenzi au kwa mwaka uliopita ulikuwa una mpenzi/mwandani…

MASWALI NA CHUJAJI		a.WA SASA, HIVI PUNDE, AU MPENZI WA MSINGI		b.MPENZI MWINGINE (1)		c.MPENZI MWINGINE (2)	
301	Nini uhusiano wako na mwandani/mpenzi wako kwako?	MUME . . . . 1<br>MPENZI MNAISHI PAMOJA. . . . . 2<br>MUME WA ZAMANI . . . . 3<br>MPENZI WA ZAMANI WA KUISHI PAMOJA . . . . .4<br>RAFIKI WA KIUME (ASIYEISHI NA MHOJIWA) . . . . .5<br>RAFIKI WA KIUME WA ZAMANI 6		MUME . . . . 1<br>MPENZI MNAISHI PAMOJA. . . . . 2<br>MUME WA ZAMANI . . . . 3<br>MPENZI WA ZAMANI WA KUISHI PAMOJA . . . . .4<br>RAFIKI WA KIUME (ASIYEISHI NA MHOJIWA) . . . . .5<br>RAFIKI WA KIUME WA ZAMANI 6		MUME . . . . 1<br>MPENZI MNAISHI PAMOJA. . . . . 2<br>MUME WA ZAMANI . . . . 3<br>MPENZI WA ZAMANI WA KUISHI PAMOJA . . . . .4<br>RAFIKI WA KIUME (ASIYEISHI NA MHOJIWA) . . . . .5<br>RAFIKI WA KIUME WA ZAMANI 6	
302	Kwa kukadiria alikuwa na umri gani kulinganisha na wewe?	MDOGO KUKUZIDI . . . . . 1<br>UNALINGANA NAYE . . . . 2<br>MWAKA 1-9 ZAIDI . . . . . 3<br>MIAKA 10+ ZAIDI . . . . . . 4<br>SIJUI 8		MDOGO KUKUZIDI . . . . . 1<br>UNALINGANA NAYE . . . . 2<br>MWAKA 1-9 ZAIDI . . . . . 3<br>MIAKA 10+ ZAIDI . . . . . . 4<br>SIJUI 8		MDOGO KUKUZIDI . . . . . 1<br>UNALINGANA NAYE . . . . 2<br>MWAKA 1-9 ZAIDI . . . . . 3<br>MIAKA 10+ ZAIDI . . . . . . 4<br>SIJUI 8	
303	Amewahi kusoma shule?	NDIO 1		NDIO 1		NDIO 1	
		HAPANA . . 2<br>SIJUI 8	➢ 306<br>➢ 306	HAPANA . . 2<br>SIJUI 8	➢ 306<br>➢ 306	HAPANA . . 2<br>SIJUI 8	➢ 306<br>➢ 306

ID: [ ][ ][ ][ ][ ][ ][ ]

MASWALI NA CHUJAJI		a.WA SASA, HIVI PUNDE, AU MPENZI WA MSINGI	b.MPENZI MWINGINE (1)	c.MPENZI MWINGINE (2)
304	Je, ni hatua gani ya juu ya elimu amefikia?	ELIMU YA AWALI . . . . . . 0<br>ELIMU YA MSINGI . . . . . .1<br>ELIMU YA MAFUNZO BAADA YA ELIMU YA MSINGI . . . . 2<br>ELIMU YA SEKONDARI . . 3<br>ELIMU YA MAFUNZO BAADA YA EMILU YA SEKONDARI . . . . .4<br>CHUO KIKUU . . . . 5<br>SIJUI . . . . 8<br>(NENDA Q 306)	ELIMU YA AWALI . . . . . . 0<br>ELIMU YA MSINGI . . . . . .1<br>ELIMU YA MAFUNZO BAADA YA ELIMU YA MSINGI . . . . 2<br>ELIMU YA SEKONDARI . . 3<br>ELIMU YA MAFUNZO BAADA YA EMILU YA SEKONDARI . . . . .4<br>CHUO KIKUU . . . . 5<br>SIJUI . . . . 8<br>(NENDA Q 306)	ELIMU YA AWALI . . . . . . 0<br>ELIMU YA MSINGI . . . . . .1<br>ELIMU YA MAFUNZO BAADA YA ELIMU YA MSINGI . . . . 2<br>ELIMU YA SEKONDARI . . 3<br>ELIMU YA MAFUNZO BAADA YA EMILU YA SEKONDARI . . . . .4<br>CHUO KIKUU . . . . 5<br>SIJUI . . . . 8<br>(NENDA Q 306)
305	Je, ni kiwango (darasa/mwaka) gani cha juu alikamilisha hatua hiyo?<br><br>**KAMA AMEKAMILISHA CHINI YA MWAKA MMOJA NAKILI '00'**	DARASA/KIDATO/MWAKA . . . .[ ] [ ]<br><br>SIJUI 9 8	DARASA/KIDATO/MWAKA . . . .[ ] [ ]<br><br>SIJUI 9 8	DARASA/KIDATO/MWAKA . . . . [ ] [ ]<br><br>SIJUI 9 8
306	Anafanya kazi gani? Nikimaanisha aina gani halisi ya kazi anayofanya?	**3) NAKILI ATACHOKISEMA:**<br><br>__________________<br><br>**4) KODI BAADAE:**<br>Kitaalam/Ufundi/Utawala. . . . . 1<br>Ukarani . . . . 2<br>Bidhaa na huduma<br>Utendaji wenye . . . . 3<br>maarifa . . . . 4<br>Utendaji usio Maarifa . . . . 5<br>Kazi za Jamii . . . . 6<br>Mkulima . . . . 7<br>Mwanafunzi . . . . 8<br>**"R"** hajui 9	**3) NAKILI ATACHOKISEMA:**<br><br>__________________<br><br>**4) KODI BAADAE:**<br>Kitaalam/Ufundi/Utawala. . . . . 1<br>Ukarani . . . . 2<br>Bidhaa na huduma<br>Utendaji wenye . . . . 3<br>maarifa . . . . 4<br>Utendaji usio Maarifa . . . . 5<br>Kazi za Jamii . . . . 6<br>Mkulima . . . . 7<br>Mwanafunzi . . . . 8<br>**"R"** hajui 9	**3) NAKILI ATACHOKISEMA:**<br><br>__________________<br><br>**4) KODI BAADAE:**<br>Kitaalam/Ufundi/Utawala. . . . . .1<br>Ukarani . . . . 2<br>Bidhaa na huduma<br>Utendaji wenye . . . . 3<br>maarifa . . . . 4<br>Utendaji usio Maarifa . . . . 5<br>Kazi za Jamii . . . . 6<br>Mkulima . . . . 7<br>Mwanafunzi . . . . 8<br>**"R"** hajui 9

ID: [ ][ ][ ][ ][ ][ ][ ]

MASWALI NA CHUJAJI		a.WA SASA, HIVI PUNDE, AU MPENZI WA MSINGI		b.MPENZI MWINGINE (1)		c.MPENZI MWINGINE (2)	
307	Je, ana/aliwahi kuwa na wake wengine au anaishi/aliwahi kuishi na wanawake wengine kama vile wameoana?	NDIO . . . . .1<br>HAPANA . . . . .2<br>SIJUI 8		NDIO . . . . .1<br>HAPANA . . . . .2<br>SIJUI 8		NDIO . . . . .1<br>HAPANA . . . . .2<br>SIJUI 8	
308	Ndani ya mwaka mmoja uliopita, je mpenzi wako alishawahi kunywa pombe?	NDIO 1		NDIO 1		NDIO 1	
		HAPANA . . . . 2<br>SIJUI 8	➢ 311<br>➢ 311	HAPANA . . . . 2<br>SIJUI 8	➢ 311<br>➢ 311	HAPANA . . . . 2<br>SIJUI 8	➢ 311<br>➢ 311
309	Je, ni mara ngapi anakunywa na kufikia hatua ya kulewa, mara zote, mara chache tu, au hajawahi?<br><br>Kwa kulewa, namaanisha kujisikia kulewa au kuchangamka, au kupoteza uwezo wake wa kutambua.	MARA ZOTE . . . . .1<br>MARA CHACHE . . . . 2<br>MARA MOJA KATIKA MIEZI 12 ILIOPITA . . . . . .3<br>SIJAWAHI . . . . .4<br>SIJUI. 8		MARA ZOTE . . . . .1<br>MARA CHACHE . . . . 2<br>MARA MOJA KATIKA MIEZI 12 ILIOPITA . . . . . .3<br>SIJAWAHI . . . . .4<br>SIJUI. 8		MARA ZOTE . . . . .1<br>MARA CHACHE . . . . 2<br>MARA MOJA KATIKA MIEZI 12 ILIOPITA . . . . . . 3<br>SIJAWAHI . . . . .4<br>SIJUI. 8	
310	Huwa anakunywa na nani?<br><br>**[WEKA ALAMA YA TIKI KWA YOTE MHOJIWA ATAKAYOJIBU; USIMSOMEE]**	[ ] a. PEKE YAKE<br>[ ] b. NA MHOJIWA<br>[ ] c. NA RAFIKI WA KIKE<br>[ ] d. NA WAKWE<br>[ ] e. NA WANAFAMILIA WAKE<br>[ ] f. NA RAFIKI ZAKE WA KIKE<br>[ ] g. NA MARAFIKI ZAKE WA KIUME<br>[ ] h. NYINGINE: TAJA ___________________		[ ] a. PEKE YAKE<br>[ ] b. NA MHOJIWA<br>[ ] c. NA RAFIKI WA KIKE<br>[ ] d. NA WAKWE<br>[ ] e. NA WANAFAMILIA WAKE<br>[ ] f. NA RAFIKI ZAKE WA KIKE<br>[ ] g. NA MARAFIKI ZAKE WA KIUME<br>[ ] h. NYINGINE: TAJA ___________________		[ ] a. PEKE YAKE<br>[ ] b. NA MHOJIWA<br>[ ] c. NA RAFIKI WA KIKE<br>[ ] d. NA WAKWE<br>[ ] e. NA WANAFAMILIA WAKE<br>[ ] f. NA RAFIKI ZAKE WA KIKE<br>[ ] g. NA MARAFIKI ZAKE WA KIUME<br>[ ] h. NYINGINE: TAJA ___________________	
311	Kwa mwaka uliopita, mpenzi wako	NDIO 1		NDIO 1		NDIO 1	
		HAPANA 2	➢ 314	HAPANA 2	➢ 314	HAPANA 2	➢ 314

ID: [ ][ ][ ][ ][ ][ ][ ]

MASWALI NA CHUJAJI		a.WA SASA, HIVI PUNDE, AU MPENZI WA MSINGI		b.MPENZI MWINGINE (1)		c.MPENZI MWINGINE (2)	
	alishawahi kuvuta bangi?	SIJUI 8	➢ 314	SIJUI 8	➢ 314	SIJUI 8	➢ 314
312	Je, ni mara ngapi alivuta bangi mpaka kufikia kulevya: mara zote, mara chache tu, au hajawahi?	MARA ZOTE . . . . 1<br>MARA CHACHE . . . . 2<br>MARA MOJA KATIKA MIEZI 12. . . . . 3<br>HAJAWAHI . . . . 4<br>SIJUI. 8		MARA ZOTE . . . . 1<br>MARA CHACHE . . . . 2<br>MARA MOJA KATIKA MIEZI 12. . . . . .3<br>HAJAWAHI . . . . .4<br>SIJUI. 8		MARA ZOTE . . . . 1<br>MARA CHACHE . . . . 2<br>MARA MOJA KATIKA MIEZI 12. . . . . 3<br>HAJAWAHI . . . . .4<br>SIJUI. 8	
313	Ni nani huwa anavuta nae bangi?<br><br>**[WEKA ALAMA YA TIKI KWA YOTE MHOJIWA ATAKAYOJIBU; USIMSOMEE]**	[ ] a. PEKE YAKE<br>[ ] b. NA MHOJIWA<br>[ ] c. NA RAFIKI WA KIKE<br>[ ] d. NA WAKWE<br>[ ] e. NA WANAFAMILIA WAKE<br>[ ] f. NA RAFIKI ZAKE WA KIKE<br>[ ] g. NA MARAFIKI ZAKE WA KIUME<br>[ ] h. NYINGINE: TAJA ___________________		[ ] a. PEKE YAKE<br>[ ] b. NA MHOJIWA<br>[ ] c. NA RAFIKI WA KIKE<br>[ ] d. NA WAKWE<br>[ ] e. NA WANAFAMILIA WAKE<br>[ ] f. NA RAFIKI ZAKE WA KIKE<br>[ ] g. NA MARAFIKI ZAKE WA KIUME<br>[ ] h. NYINGINE: TAJA ___________________		[ ] a. PEKE YAKE<br>[ ] b. NA MHOJIWA<br>[ ] c. NA RAFIKI WA KIKE<br>[ ] d. NA WAKWE<br>[ ] e. NA WANAFAMILIA WAKE<br>[ ] f. NA RAFIKI ZAKE WA KIKE<br>[ ] g. NA MARAFIKI ZAKE WA KIUME<br>[ ] h. NYINGINE: TAJA ___________________	
314	Umeshawahi kufanya ngono na mpenzi huyu?	NDIO, katika miezi 12 iliyopita . . . . 1<br><br>NDIO, lakini zaidi ya miezi 12 iliyopita. . . . . 2<br>**(NENDA KIPENGELE CHA 4)**<br><br>HAPANA, sijawahi kufanya mapenzi na mpenzi huyu . . . . 3<br>(**NENDA KIPENGELE CHA 4)**		NDIO, katika miezi 12 iliyopita . . . . 1<br><br>NDIO, lakini zaidi ya miezi 12 iliyopita. . . . . 2<br>**(NENDA KIPENGELE CHA 4)**<br><br>HAPANA, sijawahi kufanya mapenzi na mpenzi huyu . . . . 3<br>(**NENDA KIPENGELE CHA 4)**		NDIO, katika miezi 12 iliyopita . . . . 1<br><br>NDIO, lakini zaidi ya miezi 12 iliyopita. . . . . 2<br>**(NENDA KIPENGELE CHA 4)**<br><br>HAPANA, sijawahi kufanya mapenzi na mpenzi huyu . . . . 3<br>(**NENDA KIPENGELE CHA 4)**	
315	Kwa kukadiria ni mara ngapi, mlifanya ngono na mtu huyu katika <u>miezi 12 iliyopita?</u><br><br>SOMA MAKUNDI	MARA KWA MARA (mara 2-3 /wiki) . . . . 1<br><br>MARA CHACHE (mara kadhaa/mwezi) . . 2<br><br>MARA MOJAMOJA . . . . . 3<br>MARA MOJA . . . . 4<br>SIJUI 8		MARA KWA MARA (mara 2-3 /wiki) . . . . 1<br><br>MARA CHACHE (mara kadhaa/mwezi) . . 2<br><br>MARA MOJAMOJA . . . . . 3<br>MARA MOJA . . . . 4<br>SIJUI 8		MARA KWA MARA (mara 2-3 /wiki) . . . . 1<br><br>MARA CHACHE (mara kadhaa/mwezi) . . 2<br><br>MARA MOJAMOJA . . . . . 3<br>MARA MOJA . . . . 4<br>SIJUI 8	
316	Mara ya mwisho kufanya ngono na huyu mpenzi	NDIO 1		NDIO 1		NDIO 1	
		HAPANA . . 2<br>SIJUI 8	(NENDA KIPENGELE CHA 4)	HAPANA . . 2<br>SIJUI 8	(NENDA KIPENGELE CHA 4)	HAPANA . . 2<br>SIJUI 8	(NENDA KIPENGELE CHA 4)

ID: [ ][ ][ ][ ][ ][ ][ ]

MASWALI NA CHUJAJI		a.WA SASA, HIVI PUNDE, AU MPENZI WA MSINGI		b.MPENZI MWINGINE (1)		c.MPENZI MWINGINE (2)	
	wako mlitumia kondomu?						
317	<u>Katika miezi 12 iliyopita</u>, mlikuwa mnatumia kondomu kila mfanyapo ngono na mtu huyu?	NDIO . . . . 1<br>HAPANA . . . . 2<br>SIJUI 8		NDIO . . . . 1<br>HAPANA . . . . 2<br>SIJUI 8		NDIO . . . . 1<br>HAPANA . . . . 2<br>SIJUI 8	

Kipengele cha 4 : Ukatili toka kwa mpenzi/mwandani (IPV) katika miezi 12

MHOJAJI:

Soma hii mara ya kwanza tu/kwa mpenzi huyu. Sasa ningependa kukuuliza maswali kuhusu hali ambazo zinawatokea baadhi ya wanawake. Nakuomba uweze kukumbuka ndani ya miezi 12 iliyopita na tafadhali nieleze kama yamewahi kukutokea. Kuna baadhi ya maswali yanaweza kuwa ya undani sana, kama tutafikia swali lolote ambalo usingependa kulijibu, naomba unitaarifu na nitaliruka na kuendelea na maswali mengine. Na pia ningependa kukumbusha kuwa, kama ukipenda muda wowote unaweza kusimamisha mahojiano.

MASWALI na CHUJAJI		a) WA SASA, HIVI PUNDE, AU MPENZI WA MSINGI	b) MPENZI MWINGINE (1)	c) MPENZI MWINGINE (2)
**MHOJAJI**:<br>Tukiendelea kuongea kuhusiana na mpenzi/mshirika tuliyekuwa tukimuongelea, tafadhali naomba unitaarifu mara ngapi kwa namna yoyote kama yafuatayo yamewahi kukutokea ndani ya mwaka mmoja ulioisha.				
➢ **Tabia ya kutawala ya Mpenzi**				
401	Katika miezi 12 iliyopita, Je mpenzi wako amekuwa na wivu au hasira pale ulipoongea na wanaume wengine?, mara zote, mara chache tu, au hajawahi?	MARA NYINGI . . . . . . 1<br>MARA CHACHE. . . . . 2<br>MARA MOJA . . . . . 3<br>HAJAWAHI. . . . . 4<br>SIJUI . . . . 8<br>HAKUNA JIBU 9	MARA NYINGI . . . . . . 1<br>MARA CHACHE. . . . . 2<br>MARA MOJA . . . . . 3<br>HAJAWAHI. . . . . 4<br>SIJUI . . . . 8<br>HAKUNA JIBU 9	MARA NYINGI . . . . . . 1<br>MARA CHACHE. . . . . 2<br>MARA MOJA . . . . . 3<br>HAJAWAHI. . . . . 4<br>SIJUI . . . . 8<br>HAKUNA JIBU 9
402	Amewahi kukulaumu kuwa wewe sio muaminifu?	MARA NYINGI . . . . . . 1<br>MARA CHACHE. . . . . 2<br>MARA MOJA . . . . . 3<br>HAJAWAHI. . . . . 4<br>SIJUI . . . . 8<br>HAKUNA JIBU 9	MARA NYINGI . . . . . . 1<br>MARA CHACHE. . . . . 2<br>MARA MOJA . . . . . 3<br>HAJAWAHI. . . . . 4<br>SIJUI . . . . 8<br>HAKUNA JIBU 9	MARA NYINGI . . . . . . 1<br>MARA CHACHE. . . . . 2<br>MARA MOJA . . . . . 3<br>HAJAWAHI. . . . . 4<br>SIJUI . . . . 8<br>HAKUNA JIBU 9
403	Amewahi kukuzuia kukutana na rafiki zako wa kike?	MARA NYINGI . . . . . . 1<br>MARA CHACHE. . . . . 2<br>MARA MOJA . . . . . 3<br>HAJAWAHI. . . . . 4<br>SIJUI . . . . 8<br>HAKUNA JIBU 9	MARA NYINGI . . . . . . 1<br>MARA CHACHE. . . . . 2<br>MARA MOJA . . . . . 3<br>HAJAWAHI. . . . . 4<br>SIJUI . . . . 8<br>HAKUNA JIBU 9	MARA NYINGI . . . . . . 1<br>MARA CHACHE. . . . . 2<br>MARA MOJA . . . . . 3<br>HAJAWAHI. . . . . 4<br>SIJUI . . . . 8<br>HAKUNA JIBU 9
404	Alijaribu kukuzuia kuwasiliana na familia yako?	MARA NYINGI . . . . . . 1<br>MARA CHACHE. . . . . 2<br>MARA MOJA . . . . . 3<br>HAJAWAHI. . . . . 4<br>SIJUI . . . . 8<br>HAKUNA JIBU 9	MARA NYINGI . . . . . . 1<br>MARA CHACHE. . . . . 2<br>MARA MOJA . . . . . 3<br>HAJAWAHI. . . . . 4<br>SIJUI . . . . 8<br>HAKUNA JIBU 9	MARA NYINGI . . . . . . 1<br>MARA CHACHE. . . . . 2<br>MARA MOJA . . . . . 3<br>HAJAWAHI. . . . . 4<br>SIJUI . . . . 8<br>HAKUNA JIBU 9

ID: [ ][ ][ ][ ][ ][ ][ ]

MASWALI na CHUJAJI		a) WA SASA, HIVI PUNDE, AU MPENZI WA MSINGI	b) MPENZI MWINGINE (1)	c) MPENZI MWINGINE (2)
405	Kwa ujumla, je alisisitiza kujua ulipo muda wote?	MARA NYINGI . . . . . . 1<br>MARA CHACHE. . . . . 2<br>MARA MOJA . . . . . 3<br>HAJAWAHI. . . . . 4<br>SIJUI . . . . 8<br>HAKUNA JIBU 9	MARA NYINGI . . . . . . 1<br>MARA CHACHE. . . . . 2<br>MARA MOJA . . . . . 3<br>HAJAWAHI. . . . . 4<br>SIJUI . . . . 8<br>HAKUNA JIBU 9	MARA NYINGI . . . . . . 1<br>MARA CHACHE. . . . . 2<br>MARA MOJA . . . . . 3<br>HAJAWAHI. . . . . 4<br>SIJUI . . . . 8<br>HAKUNA JIBU 9
406	Alijaribu kutawala matumizi yako ya pesa?	MARA NYINGI . . . . . . 1<br>MARA CHACHE. . . . . 2<br>MARA MOJA . . . . . 3<br>HAJAWAHI. . . . . 4<br>SIJUI . . . . 8<br>HAKUNA JIBU 9	MARA NYINGI . . . . . . 1<br>MARA CHACHE. . . . . 2<br>MARA MOJA . . . . . 3<br>HAJAWAHI. . . . . 4<br>SIJUI . . . . 8<br>HAKUNA JIBU 9	MARA NYINGI . . . . . . 1<br>MARA CHACHE. . . . . 2<br>MARA MOJA . . . . . 3<br>HAJAWAHI. . . . . 4<br>SIJUI . . . . 8<br>HAKUNA JIBU 9
407	Je, unaweza kusema pesa uliyopata katika miezi 12 iliyopita, ni zaidi ya pesa mpenzi wako aliyopata, ni pungufu ya aliyopata mpenzi wako, au karibu sawasawa?	ZAIDI YAKE . . . . 1<br>PUNGUFU YAKE . . . .2<br>KARIBU SAWASAWA 3<br>SIJUI. 8	ZAIDI YAKE . . . . 1<br>PUNGUFU YAKE . . . .2<br>KARIBU SAWASAWA 3<br>SIJUI. 8	ZAIDI YAKE . . . . 1<br>PUNGUFU YAKE . . . .2<br>KARIBU SAWASAWA 3<br>SIJUI. 8
➢ **Kujisikia kunyanyaswa kihisia**				
408	Katika miezi 12 iliyopita:<br>Husema au hufanya vitu vya kukudhalilisha mbele ya watu wengine?	MARA NYINGI . . . . . . 1<br>MARA CHACHE. . . . . 2<br>MARA MOJA . . . . . 3<br>HAJAWAHI. . . . . 4<br>SIJUI . . . . 8<br>HAKUNA JIBU 9	MARA NYINGI . . . . . . 1<br>MARA CHACHE. . . . . 2<br>MARA MOJA . . . . . 3<br>HAJAWAHI. . . . . 4<br>SIJUI . . . . 8<br>HAKUNA JIBU 9	MARA NYINGI . . . . . . 1<br>MARA CHACHE. . . . . 2<br>MARA MOJA . . . . . 3<br>HAJAWAHI. . . . . 4<br>SIJUI . . . . 8<br>HAKUNA JIBU 9
409	Ametishia kukuumiza au kukudhuru, au kumuumiza mtu wa jirani yako?	MARA NYINGI . . . . . . 1<br>MARA CHACHE. . . . . 2<br>MARA MOJA . . . . . 3<br>HAJAWAHI. . . . . 4<br>SIJUI . . . . 8<br>HAKUNA JIBU 9	MARA NYINGI . . . . . . 1<br>MARA CHACHE. . . . . 2<br>MARA MOJA . . . . . 3<br>HAJAWAHI. . . . . 4<br>SIJUI . . . . 8<br>HAKUNA JIBU 9	MARA NYINGI . . . . . . 1<br>MARA CHACHE. . . . . 2<br>MARA MOJA . . . . . 3<br>HAJAWAHI. . . . . 4<br>SIJUI . . . . 8<br>HAKUNA JIBU 9
410	Alikutukana au kukufanya ujisikie vibaya?	MARA NYINGI . . . . . . 1<br>MARA CHACHE. . . . . 2<br>MARA MOJA . . . . . 3<br>HAJAWAHI. . . . . 4<br>SIJUI . . . . 8<br>HAKUNA JIBU 9	MARA NYINGI . . . . . . 1<br>MARA CHACHE. . . . . 2<br>MARA MOJA . . . . . 3<br>HAJAWAHI. . . . . 4<br>SIJUI . . . . 8<br>HAKUNA JIBU 9	MARA NYINGI . . . . . . 1<br>MARA CHACHE. . . . . 2<br>MARA MOJA . . . . . 3<br>HAJAWAHI. . . . . 4<br>SIJUI . . . . 8<br>HAKUNA JIBU 9
➢ **Hali ya kujisikia kunyanyaswa kivitendo**				
411	Alishawahi kukusukuma,	MARA NYINGI . . . . . . 1<br>MARA CHACHE. . . . . 2<br>MARA MOJA 3	MARA NYINGI . . . . . . 1<br>MARA CHACHE. . . . . 2<br>MARA MOJA 3	MARA NYINGI . . . . . . 1<br>MARA CHACHE. . . . . 2<br>MARA MOJA 3

ID: [ ][ ][ ][ ][ ][ ][ ]

MASWALI na CHUJAJI		a) WA SASA, HIVI PUNDE, AU MPENZI WA MSINGI	b) MPENZI MWINGINE (1)	c) MPENZI MWINGINE (2)
	kukutikisa, au kukurushia kitu?	HAJAWAHI. . . . . 4<br>SIJUI . . . . 8<br>HAKUNA JIBU 9	HAJAWAHI. . . . . 4<br>SIJUI . . . . 8<br>HAKUNA JIBU 9	HAJAWAHI. . . . . 4<br>SIJUI . . . . 8<br>HAKUNA JIBU 9
412	Kukupiga makofi?	MARA NYINGI . . . . . . 1<br>MARA CHACHE. . . . . 2<br>MARA MOJA . . . . . 3<br>HAJAWAHI. . . . . 4<br>SIJUI . . . . 8<br>HAKUNA JIBU 9	MARA NYINGI . . . . . . 1<br>MARA CHACHE. . . . . 2<br>MARA MOJA . . . . . 3<br>HAJAWAHI. . . . . 4<br>SIJUI . . . . 8<br>HAKUNA JIBU 9	MARA NYINGI . . . . . . 1<br>MARA CHACHE. . . . . 2<br>MARA MOJA . . . . . 3<br>HAJAWAHI. . . . . 4<br>SIJUI . . . . 8<br>HAKUNA JIBU 9
413	Alikukunja mkono au kukuvuta nywele?	MARA NYINGI . . . . . . 1<br>MARA CHACHE. . . . . 2<br>MARA MOJA . . . . . 3<br>HAJAWAHI. . . . . 4<br>SIJUI . . . . 8<br>HAKUNA JIBU 9	MARA NYINGI . . . . . . 1<br>MARA CHACHE. . . . . 2<br>MARA MOJA . . . . . 3<br>HAJAWAHI. . . . . 4<br>SIJUI . . . . 8<br>HAKUNA JIBU 9	MARA NYINGI . . . . . . 1<br>MARA CHACHE. . . . . 2<br>MARA MOJA . . . . . 3<br>HAJAWAHI. . . . . 4<br>SIJUI . . . . 8<br>HAKUNA JIBU 9
414	Alikupiga ngumi au alikupiga na kitu kinachoweza kukuumiza?	MARA NYINGI . . . . . . 1<br>MARA CHACHE. . . . . 2<br>MARA MOJA . . . . . 3<br>HAJAWAHI. . . . . 4<br>SIJUI . . . . 8<br>HAKUNA JIBU 9	MARA NYINGI . . . . . . 1<br>MARA CHACHE. . . . . 2<br>MARA MOJA . . . . . 3<br>HAJAWAHI. . . . . 4<br>SIJUI . . . . 8<br>HAKUNA JIBU 9	MARA NYINGI . . . . . . 1<br>MARA CHACHE. . . . . 2<br>MARA MOJA . . . . . 3<br>HAJAWAHI. . . . . 4<br>SIJUI . . . . 8<br>HAKUNA JIBU 9
415	Alikupiga teke, alikuburuza au alikuchapa?	MARA NYINGI . . . . . . 1<br>MARA CHACHE. . . . . 2<br>MARA MOJA . . . . . 3<br>HAJAWAHI. . . . . 4<br>SIJUI . . . . 8<br>HAKUNA JIBU 9	MARA NYINGI . . . . . . 1<br>MARA CHACHE. . . . . 2<br>MARA MOJA . . . . . 3<br>HAJAWAHI. . . . . 4<br>SIJUI . . . . 8<br>HAKUNA JIBU 9	MARA NYINGI . . . . . . 1<br>MARA CHACHE. . . . . 2<br>MARA MOJA . . . . . 3<br>HAJAWAHI. . . . . 4<br>SIJUI . . . . 8<br>HAKUNA JIBU 9
416	Alikukaba au alikuunguza makusudi?	MARA NYINGI . . . . . . 1<br>MARA CHACHE. . . . . 2<br>MARA MOJA . . . . . 3<br>HAJAWAHI. . . . . 4<br>SIJUI . . . . 8<br>HAKUNA JIBU 9	MARA NYINGI . . . . . . 1<br>MARA CHACHE. . . . . 2<br>MARA MOJA . . . . . 3<br>HAJAWAHI. . . . . 4<br>SIJUI . . . . 8<br>HAKUNA JIBU 9	MARA NYINGI . . . . . . 1<br>MARA CHACHE. . . . . 2<br>MARA MOJA . . . . . 3<br>HAJAWAHI. . . . . 4<br>SIJUI . . . . 8<br>HAKUNA JIBU 9
417	Alikutishia au alikuvamia na kisu, bunduki au silaha yoyote?	MARA NYINGI . . . . . . 1<br>MARA CHACHE. . . . . 2<br>MARA MOJA . . . . . 3<br>HAJAWAHI. . . . . 4<br>SIJUI . . . . 8<br>HAKUNA JIBU 9	MARA NYINGI . . . . . . 1<br>MARA CHACHE. . . . . 2<br>MARA MOJA . . . . . 3<br>HAJAWAHI. . . . . 4<br>SIJUI . . . . 8<br>HAKUNA JIBU 9	MARA NYINGI . . . . . . 1<br>MARA CHACHE. . . . . 2<br>MARA MOJA . . . . . 3<br>HAJAWAHI. . . . . 4<br>SIJUI . . . . 8<br>HAKUNA JIBU 9
➢	**Hali ya kujisikia kunyanyasika kingono**			
418	Alikulazimisha kwa nguvu kufanya ngono	MARA NYINGI . . . . . . 1<br>MARA CHACHE. . . . . 2<br>MARA MOJA 3	MARA NYINGI . . . . . . 1<br>MARA CHACHE. . . . . 2<br>MARA MOJA 3	MARA NYINGI . . . . . . 1<br>MARA CHACHE. . . . . 2<br>MARA MOJA 3

ID: [ ][ ][ ][ ][ ][ ][ ]

MASWALI na CHUJAJI		a) WA SASA, HIVI PUNDE, AU MPENZI WA MSINGI	b) MPENZI MWINGINE (1)	c) MPENZI MWINGINE (2)
	naye hata kama ulikuwa hutaki?	HAJAWAHI. . . . . 4<br>SIJUI . . . . 8<br>HAKUNA JIBU 9	HAJAWAHI. . . . . 4<br>SIJUI . . . . 8<br>HAKUNA JIBU 9	HAJAWAHI. . . . . 4<br>SIJUI . . . . 8<br>HAKUNA JIBU 9
419	Alikulazimisha kufanya vitendo vyovyote vya kingono ambavyo hukutaka?	MARA NYINGI . . . . . . 1<br>MARA CHACHE. . . . . 2<br>MARA MOJA . . . . . 3<br>HAJAWAHI. . . . . 4<br>SIJUI . . . . 8<br>HAKUNA JIBU 9	MARA NYINGI . . . . . . 1<br>MARA CHACHE. . . . . 2<br>MARA MOJA . . . . . 3<br>HAJAWAHI. . . . . 4<br>SIJUI . . . . 8<br>HAKUNA JIBU 9	MARA NYINGI . . . . . . 1<br>MARA CHACHE. . . . . 2<br>MARA MOJA . . . . . 3<br>HAJAWAHI. . . . . 4<br>SIJUI . . . . 8<br>HAKUNA JIBU 9
➢ **Hali ya kujisikia ukatili wa zaidi wa kimaumbile**				
**MHOJAJI**:<br>Je lolote kati ya haya yafuatayo yameshawahi kukutokea ndani ya miezi 12 iliyopita kutokana na vitendo vya mpenzi wako alivyokufanyia				
420	Ulichanika, chubuka au kupata maumivu?	MARA NYINGI . . . . . . 1<br>MARA CHACHE. . . . . 2<br>MARA MOJA . . . . . 3<br>HAJAWAHI. . . . . 4<br>SIJUI . . . . 8<br>HAKUNA JIBU 9	MARA NYINGI . . . . . . 1<br>MARA CHACHE. . . . . 2<br>MARA MOJA . . . . . 3<br>HAJAWAHI. . . . . 4<br>SIJUI . . . . 8<br>HAKUNA JIBU 9	MARA NYINGI . . . . . . 1<br>MARA CHACHE. . . . . 2<br>MARA MOJA . . . . . 3<br>HAJAWAHI. . . . . 4<br>SIJUI . . . . 8<br>HAKUNA JIBU 9
421	Uliumia macho, kuteguka, kutenguka, au kuungua?	MARA NYINGI . . . . . . 1<br>MARA CHACHE. . . . . 2<br>MARA MOJA . . . . . 3<br>HAJAWAHI. . . . . 4<br>SIJUI . . . . 8<br>HAKUNA JIBU 9	MARA NYINGI . . . . . . 1<br>MARA CHACHE. . . . . 2<br>MARA MOJA . . . . . 3<br>HAJAWAHI. . . . . 4<br>SIJUI . . . . 8<br>HAKUNA JIBU 9	MARA NYINGI . . . . . . 1<br>MARA CHACHE. . . . . 2<br>MARA MOJA . . . . . 3<br>HAJAWAHI. . . . . 4<br>SIJUI . . . . 8<br>HAKUNA JIBU 9
422	Ulipata majeraha ya ndani, ulivunjika, uling'oka au kuvunjika jino, kuvunjika mifupa au majeraha yoyote makubwa?	MARA NYINGI . . . . . . 1<br>MARA CHACHE. . . . . 2<br>MARA MOJA . . . . . 3<br>HAJAWAHI. . . . . 4<br>SIJUI . . . . 8<br>HAKUNA JIBU 9	MARA NYINGI . . . . . . 1<br>MARA CHACHE. . . . . 2<br>MARA MOJA . . . . . 3<br>HAJAWAHI. . . . . 4<br>SIJUI . . . . 8<br>HAKUNA JIBU 9	MARA NYINGI . . . . . . 1<br>MARA CHACHE. . . . . 2<br>MARA MOJA . . . . . 3<br>HAJAWAHI. . . . . 4<br>SIJUI . . . . 8<br>HAKUNA JIBU 9

ID: [ ][ ][ ][ ][ ][ ][ ]

MASWALI na CHUJAJI		a) WA SASA, HIVI PUNDE, AU MPENZI WA MSINGI	b) MPENZI MWINGINE (1)	c) MPENZI MWINGINE (2)
➤ **Vichocheo vya kunyanyaswa kwa vitendo**				
423	Ndani ya miezi 12 iliyopita, Je, wewe umewahi kumpiga, kumsukuma, kumpiga teke au kufanya kitendo chochote kumuumiza mpenzi wako kipindi bado hajawahi kukupiga au kukuujeruhi?	MARA NYINGI . . . . . . 1<br>MARA CHACHE. . . . . 2<br>MARA MOJA . . . . . 3<br>HAJAWAHI. . . . . 4<br>SIJUI . . . . 8<br>HAKUNA JIBU 9	MARA NYINGI . . . . . . 1<br>MARA CHACHE. . . . . 2<br>MARA MOJA . . . . . 3<br>HAJAWAHI. . . . . 4<br>SIJUI . . . . 8<br>HAKUNA JIBU 9	MARA NYINGI . . . . . . 1<br>MARA CHACHE. . . . . 2<br>MARA MOJA . . . . . 3<br>HAJAWAHI. . . . . 4<br>SIJUI . . . . 8<br>HAKUNA JIBU 9
**CHUJAJI:** Wapenzi wengine katika miezi 12 iliyopita?				
424	Tofauti na huyu mtu (hawa watu wawili) ambaye/ambayo tumewaongelea, umefanya ngono na mtu mwingine yeyote ndani ya miezi 12 iliyopita (kama vile, mume, mpenzi unayeishi naye, mpenzi wa kuishi naye wa zamani, rafiki wa kiume, rafiki wa kiume wa zamani)?	NDIO. . . . . .1<br>RUDI SWALI Na. 301 na uliza kuanzia swali la 301 mpaka 424 kwa mpenzi wa pili; NAKILI MAJIBU KWENYE SAFU YA PILI<br><br>Hapana 2	NDIO. . . . . .1<br>RUDI SWALI Na. 301 na uliza kuanzia swali la 301 mpaka 424 kwa mpenzi wa pili; NAKILI MAJIBU KWENYE SAFU YA PILI<br><br>Hapana 2	**NENDA KIPENGELE CHA 5**

ID: [ ][ ][ ][ ][ ][ ][ ]

Kipengele cha 5: Ukatili wa kimaumbile na kingono katika miezi 12 iliyopita

MHOJAJI: Sasa ningependa kukuuliza kuhusiana na aina ya unyanyasaji ambao unaweza kuwa umeupata kutoka kwa wengine, (KAMA KIPENGELE CHA 3 NA 4 KILIULIZWA, BASI ONGEZA: tofauti na wapenzi tuliokwisha waongelea)

MASWALI NA CHUJAJI		UFUNGUO WA MAKUNDI	NENDA
➢ **Ukatili wa kimaumbile**			
501	Katika miezi 12 iliyopita, kuna mtu mwingine yoyote [tofauti na mpenzi/wapenzi tuliokwisha waongelea (mume, mpenzi wa kuishi naye, rafiki wa kiume) alikupiga ngumi, kofi, teke au alikufanyia kitu chochote ili kukudhuru?	NDIO . . . . .1<br>HAPANA . . . . .2<br>HAKUNA JIBU 9	 ➢ 503<br>➢ 503
502	Je, ni nani aliyekuumiza katika namna hiyo? USISOME MAKUNDI, LAKINI WEKEA ALAMA YA TIKI "√" KWA YOTE ATAKAYOTAJA MHOJIWA. TUMIA KUNDI MOJA TU KWA KILA MTU ATAKAYETAJWA. KISHA ULIZA KUHUSU IDADI KWA ULIOWEKEA ALAMA: Je, hii ilitokea mara nyingi, mara chace au mara moja katika miezi 12 iliyopita?		
	a. [ ] NDUGU WA KUZALIWA NAYE	MARA NYINGI . . . . 1<br>MARA CHACHE. . . . . 2<br>MARA MOJA. 3	
	b. [ ] MKWE/NDUGU WA MPENZI	MARA NYINGI . . . . 1<br>MARA CHACHE. . . . . 2<br>MARA MOJA. 3	
	c. [ ] MUME WA ZAMANI/MPENZI WA KUISHI NAYE ZAIDI YA MIEZI 12 ILIYOPITA	MARA NYINGI . . . . 1<br>MARA CHACHE. . . . . 2<br>MARA MOJA. 3	
	d. [ ] RAFIKI WA KIUME WA ZAMANI WA ZAIDI YA MIEZI 12 ILIYOPITA	MARA NYINGI . . . . 1<br>MARA CHACHE. . . . . 2<br>MARA MOJA. 3	
	e. [ ] MWALIMU	MARA NYINGI . . . . 1<br>MARA CHACHE. . . . . 2<br>MARA MOJA. 3	
	f. [ ] MWAJIRI/MTU KAZINI	MARA NYINGI . . . . 1<br>MARA CHACHE. . . . . 2<br>MARA MOJA. 3	
	g. [ ] ASKARI/MWANAJESHI	MARA NYINGI . . . . 1<br>MARA CHACHE. . . . . 2<br>MARA MOJA. 3	
	h. [ ] JIRANI/MKAZI WA MTAANI	MARA NYINGI . . . . 1<br>MARA CHACHE. . . . . 2<br>MARA MOJA. 3	
	i. [ ] MTU NISIYE MFAHAMU	MARA NYINGI . . . . 1<br>MARA CHACHE. . . . . 2<br>MARA MOJA. 3	

ID: [ ][ ][ ][ ][ ][ ][ ]

MASWALI NA CHUJAJI		UFUNGUO WA MAKUNDI	NENDA
	j. [ ] MWINGINE, TAJA_____________	MARA NYINGI . . . . 1<br>MARA CHACHE. . . . . 2<br>MARA MOJA. 3	
➢ **Kulazimishwa kufanya ngono**			
503	Katika miezi 12 iliyopita, kuna mtu mwingine yoyote [tofauti na mpenzi/wapenzi tuliokwisha waongelea (mume, mpenzi wa kuishi naye, rafiki wa kiume) amekulazimisha kufanya ngono bila wewe kuridhia?	NDIO . . . . .1<br>HAPANA . . . . .2<br>HAKUNA JIBU 9	 ➢505<br>➢505
504	Je, ni nani aliyekulazimisha kufanya naye ngono? USISOME MAKUNDI, LAKINI WEKEA ALAMA YA TIKI “√” ATAKAYOTAJA MHOJIWA; TUMIA KUNDI MOJA TU KWA KILA MTU ATAKAYETAJWA<br><br>KISHA ULIZA KUHUSU IDADI KWA ULIOWEKEA ALAMA:<br>Je, hii ilitokea mara nyingi, mara chace au mara moja katika miezi 12 iliyopita?		
	a. [ ] BABA	MARA NYINGI . . . . 1<br>MARA CHACHE. . . . . 2<br>MARA MOJA. 3	
	b. [ ] NDUGU MWINGINE WA KUZALIWA NAYE	MARA NYINGI . . . . 1<br>MARA CHACHE. . . . . 2<br>MARA MOJA. 3	
	c. [ ] BABA WA KAMBO	MARA NYINGI . . . . 1<br>MARA CHACHE. . . . . 2<br>MARA MOJA. 3	
	d. [ ] MKWE	MARA NYINGI . . . . 1<br>MARA CHACHE. . . . . 2<br>MARA MOJA. 3	
	e. [ ] MUME WA ZAMANI/MPENZI WA KUISHI NAYE ZAIDI YA MIEZI 12 ILIYOPITA	MARA NYINGI . . . . 1<br>MARA CHACHE. . . . . 2<br>MARA MOJA. 3	
	f. [ ] RAFIKI YANGU/MSHIRIKA	MARA NYINGI . . . . 1<br>MARA CHACHE. . . . . 2<br>MARA MOJA. 3	
	g. [ ] RAFIKI WA FAMILIA AU MWANAFAMILIA	MARA NYINGI . . . . 1<br>MARA CHACHE. . . . . 2<br>MARA MOJA. 3	
	h. [ ] MWALIMU	MARA NYINGI . . . . 1<br>MARA CHACHE. . . . . 2<br>MARA MOJA. 3	
	i. [ ] MWAJIRI/MTU KAZINI	MARA NYINGI . . . . 1<br>MARA CHACHE. . . . . 2<br>MARA MOJA. 3	
	j. [ ] ASKARI/MWANAJESHI	MARA NYINGI 1	

ID: [ ][ ][ ][ ][ ][ ][ ]

MASWALI NA CHUJAJI		UFUNGUO WA MAKUNDI	NENDA
		MARA CHACHE. . . . . 2<br>MARA MOJA. 3	
	k. [ ] PADRI/MCHUNGAJI/KIONGOZI WA DINI	MARA NYINGI . . . . 1<br>MARA CHACHE. . . . . 2<br>MARA MOJA. 3	
	l. [ ] JIRANI/MKAZI WA MTAANI	MARA NYINGI . . . . 1<br>MARA CHACHE. . . . . 2<br>MARA MOJA. 3	
	m. [ ] MTU NISIYE MFAHAMU	MARA NYINGI . . . . 1<br>MARA CHACHE. . . . . 2<br>MARA MOJA. 3	
	n. [ ] MWINGINE, TAJA_____________	MARA NYINGI . . . . 1<br>MARA CHACHE. . . . . 2<br>MARA MOJA. 3	
505	Kwa ambavyo utaweza kukumbuka, je baba yako aliwahi kumpiga mama yako?	NDIO . . . . 1<br>HAPANA . . . . .2<br>SIJUI . . . . 8<br>HANA JIBU 9	

**UFUPISHO**<br>**Maelekezo kwa mhojaji: Tazama tena majibu ya maswali 411-417 na 501 na nakili taarifa zifuatazo.**		
**506**	**Kukutwa na walau aina moja ya ukatili wa kimaumbile katika miezi 12 iliyopita?**	**NDIO** . . . . **1**<br>Kama LOLOTE kati ya masharti yafuatayo yamefuatwa:<br>Swali na. 411 = 1, 2 or 3<br>Swali na. 412=1, 2 or 3<br>Swali na. 413=1, 2 or 3<br>Swali na. 414=1, 2 or 3<br>Swali na. 415=1, 2 or 3<br>Swali na. 416=1, 2 or 3<br>Swali na. 417=1, 2 or 3<br>Swali na. 501=1<br><br>**HAPANA/SIJUI/HAKUNA JIBU** . . . . **2**<br>Kama HAKUNA sharti kati ya hayo hapo juu lililofuatwa
**507**	**Kukutwa na walau aina moja ya unyanyasaji wa kingono katika miezi 12 iliyopita?**	**NDIO** . . . . **1**<br>Kama LOLOTE kati ya masharti yafuatayo yamefuatwa:<br>Swali na. 418 = 1, 2 or 3<br>Swali na. 419=1, 2 or 3<br>Swali na. 503=1<br><br>**HAPANA/SIJUI/HAKUNA JIBU**. . . . . **2**<br>Kama HAKUNA sharti kati ya hayo hapo juu lililofuatwa
**CHUJAJI**		
**Kama Swali na. 506 =1 AU Swali na. 507=1 endelea na KIPENGELE CHA 6**		

ID: [ ][ ][ ][ ][ ][ ][ ]

Kama Swali na. 506=2 NA Swali na. 507=2 basi NENDA KIPENGELE CHA 7

Kipengele cha 6: Matumizi ya Huduma za IPV katika miezi 12 iliyopita

MHOJAJI:
Umemaliza kunishirikisha kuwa umewahi kupatwa na matukio au hali ambazo ningependa kuzitambua kama "ukatili wa kijinsia". Ningependa kukuuliza kuhusiana na hatua zozote ulizochukua kutokana na matukio hayo.

MASWALI na CHUJAJI		UFUNGUO WA MAKUNDI	NENDA
601	Ndani ya miezi 12 iliyopita, je umewahi kutafuta msaada ili kuzuia ukatili wa kijinsia huo uliopitia –au kutafuta matibabu kwa ajili ya matokeo ya matukio hayo?	NDIO, NILITAFUTA MSAADA. . . . . .1<br>HAPANA, SIJATAFUTA MSAADA 2	 ➢ **Kipengele cha 7**
602	Je, wapi na nani ulimwendea kwa ajili ya msaada, katika miezi 12 iliyopita?<br><br>**USIMSOMEE; WEKA ALAMA YA TIKI KWA YOTE ATAKAYOTAJA**	a. [ ] MWANAFAMILIA MWENZANGU<br>b. [ ] WAKWE<br>c. [ ] MUME/MPENZI<br>d. [ ] MPENZI WA ZAMANI<br>e. [ ] RAFIKI WA KIUME/KIKE<br>f. [ ] RAFIKI<br>g. [ ] JIRANI<br>h. [ ] KIONGOZI WA DINI<br>i. [ ] KIONGOZI WA SERIKALI<br>j. [ ] ASKARI<br>k. [ ] MWANASHERIA/HUDUMA ZA SHERIA<br>l. [ ] ASASI YA KIRAIA/KIDINI<br>m. [ ] OFISI YA USTAWI<br>n. [ ] VIONGOZI WA SHULE<br>o. [ ] MAKAZI SALAMA<br>p. [ ] KITUO CHA AFYA<br>q. [ ] NYINGINE: TAJA<br>____________________	
603	Wapi/Nani ulimwendea kwa msaada wa kwanza?<br><br>**NAKILI HERUFI MOJA (a. – q.) YA MAJIBU HAPO JUU TOKA SWALI LA 602**	NILIENDA KWANZA . . . . [ ]	
604	Ndani ya miezi 12 iliyopita, umewahi kutembelea kituo cha afya kwa ajili ya kupata huduma, matibabu au kupata msaada kutokana na ukatili wa kijinsia ulioupata?	IDADI YA MAHUDHURIO . . . . . [ ] [ ] [ ]<br><br>**KAMA NI ZAIDI YA 1, ANZA KWA KUULIZA SWALI LA 605, KUHUSU HUDHURIO LA JIRANI ZAIDI NA ENDELEA KUULIZA MPAKA MAHUDHURIO 3**	**Kama "0," ruka mpaka kipengele cha 7**

ID: [ ][ ][ ][ ][ ][ ][ ]

MASWALI na CHUJAJI		a.<br>HUDHURIO LA KITUO CHA AFYA LA JIRANI ZAIDI	b.<br>HUDHURIO LA KITUO CHA AFYA LA PILI KWA UJIRANI ZAIDI	c.<br>HUDHURIO LA AFYA LINALOFUATIA KWA UJIRANI ZAIDI
**MHOJAJI:**<br>Sasa naelekea kukuuliza maswali kuhusiana na hayo mahudhurio kwa kuanzia na yale ya karibu kabisa.				
605	Je, kituo ulichoenda ni zahanati, kituo cha afya au hospitali.	Zahanati . . . . 1<br>Kituo cha afya . . . . . . .2<br>Hospitali . . . . 3<br>Nyingine . . . . .4<br>Taja:________________	Zahanati . . . . 1<br>Kituo cha afya . . . . . . 2<br>Hospitali . . . . 3<br>Nyingine . . . . .4<br>Taja: ________________	Zahanati . . . . 1<br>Kituo cha afya . . . . .2<br>Hospitali . . . . 3<br>Nyingine . . . . .4<br>Taja: _______________
606	Je, kituo kilikuwa kinaitwaje?	2) Jina la kituo:<br>____________________	2) Jina la kituo:<br>____________________	2) Jina la kituo:<br>_____________________
607	CHUJAJI:<br>USIULIZE HILI SWALI KWA SAFU a. ULIZA TU KAMA INAFAA KWA SAFU b and c.<br>Je mahudhurio haya ilikuwa ni sababu ya matukio ya unyanyasaji kama mahudhurio yako megine katika kituo cha afya	X	NDIO, TUKIO LILE LILE . . . . 1<br>HAPANA, TUKIO TOFAUTI 2	NDIO, TUKIO LILE LILE . . . . 1<br>HAPANA, TUKIO TOFAUTI. . . . 2

ID: [ ][ ][ ][ ][ ][ ][ ]

MASWALI na CHUJAJI		a.<br>HUDHURIO LA KITUO CHA AFYA LA JIRANI ZAIDI	b.<br>HUDHURIO LA KITUO CHA AFYA LA PILI KWA UJIRANI ZAIDI	c.<br>HUDHURIO LA AFYA LINALOFUATIA KWA UJIRANI ZAIDI
608	Ni aina gani ya huduma ulizofuata<br><br>**[USISOME, WEKA ALAMA YA TIKI KWA YOTE MHOJIWA ATAKAYOTAJA]**	a. [ ] MATIBABU YA MAJERAHA YA KIMWILI<br>b. [ ] MATIBABU YA KUBAKWA (AU UKATILI WA KINGONO)<br>c. [ ] MSAADA WA KISAIKOLOJIA NA KIJAMII<br>d. [ ] VIPIMO VYA MAGONJWA YA ZINAA<br>e. [ ] MATIBABU YA MAGONJWA YA ZINAA<br>f. [ ] KIPIMO CHA MIMBA<br>g. [ ] UZAZI WA MPANGO<br>h. [ ] KINGA YADHARURA YA MIMBA<br>i. [ ] KIPIMO CHA UKIMWI<br>j. [ ] KINGA YA KUZUIA MAAMBUKIZI YA VVU (PEP)<br>k. [ ]UZINGATIAJI WA PEP<br>l. [ ] VIPIMO/HUDUMA ZA KIMAHAKAMA<br>m. [ ] RUFAA KWENDA KITUO KINGINE<br>n. [ ] NYINGINE, TAJA: ________________	a. [ ] MATIBABU YA MAJERAHA YA KIMWILI<br>b. [ ] MATIBABU YA KUBAKWA (AU UKATILI WA KINGONO)<br>c. [ ] MSAADA WA KISAIKOLOJIA NA KIJAMII<br>d. [ ] VIPIMO VYA MAGONJWA YA ZINAA<br>e. [ ] MATIBABU YA MAGONJWA YA ZINAA<br>f. [ ] KIPIMO CHA MIMBA<br>g. [ ] UZAZI WA MPANGO<br>h. [ ] KINGA YADHARURA YA MIMBA<br>i. [ ] KIPIMO CHA UKIMWI<br>j. [ ] KINGA YA KUZUIA MAAMBUKIZI YA VVU (PEP)<br>k. [ ]UZINGATIAJI WA PEP<br>l. [ ] VIPIMO/HUDUMA ZA KIMAHAKAMA<br>m. [ ] RUFAA KWENDA KITUO KINGINE<br>n. [ ] NYINGINE, TAJA: ________________	a. [ ] MATIBABU YA MAJERAHA YA KIMWILI<br>b. [ ] MATIBABU YA KUBAKWA (AU UKATILI WA KINGONO)<br>c. [ ] MSAADA WA KISAIKOLOJIA NA KIJAMII<br>d. [ ] VIPIMO VYA MAGONJWA YA ZINAA<br>e. [ ] MATIBABU YA MAGONJWA YA ZINAA<br>f. [ ] KIPIMO CHA MIMBA<br>g. [ ] UZAZI WA MPANGO<br>h. [ ] KINGA YADHARURA YA MIMBA<br>i. [ ] KIPIMO CHA UKIMWI<br>j. [ ] KINGA YA KUZUIA MAAMBUKIZI YA VVU (PEP)<br>k. [ ]UZINGATIAJI WA PEP<br>l. [ ] VIPIMO/HUDUMA ZA KIMAHAKAMA<br>m. [ ] RUFAA KWENDA KITUO KINGINE<br>n. [ ] NYINGINE, TAJA: ________________
609	<u>CHUJAJI: JE ULITIKI MATIBABU YA KUBAKWA (UKATILI WA KINGONO) kwenye swali la 608</u>	NDIO . . . . 1<br>HAPANA . . . . 2<br>(nenda swali la 611)	NDIO . . . . .1<br>HAPANA . . . . .2<br>(nenda swali la 611)	NDIO . . . . 1<br>HAPANA . . . . 2<br>(nenda swali la 611)

ID: [ ][ ][ ][ ][ ][ ][ ]

**MASWALI na CHUJAJI**		**a.**<br>**HUDHURIO LA KITUO CHA AFYA LA JIRANI ZAIDI**	**b.**<br>**HUDHURIO LA KITUO CHA AFYA LA PILI KWA UJIRANI ZAIDI**	**c.**<br>**HUDHURIO LA AFYA LINALOFUATIA KWA UJIRANI ZAIDI**
610	Je ulienda kwenye kituo cha afya ndani ya masaa 72 baada ya kubakwa, (ukatili wa ngono)?	NDIO . . . . 1<br>HAPANA . . . . 2<br>SIJUI 8	NDIO . . . . .1<br>HAPANA . . . . .2<br>SIJUI 8	NDIO . . . . 1<br>HAPANA . . . . 2<br>SIJUI 8

ID: [ ] [ ] [ ] [ ] [ ] [ ]

611	Kwa nini ulichagua au ulichaguaje kwenda katika kituo niki?<br><br>**[USISOME, WEKA ALAMA YA TIKI KWA YOTE MHOJIWA ATAKAYOTAJA]**<br><br>**OMBA UFAFANUZI: Kama alipata rufaa, ni nani aliyekupa hiyo rufaa?**	a. [ ] NILIKUWA NAFAHAMU PANATOLEWA HUDUMA HIZO KWENYE HICHO KITUO<br>b. [ ] KITUO KIPO JIRANI NA NINAPO ISHI<br>c. [ ] NI MAHALI NILIPO KWENDA KWA HUDUMA NYINGINE ZA AFYA<br>d. [ ] NINAWEZA KUPAFIKIA BILA WATU WENGINE KUFAHAMU<br>e. [ ] NILIPATA RUFAA HAPA AU NILIPELEKWA NA POLISI<br>f. [ ] NILIPATA RUFAA KUTOKA KWA VIONGOZI WA SERIKALI ZA MITAA<br>g. [ ]NILIPEWA RUFAA KUTOKA HOSPITALI NYINGNE (TAJA____________)<br>h. [ ]NILIELEKEZWA NA ASASI YA KIRAIA<br>(TAJA____________)<br>i. [ ]NILIELEKEZWA NA NDUGU NDUGU YANGU<br>j. [ ] NILIELEKEZWA HAPA NA SHULE<br>k. [ ] NILIELEKEZWA NA AFISA USTAWI<br>l. [ ]NILIELEKEZWA NA RAFIKI/JIRANI<br>m. [ ]NDIO UAMUZI PEKEE NILIOKUWA NAO<br>n. [ ]NYINGINE<br>(TAJA_____________)	a. [ ]NILIKUWA NAFAHAMU PANATOLEWA HUDUMA HIZO KWENYE HICHO KITUO<br>b. [ ]KITUO KIPO JIRANI NA NINAPO ISHI<br>c. [ ]NI MAHALI NILIPO KWENDA KWA HUDUMA NYINGINE ZA AFYA<br>d. [ ] NINAWEZA KUPAFIKIA BILA WATU WENGINE KUFAHAMU<br>e. [ ]NILIPATA RUFAA HAPA AU NILIPELEKWA NA POLISI<br>f. [ ]NILIPATA RUFAA KUTOKA KWA VIONGOZI WA SERIKALI ZA MITAA<br>g. [ ]NILIPEWA RUFAA KUTOKA HOSPITALI NYINGNE (TAJA___________)<br>h. [ ]NILIELEKEZWA NA ASASI YA KIRAIA (TAJA___________)<br>i. [ ]NILIELEKEZWA NA NDUGU NDUGU YANGU<br>j. [ ]NILIELEKEZWA HAPA NA SHULE<br>k. [ ]NILIELEKEZWA NA AFISA USTAWI<br>l. [ ]NILIELEKEZWA NA RAFIKI/JIRANI<br>m. [ ]NDIO UAMUZI PEKEE NILIOKUWA NAO<br>n. [ ] NYINGINE (TAJA___________)	a. [ ] NILIKUWA NAFAHAMU PANATOLEWA HUDUMA HIZO KWENYE HICHO KITUO<br>b. [ ]KITUO KIPO JIRANI NA NINAPO ISHI<br>c. [ ]NI MAHALI NILIPO KWENDA KWA HUDUMA NYINGINE ZA AFYA<br>d. [ ]NINAWEZA KUPAFIKIA BILA WATU WENGINE KUFAHAMU<br>e. [ ]NILIPATA RUFAA HAPA AU NILIPELEKWA NA POLISI<br>f. [ ] NILIPATA RUFAA KUTOKA KWA VIONGOZI WA SERIKALI ZA MITAA<br>g. [ ]NILIPEWA RUFAA KUTOKA HOSPITALI NYINGNE<br>(TAJA_____________)<br>h. [ ]NILIELEKEZWA NA ASASI YA KIRAIA (TAJA____________)<br>i. [ ]NILIELEKEZWA NA NDUGU NDUGU YANGU<br>j. [ ]NILIELEKEZWA HAPA NA SHULE<br>k. [ ]NILIELEKEZWA NA AFISA USTAWI<br>l. [ ]NILIELEKEZWA NA RAFIKI/JIRANI<br>m. [ ]NDIO UAMUZI PEKEE NILIOKUWA NAO<br>n. [ ]NYINGINE<br>(TAJA_____________)

ID: [ ][ ][ ][ ][ ][ ][ ]

MASWALI na CHUJAJI		a. HUDHURIO LA KITUO CHA AFYA LA JIRANI ZAIDI			b. HUDHURIO LA KITUO CHA AFYA LA PILI KWA UJIRANI ZAIDI			c. HUDHURIO LA AFYA LINALOFUATIA KWA UJIRANI ZAIDI		
612	Ni huduma gani ulipata katika kituo hiki? SOMA KILA HUDUMA NA NAKILI MAJIBU									
	**Huduma**	**NDIO**	**HPN**	**SJUI**	**NDIO**	**HPN**	**SIJUI**	**NDIO**	**HPN**	**SIJUI**
	1. Kupimwa na kupata ushauri wa ukatili wa kijinsia	1	2	8	1	2	8	1	2	8
	2. Vipimo vya mwili	1	2	8	1	2	8	1	2	8
	3. Vipimo vya afya ya akili	1	2	8	1	2	8	1	2	8
	4. Matibabu ya majeraha	1	2	8	1	2	8	1	2	8
	5. Vipimo vya kimahakama	1	2	8	1	2	8	1	2	8
	6. Vipimo vya kimahakama vilichukuliwa	1	2	8	1	2	8	1	2	8
	7. Kujaza fomu ya polisi ya PF3	1	2	8	1	2	8	1	2	8
	8. Ushauri wa kisaikolojia	1	2	8	1	2	8	1	2	8
	9. Ushauri wa afya ya uzazi	1	2	8	1	2	8	1	2	8
	10. Ushauri kuhusu VVU	1	2	8	1	2	8	1	2	8
	11. Ushauri wa uzingatiaji wa PEP	1	2	8	1	2	8	1	2	8
	12. Kipimo cha mimba	1	2	8	1	2	8	1	2	8
	13. Kipimo cha VVU	1	2	8	1	2	8	1	2	8
	14. Kipimo cha magonjwa ya zinaa	1	2	8	1	2	8	1	2	8
	15. Njia za uzazi wa mpango	1	2	8	1	2	8	1	2	8
	16. Kukinga mimba kwa dharura	1	2	8	1	2	8	1	2	8
	17. Matibabu ya magonjwa ya zinaa	1	2	8	1	2	8	1	2	8
	18. PEP kwa ajili ya VVU	1	2	8	1	2	8	1	2	8
	19. Kinga ya pepopunda	1	2	8	1	2	8	1	2	8

ID: [ ][ ][ ][ ][ ][ ][ ]

MASWALI na CHUJAJI		a. HUDHURIO LA KITUO CHA AFYA LA JIRANI ZAIDI	b. HUDHURIO LA KITUO CHA AFYA LA PILI KWA UJIRANI ZAIDI	c. HUDHURIO LA AFYA LINALOFUATIA KWA UJIRANI ZAIDI
613	Kwa ujumla, unaweza kuongeleaje huduma uliyopata? Nzuri sana, nzuri, wastani, mbaya, mbaya sana?	NZURI SANA. . . . . . . 1<br>NZURI. . . . . .2<br>WASTANI . . . . 3<br>MBAYA . . . . 4<br>MBAYA SANA. 5	NZURI SANA. . . . . 1<br>NZURI. . . . . .2<br>WASTANI . . . . 3<br>MBAYA . . . . 4<br>MBAYA SANA. 5	NZURI SANA. . . . . . 1<br>NZURI . . . . 2<br>WASTANI . . . . .3<br>MBAYA . . . . 4<br>MBAYA SANA 5
614	Katika matembezi haya, je ulipewa rufaa kwa ajili ya huduma nyingine <u>nje ya kituo hiki?</u>	NDIO . . . . .1<br>HAPANA . . . . 2<br>(NENDA **CHUJAJI Na.2)**	NDIO . . . . .1<br>HAPANA . . . . 2<br>(NENDA **CHUJAJI Na.2)**	NDIO . . . . .1<br>HAPANA . . . . .2<br>(NENDA **CHUJAJI Na.2)**
615	Huduma gani ulizoelekezwa? Je ulifuatilia hizi rufaa na kwenda kwenye huduma hizo ulizoelekezwa? SOMA KILA HUDUMA NA NAKILI JIBU			
	a. Uangalizi wa kisaikolojia	NDIO, NILIELEKEZWA NA NIKAENDA . . . . . .1<br>NDIO NILIELEKEZWA, ILA SIKWENDA . . . . . . 2<br>SIKUELEKEZWA 3	NDIO, NILIELEKEZWA NA NIKAENDA . . . . . .1<br>NDIO NILIELEKEZWA, ILA SIKWENDA . . . . . . 2<br>SIKUELEKEZWA 3	NDIO, NILIELEKEZWA NA NIKAENDA . . . . .1<br>NDIO NILIELEKEZWA, ILA SIKWENDA . . . . 2<br>SIKUELEKEZWA . . . 3
	b. Polisi	NDIO, NILIELEKEZWA NA NIKAENDA . . . . .1<br>NDIO NILIELEKEZWA, ILA SIKWENDA . . . . 2<br>SIKUELEKEZWA . . . 3	NDIO, NILIELEKEZWA NA NIKAENDA . . . . .1<br>NDIO NILIELEKEZWA, ILA SIKWENDA . . . . 2<br>SIKUELEKEZWA . . . 3	NDIO, NILIELEKEZWA NA NIKAENDA . . . . .1<br>NDIO NILIELEKEZWA, ILA SIKWENDA . . . . 2<br>SIKUELEKEZWA . . . 3
	c. Makazi salama	NDIO, NILIELEKEZWA NA NIKAENDA . . . . .1<br>NDIO NILIELEKEZWA, ILA SIKWENDA . . . . 2<br>SIKUELEKEZWA . . . 3	NDIO, NILIELEKEZWA NA NIKAENDA . . . . .1<br>NDIO NILIELEKEZWA, ILA SIKWENDA . . . . 2<br>SIKUELEKEZWA . . . 3	NDIO, NILIELEKEZWA NA NIKAENDA . . . . .1<br>NDIO NILIELEKEZWA, ILA SIKWENDA . . . . 2<br>SIKUELEKEZWA . . . 3
	d. Matibabu katika kituo cha afya cha juu zaidi	NDIO, NILIELEKEZWA NA NIKAENDA . . . . .1<br>NDIO NILIELEKEZWA, ILA SIKWENDA . . . . 2<br>SIKUELEKEZWA . . . 3	NDIO, NILIELEKEZWA NA NIKAENDA . . . . .1<br>NDIO NILIELEKEZWA, ILA SIKWENDA . . . . 2<br>SIKUELEKEZWA . . . 3	NDIO, NILIELEKEZWA NA NIKAENDA . . . . .1<br>NDIO NILIELEKEZWA, ILA SIKWENDA . . . . 2<br>SIKUELEKEZWA . . . 3
	e. Huduma za kisheria	NDIO, NILIELEKEZWA NA NIKAENDA . . . . .1<br>NDIO NILIELEKEZWA, ILA SIKWENDA . . . . 2<br>SIKUELEKEZWA . . . 3	NDIO, NILIELEKEZWA NA NIKAENDA . . . . .1<br>NDIO NILIELEKEZWA, ILA SIKWENDA . . . . 2<br>SIKUELEKEZWA . . . 3	NDIO, NILIELEKEZWA NA NIKAENDA . . . . .1<br>NDIO NILIELEKEZWA, ILA SIKWENDA . . . . 2<br>SIKUELEKEZWA . . . 3
	f. Nyingine1: TAJA _______________	NDIO, NILIELEKEZWA NA NIKAENDA . . . . .1<br>NDIO NILIELEKEZWA, ILA SIKWENDA . . . . 2<br>SIKUELEKEZWA . . . 3	NDIO, NILIELEKEZWA NA NIKAENDA . . . . .1<br>NDIO NILIELEKEZWA, ILA SIKWENDA . . . . 2<br>SIKUELEKEZWA . . . 3	NDIO, NILIELEKEZWA NA NIKAENDA . . . . .1<br>NDIO NILIELEKEZWA, ILA SIKWENDA . . . . 2<br>SIKUELEKEZWA . . . 3

ID: [ ][ ][ ][ ][ ][ ][ ]

MASWALI na CHUJAJI		a. HUDHURIO LA KITUO CHA AFYA LA JIRANI ZAIDI	b. HUDHURIO LA KITUO CHA AFYA LA PILI KWA UJIRANI ZAIDI	c. HUDHURIO LA AFYA LINALOFUATIA KWA UJIRANI ZAIDI
	g. Nyingine2: TAJA _______________	NDIO, NILIELEKEZWA NA NIKAENDA . . . . .1<br>NDIO NILIELEKEZWA, ILA SIKWENDA . . . . 2<br>SIKUELEKEZWA . . . 3	NDIO, NILIELEKEZWA NA NIKAENDA . . . . .1<br>NDIO NILIELEKEZWA, ILA SIKWENDA . . . . 2<br>SIKUELEKEZWA . . . 3	NDIO, NILIELEKEZWA NA NIKAENDA . . . . .1<br>NDIO NILIELEKEZWA, ILA SIKWENDA . . . . 2<br>SIKUELEKEZWA . . . 3

**MASWALI na CHUJAJI**		**a. HUDHURIO LA KITUO CHA AFYA LA JIRANI ZAIDI**	**b. HUDHURIO LA KITUO CHA AFYA LA PILI KWA UJIRANI ZAIDI**	**c. HUDHURIO LA AFYA LINALOFUATIA KWA UJIRANI ZAIDI**
**CHUJAJI 1**: Kama jibu lolote la Q615 ni 2 (NILIELEKEZWA LAKINI SIKWENDA), hivyo uliza Q616. Vinginevyo nenda **CHUJAJI Na 2**.				
616	Je, ni sababu gani zilifanya usiende kwenye huduma hizi?<br><br>**[USISOME, WEKA ALAMA YA TIKI KWA YOTE MHOJIWA ATAKAYOTAJA]**	a. [ ]SIKUZIHITAJI HIZO HUDUMA<br>b. [ ] ZIKUWEZA KUMUDU<br>c. [ ]SIKUWA NA/ SIKUWEZA KUMUDU USAFIRI<br>d. [ ]SIKUWA NA MUDA<br>e. [ ]NILIKUWA NA KAZI<br>f. [ ] SIKUWA NA WA KUNISAIDIA WATOTO<br>g. [ ]NILIKUWA NA MAJUKUMU YA FAMILIA/KAYA<br>h. [ ]MUME/MPENZI ASINGERUHUSU<br>i. [ ]SIKUJUA PA KWENDA<br>j. [ ]NILIOGOPA KUONEKANA WENGINE KUGUNDUA<br>k. [ ]NYINGINE<br>TAJA:___________	a. [ ]SIKUZIHITAJI HIZO HUDUMA<br>b. [ ]ZIKUWEZA KUMUDU<br>c. [ ]SIKUWA/ SIKUWEZA KUMUDU USAFIRI<br>d. [ ]SIKUWA NA MUDA<br>e. [ ]NILIKUWA NA KAZI<br>f. [ ]SIKUWA NA WA KUNISAIDIA WATOTO<br>g. [ ]NILIKUWA NA MAJUKUMU YA FAMILIA/KAYA<br>h. [ ]MUME/MPENZI ASINGERUHUSU<br>i. [ ]SIKUJUA PA KWENDA<br>j. [ ]NILIOGOPA KUONEKANA/ WENGINE KUGUNDUA<br>k. [ ] NYINGINE<br>TAJA:____________	a. [ ]SIKUZIHITAJI HIZO HUDUMA<br>b. [ ]ZIKUWEZA KUMUDU<br>c. [ ]SIKUWA/ SIKUWEZA KUMUDU USAFIRI<br>d. [ ]SIKUWA NA MUDA<br>e. [ ]NILIKUWA NA KAZI<br>f. [ ]SIKUWA NA WA KUNISAIDIA WATOTO<br>g. [ ] NILIKUWA NA MAJUKUMU YA FAMILIA/KAYA<br>h. [ ]MUME/MPENZI ASINGERUHUSU<br>i. [ ]SIKUJUA PA KWENDA<br>j. [ ]NILIOGOPA KUONEKANA/ WENGINE KUGUNDUA<br>k. [ ]NYINGINE<br>TAJA:___________
**CHUJAJI Na 2:**		Kama swali **604>1**, basi endelea na safu b; vinginevyo NENDA KIPENGELE CHA 7	Kama swali **604>2**, basi endelea na safu c; vinginevyo NENDA KIPENGELE CHA 7	**NENDA KIPENGELE CHA 7**

ID: [ ][ ][ ][ ][ ][ ][ ]

Kipengele cha 7: Ushiriki katika miradi ya ukatili wa jinsia katika jamii

MHOJAJI: Sasa ningependa kukuuliza kuhusu ushiriki wako katika shughuli za kijamii.

MASWALI na CHUJAJI		UFUNGUO WA MAKUNDI	NENDA
➢ Ushiriki katika shughuli za kijamii za kila aina			
701a	Katika kipindi cha miaka 2 iliyopita Je, uliwahi kuhudhuria au kushiriki shughuli za makundi, asasi au jumuiya za kijamii?<br><br>KAMA JIBU HAPANA AU SIJUI, DADISI: Hii inahusisha Jumuiya kama za wanawake, au jumuiya za kijamii, jumuiya za kidini au jumuiya za kisiasa.	NDIO . . . . 1<br>HAPANA . . . . 2<br>SIJUI 8	 ➢ 702a<br>➢ 702a
701b	Je, ni aina gani ya shughuli za kijamii, asasi au jumuiya za kijamii ulizoshiriki?<br><br>**USIMSOMEE; WEKA ALAMA YA TIKI KWA YOTE ATAKAYOTAJA**	a. [ ] kikundi cha michezo<br>b. [ ] kikundi cha muziki na kucheza<br>c. [ ] kikundi cha wanawake<br>d. [ ] kikundi cha mazishi<br>e. [ ] kikundi cha dini<br>f. [ ] kikundi cha siasa<br>g. [ ] kikundi cha huduma ya VVU<br>h. [ ] kikundi cha kuelimisha rika<br>i. [ ] Kikundi cha vijana<br>j. [ ] Kikindi cha Afya ya Jamii<br>k. [ ] Asasi ya wazazi na waalimu<br>l. [ ] Kikundi cha ushonaji<br>m. [ ] Kamati ya Maendeleo (mfano; ardhi, kamati ya maji na usafi)<br>n. [ ] Nyingine, Taja ______________________	
➢ Uelewa na ushiriki katika program za jumuiya za HJFMRI/WRP GBV			
702a	Je umewahi kusikia programu inayoitwa WRP/HJFMRI – programu ya ukatili wa kijinsia pia inajulikana kama programu ya GBV ya HJFMRI au AMKA SASA?	NDIO . . . . 1<br>HAPANA 2	 ➢ 703a
702b	Je, ulishiriki katika uanzishwaji wa programu hii?	NDIO . . . . 1<br>HAPANA 2	

ID: [ ][ ][ ][ ][ ][ ][ ]

MASWALI na CHUJAJI		UFUNGUO WA MAKUNDI	NENDA
703a	Je umewahi kusikia, programu ya elimu inayoshirikisha wanawake na wanaume kuongeza uelewa kuhusu jinsia, ukatili wa kijinsia, kupunguza ukubali wa ukatili wa kijinsia, na kuwashirikisha wanaume katika kusitisha ukatili dhidi ya wanawake na watoto?	NDIO . . . . 1<br>HAPANA . . . . .2<br>SIJUI 8	➢ 704a<br>➢ 704a
703b	Je umeshawahi kushiriki kwenye kikao/vikao vya programu ya hio elimu kuhusu jinsia na ukatili wa kijinsia?	NDIO NIMESHIRIKI . . . . 1<br>SIJAWAHI KUSHIRIKI . . . . .2<br>SIJUI 8	➢ 704a<br>➢ 704a
703c	Ni vikao vingapi vya programu ya elimu hiyo ya jinsia na ukatili wa kijinsia umeshiriki?	[ ] [ ]	
704a	Je umewahi kusikia kuhusu"Couples Connect," ambayo ni programu ambayo inafanya kazi na wenza/wachumba kuboresha jinsi wanaume na wanawake wanavyohusiana (kwa mfano waume na wake) katika kuwasiliana?	NDIO . . . . .1<br>HAPANA . . . . 2<br>SIJUI 8	➢ 705a<br>➢ 705a
704b	Ni mara ngapi umeshiriki katika "wenza walioshikamana"(Couples Connect)?	MARA NYINGI . . . . .1<br>MARA CHACHE . . . . 2<br>MARA MOJA . . . . 3<br>SIJAWAHI .4<br>SIJUI/SIKUMBUKI 8	
705a	Je umewahi kusikia majadiliano ya kijamii—kwamba, mikutaniko iliyoitishwa na jamii kwa ajili ya wanajumuiya kukaa pamoja kujadili masuala ya ukatili wa kijinsia au ukatili dhidi ya wanawake na watoto?	NDIO . . . . .1<br>HAPANA . . . . .2<br>SIJUI 8	➢ 706a<br>➢ 706a
705b	Ni mara ngapi umeshiriki katika majadiliano kama hayo?	MARA NYINGI . . . . .1<br>MARA CHACHE . . . . 2<br>MARA MOJA . . . . 3<br>SIJAWAHI .4<br>SIJUI/SIKUMBUKI 8	
706a	Je, katika jamii unayoishi kuna kamati zinazojihusisha na kuratibu shughuli za ukatili wa kijinsia au ukatili dhidi ya wanawake na watoto?	NDIO . . . . .1<br>HAPANA . . . . 2<br>SIJUI 8	➢ 707a<br>➢ 707a

ID: [ ] [ ] [ ] [ ] [ ] [ ] [ ]

MASWALI na CHUJAJI		UFUNGUO WA MAKUNDI	NENDA
706b	Je, wanachama wa kamati hizo za kuratibu shughuli za ukatili wa kijinsia wanashughulika na nini?<br><br>**USIMSOMEE; WEKA ALAMA YA TIKI KWA YOTE ATAKAYOTAJA**	a. [ ] Kusaidia wahanga wa ukatili wa kijinsia kufika katika kituo cha afya<br>b. [ ]Kifikisha kesi za ukatili wa kijinsia polisi<br>c. [ ] Kutatua kesi za ukatili wa kijinsia<br>d. [ ]Kuelimisha jamii kuhusu ukatili wa kijinsia<br>e. [ ]Nyingine, Taja ______________	
707a	Je unafahamu chochote kuhusu kampeni zozote za vyombo vya habari vya sasa au hivi karibuni (kama vile, matangazo, mashindano, redio, au ujumbe wa TV), vinayoelezea kuhusu ukatili wa kijinsia au ukatili dhidi ya wanawake na watoto?	NDIO . . . . 1<br>HAPANA 2	 ➢ 708
707b	Je kampeni hiyo ama kampeni hizo zina/zilikuwa na jina au kauli mbiu yoyote? Je unaweza kuniambia jina/majina?<br><br>**USIMSOMEE MAJIBU; WEKA ALAMA YA TIKI SEHEMU INAYOHUSIKA**	a. [ ] R AMETAJA “AMKA SASA”<br>b. [ ]R AMETAJA “KUWA MFANO WA KUIGWA”<br>c. [ ]R AMETAJA MAJINA MENGINE ZAIDI YA “AMKA SASA” AU “KUWA MFANO WA KUIGWA”<br>d. [ ]R AMEONYESHA KAMPENI KUTOKUWA NA JINA<br>e. [ ]R AMEONYESHA KAMPENI KUWA NA JINA ILA HALIKUMBUKI	
708	Sasa ningependa kukuuliza kuhusiana na shughuli nyingine za kijamii ambazo zinaweza kuwa **zimehusisha taarifa dhidi ya ukatili wa kijinsia au ukatili kwa wanawake na watoto.** Nitasoma matukio na ningependa wewe kunieleza kama ulishiriki katika miaka 2 iliyopita, na kama ulishiriki ni mara ngapi.<br>SOMA MAKUNDI YOTE NA ULIZA KUHUSU NI MARA NGAPI.		
	g. Matukio yanayoendeshwa wakati wa matukio maalum, ya kitaifa na kimataifa (kwa mfano, NaneNane, SabaSaba, Siku ya UKIMWI duniani, Siku ya Wanawake Duniani n.k)	Ameshiriki:<br>MARA NYINGI. . . . . 1<br>MARA CHACHE . . . . 2<br>MARA MOJA (Ktk miaka 2 iliyopita).3<br>SIJAWAHI. . . . . .4<br>SIJUI/SIKUMBUKI 8	 <br><br><br>➢ 708c<br>➢ 708c

ID: [ ][ ][ ][ ][ ][ ][ ]

MASWALI na CHUJAJI		UFUNGUO WA MAKUNDI	NENDA
	h. Je, kuna taarifa yoyote kuhusiana na ukatili wa kijinsia au ukatili dhidi ya wanawake ilitolewa au kujadiliwa?	NDIO . . . . .1<br>HAPANA . . . . .2<br>SIJUI/SIKUMBUKI 8	
	i. Shughuli za kimichezo za kijamii?	Ameshiriki:<br>MARA NYINGI. . . . . 1<br>MARA CHACHE . . . . 2<br>MARA MOJA (Ktk miaka 2 iliyopita).3<br>SIJAWAHI. . . . . .4<br>SIJUI/SIKUMBUKI 8	 <br><br><br>➢ 708e<br>➢ 708e
	j. Je, kuna taarifa yoyote kuhusiana na ukatili wa kijinsia au ukatili dhidi ya wanawake ilitolewa au kujadiliwa?	NDIO . . . . .1<br>HAPANA . . . . .2<br>SIJUI/SIKUMBUKI 8	
	k. Shughuli za kijamii au shughuli za kijamii kwa kutumia bodaboda?	Ameshiriki:<br>MARA NYINGI. . . . . 1<br>MARA CHACHE . . . . 2<br>MARA MOJA (Ktk miaka 2 iliyopita) 3<br>SIJAWAHI. . . . . .4<br>SIJUI/SIKUMBUKI 8	 <br><br><br>➢ 709<br>➢ 709
	l. Je, kuna taarifa yoyote kuhusiana na ukatili wa kijinsia au ukatili dhidi ya wanawake ilitolewa au kujadiliwa?	NDIO . . . . .1<br>HAPANA . . . . .2<br>SIJUI/SIKUMBUKI 8	
709	Je, umewahi kusikia au kuona ujumbe wa umma unao walenga wanaume katika vilabu vya pombe kuhusiana na kuacha ukatili dhidi ya wanawake na watoto? Kama ndivyo, mara ngapi?	MARA NYINGI. . . . . 1<br>MARA CHACHE . . . . 2<br>MARA MOJA . . . . 3<br>SIJAWAHI. . . . . .4<br>SIJUI/SIKUMBUKI 8	
709a	Je katika jamii yako kuna waelimishaji jamii au wahudumu wa jamii wa kujitolea wanaosaidia wahanga wa ukatili wa kijinsia kupata huduma wanazozihitaji?	NDIO . . . . 1<br>HAPANA . . . . 2<br>SIJUI 8	 ➢ 710<br>➢ 710

ID: [ ][ ][ ][ ][ ][ ][ ]

MASWALI na CHUJAJI		UFUNGUO WA MAKUNDI	NENDA
709b	Je wanawapeleka au wanawapa rufaa kwenye huduma zipi?<br><br>**USIMSOMEE; WEKA ALAMA YA TIKI KWA YOTE ATAKAYOTAJA**	a. [ ] Kituo cha afya<br>b. [ ] Polisi<br>c. [ ] Ustawi wa jamii<br>d. [ ] Viongozi wa kata au kiongozi mwengine wa jamii<br>e. [ ] Viongozi wa dini<br>f. [ ] Nyingine<br>TAJA:<br>______________________	
710	Tofauti na tulivyoongea tayari, katika kipindi cha miaka 2 iliyopita, je umesikia kuhusiana na shughuli zingine zozote katika jamii yako zinazolenga kusitisha ukatili wa kijinsia au ukatili dhidi ya wanawake na watoto?	NDIO . . . . 1<br><br>a.[ ]TAJA SHUGHULI: ____________<br><br>b.[ ] TAJA SHUGHULI: ___________<br><br>c.[ ] TAJA SHUGHULI: ____________<br><br>d.[ ] TAJA SHUGHULI: ___________<br><br>HAKUNA .2	 <br><br><br><br><br><br><br><br><br>➢**Kipengele cha 8**

ID: [ ][ ][ ][ ][ ][ ][ ]

MASWALI na CHUJAJI		UFUNGUO WA MAKUNDI	NENDA
711	Mara ngapi umeshiriki katika shughuli hizo?<br><br>**KWA KILA SHUGHULI ILIYOTAJWA KATIKA SWALI 710 (a-d), NAKILI MARA NGAPI AMESHIRIKI.**	a.<br>MARA NYINGI. . . . . 1<br>MARA CHACHE . . . . 2<br>MARA MOJA . . . . .3<br>SIJAWAHI. . . . . .4<br>SIJUI/SIKUMBUKI . . . . 8<br><br>b.<br>MARA NYINGI. . . . . 1<br>MARA CHACHE . . . . 2<br>MARA MOJA . . . . .3<br>SIJAWAHI. . . . . .4<br>SIJUI/SIKUMBUKI . . . . 8<br><br>c.<br>MARA NYINGI. . . . . 1<br>MARA CHACHE . . . . 2<br>MARA MOJA . . . . .3<br>SIJAWAHI. . . . . .4<br>SIJUI/SIKUMBUKI . . . . 8<br><br>d.<br>MARA NYINGI. . . . . 1<br>MARA CHACHE . . . . 2<br>MARA MOJA . . . . .3<br>SIJAWAHI. . . . . .4<br>SIJUI/SIKUMBUKI 8	

Kipengele cha 8 : Uelewa na mtazamo, mila za kijinsia, na tabia za mabadiliko

MHOJAJI:

Sasa ningependa kukuuliza kuhusu baadhi ya ukatili katika jamii yako na mawazo yako kuhusiana na hayo.

MASWALI na CHUJAJI		UFUNGUO WA MAKUNDI	NENDA
➢ Uelewa wa matukio katika jamii na majibu ya Jumuiya.			
801a	Je kumewahi kuwa matukio yoyote katika jamii unayoishi ndani ya miezi 12 iliyopita ambapo mwanamke amekatiliwa kingono ama kijinsia na mume wake au mwenzi wake?	NDIO . . . . 1<br>HAPANA . . . . 2<br>SIJUI 8	 ➢ 802a<br>➢ 802a
801b	Ni matukio mangapi unayoyafahamu?<br><br>**NAKILI IDADI YA MATUKIO KAMA YALIVYOSEMWA NA MHOJIWA.**	IDADI YA MATUKIO [ ] [ ] [ ] [ ]<br><br>SIJUI 9 9 9 8	

ID: [ ] [ ] [ ] [ ] [ ] [ ] [ ]

802a	Je kumewahi kuwa na matukio yoyote katika jamii unayoishi ndani ya miezi 12 iliyopita ambapo mtoto amekatiliwa kingono?	NDIO . . . . 1<br>HAPANA . . . . 2<br>SIJUI 8	➢ 803<br>➢ 803
802b	Ni matukio mangapi unayoyafahamu?<br><br>**NAKILI IDADI YA MATUKIO KAMA YALIVYOSEMWA NA MHOJIWA.**	IDADI YA MATUKIO [ ] [ ] [ ] [ ]<br><br>SIJUI 9 9 9 8	
803	Kwa maoni yako, katika miezi 12 iliyopita, je jumuiya yako imefanya kazi nzuri katika kushughulikia matukio ya ukatili kutoka kwa wanaume/wenzi na ukatili wa kingono kwa watoto?	Kazi nzuri sana . . . . 1<br>Kazi nzuri . . . . 2<br>Kazi ya wastani . . . . 3<br>Kazi hafifu . . . . .4<br>Kazi hafifu sana . . . . 5<br>Sijui 8	
➢ Uelewa wa Sheria/Sera na hatua za hivi karibuni za viongozi wa serikali za mtaa kuhusu Ukatili			
804	Je, unafahamu sheria zozote za Tanzaniaau sera dhidi ya ukatili kwa wanawake na watoto?	NAFAHAMU/NAELEWA SANA . . . . . .1<br>WASTANI. . . . . .2<br>SIFAHAMU . . . . 3<br>SIJUI. 8	
805	Katika miezi 12 iliyopita, ni mara ngapi viongozi wa serikali za mitaa wameongea kwa uwazi au wamechukua hatua kushughulikia suala la ukatili wa kijinsia au ukatili dhidi ya wanawake au watoto?	MARA NYINGI. . . . . .1<br>MARA CHACHE. . . . . 2<br>MARA MOJA. . . . . 3<br>HAWAJAWAHI. . . . . 4<br>SIJUI. 8	

ID: [ ] [ ] [ ] [ ] [ ] [ ] [ ]

➢ Maelezo au utambuzi wa Unyanyasaji dhidi ya wanawake (Imetoholewa kwenye DHS na utafiti wa CHAMPION)

806	Kwa maoni yako, je, unachukulia hali hizi ni unyanyasaji dhidi ya mwanamke kama mume wake...	**NDIO**	**HPN**	**SIJUI**
	a. Akifanya kitu kumdhalilisha mbele ya wengine	1	2	8
	b. Akitishia kumdhuru au kumdhuru mtu wa jirani	1	2	8
	c. Akimtukana au akimfanya ajisikie vibaya	1	2	8
	d. Akimsukuma, kumpiga makofi, kumpiga ngumi au teke	1	2	8
	e. Akitishia kumdhuru kwa kisu, bunduki au silaha yoyote	1	2	8
	f. Akimlazimisha kwa nguvu, kufanya naye ngono hata kama akiwa hataki	1	2	8
	g. Akimlazimisha kufanya kitu fulani cha kingono ambacho hataki kufanya	1	2	8
	h. Akizuia matembezi yake	1	2	8
	i. Akimfukuza nyumbani	1	2	8
	j. Akiwa na mapenzi nje ya ndoa	1	2	8
	k. Akiwa anafanya maamuzi yote kuhusiana na jinsi mapato ya familia yanavyotumika ikiwa ni pamoja na mapato ya mke wake	1	2	8
807	Kwa maoni yako, unachukulia haya kama unyanyasaji dhidi ya mwanamke kama mtu mwingine tofauti na mume wake...	**NDIO**	**HPN**	**SIJUI**
	a. Akifanya kitu kumdhalilisha mbele ya wengine	1	2	8
	b. Akitishia kumdhuru au kumdhuru mtu wa jirani	1	2	8
	c. Akimtukana au akimfanya ajisikie vibaya	1	2	8
	d. Akimsukuma, kumpiga makofi, kumpiga ngumi au teke	1	2	8
	e. Akitishia kumdhuru kwa kisu, bunduki au silaha yoyote	1	2	8
	f. Akimlazimisha kwa nguvu, kufanya naye ngono hata kama akiwa hataki	1	2	8
	g. Akimlazimisha kufanya kitu fulani cha kingono ambacho hataki kufanya	1	2	8

ID: [ ][ ][ ][ ][ ][ ][ ]

➢ Maelezo au utambuzi wa Unyanyasaji dhidi ya watoto				
808	Nina kwenda kusoma baadhi ya kauli na ningependa uniambie kama wewe unakubaliana na hiyo kauli, unakubaliana kidogo, au hukubaliani.	**NAKUBALI**	**NAKUBALI KIDOGO**	**SIKUBALI**
	a. Mtoto anakua amekatiliwa kingono pale tu kitendo cha kujamiiana kimefanyika	1	2	3
	b. Mtoto anapokuwa amekatiliwa kingono, mara chache aliyemkatili ni mmoja wa familia	1	2	3
	c. Ni watoto wa kike tu ndio wanakatiliwa kingono	1	2	3
	d. Haiwezekani watoto wenye umri chini ya miaka 10 kufanyiwa ukatili wa kingono (sexually abused)	1	2	3
	e. Watoto wanaotoka kwenye familia zinazojiweza hawana uzoefu na ukatili wa kingono	1	2	3
➢ Mitazamo kuhusu ukatili ndani ya ndoa/majumbani (kutoka DHS)				
809	Katika mtazamo wako, je mume anaruhusiwa kumpiga mke wake katika matukio yafuatayo:	**NDIO**	**HPN**	**SIJUI**
	a. Kama akitoka bila kumpa taarifa mumewe/mpenzi wake?	1	2	8
	b. Kama akitelekeza watoto?	1	2	8
	c. Kama akibishana na mumewe/mpenzi wake?	1	2	8
	d. Kama akikataa kufanya ngono na mumewe/mpenzi wake?	1	2	8
	e. Kama akiunguza chakula?	1	2	8
➢ Mila kuhusu jinsia (Kipimo cha GEM: Ukatili na vidokezo vyake)				
810	Sasa nitasoma sentensi na ningependa unieleze kama unakubaliana nazo, unakubaliana kidogo au hukubaliani nazo.	**NAKUBALI**	**NAKUBALI KIDOGO**	**SIKUBALI**
	a. Kuna muda mwanamke anastahili kupigwa.	1	2	3
	b. Mwanamke anabidi kuvumilia unyanyasaji ili kutunza familia yake pamoja.	1	2	3
	c. Ni sawa kwa mwanaume kumpiga mke wake kama si mwaminifu.	1	2	3
	d. Mwanaume anaweza kumpiga mke wake kama anakataa kufanya naye ngono.	1	2	3
	e. Kama mtu akimtukana mwanaume, lazima alinde heshima yake hata kama ni kwa kutumia nguvu.	1	2	3
	f. Mwanaume anayetumia nguvu kwa mkewe ni suala binafsi ambalo halitakiwi kujadiliwa nje yao wao wawili.	1	2	3
➢ Mila kuhusu jinsia (Kipimo cha GEM: Shughuli za ndani na umilikaji vitu)				
811	a. Kubadili nepi, kumwosha, na kumlisha mtoto ni jukumu la mama.	1	2	3
	b. Jukumu la mwanamke ni kuitunza familia yake.	1	2	3
	c. Mume ndio anatakiwa kuamua kufanya manunuzi ya vitu muhimu vya kaya.	1	2	3

ID: [ ][ ][ ][ ][ ][ ][ ]

	d. Mwanaume ndio anatakiwa awe na uamuzi wa mwisho kuhusiana na maamuzi nyumbani.	1	2	3
	e. Mwanamke anatakiwa kumuheshimu mume wake katika kila kitu.	1	2	3

➢ Mabadiliko ya tabia ya Mhojiwa: Kuchukua hatua.

812a	Katika miezi 12 iliyopita, je umeanzisha mazungumzo juu ya ukatili wa kijinsia au ukatili dhidi ya wanawake na watoto na mtu yeyote?	Ndio . . . . 1<br>Hapana 2	 ➢ 813a
812b	Ulifanya haya mazungumzo na nani?<br><br>NAKILI YOTE ATAKAYOTAJA.	a. [ ] Mpenzi/wapenzi<br>b. [ ] Rafiki<br>c. [ ] Mwanafamilia<br>d. [ ] Jirani au mwanajumuiya<br>e. [ ] Mwingine, taja ____________________<br>f. [ ] Mwingine, taja ____________________	
813a	Katika miezi 12 iliyopita, je wewe mwenyewe umeshashuhudia vitendo vyovyote vya ukatili wa kijinsia au ukatili dhidi ya wanawake au watoto au umeshawahi kukutana na mtu ambaye amefanyiwa huu ukatili?	Ndio . . . . 1<br>Hapana 2	 ➢ 814
813b	Je, ulichukua hatua yoyote kuzuia au kumsaidia mhanga? (Kwa mara zozote ulizoshuhudia)?	Ndio . . . . 1<br>Hapana 2	 ➢ 814
813c	Ni hatua gani ulizochukua?<br><br>**NAKILI YOTE ATAKAYOTAJA, USISOME!.**	a. [ ] Nilimsikiliza mhanga na kumpatia usaidizi wa kihisia.<br>b. [ ] Nilimpatia taarifa kuhusu huduma na programu kwa ajili ya wahanga.<br>c. [ ] Nilimsindikiza mhanga kwenda kliniki au kwa ajenti wa usaidizi.<br>d. [ ] Niliongea na wanafamilia wa mhanga kuhusiana na unyanyasaji.<br>e. [ ] Nilijaribu kuzuia au nilizuia unyanyasaji<br>f. [ ] Nilitoa taarifa kwenye mamlaka<br>g. [ ] Nyingine, taja ____________________	
814	Je una mtazamo tofauti <u>sasa</u> kuhusu ukatili wa kijinsia au ukatili dhidi ya watoto na wanawake tofauti na ulivyokuwa unafikiria miezi 12 iliyopita?	Ndio . . . . .1<br>Hapana 2	
815	Kama ungeshududia unyanyasaji leo je ungekuwa na uwezo zaidi, au usingekuwa na uwezo wa kuchukua hatua kama ambavyo ungekuwa miezi 3 iliyopita?	UWEZEKANO MKUBWA . . . . 1<br>VILEVILE TU . . . . 2<br>UWEZEKANO MDOGO 3	

ID: [ ][ ][ ][ ][ ][ ][ ]

Kipengele cha 9: Kumaliza Mahojiano

901	**MHOJAJI:**<br>Tunaelekea mwisho wa mahojiano yetu. Kuna hatua moja tu ya mwisho.<br>Nitakupatia kadi. Katika kadi hii kuna picha mbili. Hakuna taarifa nyingine yoyote iliyopo kwenye kadi hii. Picha moja ni ya furaha na picha nyingine ni ya huzuni.<br>**MPE MHOJIWA KADI NA KALAMU.**<br>Bila kujali kile ulichoniambia, ningependa uweke alama kwenye picha isiyo na furaha kama kuna mtu alikuumiza **WEWE** kimaumbile, alikugusa kingono/kimapenzi, au alikulazimisha kufanya kitu chochote cha kingono/kimapenzi ambacho hukuridhia katika miezi 12 iliyopita.<br>Tafadhali weka alama kwenye picha yenye furaha kama hili halikukutokea katika miezi 12 iliyopita. Ukisha weka alama kwenye kadi, ikunje na kisha iweke ndani ya mfuko huu. Mfuko huu una kadi sawasawa na hii kutoka kwa wanawake wengi wengine. Hii itahakikisha sitajua jibu lako.<br>USIANGALIE JIBU, MWOMBE MHOJIWA KUWEKA ALAMA NA KUWEKA KADI ILIYOKUNJWA KWENYE MFUKO ULIO NA KADI NYINGINE. USIWEKE ALAMA YOYOTE KWENYE KADI	KADI IMEKAMILIKA……..1<br>KADI HAIJAKAMILIKA…..2
902	Nimekuuliza mambo mengi magumu. Je, kuongelea mambo haya kumekufanya ujisikie vipi?<br>**SOMA MAJIBU**	VIZURI/VIZURI ZAIDI . . . . 1<br>VIBAYA/VIBAYA ZAIDI . . . . .2<br>VILEVILE/ HAKUNA TOFAUTI 3
903	Sasa tumemaliza mahojiano. Je una maoni yoyote, au kuna chochote ambacho ungependa kunishirikisha?<br>**NAKILI MAONI**	

ID: [ ][ ][ ][ ][ ][ ][ ]

904	**HITIMISHO**<br><br>Ningependa kukushukuru sana kwa kutusaidia. Na ninashukuru pia kwa muda uliojitoa. Ninatambua kuwa maswali haya yameweza kuwa magumu kwako, lakini ni kwa kusikia tu kutoka kwa wanawake wenyewe ndio tunaweza kuelewa uhalisia wa afya yao na uzoefu wa maisha yao.<br><br>Kwa mfano kama utakuwa na nia au kama ukisikia kuhusiana na mtu mwingine atakayehitaji msaada kuhusiana na masuala ya unyanyasaji na vitu ambavyo tumekuwa tukiongele, nitakuachia jina na mawasiliano ya mtoa huduma katika kituo afya cha __________________ au Hospitali ya Center/_______________ambaye atakupa usaidizi. Mtu huyu ni mtoa huduma katika kituo hiki cha afya ambaye pia anafanya kazi na sisi katika utafiti huu. Yeye anatambua kuwa tunatoa taarifa hii kama sehemu ya Mahojiano. Kama ukienda unahakikishiwa atatunza chochote utakachosema atakitunza kwa usiri. Unaweza kwenda muda wowote utakapojisikia uko tayari, hivi karibuni hata baadaye.<br><br>**MWACHIE KARATASI YENYE JINA LA KITUO CHA AFYA KATIKA KUNDI LA UTAFITI, JINA LA MHUSIKA WA TATHMINI GBV WA ENEO HILO, NA TAARIFA YA MAWASILIANO**.<br><br>Kama ungependa pia, ninaweza kumwomba mhudumu huyu wa afya awasiliane nawe moja kwa moja. Na kama ungependa uelekezo/rufaa hii, nitachukua taarifa ya mawasiliano yako, pamoja na jina lako na sehemu salama au faragha ambapo ungependa mtaalamu mwingine aweze kukukuta. Nitanakili taarifa hii kwenye karatasi tofauti, ambayo haitahusishwa kwa namna yoyote na mahojiano tuliyomaliza. Na nitampa taarifa hizi kiongozi wangu ambaye ataifikisha karatasi hii kwa mtoa huduma. Taarifa uliyonishirikisha kwenye mahojiano haitatolewa kwa mtoa huduma.<br><br>Je ungependa nikukutanishe moja kwa moja na Mtoa huduma?<br>NDIO……………………..1<br>HAPANA……………….2<br><br>**KAMA MHOJIWA AKISEMA 'NDIO', JAZA KARATASI YA RUFAA, IWEKE KWENYE BAHASHA ILIYOANDALIWA/ISIYO NA ALAMA NA MPATIE KIONGOZI WA KIKUNDI.**

**MHOJAJI:**	Nakili muda wa kumaliza mahojiano: Saa [ ][ ]:[ ] [ ] (tumia saa 24)
	Mahojiano yamekamilika: _______Ndio, yamekamilika ______Hapana, Hayajakamilika<br><br>Kama mahojiano hayajakamilika, tafadhali toa sababu:<br><br>**Sahihi ya mhojaji:**<br>____

ID: [ ] [ ] [ ] [ ] [ ] [ ] [ ]
